# Integrated proteogenomic deep sequencing and analytics accurately identify non-canonical peptides in tumor immunopeptidomes

**DOI:** 10.1038/s41467-020-14968-9

**Published:** 2020-03-10

**Authors:** Chloe Chong, Markus Müller, HuiSong Pak, Dermot Harnett, Florian Huber, Delphine Grun, Marion Leleu, Aymeric Auger, Marion Arnaud, Brian J. Stevenson, Justine Michaux, Ilija Bilic, Antje Hirsekorn, Lorenzo Calviello, Laia Simó-Riudalbas, Evarist Planet, Jan Lubiński, Marta Bryśkiewicz, Maciej Wiznerowicz, Ioannis Xenarios, Lin Zhang, Didier Trono, Alexandre Harari, Uwe Ohler, George Coukos, Michal Bassani-Sternberg

**Affiliations:** 1 Ludwig Institute for Cancer Research, University of Lausanne, Agora Center, Rue du Bugnon 25A, 1005 Lausanne, Switzerland; 2 Department of Oncology, Centre hospitalier universitaire vaudois (CHUV), Rue du Bugnon 46, 1011 Lausanne, Switzerland; 3 Vital IT, Swiss Institute of Bioinformatics, Quartier Sorge, Bâtiment Amphipôle, 1015 Lausanne, Switzerland; 4 Max Delbrück Centre for Molecular Medicine in the Helmholtz Association, Institute for Medical Systems Biology, Hannoversche Straße 28, 10115 Berlin, Germany; 5 School of Life Sciences, École Polytechnique Fédérale de Lausanne (EPFL), Route Cantonale, 1015 Lausanne, Switzerland; 6 Swiss Institute of Bioinformatics, Quartier Sorge, Bâtiment Amphipôle, 1015 Lausanne, Switzerland; 7 Department of Genetics and Pathology, International Hereditary Cancer Center, Pomeranian Medical University, ul. Rybacka 1, 70-204 Szczecin, Poland; 8 International Institute for Molecular Oncology, Jakuba Krauthofera 23, 60-203 Poznań, Poland; 9 Poznan University of Medical Sciences, Fredry 10, 61-701 Poznań, Poland; 10 Genome Center Health 2030, Chemin de Mines 9, 1202 Genève, Switzerland; 11 Department of Training and Research, CHUV/UNIL Agora Center, Rue du Bugnon 25A, 1005 Lausanne, Switzerland; 12 Center for Research on Reproduction and Women’s Health, University of Pennsylvania, 421 Curie Boulevard, Philadelphia, PA 19104 USA; 13 Department of Obstetrics and Gynecology, University of Pennsylvania, 3400 Civic Center Boulevard, Philadelphia, PA 19104 USA; 14 Departments of Biology and Computer Science, Humboldt-Universität zu Berlin, Unter den Linden 6, 10099 Berlin, Germany

**Keywords:** Peptides, Mass spectrometry, DNA sequencing, RNA sequencing, Tumour immunology

## Abstract

Efforts to precisely identify tumor human leukocyte antigen (HLA) bound peptides capable of mediating T cell-based tumor rejection still face important challenges. Recent studies suggest that non-canonical tumor-specific HLA peptides derived from annotated non-coding regions could elicit anti-tumor immune responses. However, sensitive and accurate mass spectrometry (MS)-based proteogenomics approaches are required to robustly identify these non-canonical peptides. We present an MS-based analytical approach that characterizes the non-canonical tumor HLA peptide repertoire, by incorporating whole exome sequencing, bulk and single-cell transcriptomics, ribosome profiling, and two MS/MS search tools in combination. This approach results in the accurate identification of hundreds of shared and tumor-specific non-canonical HLA peptides, including an immunogenic peptide derived from an open reading frame downstream of the melanoma stem cell marker gene *ABCB5*. These findings hold great promise for the discovery of previously unknown tumor antigens for cancer immunotherapy.

## Abstract (web summary)

Non-canonical HLA-bound peptides from presumed non-coding regions are potential targets for cancer immunotherapy, but their discovery remains challenging. Here, the authors integrate exome sequencing, transcriptomics, ribosome profiling, and immunopeptidomics to identify tumor-specific non-canonical HLA-bound peptides.

## Introduction

The efficacy of T cell-based cancer immunotherapy relies on the recognition of human leukocyte antigen (HLA)-bound peptides (HLAp) presented on the surface of cancer cells. Characterizing and classifying immunogenic epitopes is an ongoing endeavor for developing cancer vaccines and adoptive T cell-based immunotherapies. Neoantigens, peptides derived from mutated proteins, are absolutely tumor-specific yet mostly patient-specific, and are implicated in the efficacy of checkpoint blockade immunotherapy^1–4^. In contrast to tumor-specific private neoantigens, tumor-associated antigens (TAAs) that are shared across patients may be more attractive for immunotherapy due to the more efficient and rapid treatment of a greater number of patients^5–7^. Recent studies have focused on the discovery of non-canonical antigens, which are antigens derived from the aberrant translation of presumed non-coding transcripts and/or the aberrant or deregulated transcription of non-coding genomic regions, untranslated regions (UTRs), or genomically altered frames. Such aberrant transcription and translation events lead to the generation of peptide sequences that are missing in conventional protein sequence repositories^8,9^. If such translation events lead to the presentation of tumor-specific and immunogenic HLA ligands, these occurrences could substantially expand the repertoire of targetable epitopes for cancer immunotherapy^8–19^. Currently, ~1% of the entire genome is annotated as protein-coding regions, yet 75% of the genome can be transcribed and theoretically translated, potentially offering a pool of previously unexplored peptide targets^20^.

To date, mass spectrometry (MS) is the only analytical methodology that allows the direct identification of the HLAp repertoire in vivo^21^. Often, MS-based immunopeptidomic discoveries are limited to the standard, available protein sequence database, usually containing only annotated proteome-derived sequences. Recently, several studies have included protein sequences derived from the translation of transcripts identified from RNA-Seq, or ribosome profiling, in MS-based searches^8,22–27^. Overall, these studies warrant further development regarding many key aspects. Importantly, elevated false discovery rates (FDRs) for the non-canonical space can occur when MS reference data are populated with polypeptide sequences derived from all potential three- or six-frame translations of transcribed regions^28^. Several studies did not compute FDRs, while others applied sample-specific thresholds for FDR calculation^23,27^. Furthermore, rigorous experimental confirmations of such non-canonical sequences by targeted MS is currently lacking. Additionally, current workflows often introduce a risk of bias by pre-filtering peptide identification based on HLA binding predictions^23,27^. Owing to the above limitations and to an a priori restriction of the search space to tumor-specific non-canonical polypeptide sequences^23^, the overall biogenesis of non-canonical HLA-binding peptides (noncHLAp) remains to date understudied.

Here, we describe a proteogenomic approach that allows identifying tumor-specific noncHLAp derived from the translation of presumed non-coding transcripts, such as from (long) non-coding genes (lncRNAs), pseudogenes, UTRs of coding genes, and transposable elements (TEs). We perform immunopeptidomics analyses while integrating tumor exome, bulk and single-cell transcriptome (scRNA-Seq), and whole translatome data. We then implement NewAnce, a new analytical approach for non-canonical element identification that combines two MS/MS search tools, along with group-specific FDR calculations to identify noncHLAp. Altogether, this approach unveils a large number of unique noncHLAp, highlighting the potential of this approach to increase the range of targetable epitopes in cancer immunotherapy.

## Results

### A comprehensive strategy for noncHLAp identification

**Fig. 1:**
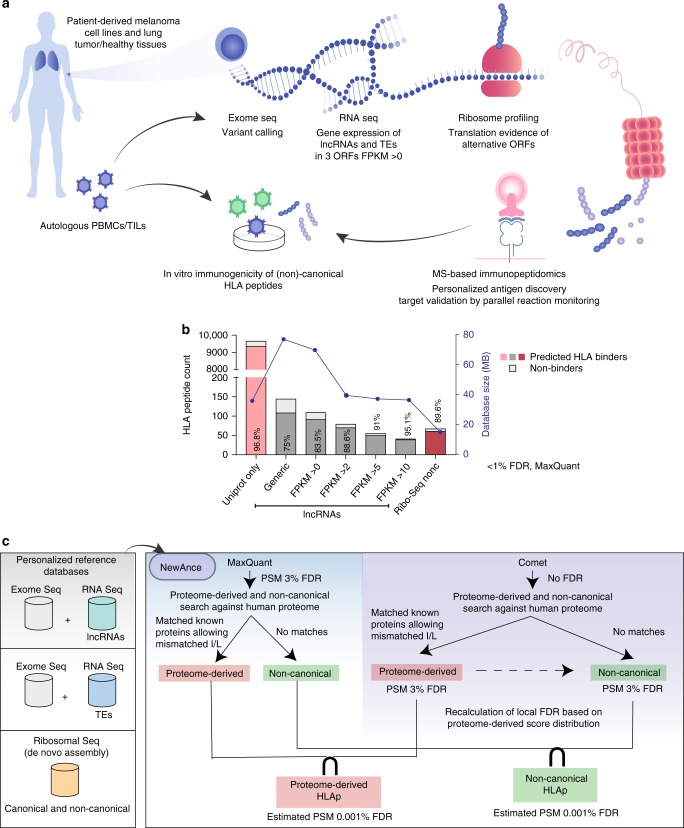
A proteogenomics approach for the robust identification of noncHLAp. **a** A schematic of the entire workflow is shown, where tissue samples or tumor cell lines were obtained from patients, and exome, RNA- and Ribo-Seq were performed to provide a framework to assess the non-canonical antigen repertoire. HLAp were immunoaffinity-purified from cancer cell lines and matched tumor/healthy lung tissues and then analyzed by MS. Immunopeptidomics spectra were then searched against RNA- and Ribo-Seq-based personalized protein sequence databases that contain non-canonical polypeptide sequences. MS-identified noncHLAIp were validated by targeted MS-based PRM and tested for immunogenicity using autologous T cells or PBMCs. **b** The percentage of predicted HLA binders of length 8–14 mer peptides with a MixMHCpred *p*-value ≤ 0.05 was used to evaluate the accuracy of the identified HLAIp by MaxQuant at 1% FDR as a function of database size (blue line). The percentage of predicted binders obtained for each condition is shown for each bar for the melanoma cell line 0D5P. **c** Different protein sequence databases combining whole-exome sequencing and inferences from RNA-Seq and Ribo-Seq data were utilized. NewAnce was implemented by retaining the PSM intersection of the two MS search tools MaxQuant and Comet, and applying group-specific FDR calculations for protHLAp and noncHLAp. Source data are provided as a Source Data file.

MS-based immunopeptidomics was performed on seven patient-derived melanoma cell lines and two pairs of lung cancer samples with matched normal tissues (Fig. 1a), which resulted in the identification of 60,320 unique proteome-derived HLA class-I bound peptides (protHLAIp) and 11,256 proteome-derived HLA class-II bound peptides (protHLAIIp). For the exploration and identification of non-canonical peptides presented naturally in vivo, whole-exome and RNA-Seq data were generated from all samples (Fig. 1a and Supplementary Data 1). We inferred expression of presumed non-coding genes, such as lncRNAs, pseudogenes and other non-protein-coding genes, from individual samples’ RNA-Seq data. In addition, we applied an analytical pipeline to assign TE-derived RNA-Seq reads to single loci (see Methods section for more details), resulting in expression data for transcribed TEs. All three forward open reading frames (ORFs) (stop-to-stop) in the above transcripts were subsequently in silico translated into polypeptide sequences. For every sample, the polypeptide sequences were concatenated to personalized canonical proteome references containing allelic variant information from patient tumor exome data. Finally, we searched the MS immunopeptidomics data against these personalized reference databases.

### Database size affects false positives in noncHLAp detection

In silico translation of transcripts in three forward reading frames results in a large number of potential polypeptide sequences. In proteogenomics, searching MS data against such inflated protein reference databases may propagate false positives^28,29^. Hence, our first investigative step was to understand the impact of database size on the level of false positives in immunopeptidomics datasets. We searched reference databases containing canonical (i.e., UniProt) and our non-canonical polypeptide sequences with a single search tool (MaxQuant) and at a global 1% FDR. The accuracy was assessed by assigning HLA binding prediction scores to the MS-identified peptides with MixMHCpred^30^. We reasoned that non-canonical HLA class-I bound peptides (noncHLAIp) should follow the same binding rules as protHLAIp^31^. First, we compared a generic non-canonical protein sequence database derived from the three forward frame (three-frame) translation of all non-coding transcripts from ENCODE^32^ with a sample-specific protein sequence database derived from the three-frame translation of lncRNAs and pseudogenes from the RNA-Seq data using an expression cutoff value of > 0 fragments per kilobase of transcript per million mapped reads (FPKM). Additional databases of decreasing size were assembled by retaining only the sequences that originated from more highly expressed genes (FPKM > 2, > 5 or > 10). Reducing the size of the database by personalizing and focusing on highly expressed genes led to an increase in the percentage of noncHLAIp that were predicted to bind to their respective HLA alleles (MixMHCpred *p*-value ≤ 0.05) (Fig. 1b). Restricting the database to polypeptide sequences originating from highly expressed genes should, on the one hand, improve the accuracy of MS-based non-canonical peptide identification, while on the other hand, lead to the potential loss of peptides encoded by lower-expressing transcripts. Hence, in this study, we included all non-coding transcripts with FPKM > 0 to circumvent the need to exclude polypeptide sequences based on low-expressing transcripts.

### NewAnce improves the accurate identification of noncHLAp

We developed the computational module NewAnce, which combines the MS search tools MaxQuant^33^ and Comet^34^, with the implementation of a group-specific strategy for the FDR calculation (see Methods section for more information and Supplementary Fig. 1a–g for performance evaluation). All HLAp identified by either of the search tools were consequently matched against an up-to-date UniProt/TrEMBL sequence database (95,106 protein sequences of the human reference proteome (up000005640), with isoforms) to extract noncHLAp that do not map back to known human proteins in UniProt. For every sample, FDRs were calculated separately for protHLAp and noncHLAp identified by Comet (Fig. 1c and Supplementary Fig. 1a). Only consensus (intersection) peptide-spectrum matches (PSMs) from Comet and MaxQuant were retained for further downstream analyses. Estimating the FDR after retaining the intersection is challenging. Nevertheless, most false-positive PSMs are specific to one search tool, and the remaining decoys in NewAnce indicated an estimated FDR of <0.001%.

With NewAnce, the number of protHLAIp identified across 11 samples ranged from 3490 to 16,672 per sample, and from 817 to 5777 for protHLAIIp (Supplementary Data 2). Furthermore, up to 148 noncHLAIp per individual sample were identified with NewAnce, with a combined total of 452 unique noncHLAIp (Supplementary Data 2 and Supplementary Data 3). Of note, noncHLAp are defined here as the peptides derived from either non-protein-coding genes, such as lncRNAs and pseudogenes, or TEs. As the majority of the non-protein-coding genes were lncRNAs, these will be henceforth collectively termed lncRNAs. Among the four HLA-II expressing samples investigated, only four non-canonical HLA class II bound peptides (noncHLAIIp) were detected out of 11,256 protHLAIIp. Re-searching the 2,597 PSMs of identified noncHLAp against the human proteome UniProt database concatenated with the list of non-canonical peptide sequences and allowing identification of alternative sequences, including six common modifications (see Methods section) revealed a very low level of ambiguity (Supplementary Data 4 and 5).

**Fig. 2:**
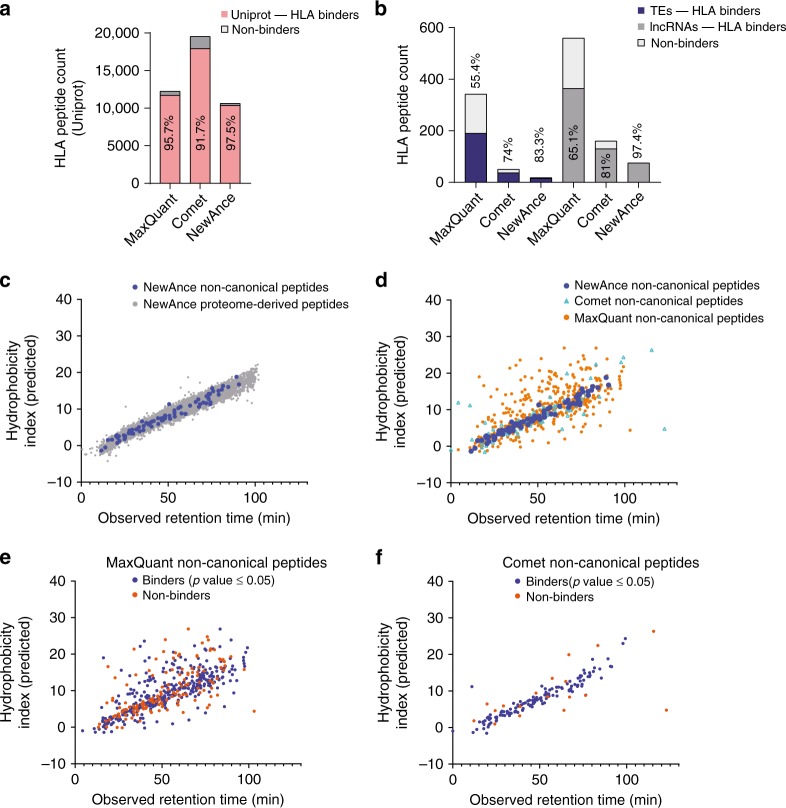
Two complementary methods to assess the accuracy of NewAnce. **a** The percentages of predicted proteome-derived HLA-I binders in 0D5P were assessed with each MS search tool (MaxQuant and Comet at FDR 3%) and NewAnce. **b** Similar to **a**, the comparisons were performed for the different non-canonical antigen classes. **c** Hydrophobicity index calculations by SSRCalc for peptides identified in melanoma 0D5P. The observed mean retention time is plotted against the hydrophobicity indices for NewAnce-identified proteome-derived versus lncRNA-derived non-canonical peptides. **d** All peptides identified with each tool (MaxQuant, Comet, NewAnce) were analyzed based on their hydrophobicity indices. **e** Hydrophobicity index calculation for MaxQuant- or **f** Comet-identified 8- to 14-mer peptides, based on predicted HLA binding. Source data are provided as a Source Data file.

We employed two complementary methods to assess the accuracy of our approach. First, we predicted the binding of peptides to their respective HLA allotypes. Across all 11 samples, 90% of the noncHLAIp and 91% of the protHLAIp identified with NewAnce were predicted to bind the HLA allotypes (median values, Supplementary Fig. 1h). As expected, NewAnce detected fewer HLAp than Comet (PSM FDR of 3%) or MaxQuant (PSM FDR of 3%), while with a more routinely applied FDR of 1% using MaxQuant alone, more HLAp were obtained with NewAnce (Fig. 2a, Supplementary Fig. 1i–l, and Supplementary Data 2). Importantly, for the noncHLAIp repertoire (lncRNAs and TEs), significantly higher percentages of peptides predicted to bind the HLA allotypes were identified by NewAnce than MaxQuant or Comet alone (Fig. 2b and Supplementary Fig. 1i–l).

In addition, we correlated the observed mean retention time (RT) of a given peptide against the calculated hydrophobicity index (HI), which corresponds to the percentage of acetonitrile at which the peptide elutes from the analytical high-performance liquid chromatography (HPLC) system. Calculating the sequence-specific hydrophobicity of peptides identified by NewAnce  with SSRCalc^35^ showed that the RT distribution of non-canonical peptides was on the diagonal line, and was not significantly different from the distribution of proteome-derived peptides, supporting their correct identification (Fig. 2c) (one-sided *F*-test *p*-value: 1.0e+0). However, a significant difference in RT distribution was observed when comparing non-canonical peptides identified by NewAnce to those identified by MaxQuant (one-sided *F*-test *p*-value: 6.3e−32) or Comet alone (one-sided *F*-test *p*-value: 8.4e−20) (Fig. 2d). Similar results were obtained for all investigated samples (Supplementary Fig. 2).

A common approach to boosting non-canonical peptide identifications is searching the MS data with a single tool (or the union of two tools) while applying a permissive FDR followed by an additional step of filtering to include only peptides predicted to bind the relevant HLA allotypes^36^. To evaluate this approach, we compared the correlation between the HIs and RTs of predicted non-canonical HLA binders and non-binders identified at 3% PSM FDR with either MaxQuant (Fig. 2e) or Comet (Fig. 2f). Predicted binders showed better correlations between the HI and RT than non-binders (one-sided *F*-test *p*-values: 8.4e-6 for MaxQuant and 4.4e-18 for Comet). These correlations were fairly poor for MaxQuant, while a much better correlation was calculated for Comet, likely due to the conservative group-specific FDR control strategy we applied for Comet.

Notably, when examining the source protein sequence origins of all noncHLAIp, we detected an enrichment towards the C-terminus of their precursor protein sequences. This effect was also observed for protHLAIp originating from similarly short canonical proteins (Supplementary Fig. 3a, b).

### Targeted-MS and Ribo-Seq confirm noncHLAIp detection

**Fig. 3:**
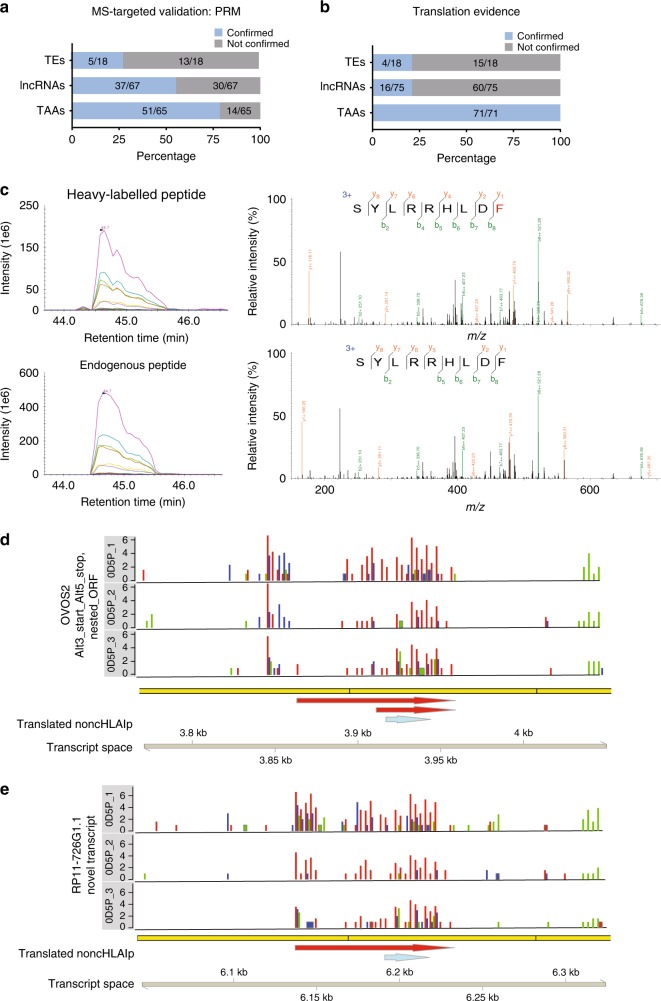
MS and ribosome footprint-based evidence of non-canonical peptide generation. A set of proteome-derived tumor-associated antigens, and noncHLAIp (lncRNAs and TEs), from melanoma 0D5P were synthesized in their heavy-labeled form and spiked back into replicates of HLAIp eluted from 0D5P cells to confirm the presence of endogenous HLAIp. The proportions of confirmed and non-confirmed HLAIp as determined by **a** PRM and **b** Ribo-Seq-targeted validation are shown for each of the antigen classes. **c** An example of the co-elution profiles of the transitions of heavy-labeled and endogenous noncHLAIp (from lncRNA; SYLRRHLDF) from 0D5P (left) is shown. The MS/MS fragmentation pattern further confirms the presence of the endogenous peptide (Δ*m* = 10 Da) (right). **d**, **e** The Ribo-Seq profiles of two source genes show the frequency of Ribo-Seq reads from the ribosome’s P-site in three replicates. Library size-normalized P-sites per basepair are shown on a log2 scale on the *y*-axis, with P-sites inferred as a constant offset from the 5ʹ end of the footprint for each read length. The colored bars represent different reading frames. The yellow bars below the plots represent exons. For example, the noncHLAIp SYLRRHLDF in *OVOS2* (blue arrow) falls within two nested, Ribo-Seq-supported ORFs (red arrows), within which most P-sites (red bars) fall in the first reading frame. Source data are provided as a Source Data file.

To experimentally validate the NewAnce computational pipeline, we investigated a selection of NewAnce-identified HLAp from a melanoma sample (0D5P) with targeted MS-based analyses. All identified noncHLAIp (lncRNAs and TEs, *n* = 93), as well as a similarly sized subset of protHLAIp from clinically relevant TAAs (*n* = 71) detected in 0D5P, were synthesized in their heavy isotope-labeled forms for MS-targeted validation. The selected TAAs were chosen solely based on their interesting tumor-associated biological functions, such as known cancer/testis or melanoma antigens. Here, MS-based targeted confirmation by parallel reaction monitoring (PRM) was directly compared between the non-canonical and proteome-derived peptide groups by spiking the heavy-labeled peptides into multiple independent replicates of 0D5P immunopeptidomic samples, revealing that protHLAIp confirmation was superior to that of noncHLAIp (78.5% for TAAs versus 55.2% for lncRNAs and 27.7% for TEs) (Fig. 3a, Supplementary Data 6, and Supplementary Data 7). We also observed that the PRM validation was dependent on the source RNA expression level (Supplementary Fig. 4a–d), measured peptide intensities (Supplementary Fig. 4e–h), and detectability by MS/MS across multiple 0D5P replicates (Supplementary Fig. 4i–l).

To further validate the noncHLAIp with an additional targeted strategy, we analyzed sample 0D5P also by Ribo-Seq, which involves the sequencing of ribosome protected fragments (RPFs). Periodic RPF distributions (see Methods section) that supported translation from the correct ORFs of the transcripts encoding the identified noncHLAIp were observed for 22.2% of the TE peptides and 21.3% of the lncRNA peptides, compared to 100% of the TAAs (Fig. 3b). Notably, nine lncRNA HLAIp and two TE peptides were validated independently by both the PRM and Ribo-Seq approaches. For example, the noncHLAIp SYLRRHLDF was confirmed by MS (Fig. 3c), and the translated ORF that generated the peptide was mapped back to two non-coding RNA transcripts (Fig. 3d–e).

### Low RNA expression limits noncHLAIp presentation

**Fig. 4:**
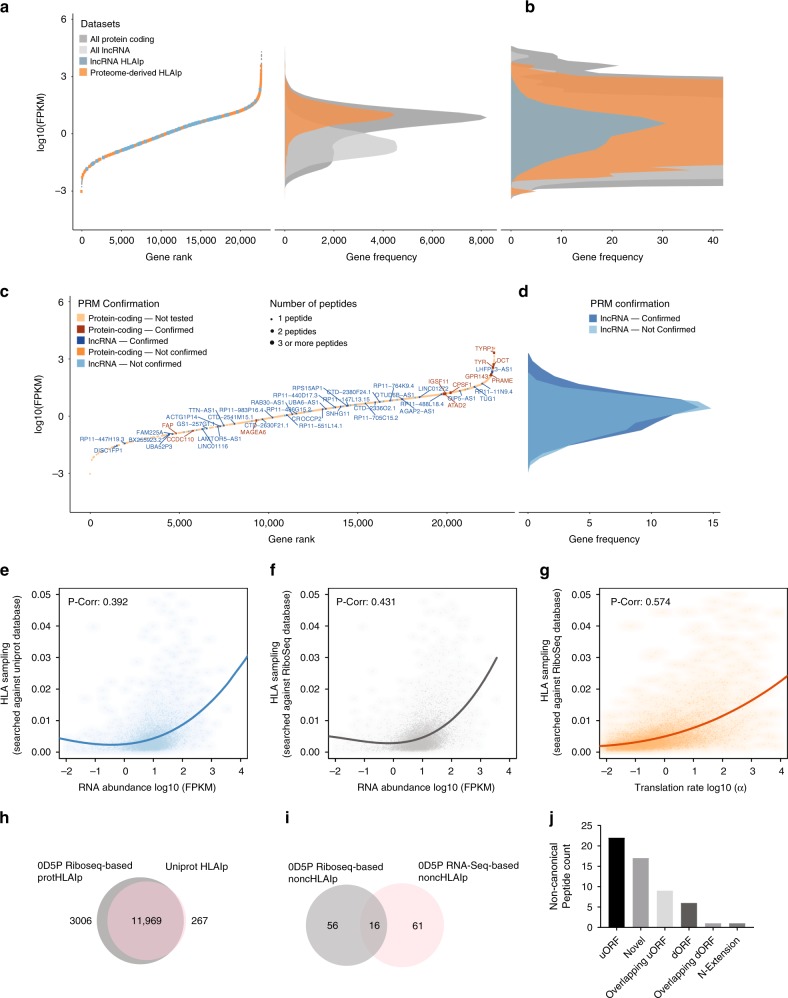
RNA- and Ribo-Seq-based gene expression analyses from melanoma 0D5P. **a** (Left panel) Genes are ranked based on their RNA expression levels in 0D5P, with protein-coding and presumed non-coding source genes, in which HLAIp were identified, marked in orange, or in blue, respectively. (Right panel) The frequency distributions of the gene expression levels of protein-coding and non-coding (lncRNA) genes are shown. **b** The region of interest is magnified to show the distribution of noncHLAIp source gene expression. **c** Plot restricted to source genes. Targeted MS validation was performed, and confirmations are denoted for all identified non-canonical peptides and for a subset of protHLAIp (selected TAAs). Confirmed hits indicate that one or more peptides from that source gene were validated by PRM. Point sizes represent the number of peptides identified per source gene. **d** Frequency distribution of gene expression for MS-confirmed versus non-confirmed (or inconclusive) noncHLAIp. Scatterplots show the correlation between **e** UniProt-based HLA-I sampling and RNA abundance**, f** Ribo-Seq-based HLA-I sampling and RNA abundance, and **g** Ribo-Seq-based HLA-I sampling and translation rate. HLA-I sampling was calculated from the adjusted peptide counts normalized by protein length. Determination of the correlation between gene expression and HLA-I sampling was assessed by fitting a polynomial curve of degree 3 to each dataset. Pearson correlation values were calculated to assess the correlation between the fitted curve and the corresponding dataset. **h** With data derived from 0D5P, a comparison of the overall overlap in unique HLAIp identified with RNA-Seq-based and Ribo-Seq-based assembled databases for MS search is shown. **i** Overlap of noncHLAIp identified by RNA-Seq- and Ribo-Seq-based searches. **j** The total number of noncHLAIp identified by Ribo-Seq is depicted for each of the respective ORF types. Source data are provided as a Source Data file.

We then characterized the expression levels of source RNAs encoding HLAIp in more depth. For this purpose, we compared all identified source genes of protHLAIp to source genes of noncHLAIp in the 0D5P sample. The protein-coding source genes had a median FPKM value of 9.3, whereas the presumed non-coding source genes showed lower expression overall, with a median FPKM of 2.1 (Fig. 4a, b). Generally, higher numbers of unique peptides identified per gene were correlated with higher expression levels. PRM-validated noncHLAIp covered a large dynamic range of gene expression, and interestingly, a few were confirmed at very low source RNA expression levels (Fig. 4c–d).

The low expression levels of source genes that generated noncHLAp prompted us to investigate the regulation of non-canonical HLA presentation and whether their expression can be induced or enhanced with drug treatments. We treated melanoma cells with either decitabine (DAC), a DNA methyltransferase inhibitor, known to reactivate epigenetically silenced genes, or with interferon gamma (IFNγ), known to upregulate antigen presentation^37–40^. As expected, we observed large quantitative changes in the presentation of protHLAIp when T1185B melanoma cells were treated with IFNγ. Specifically, we found enhanced presentation of peptides derived from immune-related genes, likely due to their high gene expression and increased production of HLA-I molecules (Supplementary Fig. 4m). However, no obvious change was observed for the noncHLAIp repertoire, with 60% of the identified noncHLAIp remaining unaltered by IFNγ treatment, suggesting that transcription is the limiting step in the presentation of noncHLAIp, or that transcription of noncHLAIp is not affected generally by IFNγ (Supplementary Fig. 4n). Furthermore, we explored the effect of the hypomethylating agent DAC on noncHLAIp in melanoma. Although DAC induced the expression of selected hypomethylating  agent-induced immune genes^41^, TAAs and non-coding transcripts (Supplementary Fig. 4o–q), changes in the 0D5P noncHLAIp repertoire were modest. Nonetheless, we identified and confirmed the presence of a unique DAC-induced noncHLAIp derived from a lncRNA (Supplementary Fig. 4r).

### Ribo-Seq improves the coverage of protHLAIp and noncHLAIp

Next, we hypothesized that immunopeptidomes would correlate more closely with translatome than transcriptome data. To build the translatome-based database for the MS search, all ORFs showing periodic RPF distribution were extracted for the 0D5P sample, and translated in silico. This technique reduced the size of the search space, and we used this independent discovery method in our study to identify additional noncHLAIp, including those derived from previously unexplored ORFs in coding genes.

We investigated the extent by which a protein sequence database inferred by Ribo-Seq could replace or complement the search performed with our personalized references comprising canonical protein sequences concatenated with polypeptidecsequences from the three-frame translation of expressed non-coding transcripts. Using 0D5P as a representative immunopeptidomic dataset, we observed a positive correlation between RNA expression and HLAIp-sampling (see Methods section) searched against the personalized protein sequence database (*r* = 0.392) (Fig. 4e). Then, we searched the same immunopeptidomics MS data against the de novo-assembled Ribo-Seq inferred database, and we correlated this HLAIp-sampling with RNA abundance (Fig. 4f) or with translation rates based on the spectral coefficient of the 3-periodic signal in the Ribo-Seq data (see Methods section) (Fig. 4g). This approach resulted in a significantly higher positive correlation between HLAIp-sampling searched against the Ribo-Seq inferred database and the translation rate (*r* = 0.574) than the overall RNA abundance (*r* = 0.431, two-sided *p*-value < 10e-16). Thus, evidence exists that the immunopeptidome, at least for the 0D5P sample, is better captured by the translatome than the transcriptome.

Notably, restricting the database to actual translation products detected by Ribo-Seq provided a deeper coverage of the immunopeptidome than a canonical protein sequence database (Fig. 4h). This enhanced coverage led to the identification of additional noncHLAIp derived mainly from ORFs that are not included in canonical annotation but still showing periodic footprint of translation, such as those originating in 5ʹ or 3ʹ UTRs, presumed non-coding RNAs, retained introns, and pseudogenes. The majority of those identified were derived from either upstream ORFs or other un-annotated ORFs (Fig. 4i–j). Many of these additional noncHLAIp were missed using the RNA-Seq inferred database. Of note, this method also takes into account products arising from ribosomal frameshifting, which could be relevant in the context of non-canonical antigens^42^. Interestingly, only 16 common lncRNA-derived noncHLAIp were found when comparing both strategies, which likely reflects the limited detection of periodic Ribo-Seq reads in transcripts with low expression or low mappability (Supplementary Fig. 5a–c).

### scRNA-Seq reveals heterogeneity in presumed non-coding genes

**Fig. 5:**
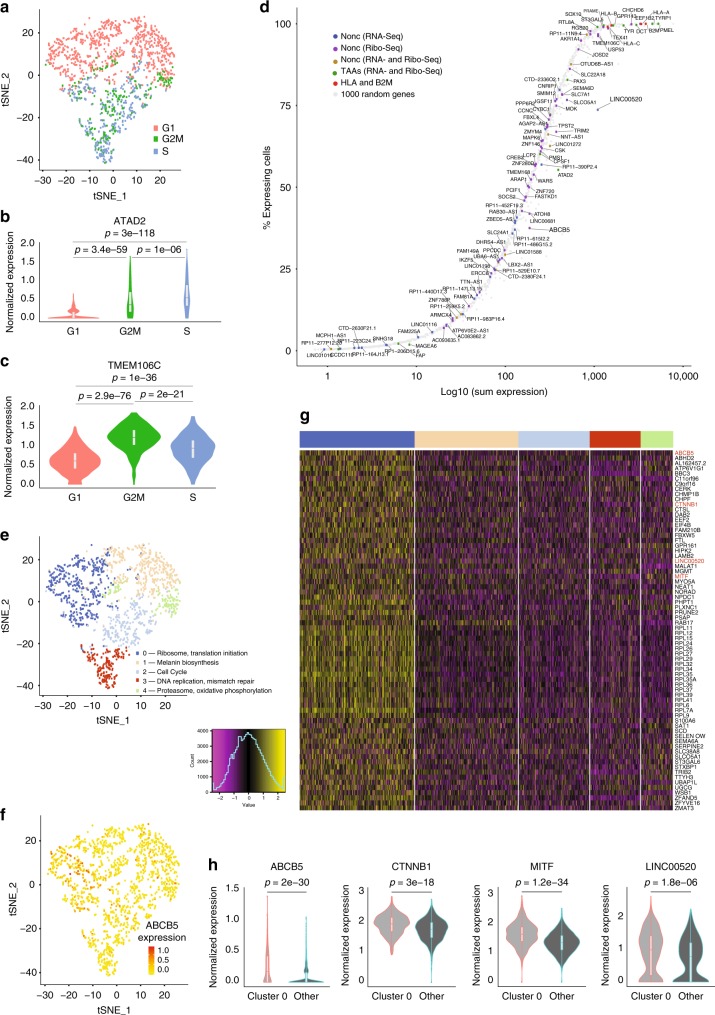
scRNA-Seq reveals non-coding transcriptional heterogeneity in melanoma 0D5P. **a** t-SNE plot of the 1365 cells colored by their “cell cycle” scores. **b** Examples of cell cycle dependent genes: *ATAD2*, a tumor-associated antigen, and **c** *TMEM106C*, from which a noncHLAIp originated. **d** Genes of interest were plotted based on their sum normalized expression by scRNA-Seq and ordered based on the percentage of cells that expressed the gene. The color codes denote the type of HLAIp identified from those genes. **e** t-SNE plot of the 1365 cells colored by the five identified clusters. Clusters were annotated based on functional enrichment analyses of marker genes. **f** t-SNE plot highlighting the expression of the *ABCB5* gene enriched in cluster 0. **g** Heatmap showing the scaled and centered expressions of marker genes in cluster 0. The cluster colors from **e** are represented above the plot. **h** Expression profiles of four marker genes in cluster 0 over all other clusters, including two well-known cancer biomarkers, *MITF* and *CTNNB1*, and two source genes for which noncHLAIp were identified, the *ABCB5* gene with a dORF and *LINC00520*. The *p*-values represented in **b**, **c**, and **h** were obtained with Wilcoxon tests. Source data are provided as a Source Data file.

Tumor cell heterogeneity could be a key factor underlying immune escape, leading to the inefficacy of cancer immunotherapies. To understand the pattern of non-coding gene expression at the single-cell level, we performed scRNA-Seq on the 0D5P melanoma cell line. Overall, 1400 cells were sequenced at a total depth of 176 million reads, resulting in the detection of a median of 6261 genes per cell (total of 19,178 detected genes). As expected, clustering of 0D5P cells revealed dependency on the cell cycle status (Fig. 5a), and thus we explored whether source genes associated with the cell cycle (Fig. 5b, c). Then, we confirmed that the antigen presentation machinery as well as many of the selected TAAs were uniformly expressed in all cells and were thus independent of the cell cycle, as expected (Fig. 5d).

Out of the 71 presumed non-coding source genes identified by bulk transcriptomics, 35 were also detected at the single-cell level (Fig. 5d). HLAIp derived from presumed non-coding source genes detected with higher coverage at the single-cell level were also those confirmed by PRM (6 out of 8 genes confirmed in >50% cells and 14 out of 27 genes confirmed in <50% cells) and by Ribo-Seq (37 out of 41 genes confirmed in >50% cells and 25 out of 46 genes confirmed in <50% cells). Importantly, source non-coding genes clearly showed expression heterogeneity: nearly none of them were uniformly expressed across cells, although the limited sensitivity of scRNA-Seq could account for this variation. The expression of *LINC00520* was higher than expected given its detection in only 75% of cells, suggesting that it is not uniformly expressed (Fig. 5d). Sufficient expression level in a subset of cells would allow the sampling for HLA presentation of overall lowly expressed genes and eventually their detection in the immunopeptidome.

We thus sought to explore the cell subset by identifying known biomarker genes co-expressed with *LINC00520* (Fig. 5e–h). Interestingly, we found that *LINC00520* was co-expressed with the ATP-binding cassette sub-family B member 5 (*ABCB5*) gene (Fig. 5g). The ABCB5 mediates chemotherapy drug resistance in stem-like tumor cell subpopulations in human malignant melanoma and is commonly over-expressed in circulating melanoma tumor cells^43^, together with beta-catenin (CTNNB1), a key regulator of melanoma cell growth^44^, and with its critical downstream target microphthalmia-associated transcription factor (MITF), which mediates melanocyte differentiation^45^ (Fig. 5h). *ABCB5* was detected in 37% of 0D5P cells (Fig. 5f), which also co-expressed *LINC005520*. Importantly, we also detected a noncHLAIp epitope encoded by a previously unknown ORF embedded within the *ABCB5* gene which, as shown below, is immunogenic. The detection of such non-canonical neoantigens in subsets of melanoma cells with regenerative or metastatic potential could prove highly interesting in the context of immunotherapy.

### Identification of tumor-specific noncHLAIp

**Fig. 6:**
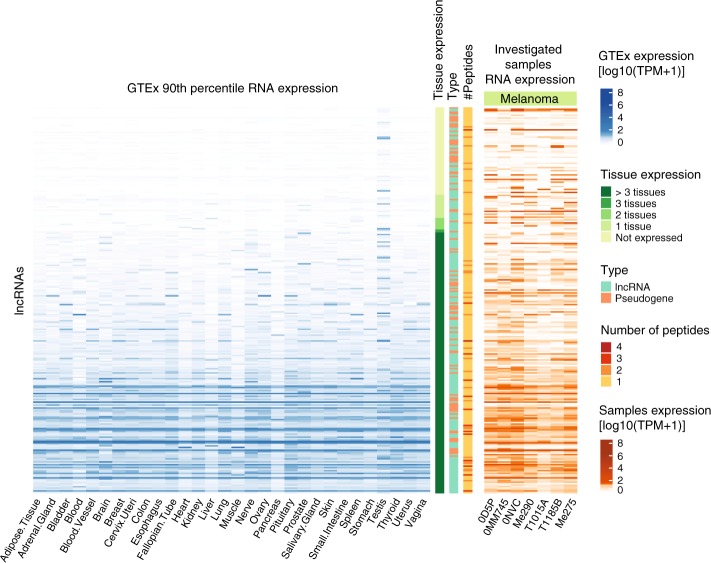
Non-coding source gene expression in healthy tissues. A comparison of presumed non-coding source gene expression in the investigated melanoma samples to that in healthy tissues (GTEx) reveals that a substantial proportion of source non-coding genes are tumor-specific. Heatmap of lncRNA source genes showing the 90th percentile gene expression levels across 30 healthy tissues on the left and the gene expression levels across our investigated melanoma samples on the right. Tissue gene expression was classified as not expressed (90th percentile TPM ≤ 1) in any, 1–3, or >3 tissues other than testis to assess tumor specificity. Specifically for sample 0D5P, a total of 21.4% of the lncRNA source genes were considered as tumor-specific compared to <1% of the randomly selected protein-coding source genes with similar expression levels (*p*-value = 1.04 e-33). The number of HLAIp identified per gene is depicted as well as the gene (GENCODE) and sample type. Source data are provided as a Source Data file.

As our initial MS search space was not restricted to polypeptide sequences derived from tumor-specific transcripts, we retrospectively investigated the potential of identified noncHLAIp to be classified as tumor-specific. A public database of RNA sequencing data from 30 different healthy tissues (Genotype-Tissue Expression, GTEx^46^) was assessed at a strict 90^th^ percentile, which sets the expression of a gene at the top 10% of its expression across all samples. We identified 335 noncHLAIp from 280 lncRNA genes in the seven melanoma samples, of which 23% were expressed in any of our tumor samples and not in the healthy tissues (excluding testis due to its immunoprivileged nature) (Fig. 6). Among these genes was the tumor-specific *LINC00518* gene, which has been proposed as a two-gene classifier for melanoma detection, together with the TAA *PRAME*^47^.

Using an in-house curated inventory of human TE-derived polypeptide sequences (from three-frame translations) as a reference, we found 88 unique TE-HLAIp in our whole dataset. Some were derived from autonomous TEs, such as long-tandem repeat (LTR) retrotransposons and long interspersed nuclear elements (LINEs), and others were derived from non-autonomous retrotransposons such as short interspersed nuclear elements (SINE) and SINE-VNTR-Alu (SVA) elements (Supplementary Fig. 6a). Importantly, 60 of the 88 TE-HLAIp were found in presumed non-coding TE regions and therefore represent previously unknown HLA peptides. For example, peptides derived from AluSq2 SINE/Alu and L1PA16 LINE/L1 elements were expressed in only skin and testis. These TE-HLAIp would have been overlooked in canonical MS-based searches.

**Fig. 7:**
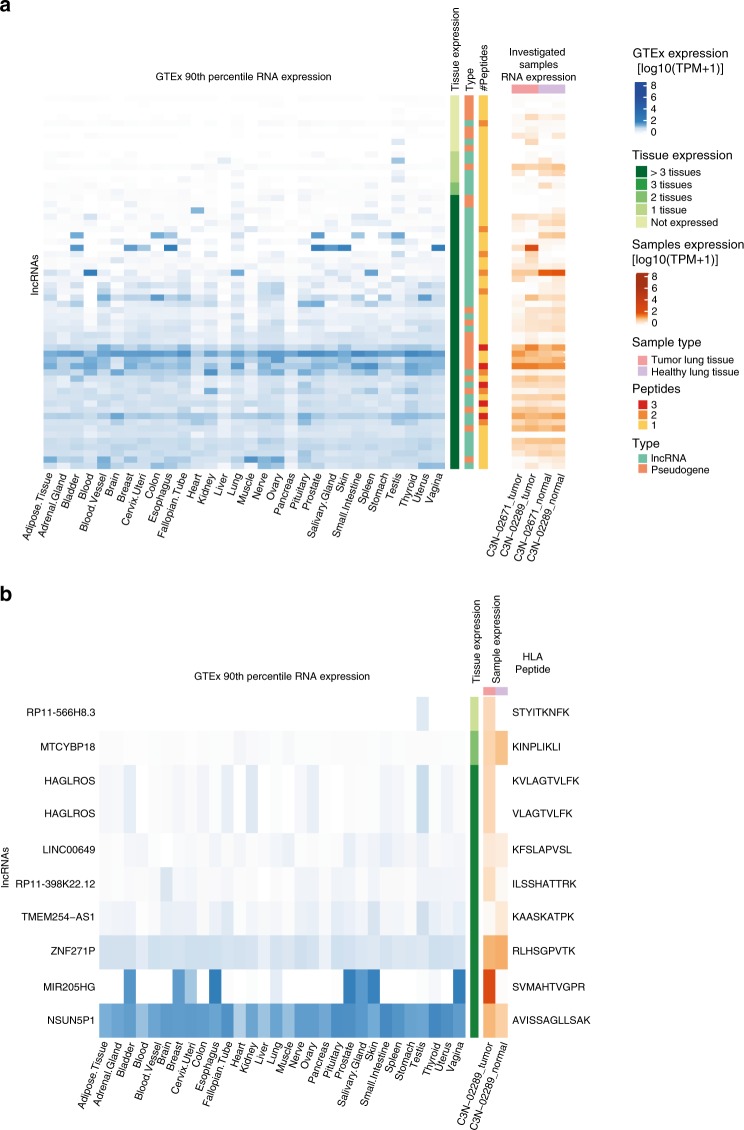
Non-coding source gene expression from lung cancer patient samples in healthy tissues. A comparison of presumed non-coding source gene expression in the investigated samples to that in healthy tissues (GTEx). **a** Heatmap of lncRNA source genes showing the 90th percentile gene expression levels across 30 healthy tissues on the left and the gene expression levels identified in lung tissue samples on the right. Tissue gene expression was classified as not expressed (90th percentile TPM ≤ 1) in any, 1–3, or >3 tissues other than testis to assess tumor specificity. The number of HLAIp identified per gene is depicted as well as the gene (GENCODE) and sample type. **b** Specifically, this was also plotted for the tumor-specific noncHLAIp identified in lung cancer patient C3N-02289. Source data are provided as a Source Data file.

We next examined whether our approach could identify tumor-specific non-canonical targets in the ideal case in which normal and tumor biopsies are available, i.e., from the two lung cancer patient samples included in the present dataset. For the C3N-02671 lung tumor sample, 21 noncHLAp were detected by MS; however, none of the peptides were tumor-specific. In the C3N-02289 sample, we identified 45 noncHLAIp by MS (Fig. 7a), among which ten peptides were identified uniquely in the tumor tissue. Four of these source genes were also entirely absent at the RNA level in the adjacent lung (Fig. 7b). Interestingly, the noncHLAIp from *RP11-566H8.3* was also testis-specific in the GTEx database (90^th^ percentile transcripts per million (TPM) ≤ 1) (Fig. 7b), thus qualifying as a non-canonical cancer/testis antigen.

The same analyses of TEs in lung tumor sample C3N-02289 resulted in the identification of one LTR7B LTR/ERV1 TE-HLAIp that was present in the tumor tissue; however, this gene is also expressed in healthy brain. For sample C3N-02671, no TE-derived HLAIp were detected.

To comparatively assess the expression of canonical tumor antigens in the same samples, we investigated select TAAs using the same methodology (Supplementary Fig. 6b, c). We identified six TAA protHLAIp that were exclusively detected in the tumor tissue of C3N-02289 (BIRC5, TERT, FAP, SPAG4, MAGEA9, and BCL2L1). We also detected uniquely in C3N-02671 tumor tissue two protHLAIp TAAs (CCND1 and PXDNL) and five protHLAIIp TAAs (MMP2 and CEACAM5).

### NoncHLAIp are shared across patient samples

**Fig. 8:**
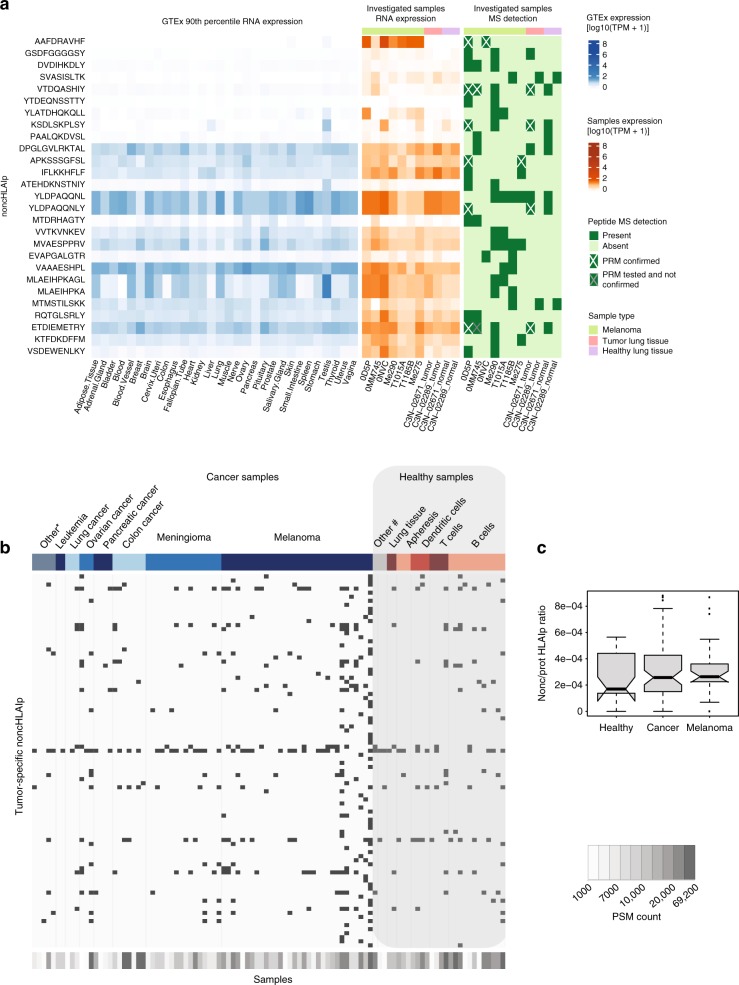
NoncHLAIp can be shared across individuals. **a** The noncHLAIp-centric heatmap (left) shows the corresponding presumed non-coding gene expression (90th percentile) across healthy tissues as well as in our investigated samples (middle). The peptides that were identified by MS across the investigated samples, and therefore shared, are outlined in the rightmost heatmap. Validation by PRM was performed for multiple noncHLAIp across the corresponding samples and are denoted with cross markings. **b** NoncHLAIp identified across a large collection of immunopeptidomics datasets (ipMSDB) consisting of both cancer and healthy samples. Tumor-specific noncHLAIp were re-identified and a trend of enrichment in cancer samples was observed. The noncHLAIp sequences can be found in the source data file. Cancer samples are labeled in shades of blue, and the star symbol include tumor metastases, myeloma, uterine, brain, and liver cancer. Healthy samples are indicated in shades of red, and the hashtag symbol include fibroblast cells and epithelial cells. **c** Boxplot depicting the ratio of noncHLAIp over protHLAIp identified in the different groups of samples derived from ipMSDB (healthy *n* = 27, cancer *n* = 63, melanoma *n* = 25) One-sided *t*-test was performed, without multiple testing correction. Healthy versus cancer *p*-value = 0.17, healthy versus melanoma *p*-value = 0.12. Please refer to the Methods section for boxplot parameters. Source data are provided as a Source Data file.

We investigated the prevalence of shared noncHLAIp among the nine tumor samples analyzed. We identified 27 peptides that were detected in at least two patient samples. Seven noncHLAIp, already validated in 0D5P, were confirmed by PRM in at least one other patient sample that expressed HLA allotypes with identical or highly similar binding specificities (Supplementary Table 1), with a total of 15 individually detected PRM events (Fig. 8a). One noncHLAIp, VTDQASHIY, derived from microcephalin-1 antisense RNA (*MCPH1-AS1*), was independently confirmed with PRM in three melanoma or lung cancer patients (Supplementary Fig. 7a, b). Further, the shared presentation of the noncHLAIp AAFDRAVHF, derived from the family of LINEs (LINE/L2) on chromosome 6, was confirmed in two melanoma samples (Supplementary Fig. 7c, d). Interestingly, the corresponding source RNA expression is restricted to the skin and testis.

Next, we assessed a large collection of immunopeptidomic datasets (ipMSDB^48^, 91 biological cancer tissue/cell line sources, 35 biological healthy tissues/cell line sources; 1,102 MS raw files in total) and obtained the first large-scale signature of noncHLAIp presentation (Supplementary Data 8). In total, 220,293 peptides were obtained from healthy samples versus 280,385 peptides from cancer samples. We re-identified in ipMSDB 92 tumor-specific noncHLAIp (source genes described above and were expressed at 90th percentile TPM ≤ 1 in a maximum of three tissues) (Fig. 8b), 60 of which were only detected in cancer immunopeptidome samples. From those, 14 were detected in at least one additional cancer sample in ipMSDB. Overall, noncHLAIp presentation showed a trend of enrichment in cancer samples in ipMSDB (Fig. 8c). Interestingly, two noncHLAIp from the lncRNA *HAGLROS* (KVLAGTVLFK and VLAGTVLFK), identified specifically in the lung cancer tissue in our samples, were exclusively found in cancer samples in ipMSDB, mainly in ovarian cancer samples, consistent with a previous report^49^.

### Immunogenicity of noncHLAIp with autologous T cells

**Fig. 9:**
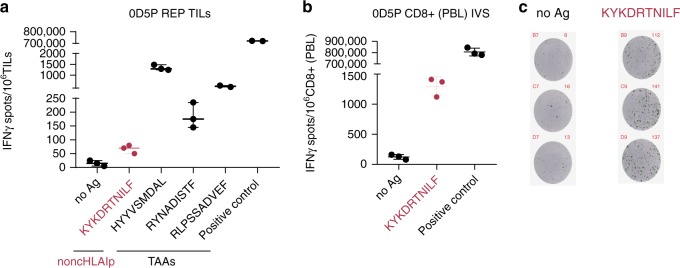
Non-canonical ABCB5 peptide induced an IFNγ response. **a** Reactivity was measured in melanoma 0D5P by the IFNγ ELISpot assay using autologous REP TILs. Representative example of three TAAs from TYR and TYRP1 and one non-canonical dORF-derived HLAIp from ABCB5 (written in red) that induced an IFNγ response. **b** In addition, a representative example of CD8+ T lymphocytes from PBLs is shown when re-challenged with autologous CD4+ blasts together with 1 μM of the non-canonical ABCB5 HLAIp. (No Ag: no peptide, positive control: 1x cell stimulation cocktail).**c** Representative images of the IFNγ ELISpot response against the non-canonical ABCB5 peptide. In **a** and **b**, T cell reactivity for every peptide was validated by ≥ 2 independent experiments. Please refer to the Methods section for boxplot parameters. Source data are provided as a Source Data file.

The involvement of noncHLAIp in tumor immune recognition was assessed by measuring IFNγ release upon peptide stimulation of autologous tumor-infiltrating lymphocytes (TILs) or peripheral blood mononuclear cells (PBMCs) from the same patient. Out of the 786 peptides screened (94 TEs, 421 lncRNAs, 56 alternative ORFs, and 215 TAAs), we confirmed the specific recognition by autologous TILs of TAAs, such as the HYYVSMDAL and RLPSSADVEF peptides from tyrosinase (TYR) and RYNADISTF from tyrosinase-related protein 1 (TYRP1) in melanoma sample 0D5P, and of the YLEPGPVTA peptide from the promelanosome protein (PMEL) in melanoma sample T1015A. One non-canonical peptide, KYKDRTNILF, derived from the downstream ORF (dORF) of the melanoma stem-cell marker *ABCB5* gene in 0D5P, was also found to be immunogenic in both autologous CD8+ TILs and CD8+ T cells from peripheral blood lymphocytes (PBLs) (Fig. 9a–c). Notably, this peptide was found shared across three additional melanoma samples in ipMSDB.

## Discussion

Our proteogenomics approach led to the stringent identification of hundreds of noncHLAIp derived from presumed non-coding genes, TEs and alternative ORFs. This feat was achieved with NewAnce, a computational module that overcomes the challenge of reduced sensitivity and specificity when searching against large MS search spaces, and it can be applied to any (non-canonical) protein sequence database of interest^28,50^. We rigorously tested the validity of noncHLAIp identifications with HLA binding predictions, sequence-specific hydrophobicity characteristics, targeted MS analyses, and provided evidence of translation in peptide-encoding ORFs by Ribo-Seq. Using all of these strategies together, we confirmed that NewAnce was superior to MaxQuant and Comet alone, across all the investigated samples. Taking one patient as an example, we conducted PRM and Ribo-Seq analyses to compare a subset of protHLAIp to non-canonical antigen classes (lncRNAs and TEs), thereby validating the identified noncHLAIp at the experimental level. We found that noncHLAIp had an overall lower confirmation rate than protHLAIp, possibly due to their lower expression, which also led to their stochastic detection by MS. Interestingly, the expression and translation of microproteins derived from presumed non-coding RNAs in the heart were recently discovered using a Ribo-Seq directed proteogenomics approach. Similar to our results, evidence of translation was confirmed for 22.5% of the lncRNAs, while 55.4% of the micropeptides were validated by PRM MS^51^. Importantly, our results additionally demonstrate that the correct identification of noncHLAIp in proteogenomic workflows requires proper FDR control and validation using multiple independent methods.

Combining immunopeptidomics with RNA-Seq and Ribo-Seq datasets enables the comprehensive assessment of how transcription, translation and HLA presentation are correlated. Despite the different methodological challenges^52–54^, we observed the expected correlations between HLA presentation level and expression, especially by Ribo-Seq, presumably because translation is biologically closer to antigen processing and presentation than transcription is. In addition, we found that in the melanoma sample 0D5P, most of the noncHLAIp derived from the Ribo-Seq inferred database originated from source genes harboring upstream ORFs (uORFs). Notably, uORFs can trigger the non-sense-mediated decay of messenger RNAs (mRNAs) and provide a rich source of noncHLAIp^55–57^.

While a previous study showed that the presentation of non-canonical peptides was enhanced by inflammatory stimuli, the presentation of only specific HLA peptides was documented^58^. In contrast, our large-scale analysis of both DAC- and IFNγ-treated cells did not detect profound changes in noncHLAIp presentation, although non-coding source genes were induced. Hence, we hypothesize that low copy numbers of such noncHLAIp remain a limiting factor for their presentation. Moreover, corroborating prior research^27^, we report the enrichment of noncHLAIp originating from the C-terminus of source protein sequences. Translation products of such presumed non-coding regions could be considered defective ribosomal products that are expected to be unstable and rapidly degraded, likely bypassing the proteasome^59^.

Given the lack of complete tissue immunopeptidomics reference libraries from healthy donors, we propose a workflow to retrospectively search for tumor-specific non-coding source genes with publicly available RNA-Seq databases (such as GTEx^46^). We observed that 23% of the source non-canonical genes were not expressed in healthy tissues (with our selected thresholds), and could be considered tumor-specific. However, in the ideal situation in which both tumor and matched normal tissues were available, we found that the majority of peptides were detected in both, suggesting that the comparison with GTEx overestimates the fraction of true tumor-specific non-canonical ligands, and that some might be patient-specific. Interestingly, two overlapping epitopes were identified in the lncRNA *HAGLROS*, which were expressed and presented uniquely in the lung tumor tissue. This lncRNA has been implicated in cancer progression^60,61^ and should be prioritized for downstream validation. Moreover, while Laumont et al.^23^ first proposed the existence of shared noncHLAIp, our work validates that noncHLAIp can be shared across multiple tumor samples, and we anticipate better treatment efficacy with such shared noncHLAIp compared to that achieved with private neoantigens^62,63^.

The expression of tumor-specific noncHLAp in a subpopulation of tumor cells suggests a dependency on a molecular or functional state. For example, the immunogenic noncHLAIp derived from the dORF in the *ABCB5* gene was moderately expressed in only 37% of the melanoma cells compared to the expression of the *TYR* and *TYRP1* genes, both of which were highly and uniformly expressed and produced confirmed immunogenic epitopes. Immune pressure on selected tumor cell subsets with particular biological relevance, such as cancer stem-like cells, tumor cells with epithelial-mesenchymal transition features and proliferating tumor cells, could greatly impact tumor behavior and be clinically beneficial.

Indeed, we found such an immunogenic noncHLAIp from 0D5P derived from the dORF of the *ABCB5* gene. ABCB5 has been shown to be expressed in malignant melanoma-initiating cells and is thought to be responsible for both the progression and chemotherapeutic refractoriness of advanced malignant melanoma^43^. Through an IL1β/IL8/CXCR1 cytokine signaling circuit, ABCB5 has been shown to control IL1β secretion and maintain slow cycling and chemoresistance^64^. Blocking ABCB5 reversed resistance to multiple chemotherapeutic agents, induced cellular differentiation and impaired tumor growth in vivo^64^. We found that *ABCB5* was differentially co-expressed in a cluster of 0D5P cells with the transcription factor *MITF* and *CTNNB1*, whose expression may be enriched in melanoma stem-cell populations^65^. The presence of spontaneous specific T cells recognizing the noncHLAIp derived from the dORF of the *ABCB5* gene in both peripheral blood and TILs suggests no central tolerance and that this target could allow immune targeting of the melanoma stem-cell subpopulation to curtail tumor growth.

Out of >500 noncHLAIp screened, immune recognition by rapidly expanded TILs and PBMCs was detected for only a single immunogenic noncHLAIp. Various mechanisms could account for the lack of recognition by autologous T cells. First, we were able to screen only autologous TILs that had long propagated in culture. We previously reported that TIL ex vivo expansion may lead to depletion of T cell clones that recognize tumor neoantigens^66^. Second, it is possible that the melanoma cells, which had to be expanded considerably in culture for immunopeptidomics analyses, could have undergone an alteration of their HLA peptide repertoire, leading to the identification of noncHLAIp that were not originally present in the freshly extracted cells. However, we also assessed snap-frozen lung cancer tissues and still did not observe the immune recognition of identified non-canonical targets in autologous PBMCs. Alternatively, the ability of noncHLAp to induce a natural immune response might be inferior to that of protHLAp. Low expression might limit the uptake by professional antigen-presenting cells and thus also limit the priming of naive T cells in vivo through cross presentation. Similarly, the engagement of CD4+ T helper cells through HLA class II presentation might also be limited. Nevertheless, tumor-specific non-canonical targets may still be valuable for immunotherapy, even when no prior immune response against the targets has been detected ex vivo, as was previously shown for neoantigens^3,4,67^. More research should be performed to thoroughly assess the ability of noncHLAp to augment protective immune responses in vivo. Such approaches are supported by evidence in mouse models demonstrating that peptides derived from non-canonical regions can be spontaneously recognized and leveraged in cancer immunotherapy^23,68^.

Remarkably, across tumor types, the potential number of predicted noncHLAp is orders of magnitude larger than that of neoantigens encompassing non-synonymous somatic mutations. As T cell-based screenings currently have limited throughput and are expensive^69^, an accurate and cost-effective non-canonical target discovery approach is crucial for their further development and use in cancer immunotherapy. With the renewed interest in cancer vaccines and the constantly growing number of antigens screened for immune recognition, we expect that enough training data will become available to allow the development of accurate predictors of immunogenicity. Combining this approach with our developed module NewAnce to shortlist noncHLAp presented in vivo and to rank them according to their predicted immunogenicity will facilitate the comprehensive exploration of non-canonical antigens, their association with immune responses and their potential for building effective cancer immunotherapies.

## Methods

### Patient material

Melanoma cell lines (0D5P, 0MM745, 0NVC) were generated as follows: patient-derived tumors were cut into small pieces before being transferred into a digestion buffer containing collagenase type I (Sigma Aldrich) and DNase I (Roche) for at least 1 h. Dissociated cells were washed and maintained in RPMI 1640 +  lutaMAX medium (Life Technologies) supplemented with 10% heat-inactivated FBS (Dominique Dutscher) and 1% Penicillin/Streptomycin Solution (BioConcept). If fibroblasts appeared, they were selectively eliminated with G418 (Geneticin; Gibco) treatment. The primary melanoma cell lines T1185B, T1015A, Me290, and Me275 were generated at the Ludwig Institute for Cancer Research, Department of Oncology, University of Lausanne^70,71^. All established melanoma cells were subsequently grown to 1 × 10^8^ cells, collected by centrifugation at 151 x *g* for 5 min, washed twice with ice-cold PBS and stored as dry cell pellets at −20 °C until use. For the in vitro 72 h treatment with IFNγ (100 IU/mL, Miltenyi Biotec), T1185B cells were grown to 2 × 10^8^ in triplicate. For the treatment with DAC (Sigma Aldrich), 2 × 10^8^ melanoma cells were grown for 8 days in medium containing 0.5 µM DAC, and the drug was readministered on the fourth day.

Autologous TILs were expanded from fresh melanoma tumor samples from patients 0D5P, 0MM745, 0NVC, LAU1185 (tumor cell line T1185B), LAU1015 (tumor cell line T1015A), LAU203 (tumor cell line Me290) and LAU50 (tumor cell line Me275) at the Ludwig Institute for Cancer Research, Department of Oncology, University of Lausanne. The fresh tissues were manually cut into fragments of one to two mm^3^. The tumor fragments were then placed in 24-well plates containing RPMI CTS grade (Life Technologies), 10% human serum (Biowest), 0.025 M HEPES (Life Technologies), 55 μmol/L 2-mercaptoethanol (Life Technologies) and supplemented with IL-2 (6000 IU/mL, Proleukin) for 3 to 5 weeks. Following this pre-rapid expansion protocol (REP), TILs were then expanded with another REP as follows: 5 × 10^6^ TILs were stimulated with irradiated feeder cells (Ratio 1:200), anti-CD3 (OKT3, 30 ng/mL, Miltenyi Biotec) and IL-2 (3000 IU/mL) for 14 days. After 14 days of REP, ~2 × 10^9^ TILs were harvested, washed, and cryopreserved until use. The purity (i.e., the percentage of CD3 T cells) was >95%. As an additional control, one flask with the exact same REP conditions without TILs was cultured in parallel, and no cells were detectable at day 14. REP TILs were thawed in 5 IU/mL DNase I (Sigma Aldrich) and cultured in 3000 IU/mL IL-2 for two days in RPMI 1640 medium with GlutaMAX™ Supplement (Gibco), and 8% human serum (Biowest), 10 mM HEPES (Gibco), 50 μM Beta-mercaptoethanol (Gibco), 100 μM non-essential amino acids (Gibco), 100 IU/mL penicillin, 0.1 mg/mL streptomycin, 2 mM l-glutamine (Gibco), 0.1 mg/mL kanamycin sulfate (Carl Roth), and 1 mM sodium pyruvate (Gibco). The cells were then washed twice in complete medium and subsequently rested overnight in the presence of 150 IU/mL IL-2 prior to peptide stimulation.

Snap-frozen normal and lung tumor tissue materials from the C3N-02289 (Lung squamous cell carcinoma, grade 2) and C3N-02671 (lung adenocarcinoma, G2) samples were kindly provided by the International Institute of Molecular Oncology. Informed consent was obtained from the participants in accordance with the requirements of the institutional review board (Ethics Commission, CHUV, Bioethics Committee, Poznan University of Medical Sciences, Poznań, Poland).

All cells tested negative for mycoplasma contamination. High-resolution 4-digit HLA-I and HLA-II typing (Supplementary Data 1) was performed at either the Laboratory of Diagnostics, Service of Immunology and Allergy, CHUV, Lausanne or in-house using the HLA amplification method with the TruSight HLA v2 Sequencing Panel kit (CareDx) according to the manufacturer’s protocol. Sequencing was performed on the Illumina® MiniSeq™ System (Illumina) using a paired end 2 × 150 bp protocol. The data were analyzed with Assign TruSight HLA v2.1 software (CareDx).

### Immunoaffinity purification of HLA peptides

We performed HLA immunoaffinity purification according to our previously established protocols^39,72^. W6/32 and HB145 monoclonal antibodies were purified from the supernatants of HB95 (ATCC® HB-95™) and HB145 cells (ATCC® HB-145™) using protein-A sepharose 4B (Pro-A) beads (Invitrogen), and antibodies were then cross-linked to Pro-A beads. Cells were lysed with PBS containing 0.25% sodium deoxycholate (Sigma Aldrich), 0.2 mM iodoacetamide (Sigma Aldrich), 1 mM EDTA, a 1:200 protease inhibitors cocktail (Sigma Aldrich), 1 mM phenylmethylsulfonylfluoride (Roche), and 1% octyl-beta-d glucopyranoside (Sigma Alrich) at 4 °C for 1 h. The lysates were cleared by centrifugation in a table-top centrifuge (Eppendorf) at 4 °C for 50 min at 21,191 x *g*. Snap-frozen tissue samples were homogenized on ice in 3–5 short intervals of 5 s each using an Ultra Turrax homogenizer (IKA) at maximum speed. The lysates were then cleared by centrifugation at 75,600 x *g* in a high-speed centrifuge (Beckman Coulter, Avanti JXN-26 Series, JA-25.50 rotor) at 4 °C for 50 min. For HLA immunopurification, we employed the Waters Positive Pressure-96 Processor (Waters) and 96-well single-use micro-plates with 3 µm glass fibers and 10 µm polypropylene membranes (Seahorse Bioscience, ref no: 360063). Anti pan HLA-I and HLA-II antibodies cross-linked to beads were loaded onto separate plates, respectively. For tissue samples, depletion of endogenous antibodies was required with Pro-A beads. The lysates were passed sequentially through the first plate containing pan HLA-I antibody-cross-linked beads, then through the second plate with pan HLA-II antibody-cross-linked beads, at 4 °C. The beads in the plates were then washed separately with varying concentrations of salts using the processor. Finally, the beads were washed twice with 2 mL of 20 mM Tris-HCl pH 8.

Sep-Pak tC18 100 mg Sorbent 96-well plates (Waters, ref no: 186002321) were used for the purification and concentration of HLA-I and HLA-II peptides. The C18 sorbents were conditioned, and the HLA complexes and bound peptides were directly eluted from the affinity plate with 1% trifluoroacetic acid (TFA; Sigma Aldrich). After washing the C18 sorbents with 2 mL of 0.1% TFA, HLA-I peptides were eluted with 28% acetonitrile (ACN; Sigma Aldrich) in 0.1% TFA, and HLA-II peptides were eluted with 32% ACN in 0.1% TFA. Recovered HLA-I and -II peptides were dried using vacuum centrifugation (Concentrator plus, Eppendorf) and stored at −20 °C.

### Liquid chromatography–mass spectrometry (LC-MS/MS) analyses

The LC-MS/MS system consisted of an Easy-nLC 1200 (Thermo Fisher Scientific) connected to a Q Exactive HF-X mass spectrometer (Thermo Fisher Scientific). Peptides were separated on a 450 mm analytical column (8 µm tip, 75 µm inner diameter, PicoTip^TM^Emitter, New Objective) packed with ReproSil-Pur C18 (1.9 µm particles, 120 Å pore size, Dr. Maisch GmbH). The separation was performed at a flow rate of 250 nL/min by a gradient of 0.1% formic acid (FA) in 80% ACN (solvent B) in 0.1% FA in water (solvent A). HLAIp were analyzed by the following gradient: 0–5 min (5% B); 5–85 min (5–35% B); 85–100 min (35–60% B); 100–105 min (60–95% B); 105–110 min (95% B); 110–115 min (95–2% B) and 115–125 min (2% B). HLAIIp were analyzed by the following gradient: 0–5 min (2–5% B); 5–65 min (5–30% B); 65–70 min (30–60% B); 70–75 min (60–95% B); 75–80 min (95% B), 80–85 min (95–2% B) and 85–90 min (2% B).

The mass spectrometer was operated in the data-dependent acquisition (DDA) mode. Full MS spectra were acquired in the Orbitrap from *m*/*z* = 300–1650 with a resolution of 60,000 (*m*/*z* = 200) and an ion accumulation time of 80 ms. The auto gain control (AGC) was set to 3e6 ions. MS/MS spectra were acquired in a data-dependent manner on the ten most abundant precursor ions (if present) with a resolution of 15,000 (*m*/*z* = 200), an ion accumulation time of 120 ms, and an isolation window of 1.2 m/z. The AGC was set to 2e5 ions, the dynamic exclusion was set to 20 s, and a normalized collision energy (NCE) of 27 was used for fragmentation. No fragmentation was performed for HLAIp with assigned precursor ion charge states of four and above or for HLAIIp with an assigned precursor ion charge state of one, or six and above. The peptide match option was disabled.

### Parallel reaction monitoring

Selected endogenous HLAp that required confirmation by PRM were ordered from Thermo Fisher Scientific as crude (PePotec grade 3) or HPLC grade (purity >70%) with one stable isotope-labeled amino acid. The mass spectrometer was operated at a resolution of 120,000 (at *m*/*z* = 200) for the MS1 full scan, scanning a mass range from 300 to 1650 m/z with an ion injection time of 100 ms and an AGC of 3e6. Then each peptide was isolated with an isolation window of 2.0 m/z prior to ion activation by high-energy collision dissociation (HCD, NCE = 27). Targeted MS/MS spectra were acquired at a resolution of 30,000 (at *m*/*z* = 200) with an ion injection time of 60 ms and an AGC of 5e5. Only those peptides that ultimately passed quality control were considered for further downstream analyses by spiking them back into the patient sample.

The PRM data were processed and analyzed by Skyline (v4.1.0.18169)^73^, and an ion mass tolerance of 0.02 m/z was used to extract fragment ion chromatograms. To display MS/MS spectra, raw data were converted into the MGF format by MSConvert (Proteowizard v3.0.18136), and peak lists for the heavy-labeled peptides and light counterparts were extracted. The assessment of MS/MS matching was performed by pLabel (v2.4.0.8, pFind studio, Sci. Ac.) and Skyline.

### Exome/RNA sequencing

DNA was extracted for HLA typing and exome sequencing with the commercially available DNeasy Blood & Tissue Kit (Qiagen) according to the manufacturers’ protocols. For tissue samples, pelleted DNA was used, which was obtained after lysis of the tissue and centrifugation during HLA immunopurification. The supernatant was used for HLA immunopurification, whereas the pelleted DNA was resuspended in PBS using a pestle (70 mm, 1.5/2.0 mL, Schuett-Biotec) before DNA extraction according to the manufacturer’s instructions.

RNA was extracted for RNA sequencing using the Total RNA Isolation RNeasy Mini Kit (Qiagen) according to the manufacturer’s protocol for all melanoma cell lines (including DNase I (Qiagen) on-column digestion). Frozen pieces of tumor and normal tissue samples (< 20 mg) were directly submerged in 350 µL of RLT buffer supplemented with 40 µM dithiothreitol (DTT, Sigma Aldrich). Tissues were then completely homogenized on ice using a pestle (70 mm, 1.5/2.0 mL, Schuett-Biotec) and passed through a 26G needle syringe five times (BD Microlance). Centrifugation was performed in a table-top centrifuge (Eppendorf) at 4 °C for 3 min at 18,213 x *g* before the supernatant was removed and used for RNA extraction. All subsequent steps are described in detail in the manufacturer’s protocol (including DNase I (Qiagen) on-column digestion).

Three micrograms of genomic DNA were fragmented to 200 bp using Covaris S2 (Covaris). Sequencing libraries were prepared with the Agilent SureSelectXT Reagent Kit (Agilent Technologies). Exome enrichment was performed with Agilent SureSelect XT Human All Exome v5 probes. Cluster generation was performed from the resulting libraries using the Illumina HiSeq PE Cluster Kit v4 reagents and sequenced on the Illumina HiSeq 2500 platform using SBS Kit v4 reagents. At least 70x coverage was required for the melanoma cell lines and PBMCs/TILs. For tumor/normal lung tissues, at least 100x coverage was required. Sequencing data were de-multiplexed using bcl2fastq Conversion Software (v. 1.84, Illumina).

RNA quality was assessed on a Fragment Analyser (Agilent Technologies), and all RNAs had an RNA quality number (RQN) ranging from 7.4 to 10. RNA-Seq libraries were prepared using 500 or 375 ng of total RNA with the Illumina TruSeq Stranded mRNA reagents (Illumina) according to the manufacturer’s recommendations. Libraries were quantified by a fluorometric method and their quality was assessed on a Fragment Analyser. Cluster generation was performed from the resulting libraries using the Illumina HiSeq PE Cluster Kit v4 reagents and sequenced on the Illumina HiSeq 2500 platform using HiSeq SBS Kit v4 paired end reagents for 2 × 100 cycles paired end sequencing. Sequencing data were de-multiplexed using bcl2fastq2w Conversion Software (v. 2.20, Illumina).

### RNA-Seq processing for lncRNA and gene expression analysis

The GENCODE comprehensive gene annotation version 221.2 was downloaded from the GENCODE website (https://www.gencodegenes.org/human/release_22.html) and used to define the protein-coding and non-coding gene features, including chromosome position, transcript structure, and transcript and protein sequences. Here, the human reference genome GRCh38/hg38 was downloaded from the NCBI (https://www.ncbi.nlm.nih.gov/assembly/5800238) and used as the genome assembly. The RNA-Seq reads were aligned to the GRCh38/hg38 reference genome using RNA-Star (v2.4.2a; https://github.com/alexdobin/STAR). Gene expression was normalized and calculated as FPKM values by Cufflinks (v2.2.1) (https://github.com/cole-trapnell-lab/cufflinks). The gene-level RNA expression data for both protein-coding and non-coding genes were used for downstream gene expression analysis^32,74^.

### RNA-Seq data processing for TE expression analysis

We developed an analytical pipeline that was capable of assigning TE-derived RNA-Seq reads to single loci in >95% of the cases. Reads from the investigated samples and public data from GTEx were mapped to the human (GRCh37) genome using hisat2 v.2.1.0^75^. Counts on genes and TEs were generated using featureCounts 1.6.2^76^. To avoid read assignment ambiguity between genes and TEs, a gtf file containing both was provided to featureCounts. For repetitive sequences, an in-house curated version of the Repbase database was used (fragmented LTR and internal segments belonging to a single integrant were merged). Only uniquely mapped reads were used for counting genes and TEs. Finally, features that did not have at least one sample with 20 reads were discarded from the analysis. Normalization for sequencing depth was performed for both genes and TEs using the Trimmed Mean of *M* values (TMM) method as implemented in the limma v.3.36.5 package of Bioconductor^77^ and using the counts on genes as the library size.

### Personalized sequence databases from non-coding transcripts

The curated set of human ENCODE non-coding transcripts (GRCh37 reference assembly) was downloaded from https://www.gencodegenes.org/human/release_24lift37.html. ORFs in all three forward reading frames were identified using a stop-to-stop strategy. The minimum peptide length was set to eight amino acids, and the longest polypeptide identified was 3644 amino acids. Unless otherwise mentioned, to build the personalized protein fasta file, we selected transcripts from non-coding genes that were expressed in each sample (i.e., FPKM > 0) and translated them in all three forward reading frames.

### Personalized databases with variants

GENCODE v24 (GRCh37 human reference assembly) downloaded from https://www.gencodegenes.org/human/release_24lift37.html was chosen as the standard reference dataset. Whole-exome sequence reads were aligned to the GRCh37 human assembly with BWA-MEM^78^, and variants were predicted using GATK framework v3.7 and Picard Tools v2.9.0^79^. Small nucleotide polymorphisms (SNPs) were defined as variants present in both tumor and germline samples, and somatic mutations (somatic nucleotide variants (SNVs) and indels) were defined as being present in only tumors. The GENCODE comprehensive gene annotation file, in GFF3 format, was parsed to extract genomic coordinate information for every exon in each protein-coding transcript, and those coordinates were compared with sample-specific variant coordinates to derive non-synonymous amino acid changes within each protein. For every sample, we created a separate fasta file for which residue mutation information was added to the header of the affected translated protein-coding transcripts, in a format compatible with MaxQuant v1.5.9.4i^80^.

### Mass spectrometry database search

We used two widely used search tools: Comet 2017.01 rev. 2^34^ and the Andromeda search engine within MaxQuant v1.5.9.4i^81^. Both Andromeda and Comet allow searching for peptides with and without variants. Andromeda matched the MS/MS spectra of each sample against the personalized reference libraries (mentioned above). Similarly, the variants were annotated in the PEFF format (http://www.psidev.info/peff) for Comet. Both search tools were run with the same principal search parameters: precursor mass tolerance 20 ppm, MS/MS fragment tolerance of 0.02 Da, peptide length of 8–15 when searching only HLA-I peptides and 8–25 for both HLA-I and HLA-II peptides and no fixed modifications. For samples 0D5P, 0NVC and 0MM745, oxidation (M) and phosphorylation (STY) were set as variable modifications; for the remaining samples only oxidation (M) was included as a variable modification. A PSM FDR of 3% was used for Andromeda as a first filter, and non-canonical reference sequences were loaded into the “proteogenomics fasta files” module for FDR calculations for proteome-derived and non-canonical sequences. For each spectrum the annotated PSMs with the highest score were kept (including the decoy hits calculated by Andromeda from reversed protein sequences) and stored in binary files.

To assure that non-canonical peptide sequences did not match other protein-coding genes, all peptides found by Andromeda or Comet were aligned against an up-to-date UniProt/TrEMBL sequence database (95,106 protein sequences of the human reference proteome up000005640, with isoforms, downloaded 26/09/2018) using an algorithm built in NewAnce. Leucine and iso-leucines were treated as equal since they are not distinguishable by MS. If peptides were found to match standard UniProt sequences, they were assigned as proteome-derived with the UniProt IDs. However, we retained non-canonical TE peptide sequences that matched annotated TEs that were integrated into the human reference in UniProt.

Comet PSMs were read from Comet pep.xml files and all peptides were aligned against the UniProt database as described above. Equivalent to the Andromeda PSMs processing, PSM were annotated and the highest scoring PSMs were stored in binary files. Comet PSM processing was implemented in Java and utilizes the MzJava class library^82^. As described in detail below, it consisted of two main steps: first, three Comet scores *XCorr, deltaCn*, and *spScore* and the spectrum charge were combined, and second, the FDR was calculated separately for proteome-derived and non-canonical peptides. The first step boosted the overall number of identified PSMs at a given global FDR, whereas the second step limited the number of false positives in the group of non-canonical peptides at a given global FDR.

All PSMs resulting from the Comet binary files were split into three sublists with PSMs of charge (*Z*) 1 (applicable to HLAIp only), 2, and charge 3 or higher. Further, the three Comet scores XCorr, deltaCn and spScore were considered (the expect score was left out because it depends on the size of the sequence database). In order to calculate the FDR for three-dimensional (3D) vectors ${\mathbf{x}} = \left( {XCorr,\;deltaCn,spScore} \right)$, the 3D spaces (one 3D space per charge state *Z*) were partitioned into small cells with 40 intervals in each dimension (Supplementary Fig. 1a). The PSMs in the sublist of charge *Z* were then parsed and for each cell, the number of wrong hits (*n*_0_) was set to the number of decoy PSMs in that cell, and the number of true hits (*n*_1_) was set to the number of target (non-decoy) PSMs minus *n*_0_. The 3D probability distributions were estimated by dividing the counts in each cell by the total counts summed over all cells resulting in a distribution for each charge state *Z* for true $\left( {p\left( {{\mathbf{x}}{\mathrm{|}}Z,H = 1} \right)} \right)$ and wrong $\left( {p\left( {{\mathbf{x}}{\mathrm{|}}Z,H = 0} \right)} \right)$ PSMs. In order to obtain smoother distributions, both true (*n*_1_) and decoy (*n*_0_) counts were averaged over a 9-cell nearest neighborhood. This 3D histogram based approach has the advantage that it does not require strong assumptions about the shape of the probability distributions, and in contrast to one-dimensional (1D) projection methods, it does take into account the full 3D structure of the score space. On the other hand, it requires fairly large datasets with >100,000 PSMs.

The local FDR (*lFDR*) is the probability that a PSM within a given cell is wrong, whereas the global FDR is the probability that a PSM in the final result list from all cells is wrong. It has been shown that *lFDR* calculation provides the most sensitive decision boundaries while controlling the global FDR^83^. Mathematically, $lFDR\left( {{\mathbf{x}},Z} \right)$ values for a score vector **x** and charge *Z* can be calculated by Equation (1):

1 $$lFDR\left( {{\mathbf{x}},Z} \right) =	 \; \frac{{\pi _0p\left( {{\mathbf{x}}{\mathrm{|}}Z,H = 0} \right)}}{{\pi _0p\left( {{\mathbf{x}}{\mathrm{|}}Z,H = 0} \right) + \pi _1p\left( {{\mathbf{x}}{\mathrm{|}}Z,H = 1} \right)}} = \left( {1 + \frac{{\pi _1}}{{\pi _0}} \cdot \frac{{p\left( {{\mathbf{x}}{\mathrm{|}}Z,H = 1} \right)}}{{p\left( {{\mathbf{x}}{\mathrm{|}}Z,H = 0} \right)}}} \right)^{ - 1}\\ =	 \; \left( {1 + \frac{{\pi _1}}{{\pi _0}}\gamma \left( {{\mathbf{x}},Z} \right)} \right)^{ - 1}$$

where *π*_0_ and *π*_1_ are the class probabilities for true (*H* = 1) and wrong (*H* = 0) PSMs, and $p\left( {{\mathbf{x}}{\mathrm{|}}Z,H = 0,1} \right)$ are the probability distributions as described above. Finally, the *lFDR* threshold was adjusted to yield a global FDR of 3% and all PSMs within cells with *lFDR* values smaller than this threshold were added to the list of PSMs. Supplementary Fig. 1b shows a comparison of this 3D histogram approach to a simpler 1D method, where only the *XCorr* score was used, for the 0D5P sample. At the same FDR of 3%, the 3D histogram approach was able to boost the number of unique peptides for both proteome-derived and non-canonical peptides by 22% and 13%, respectively. Importantly, the percentage of predicted HLA binders and the standard error in hydrophobicity index calculation by SSRCalc remained unchanged (Supplementary Fig. 1c, d), indicating that the 3D method used in NewAnce did not inflate the error. However, Supplementary Fig. 1c also reveals that the percentage of predicted binders is low for the group of non-canonical peptides (only 55% compared to 95% for proteome-derived peptides), indicating a large portion of wrong PSMs in the non-canonical group. This phenomenon has been reported before^28^ and is due to a misbalance of true and false hits in the non-canonical sequence databases. Non-canonical sequence databases are typically very large, and they contain mostly sequences that have low probability to contribute to true hits.This causes a strong prevalence for wrong PSMs or a low $\pi _1/\pi _0$ ratio ($\pi _1/\pi _0$ ratio is the total number of true PSMs divided by the total number of wrong PSMs) compared to the proteome-derived database.

In order to tackle this problem, we implemented an approach that estimates the *lFDR* values separately for non-canonical and proteome-derived PSM groups. Since there are only several hundreds of non-canonical PSMs, we could not use the 3D histogram approach directly for the non-canonical PSM group. Instead, we assumed that the probability distributions $p\left( {{\mathbf{x}}{\mathrm{|}}Z,H = 0,1} \right)$ are the same for non-canonical and proteome-derived PSMs, and that the $\pi _1/\pi _0$ ratios strongly depend on the PSM group. The $\pi _1/\pi _0$ ratios are global measures and can be readily calculated with a few hundred PSMs. Therefore, we first calculated the probability ratios $\gamma \left( {{\mathbf{x}},Z} \right)$ for each cell using all PSMs and then calculated the $\pi _1/\pi _0$ ratios separately for the non-canonical and the proteome-derived groups. We then plugged the group-specific $\pi _1/\pi _0$ ratios into Eq. (1) and obtained a group-specific calculation of the *lFDR* for each cell. The *low* $\pi _1/\pi _0$ *ratio* in the non-canonical group will increase the *lFDR* values for this group. When the *lFDR* threshold was adjusted to yield a global FDR of 3%, less but higher quality non-canonical PSMs passed this filter. Supplementary Fig. 1b shows that the number of passing non-canonical peptides (3D, two groups) dropped to 28% compared to the number of peptides identified without group adjustment (3D, one group), whereas the number of proteome-derived peptides increased slightly by 8%. However, the percentage of predicted binders among the passing non-canonical peptides (3D, two groups) increased to 85% (Supplementary Fig. 1c) and the standard error of HI decreased significantly (Supplementary Fig. 1d).

Even if this group-specific *lFDR* calculation improved the accuracy of non-canonical PSMs, the fairly low percentage of predicted binders indicated that there was still a larger error in this group. In order to discard more of this residual error, we combined the Comet and Andromeda search results and only the intersection, i.e., PSMs with identical Comet and Andromeda matches (same peptide sequence with the same identification) were retained. As shown in Supplementary Fig. 1e–g, this additional filter further reduced the number of non-canonical PSMs, but significantly increased the percentage of predicted binders to 97.3% and decreased the standard error of hydrophobicity index. Without the post-processing of Comet results performed in NewAnce, this improvement would not be possible. When only considering the *XCorr* score and without group-specific *lFDR* calculation, combining Comet and MaxQuant would yield more peptides, but with significantly lower percentage of predicted binders (87.3%) and almost double the standard error of HI (1D, one group in green color compared with NewAnce 3% FDR in gray color in Supplementary Fig. 1e–g). Using a FDR threshold of 1% instead of 3% for MaxQuant and Comet in NewAnce would only reduce the number of peptides but not increase the percentage of predicted binders, or decrease the standard error of HI, thus justifying our choice of utilizing a 3% FDR threshold (NewAnce 3% FDR in gray color compared with NewAnce 1% FDR in beige color in Supplementary Fig. 1e–g).

In order to assign peptides into source protein groups, we implemented a greedy bipartite graph protein grouping algorithm^84^. The total and unique peptide counts were calculated for each protein. To calculate the adjusted peptide counts we sorted the proteins in each group by decreasing number of peptides and for each protein removed the peptides of all proteins higher up in the list.

In order to test the robustness of our approach, the 2597 PSMs of identified noncHLAp were re-searched against the human reference proteome UniProt database concatenated with the list of non-canonical peptide sequences, including six common modifications. The variable modifications included were 15.9949 Da for oxidation on M, 42.010565 Da for acetylation on the N-terminus, 79.966331 Da for phosphorylation on STY, 119.004099 Da for cysteinylation, 0.98402 Da for deamidation NQ and 57.021464 Da for carbamidomethyl on C. Comet was employed (same parameters as above, but no FDR) to investigate whether PSMs would better fit another possible proteome-derived (modified) sequence based on *XCorr*. The results are reported in Supplementary Data 4 and 5.

To build the ipMSDB database, we searched 1102 immunopeptidomic raw files with Comet (PSM FDR of 1%, as described above), and the Apache Spark cluster computing framework^85^ was used to process the results and calculate the FDR. The samples were annotated with basic biological information for further statistical analysis.

### Ribo-Seq: experimental protocol

Ribo-Seq was performed according to Calviello et al.^86^. Ribo-Seq libraries were derived from adherent melanoma 0D5P cells that were 80% confluent in 10 cm tissue culture dishes. After washing with ice-cold PBS supplemented with 100 μg/mL cycloheximide (Sigma Aldrich), the cells were immediately snap-frozen by placement in liquid nitrogen followed by placement on wet ice. A lysis buffer containing 20 mM Tris-HCl pH 7.4, 150 mM NaCl, 5 mM MgCl2, 1 mM DTT (Sigma Aldrich), 100 μg/mL cycloheximide, 1% (v/v) Triton X-100 (Calbiochem) and 25 U/mL TURBO DNase (Life Tech) in a volume of 400 µL was immediately added to the frozen cells. The cells and buffer were then scraped off, mixed by pipetting, transferred to Eppendorf tubes and lysed on ice for 10 min. The lysate was then titurated by passage through a 26-G needle ten times with a 1 mL syringe and cleared by centrifugation at 20,000 x *g* for 10 min at 4 °C. The cleared supernatant was then transferred to a pre-cooled tube on ice, and footprinting was performed by adding 1000 U of RNase I (Life Tech. #AM2295) per 400 μL of lysate and incubating in a thermomixer set at 23 °C, while shaking at 500 rpm for 45 min. The digestion was stopped by adding 13 µL of SUPERASE-In (Thermo, 20 U/µL) per 400 µL of lysate.

Ribosomes were recovered using two MicroSpin S-400 HR columns (GE Healthcare) per sample. The columns were first equilibrated with a total of 3 mL of buffer containing 20 mM Tris-Cl pH 7.4, 150 mM NaCl, 5 mM MgCl2, and 1 mM DTT by performing six rounds of washes with 500 µL of the buffer. The resin was resuspended with the last wash and drained by centrifugation for 4 min at 600 x *g*. One-half of the sample volume was then filtered per column for 2 min at 600 x *g*, and the filtered halves were then combined. To the combined flow-through, three volumes of TRIzol LS (Life Tech) were added and RNA was extracted using the Direct-zol RNA Mini-Prep kit (Zymo Research) according to the manufacturer’s instructions (including DNase I digestion). RNA was finally eluted in 30 μL of nuclease-free water and quantified using the Qubit RNA Broad Range Assay (Life Tech).

Ribosomal RNA was depleted from up to 5 μg of footprinted RNA using the RiboZero Magnetic Gold kit (Illumina) according to the manufacturer’s protocol. Footprinted RNA was precipitated from the supernatant (90 μL) using 1.5 μL of glycoblue (Life Tech), 9 μL of 3 M sodium acetate and 300 μL of ethanol by snap-freezing in liquid nitrogen, incubating for 1 h up to overnight at –80 °C, and pelleting at 21,000 x *g* for 30 min at 4 °C. The RNA pellet was dissolved in 10 μL of RNase-free water.

Following ribosomal RNA (rRNA) depletion, isolation of short fragments and phosphorylation of these fragments by T4 PNK treatment, sequencing libraries were prepared using the NEXTflex Small RNA-Seq Kit v3 (Bioo Scientific). According to the manufacturer’s instructions, adapters were diluted 1:2 to decrease adapter dimerization. To determine the optimal number of PCR cycles for library amplification, pilot PCRs with the respective forward and reverse primers were performed for each sample for 12, 14, 16, 18, and 20 cycles. Adapter and primer sequences are published by Bioo Scientific. Products were separated on a native PAGE, and optimal cycle numbers were determined as the threshold cycle of the library product at 160 bp, the expected size for RPFs, with the smallest amount of adapter dimer product (130 bp) possible. After the final PCR, libraries were separated on and excised from an agarose gel, and then cleaned using the Zymoclean Gel DNA Recovery kit (Zymo Research). Library quantification and validation were performed using the Qubit dsDNA HS and Bioanalyzer DNA HS assays, respectively. Three 0D5P control samples and three DAC treated samples (in a pool of 21 libraries) and two 0D5P samples (in a pool of 3 libraries) were sequenced on a NextSeq 500 machine at a loading concentration of 1.6 pM using High Output Kits v2 (Illumina) with 75 cycle single-end reads.

### Ribo-Seq: analysis

Ribo-Seq reads were stripped of adaptor sequences using cutdapt, and contaminants such as transfer RNAs (tRNAs) and rRNA were removed by alignment to a contaminants index via Bowtie v 2.3.5, consisting of nucleotide sequences from known human rRNA and tRNA sequences drawn from the GENCODE annotation v24^87^. Unaligned reads from this analysis were then aligned to human genome version hg19 with the STAR v 2.6.1a_08-27^88^ splice-aware alignment tool allowing for up to 1 mismatch. The star genome index was built using GENCODE v24 (lift 37). Reads with up to 20 multi-mapping positions were included, with multi-mapping reads beings separately treated in subsequent periodicity analysis. The RIboseQC pipeline v1.0^89^ was used to deduce P-site positions from the Ribo-Seq reads, and the P-site data were then used as input into the ORFquant pipeline v0.9^90^ in combination with custom R scripts^86^ for ORF calling. The ORFquant pipeline searches for the periodic ribosomal footprint pattern characteristic of translated ORFs using a supplied database of transcripts, yielding a set of ORFs corresponding to known coding regions, as well as ORFs originating from UTRs, non-coding RNAs, intron retentions, and read-through events. The 0D5P samples had a median of 2.8 million reads mapped to coding sequences per sample, which constituted a median of 81% of the total reads (Supplementary Table 2). Since the false-positive rate of periodicity based ORF calling is thought to be tolerant of non-periodic sources of noise such as genomic contamination, we included all samples for 0D5P. ORFs were called in both individual libraries and in the pooled set of all libraries for 0D5P, and ORFs that were fully contained within ORFs detected in another library were merged. ORFs were tested for periodicity, by a multitaper test^86^, and those with a *p*-value below 0.05 were retained for analyses.

Polypeptide sequences in fasta format were generated from the coordinates of these ORFs and used for both validation of the peptides found using the RNA-Seq-based database and as a de novo-assembled database for the subsequent round of peptide detection. Peptides were considered validated by Ribo-Seq if they matched anywhere within the translated ORF sequences.

Ribo-Seq profile plots were plotted with P-site numbers per-base on a log2 (*n* + 1) scale.

### The 10x genomics pipeline and gene expression analyses

For single-cell library preparation on the 10x Genomics platform, the Chromium Single-Cell 3′ Library and SingleCell 3ʹ Reagent v3 were utilized, together with the 10x Chromium single-cell controller instrument in accordance with the official CG000183 RevA user guide. A total of 1692 0D5P cells were captured for single-cell transcriptomics. The resulting cDNA libraries were sequenced on NextSeq v 2.5 (with Illumina protocol #15048776). Cell Ranger v.3.0.1 software (10x Genomics, https://support.10xgenomics.com/single-cell-gene-expression) was used to process data generated using the 10x Chromium platform, with a restriction of including only 1400 cells to avoid cells or debris with low unique molecular identifier (UMI) counts. This approach led to the detection of 19,178 genes with a mean of 125,937 mapped reads. Genes present in at least five cells and cells with at least 200 genes but no >50% of mito genes were retained for analysis, resulting in a reduced matrix of 15,710 genes over 1365 cells.

The raw counts were log-normalized using the NormalizeData implemented in the Seurat R package (Seurat v3). Prior to further processing, we scaled the data to remove cell-cell variations due to cell cycling or a high percentage of mitochondrial genes. For cell cycling correction, we followed the scoring strategy described by Tirosh et al.^91^: each cell was assigned a Cell Cycle score and the difference between G2M and S phase scores was regressed out. Clusters were obtained using a graph-based method implemented in Seurat (FindClusters with a resolution set to 0.5), leading to the identification of five clusters. Marker genes for each cluster were identified with FindMarkers from Seurat by setting the logFC threshold parameter to 0.15. Marker genes with an adjusted Bonferroni *p*-value <0.05 were considered significantly differentially expressed. Functional analyses of each cluster were performed with STRING-db v11 using their corresponding marker genes as input.

### Assessment of T cell reactivity

Peptides were synthesized and lyophilized by the Protein and Peptide Chemistry Facility at the Ludwig Institute for Cancer Research (crude, >80% purity), Department of Oncology, University of Lausanne, or by Thermo Scientific, and resuspended in dimethyl sulfoxide at 10 mg/mL. IFNγ ELISpot assays were conducted to assess the reactivity of the REP TILs towards antigens of interest (TAAs, noncHLAIp) using pre-coated 96-well ELISpot plates (Mabtech) according to the manufacturer’s protocol. If necessary, REP TILs were stimulated with a single peptide or a peptide pool at 1 μg/mL in vitro for 14 days before re-challenging with the peptide to assess the IFNγ response. For this purpose, REP TILs were plated at 1–2 × 10^5^ cells per well and challenged for 18 h with cognate peptides at a final peptide concentration of 1 µM, in duplicate or triplicate. Medium without peptide was used as a negative control, and 1x Cell Stimulation Cocktail (eBioscience™, Thermo Fisher Scientific) was used as a positive control. Spot-forming units were quantified using the Bioreader-6000-E automated counter (BioSys). Positive hits were identified by having more spots than the negative control wells, which did not contain any peptide, plus three times the standard deviation of the negative control. Positivity was confirmed in at least ≥2 independent experiments.

The identification of circulating antigen-specific T cells in patient 0D5P was performed as such^66,92^: CD19+ cells were isolated from cryopreserved PBLs using magnetic beads (Miltenyi) and expanded for 14 days with multimeric-CD40L (Adipogen, Epalinges, Switzerland, 1 μg/mL) and IL-4 (Miltenyi, 200 IU/mL). CD8+ T lymphocytes were isolated from cryopreserved PBLs using magnetic beads (Miltenyi) and co-incubated at a 1:1 ratio with irradiated autologous CD40-activated B cells and peptides (single peptides or pools of ≤ 50 peptides, 1 µM each). After 12 days of in vitro expansion, CD8+ T cells were re-challenged with cognate peptide and T cell responses were assessed by the ELISpot assay.

### Statistical analyses

Statistical analyses were performed where appropriate. The following tools were used for statistical analyses: GraphPad Prism 8, Perseus 1.5.5.3, RStudio 3.5.1 and Python 3.6. Specifically, the boxplots in Fig. 8c, Fig. 9a, b, Supplementary Fig. 1c, d and 1f–h, Supplementary Fig. 3a, b and Supplementary Fig. S4a–l were generated using the standard settings in either RStudio or GraphPad Prism. The boxplot settings were: Hinges (25% and 75%), with the median plotted. For Fig. 8c and Supplementary Fig. 1c, d and 1f–g, the notch is additionally shown at +/–1.58 IQR/sqrt(*n*), where IQR is the interquartile range (difference between 75- and 25-percentile) and *n* the number of data points. A median at the notch edge corresponds to a 95% significant difference (*p*-value = 0.05). Sample sizes and *p*-values for Supplementary Fig. 1c, d and 1f–g can be found in the Source Data File. In Fig. 9a–b, Supplementary Fig. 1h, Supplementary Fig. 3 and 4, the whiskers are plotted down to the minimum and up to the maximum value, and each individual value is plotted as a point superimposed on the graph.

### HLA binding predictions

To evaluate the binding affinity of HLAIp, MixMHCpred.v2 prediction software was run on all HLAIp ranging in length from 8 to 14 amino acids. Peptides with a *p*-value ≤ 0.05 were considered binders.

### Sequence-specific HI calculator

Sequence-specific HI was calculated with the SSRCalc vQ.0 tool^35^, available online at http://hs2.proteome.ca/SSRCalc/SSRCalcQ.html. Only unmodified peptides were included and parameters were set to: 100 Å C18 column, 0.1% formic acid separation system and without cysteine protection. Observed RTs were obtained from Comet pep.xml files. If a peptide was detected multiple times in the same sample, the mean RT was used. Peptides and their mean RTs were plotted against the calculated HIs. For Fig. 2c–f, to compare the variances in the differences between the RTs and the regression line, we applied a one-sided *F*-test.

In order to calculate the standard errors of HI, we regressed the measured RTs against the calculated HI using the lm function in R. This function returns the residuals between the regression line and HI values. The residual absolute errors of the lm-regression were plotted in Supplementary Fig. 1d, g (the higher this value, the worse the correlation between predicted and measured values). In this manner, we observe how well the HI calculations correlate with the experimentally observed RT.

### Correlation analyses

Correlative analyses of the immunopeptidome and transcriptome of 0D5P (Fig. 3a–d) were performed by first assigning HLAp to their respective source genes. For noncHLAp, the gene with the highest transcript expression was allocated for further analyses if the peptide map back to more than one non-coding source gene, unless otherwise indicated.

### Assessment of HLAIp sampling

For HLAIp sampling analyses, peptides were assigned to source protein groups as described above. Adjusted peptide counts were taken, summed over a gene, and subsequently matched to their corresponding expression values (either transcriptome or translatome based). Normalized sampling corresponds to the adjusted peptide count per protein, normalized by the protein length. The correlation between gene expression or the spectral coefficients of 3-periodic signals in Ribo-Seq data and HLA presentation were assessed by fitting a polynomial curve of degree 3 to each dataset. Pearson correlation was used to assess the correlation between the fitted curve and the data.

### Peptide position analysis

For peptide position analysis within a protein sequence (Supplementary Fig. 3), proteome-derived datasets fitting to the length distribution of the 95% confidence level of the lncRNA dataset were selected. Then, the position of the HLAp, relative to the full protein sequence, was calculated for source lncRNA and proteome-derived sequences. Since the data were not normally distributed, the Wilcoxon test was utilized for statistical analysis.

### PRM analyses

For analyses of PRM statistics, MS-based intensities were taken from the initial MaxQuant peptide table output. TAAs for PRM and further comparative analyses were selected from a non-exhaustive list of known and clinically relevant TAAs.

### GTEx RNA expression analyses

Tissue-specific gene expression data was downloaded from GTEx, a public resource that contains data from 53 non-diseased tissues across nearly 1000 individuals^46^. We used a custom R script to retrieve gene expression values, based on publicly available GTEx v7 data. In the case of multiple transcripts matching the same entry, expression data for the most expressed transcript were used. The 90th percentile expression of the gene in the tissue- was reported. The FPKM expression units of the investigated sample were converted into TPM units for comparison with the GTEx data. The R package “ComplexHeatmap v1.99.4”^93^ from the Bioconductor suite was used to draw heatmaps.

### Reporting summary

Further information on research design is available in the Nature Research Reporting Summary linked to this article.

## Supplementary information

Supplementary Information

Description of Additional Supplementary Files

Supplementary Data 1

Supplementary Data 2

Supplementary Data 3

Supplementary Data 4

Supplementary Data 5

Supplementary Data 6

Supplementary Data 7

Supplementary Data 8

Reporting Summary

## Data Availability

Sequence data have been deposited into the European Genome-phenome Archive (EGA), which is hosted by the EBI and the CRG, under accession numbers EGAS00001003723 and EGAS00001003724. MS raw files, corresponding fasta reference files and NewAnce outputs have been deposited into the ProteomeXchange Consortium via the PRIDE^94^ partner repository with the dataset identifier PXD013649. The GENCODE v221.2 can be accessed through https://www.gencodegenes.org/human/release_22.html The human reference genome GRCh38/hg38 can be accessed through https://www.ncbi.nlm.nih.gov/assembly/5800238. Human ENCODE non-coding transcripts can be accessed through https://www.gencodegenes.org/human/release_24lift37.html. GTEx v7 can be accessed through https://www.gtexportal.org/home/datasets. The UniProt/TrEMBL database can be accessed through https://www.uniprot.org/proteomes/UP000005640. The source data underlying Figs. 1–9 and Supplementary Figs. 1–7, where applicable, are provided as a Source Data file. All other data are available from the corresponding author on reasonable request.

## Source data

Source Data

## Code availability

An executable jar file of NewAnce has been deposited to PRIDE with the dataset identifier PXD013649. The NewAnce code is available on the following GitHub link https://github.com/bassanilab/NewAnce.git.
