# Supplementary material for: Integrated proteogenomic deep sequencing and analytics accurately identify non-canonical peptides in tumor immunopeptidomes
Source: Nat Commun. 2020 Mar 10;11:1293. doi: 10.1038/s41467-020-14968-9 (PMC7064602; doi:10.1038/s41467-020-14968-9)
Supplement: Supplementary file 1 — Supplementary Information [file 41467_2020_14968_MOESM1_ESM.pdf]

Supplementary Information

Integrated Proteogenomic Deep Sequencing and Analytics Accurately Identify Non-Canonical Peptides in Tumor Immunopeptidomes

Chong et al.

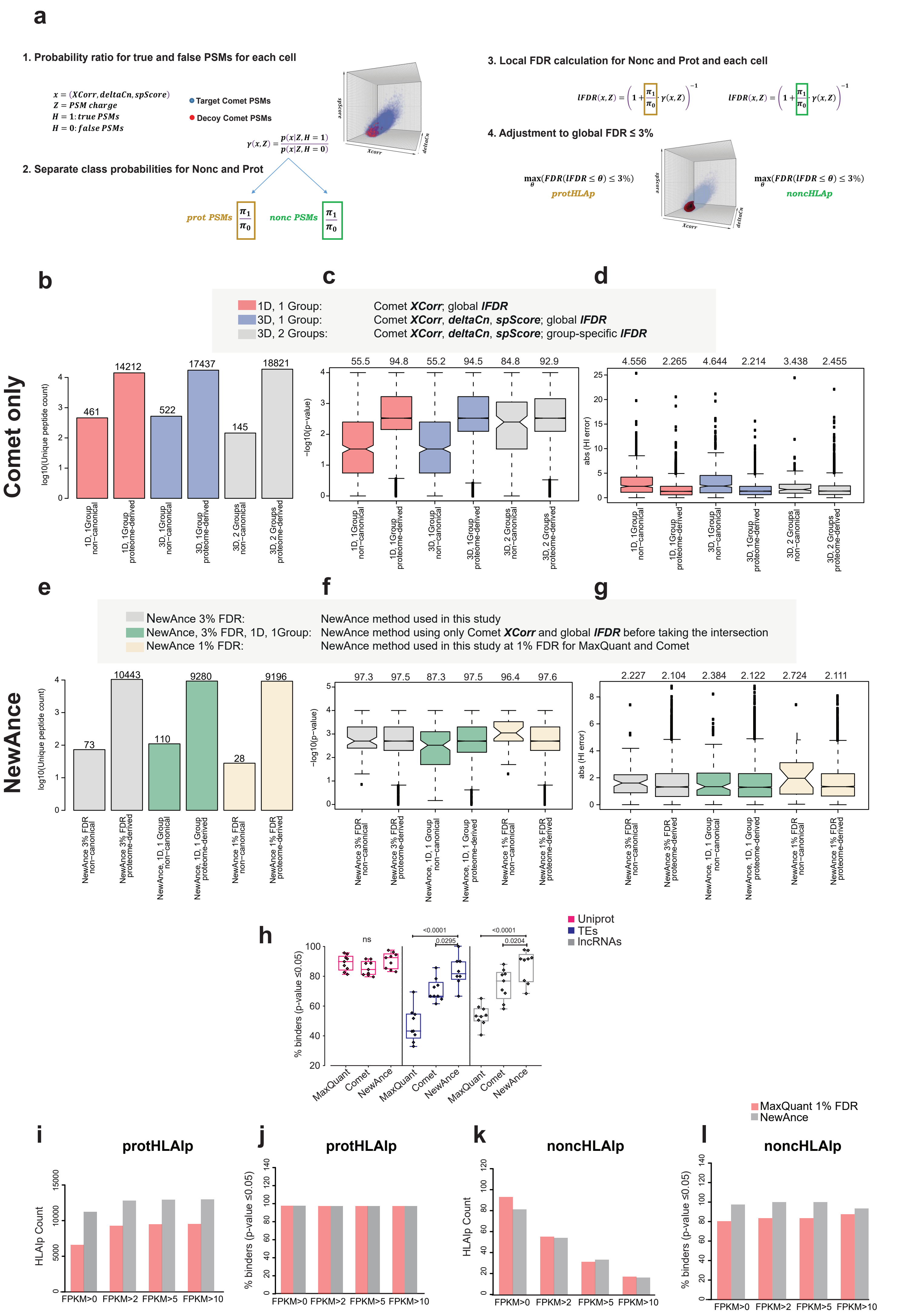
a
1. Probability ratio for true and false PSMs for each cell
x = (XCorr, deltaCn, spScore)
Z = PSM charge
H = 1: true PSMs
H = 0: false PSMs
Target Comet PSMs
Decoy Comet PSMs
2. Separate class probabilities for Nonc and Prot
prot PSMs
nonc PSMs
3. Local FDR calculation for Nonc and Prot and each cell
4. Adjustment to global FDR ≤ 3%
protHLAp
noncHLAp
b
c
d
1D, 1 Group: Comet XCorr; global lFDR
3D, 1 Group: Comet XCorr, deltaCn, spScore; global lFDR
3D, 2 Groups: Comet XCorr, deltaCn, spScore; group-specific lFDR
Comet only
log10(Unique peptide count)
−log10(p−value)
abs (HI error)
e
f
g
NewAnce 3% FDR: NewAnce method used in this study
NewAnce, 3% FDR, 1D, 1Group: NewAnce method using only Comet XCorr and global lFDR before taking the intersection
NewAnce 1% FDR: NewAnce method used in this study at 1% FDR for MaxQuant and Comet
NewAnce
h
Uniprot
TEs
lncRNAs
% binders (p-value ≤0.05)
MaxQuant
Comet
NewAnce
i
j
k
l
protHLAIp
noncHLAIp
MaxQuant 1% FDR
NewAnce
HLAIp Count
FPKM>0
FPKM>2
FPKM>5
FPKM>10

Supplementary Fig. 1 NewAnce for robust noncHLAIp identification. Group specific FDR calculation and the combination of two MS search tools enable the robust identification of noncHLAIp. **a** Schematic description of Comet FDR calculation workflow within NewAnce. 1) For PSMs of charge *Z*, the 3D Comet score space was divided into 40x40x40 cells. For every cell the probability ratios were calculated. 2) PSMs were split into non-canonical and proteome-derived groups and the class probability ratios were estimated for each group separately. 3) *lFDR* values were calculated for each cell and group. 4) Finally, the *lFDR* threshold corresponding to a global FDR of 3% was calculated and used to filter the PSMs. **b** The log10 of the number of unique peptides is shown for the comparison of 3 processing strategies tested for the identification of lncRNA- and proteome derived peptides at FDR of 3% in 0D5P sample. **c** The p-values for the MixMHCpred binding predictions are shown for the same comparisons as in b. The percentages of predicted binders (MixMHC p-values ≤ 0.05) are indicated as numbers above the boxplots. **d** The residual absolute errors of hydrophobicity index calculations by SSRCalc are shown for the same comparisons as in b. Standard errors are indicated as numbers above the boxplots. **e** The log10 of the number of unique peptides is shown for the comparison of 3 combiner options tested for the identification of lncRNA- and proteome derived peptides in the 0D5P sample. **f** The p-values for the MixMHCpred binding predictions are shown for the same comparisons as in e. The percentages of predicted binders (MixMHC p-values ≤ 0.05) are shown as numbers above the plots. **g** The residual absolute error of hydrophobicity index calculations by SSRCalc are shown for the same comparisons as in e. Standard errors are indicated as numbers above the boxplots. Please refer to the Methods section for boxplot parameters. **h** Systematic assessment of percentages of proteome-derived and predicted non-canonical HLA-I binders for each MS search tool (MaxQuant and Comet at FDR 3%) and NewAnce, for all n=11 samples, were performed. Ordinary one-way ANOVA, Sidak's multiple comparisons test was performed separately for Uniprot, TEs and lncRNAs. P-values between MaxQuant and NewAnce, and Comet and NewAnce are shown above the boxplots. ns: non-significant. **i-l** MaxQuant identified 0D5P prot- and noncHLAIp were compared to the NewAnce output, while reducing database sizes at FPKM thresholds. The total number of protHLAIp identified is plotted in **i**, and their corresponding percentage of predicted HLA binders in **j**. Similarly, the total number of noncHLAIp identified is plotted in **k** and the corresponding % of predicted HLA binders in **l**. Source data are provided as a Source Data file.

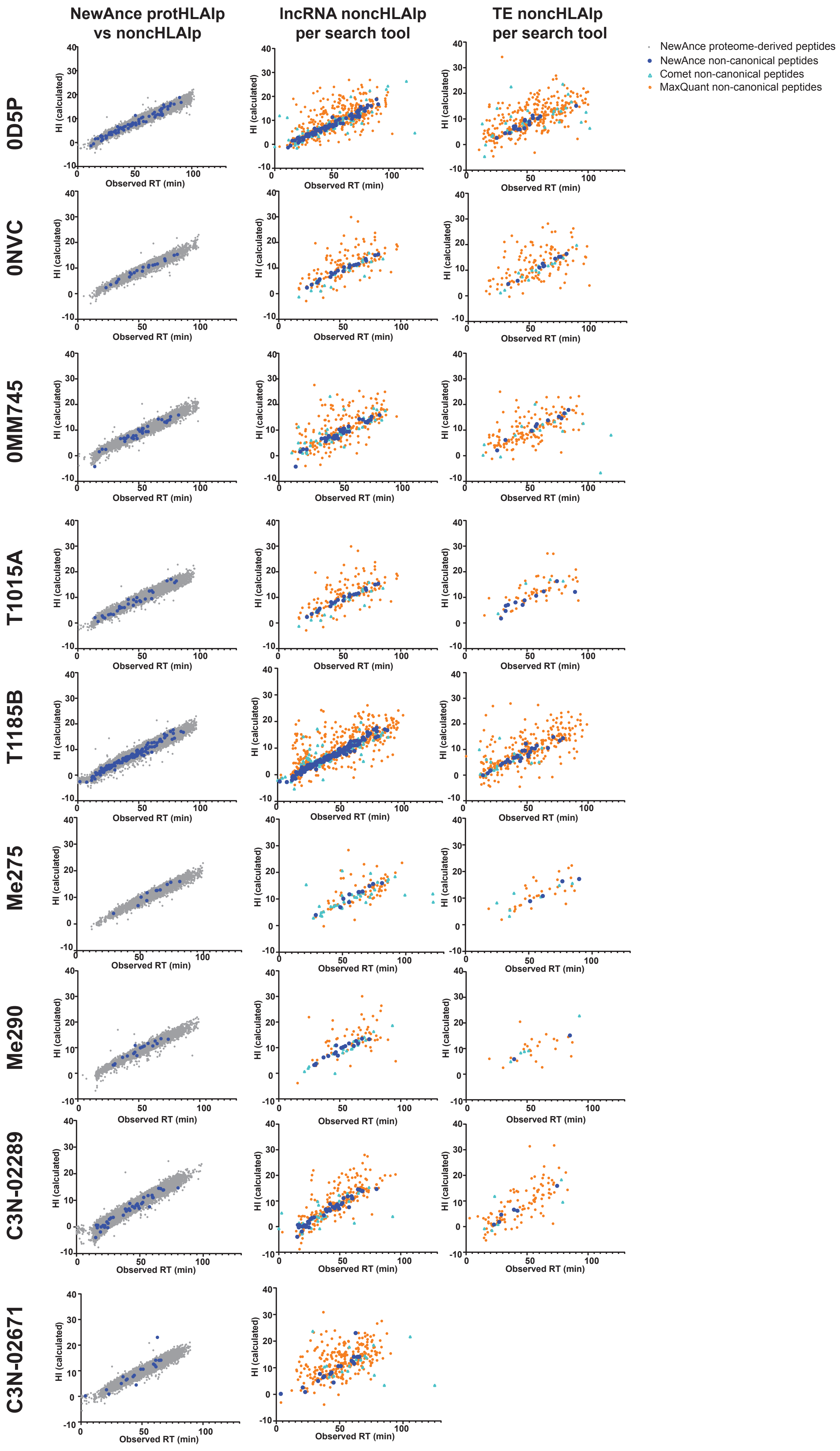

NewAnce protHLAIp vs noncHLAIp
lncRNA noncHLAIp per search tool
TE noncHLAIp per search tool
NewAnce proteome-derived peptides
NewAnce non-canonical peptides
Comet non-canonical peptides
MaxQuant non-canonical peptides
0D5P
0NVC
0MM745
T1015A
T1185B
Me275
Me290
C3N-02289
C3N-02671
HI (calculated)
Observed RT (min)

Supplementary Fig. 2 Hydrophobicity index calculation for all identified peptides. The data is shown for all assessed patient samples. In the leftmost panel, the observed mean retention time (RT) is plotted against the hydrophobicity indices (HI) for NewAnce-identified proteome-derived versus lncRNA-derived non-canonical peptides. All lncRNA-derived peptides (middle panel) or TE-derived peptides (rightmost panel) identified with each tool (MaxQuant, Comet, NewAnce) were analysed based on their hydrophobicity indices. Source data are provided as a Source Data file.

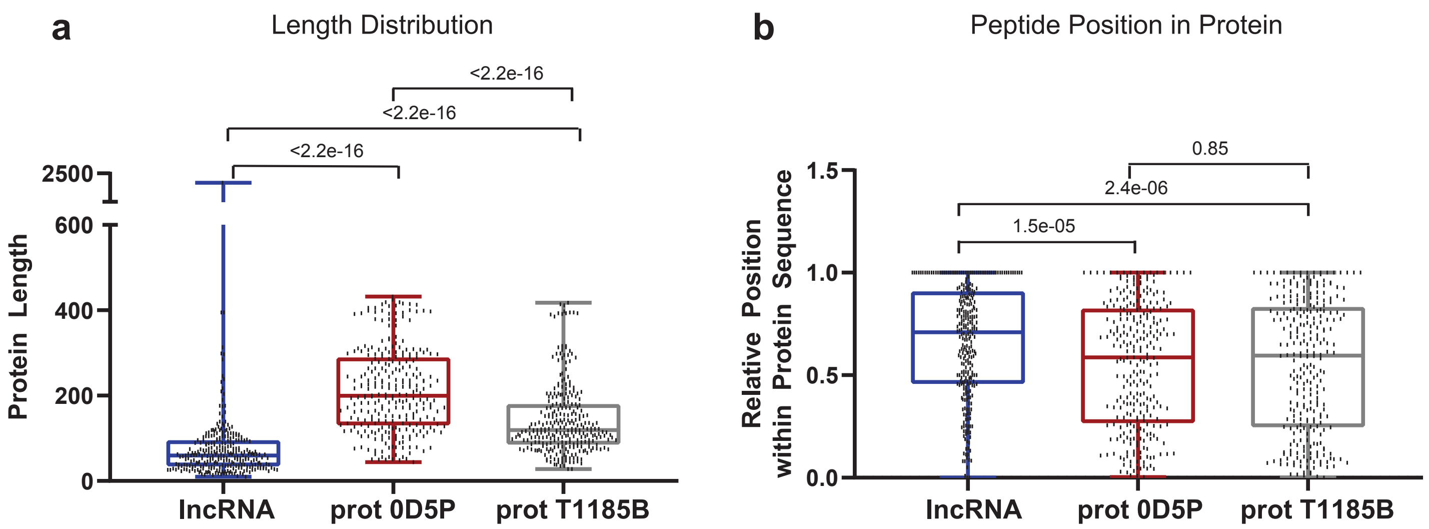

Supplementary Fig. 3 The origin of lncRNA-derived nonHLAps. LncRNA-derived noncHLAps are mainly derived from the C-terminus of the source translation products. **a** Protein length differences were assessed by sampling a matching-sized subset of both of the proteome-derived datasets fitting the length distribution of the lncRNA dataset (n=276) for a fair comparison. **b** Using the same dataset as that in (a), the corresponding HLA peptide's relative position (0 for N-terminus, 1 for C-terminus) was calculated for source lncRNA non-canonical and proteome-derived sequences. Statistical significance was performed with Wilcoxon testing. Please refer to the Methods section for boxplot parameters. Source data are provided as a Source Data file.

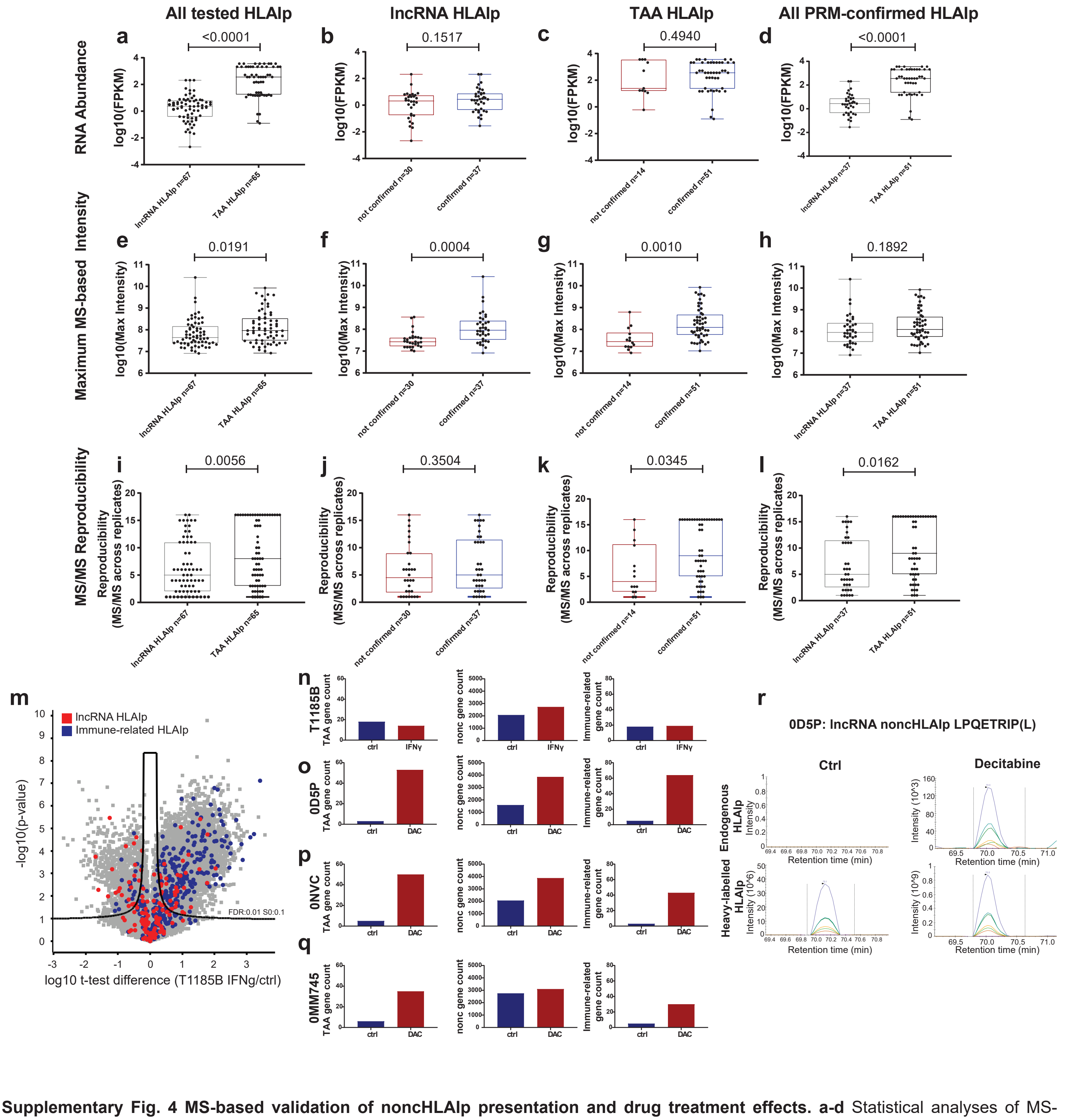

Supplementary Fig. 4 MS-based validation of noncHLAIp presentation and drug treatment effects. a-d Statistical analyses of MS-validated lncRNA-derived noncHLAIps and TAAs in the melanoma cell line 0D5P. The same comparisons were made first for all PRM-tested HLAIps regardless of the validation status (lncRNA HLAIp n=67, TAA HLAIp n=65); made second for lncRNA- (not confirmed n=30, confirmed n=37) and TAA HLAIps (not confirmed n=14, confirmed n=51) separately; and finally, made for only the PRM-confirmed HLAIps (lncRNA HLAIp n=37, TAA HLAIp n=51). RNA abundance in FPKM was extracted from the RNA-Seq data and compared within the corresponding groups. **e-h** MS-based intensity values were taken from the MaxQuant peptide output table and compared within the corresponding groups. **i-l** Last, MS/MS reproducibility, based on fragmentation by MS/MS per raw file (16 raw files for 0D5P in total), was analysed and compared within the corresponding groups. Unpaired two-sided t-test at 95% confidence interval. P-values are indicated above the plots. **m** Volcano plot depicting t-test analysis of HLAIps of IFNγ-treated versus untreated T1185B melanoma cells. Peptides located above the lines are statistically significantly up- or downregulated (FDR: 0.01, S0:0.1). All HLAIps derived from immunity-related genes are highlighted in dark blue, whereas all lncRNA-derived noncHLAIps are highlighted in red. **n** RNA expression analyses upon IFNγ treatment in T1185B. Induction of the total number of genes in per gene set was analysed for control and treated samples separately. The following gene sets were analysed: a selected set of TAA genes, all non-coding genes, and a subset of hypomethylating agent-induced immune-related genes (see main text). **o-q** Decitabine-treated melanoma cell lines were investigated at the RNA level. Only the total number of genes of interest that were exclusively expressed in each condition were taken into account. The same groups described above were analysed. For each gene category, decitabine induced the expression of more genes. **r** One example of a lncRNA-derived noncHLAIp that was induced by decitabine treatment in 0D5P, which was analysed by PRM. Co-elution of heavy and endogenous light transitions was found in only decitabine-treated samples. Source data are provided as a Source Data file.

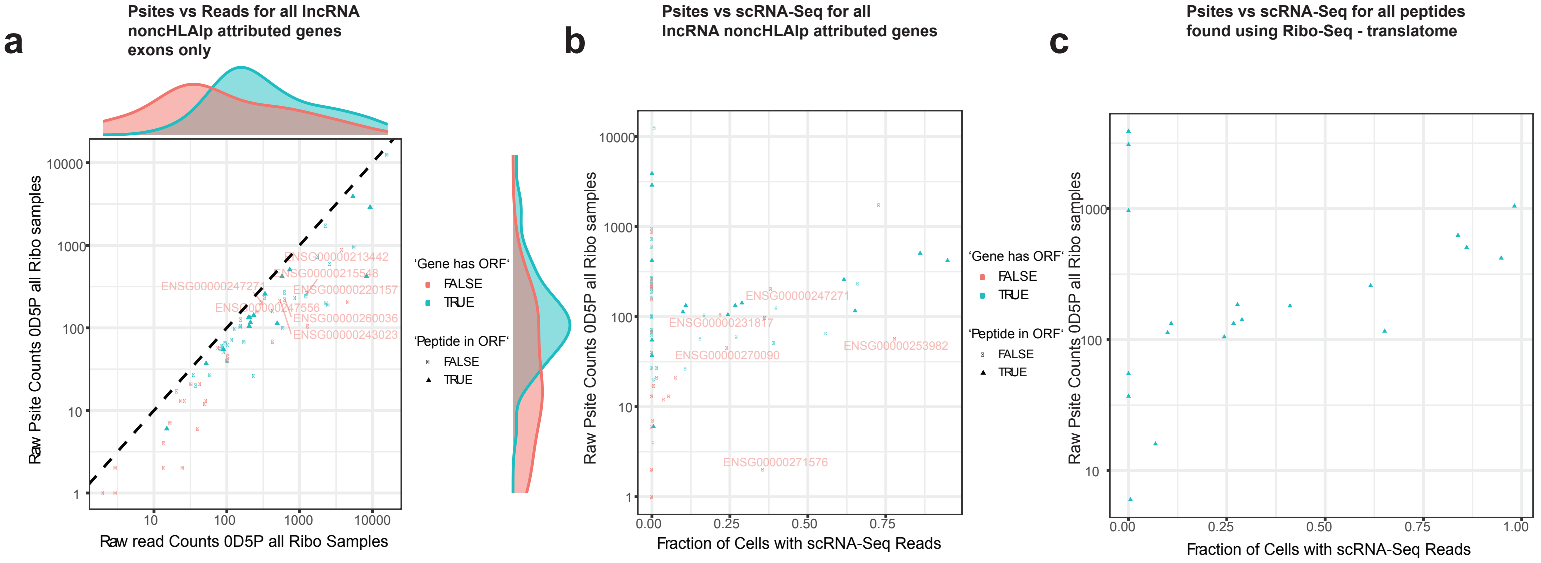

Supplementary Fig. 5 Limits of detection by Ribo-Seq analysis. **a** Scatterplot showing processed P-sites vs Raw Ribo-Seq reads for the noncHLAps detected using RNA-Seq data. Triangles indicate peptides that were contained within an ORF with a periodic Ribo-Seq signal. The blue symbol indicates peptides originating from genes that contained at least one periodic Ribo-Seq signal. We had difficulty determining correct P-site offsets for some read lengths, as the mapping quality and other factors reduced the number of P-sites available for the detection of periodicity, with detection becoming difficult for genes with low expression; however, all noncHLAIps showed at least some raw Ribo-Seq signals. **b** P-sites vs scRNA-Seq for noncHLAIps detected using RNA-Seq data. With some exceptions due to imperfect mappability, genes with few P-sites tended to show low scRNA-Seq signals and were detected in few cells. Note that only one gene (ENSG00000247271 - labelled) showed more than 100 P-sites across all samples and was detected in more than 10% of the cells. **c** scRNA-Seq signals for HLAIps detected using a Ribo-Seq-derived translatome. NoncHLAIps (blue) detected using the Ribo-Seq translatome showed a higher rate of detection in scRNA-Seq experiments, again indicating that SaTAnn identifies ORFs that show reproducible evidence of translation. Source data are provided as a Source Data file.

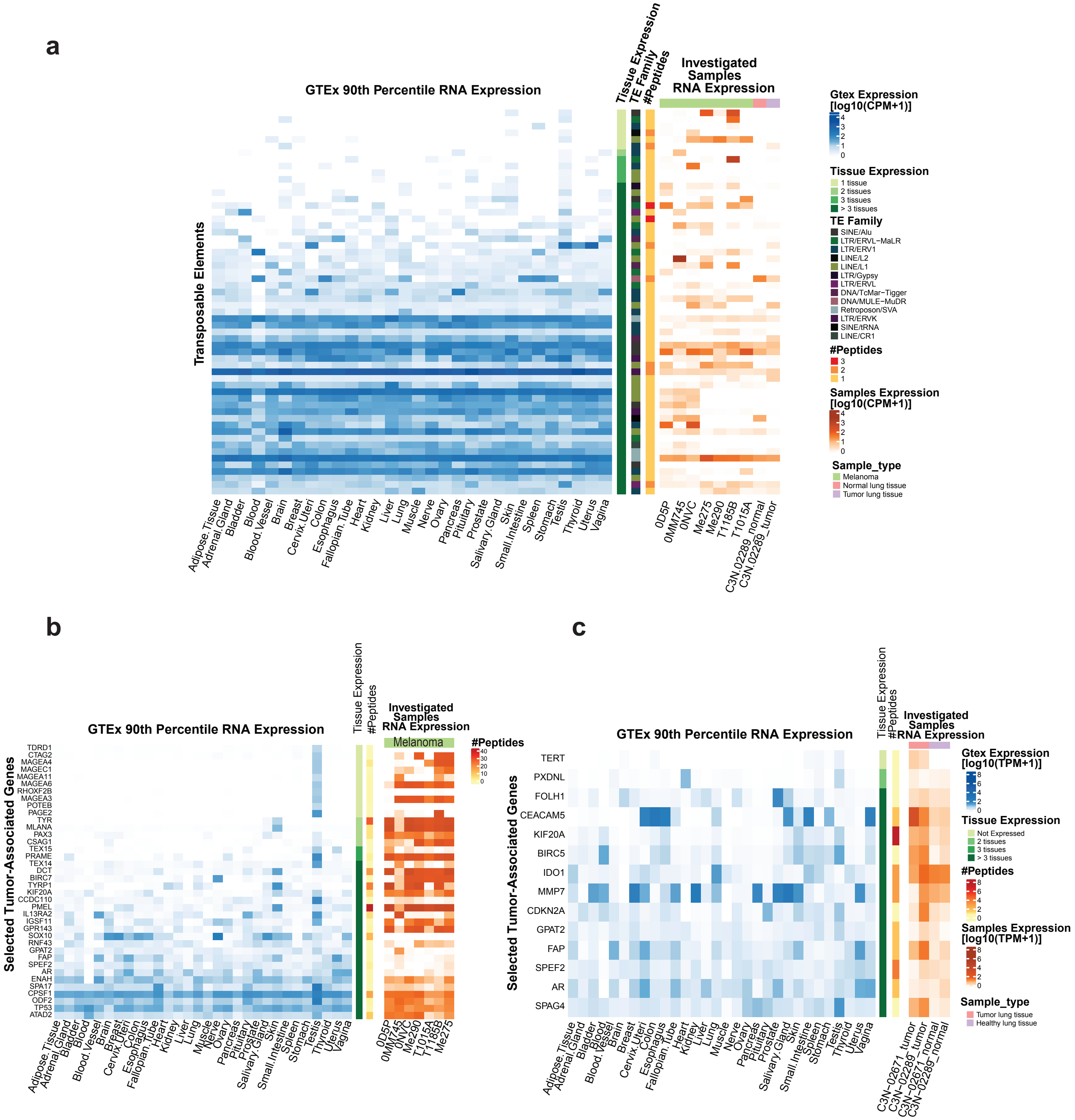

Supplementary Fig. 6 TE and tumor associated gene expression in healthy tissues. A comparison of presumed non-coding source gene expression in the investigated samples and healthy tissues (GTEx) for TE-derived HLAIps and TAAs. **a** Heatmap of TEs showing the 90th percentile gene expression levels in 30 healthy tissues on the left and the TE expression levels in our investigated samples on the right. Samples were classified as not expressed (90th percentile CPM ≤ 1) in any, 1-3, or more than 3 tissues other than testis to assess tumor specificity. The TEs were classified according to their TE family. Ten percent of the noncHLAIps derived from TEs were found to be expressed in only a single healthy tissue excluding the testis. **b** The same data described above are also plotted for selected source tumor-associated protein-coding genes in melanoma samples and for **c** lung tissue samples in TPM. Source data are provided as a Source Data file.

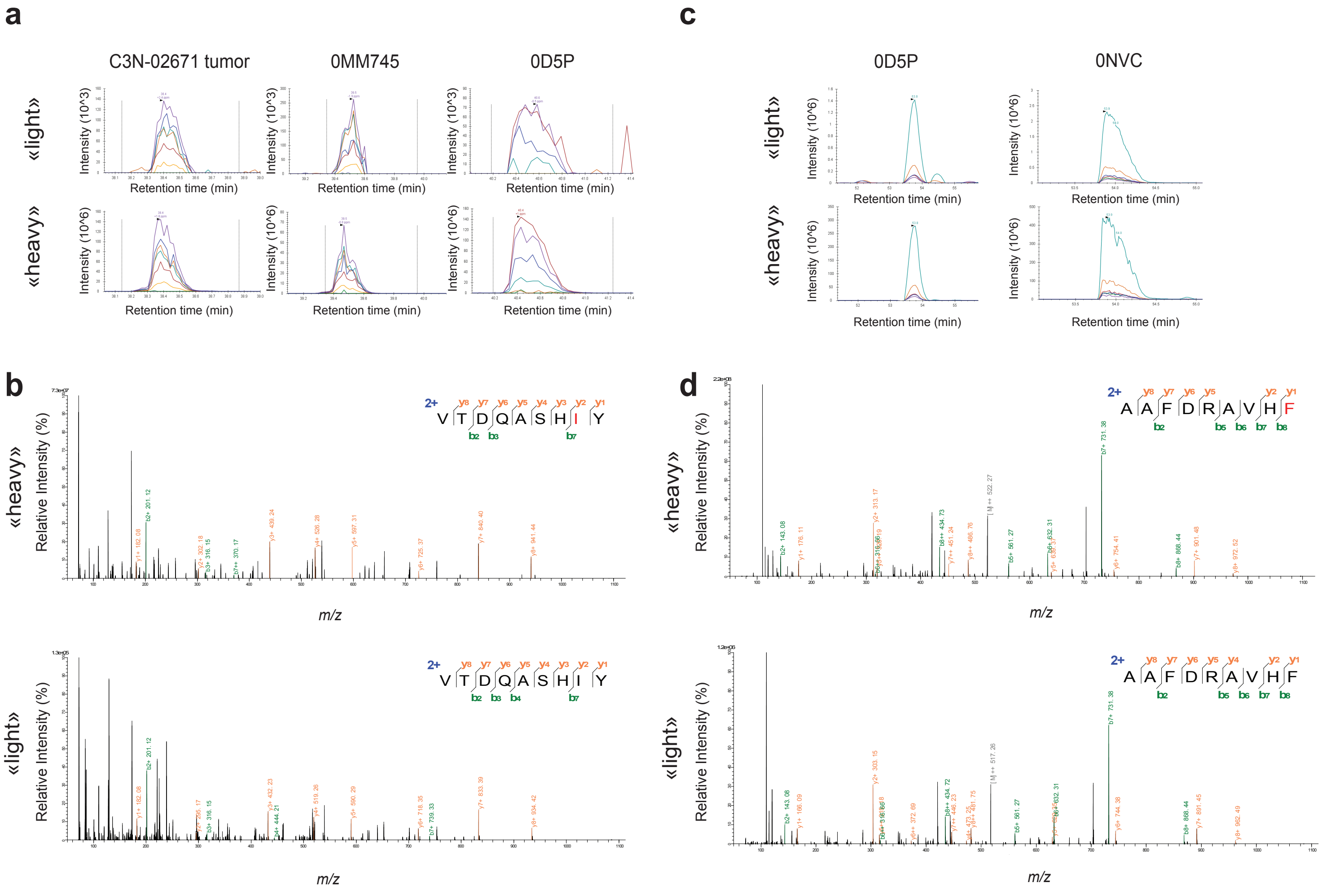

Supplementary Fig. 7 NoncHLAIp presentation can be shared across individuals. a Elution profiles of light and heavy labelled transitions and **b** representative MS/MS fragmentation pattern for the noncHLAIp VTDQASHIY. **c-d** The same representation is shown for TE-HLAIp AAFDRAVHF. Source data are provided as a Source Data file.

Supplementary Table 1 PRM-confirmed noncHLAIps that are shared across different samples. These patients express HLA allotypes that have identical or highly similar binding specificities.

Class	HLAIp	0D5P	0NVC	0MM745	C3N02671	Me275	T1015A	Motif	Motif
TE/lncRNA	**AAFDRAVHF**	C1203	C1203					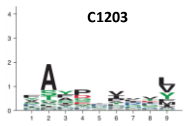	
lncRNA	**VTDQASHIY**	A0101		A0101	A0101			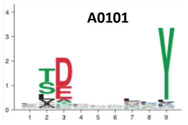	
lncRNA	**KSDLSKPLSY**	A0101			A0101			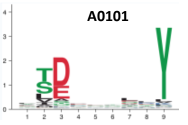	
lncRNA	**APKSSSGFSL**	B0702				B0702		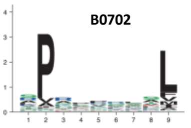	
lncRNA	**YLDPAQQNLY**	A0101			A0101			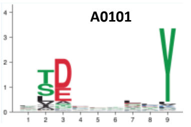	
lncRNA	**ETDIEMETRY**	A0101		A0101	A0101			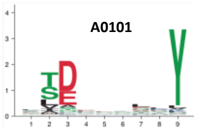	
TE	**KVFKNGNAF**	B1501					A3201	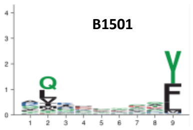	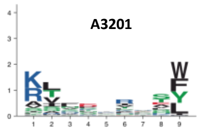

Supplementary Table 2 The number of reads for the various gene features are shown for each library of 0D5P sample used for ribosomal sequencing.

sample	coding sequences	5' untranslated regions	3' untranslated regions	non-coding exons of protein-coding genes	ncRNAs	introns	intergenic	total	coding sequence fraction
**0D5P_ctrl_1**	4400694	105786	83403	51027	346510	67675	155554	5210649	0.8445578
**0D5P_ctrl_2**	1536300	50939	50498	33487	178853	83700	193350	2127127	0.7222418
**0D5P_ctrl_3**	2636780	24335	43383	30810	258093	49229	213592	3256222	0.8097667
**0D5P_ctrl4B**	4510023	185817	219233	100180	645713	128480	326054	6115500	0.7374741
**0D5P_ctrl5B**	1404981	59688	76844	32808	217170	53621	157586	2002698	0.7015441
**0D5P_05_uM_DAC_1**	2431969	30950	41030	26017	226756	39433	232525	3028680	0.8029798
**0D5P_05_uM_DAC_2**	3450894	38133	76831	37924	271107	63493	268217	4206599	0.8203525
**0D5P_05_uM_DAC_3**	2973768	37896	53655	34141	233149	76593	239118	3648320	0.8151061
