# Supplementary material for: Integrated proteogenomic deep sequencing and analytics accurately identify non-canonical peptides in tumor immunopeptidomes
Source: Nat Commun. 2020 Mar 10;11:1293. doi: 10.1038/s41467-020-14968-9 (PMC7064602; doi:10.1038/s41467-020-14968-9)
Supplement: Supplementary file 2 — Description of Additional Supplementary Files [file 41467_2020_14968_MOESM2_ESM.docx]

**Description of Supplementary Files**

**File Name: Supplementary Data 1**

**Description:** Immunopeptidomics, sequencing and HLA-I typing information are provided for all the samples investigated in the present study.

**File Name: Supplementary Data 2**

**Description:** Report of the numbers of HLAp and percentages of predicted HLA binders identified across each MS search tool and when NewAnce was applied for every investigated sample along with their clinical characteristics.

**File Name: Supplementary Data 3**

**Description:** List of all PSMs of lncRNA-derived HLAIps and TE-derived HLAIps identified across the investigated samples in NewAnce. Additionally, Ribo-Seq-identified noncHLAIps are reported for melanoma 0D5P. More information on the PSMs can be found in the PRIDE repository with the dataset identifier PXD013649.

**File Name: Supplementary Data 4**

**Description:** All noncHLAp PSMs were extracted and searched against six common modifications, using Comet at 1% FDR. XCorr scores and other PSM parameters, are listed. Out of the 2,597 MSMS spectra, only 37 had a higher Comet XCorr score for a modified or an alternative UniProt peptide, corresponding to 17 unique non-canonical peptide sequences and 3.3% of total identified noncHLAp.

**File Name: Supplementary Data 5**

**Description:** A summary of the noncHLAp PSMs that were ambiguously identified matching another Uniprot peptide and/or modified peptide. Out of the 17 peptides, two peptides had several PSMs being unambiguously identified as the non-canonical peptides, and therefore the non-canonical sequences are likely to be correct. One of the two peptides was confirmed by our PRM validation. The rest of the PSMs that showed higher scores as compared to the non-canonical counterparts were identified as de-amidated (Uniprot) peptides (n=6 unique peptides), carbamidomethylated Uniprot peptides (n=3) and alternative Uniprot sequences (n=8). Only one phosphorylated noncHLAIp identified and it was among those ultimately fitting better a canonical UniProt sequence.

**File Name: Supplementary Data 6**

**Description:** PRM-tested HLAIps, including noncHLAIps and a subset of protHLAIps identified in 0D5P. The sequences, their origin, and their PRM status are shown along with their “heavy” and “light” theoretical masses of 2+.

**File Name: Supplementary Data 7**

**Description:** Targeted MS-based confirmation by PRM of selected prot- and noncHLAIps for 0D5P. For all confirmed sequences, the co-elutions of heavy and light transitions are shown, along with their respective MS/MS spectra.

**File Name: Supplementary Data 8**

**Description:** The samples that were used as input in ipMSDB are listed together with their corresponding information and PRIDE identification numbers.
