# Supplementary material for: Integrated proteogenomic deep sequencing and analytics accurately identify non-canonical peptides in tumor immunopeptidomes
Source: Nat Commun. 2020 Mar 10;11:1293. doi: 10.1038/s41467-020-14968-9 (PMC7064602; doi:10.1038/s41467-020-14968-9)

Supplementary Data 7. Targeted-MS based confirmation by PRM of selected prot- and noncHLAIp for 0D5P. For all confirmed sequences, co-elution of heavy and light transitions are shown, along with their respective MS/MS spectra.

SYLRRHLDF

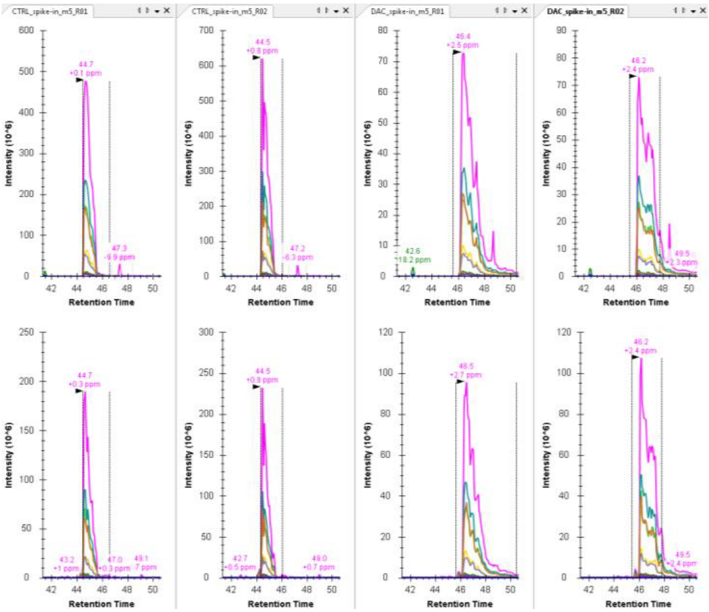

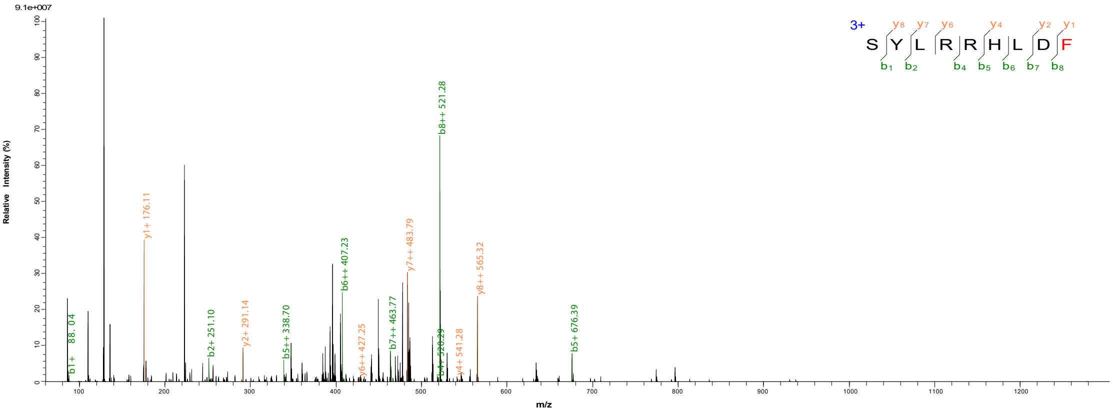

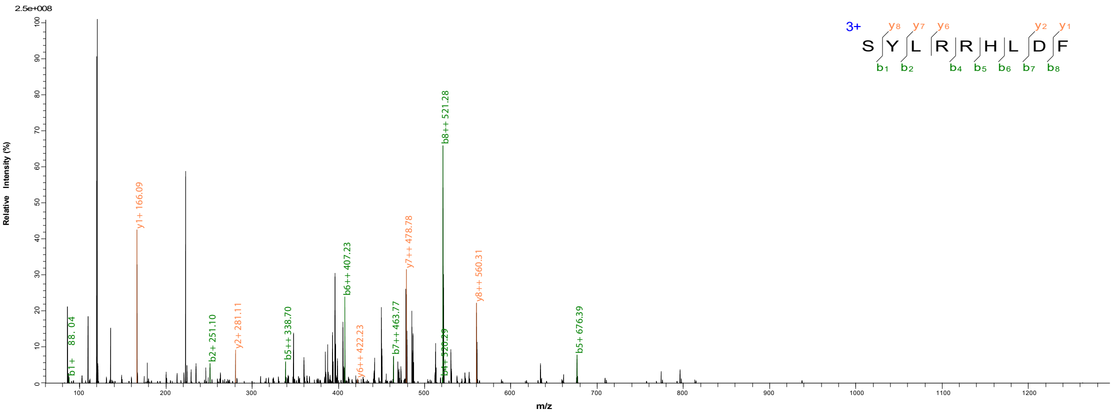

SPREPAGGGQV

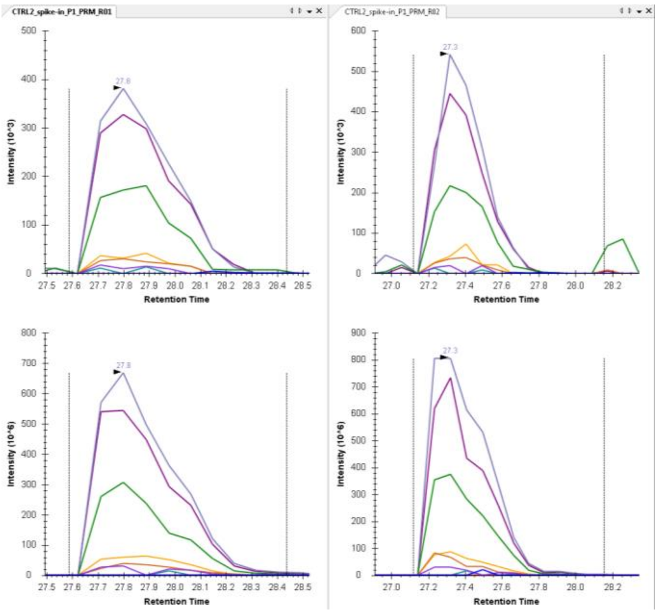

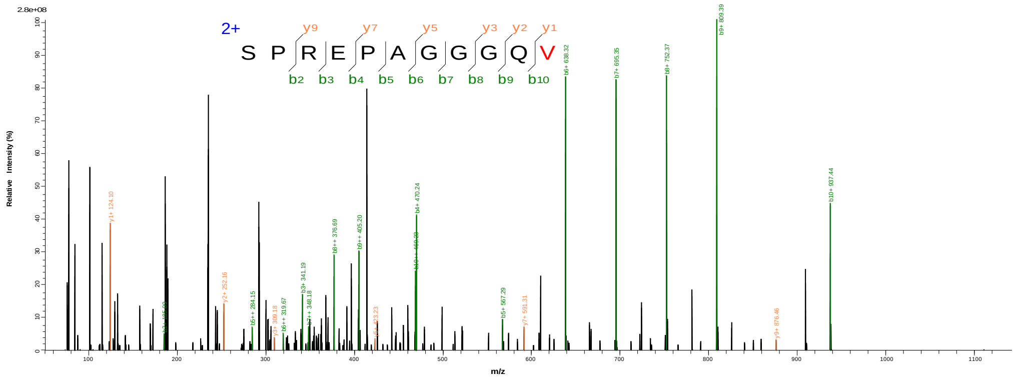

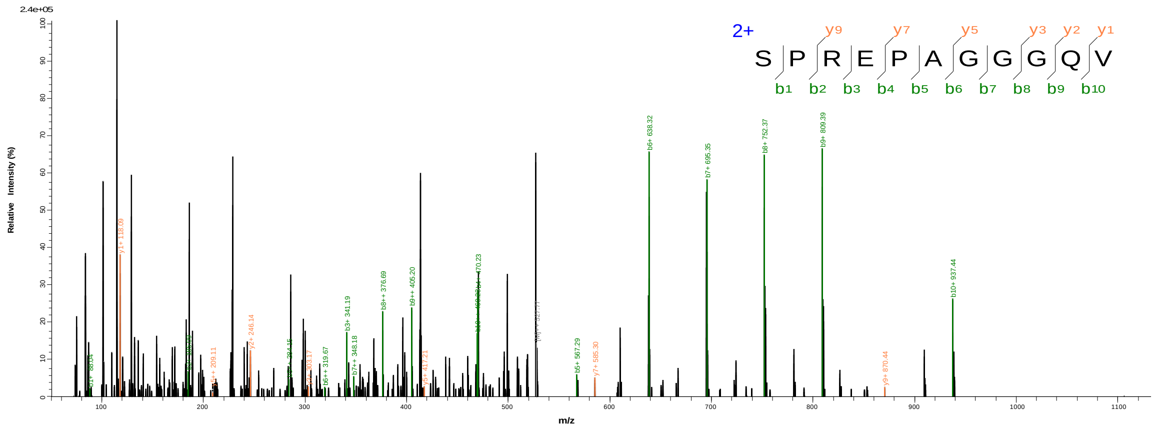

VTDQASHIY

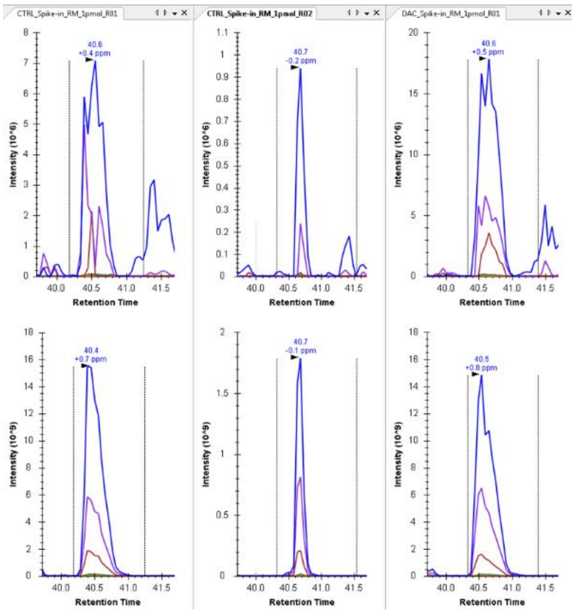
CTRL_Spike-in_RM_1pmol_R01
CTRL_Spike-in_RM_1pmol_R02
DAC_Spike-in_RM_1pmol_R01
Intensity (10^6)
Intensity (10^9)
Retention Time
40.6 +0.4 ppm
40.7 -0.2 ppm
40.6 +0.5 ppm
40.4 +0.7 ppm
40.7 -0.1 ppm
40.5 +0.8 ppm

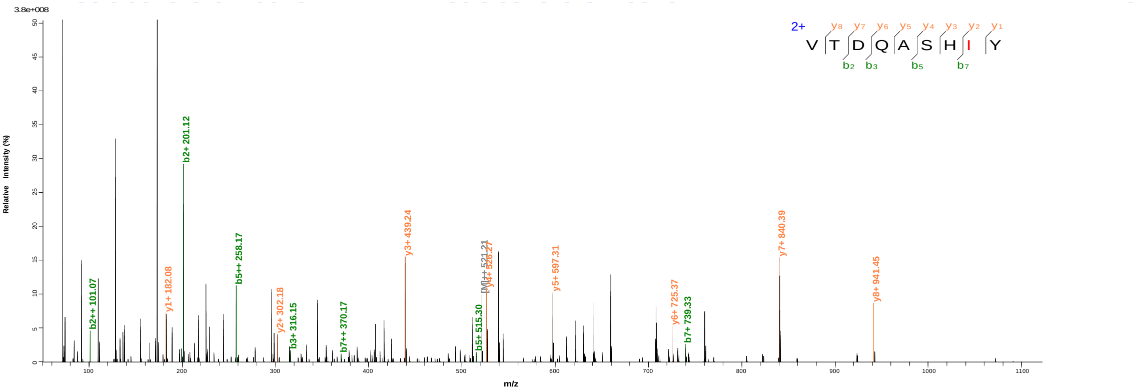
3.8e+008
Relative Intensity (%)
m/z
2+
V T D Q A S H I Y
b2+ 201.12
b5++ 258.17
y3+ 439.24
y7+ 840.39
y8+ 941.45
y5+ 597.31
y6+ 725.37
b7+ 739.33
b2++ 101.07
y1+ 182.08
y2+ 302.18
b3+ 316.15
b7++ 370.17
b5+ 515.30

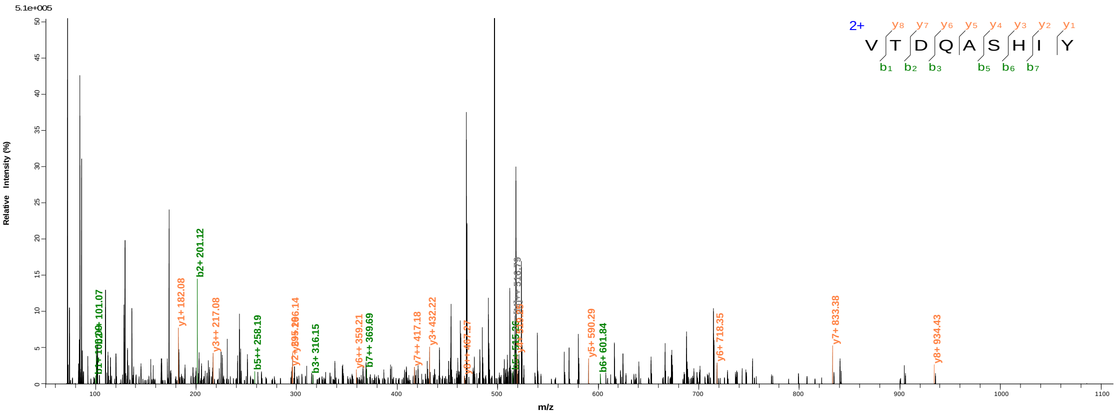
5.1e+005
Relative Intensity (%)
m/z
2+
V T D Q A S H I Y
b2+ 201.12
b2++ 101.07
y1+ 182.08
y3++ 217.08
b5+ 258.19
b3+ 316.15
b7++ 369.69
y7++ 417.18
y3+ 432.22
y5+ 590.29
b6+ 601.84
y6+ 718.35
y7+ 833.38
y8+ 934.43

ATEHDKNSTNIY

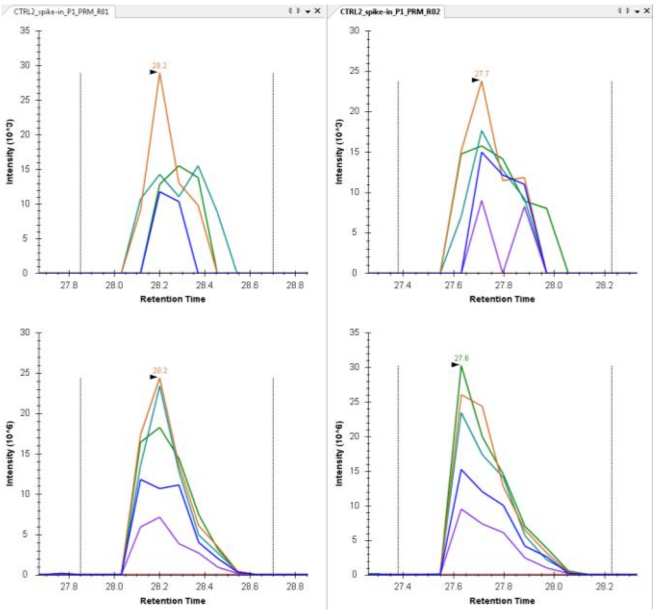

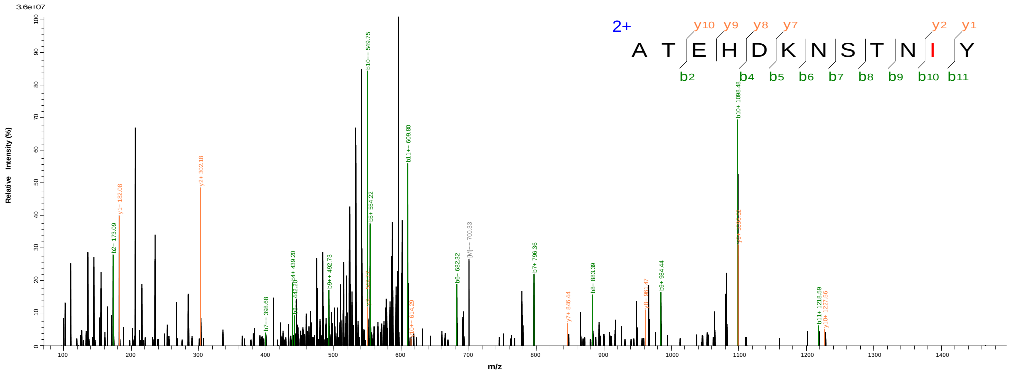

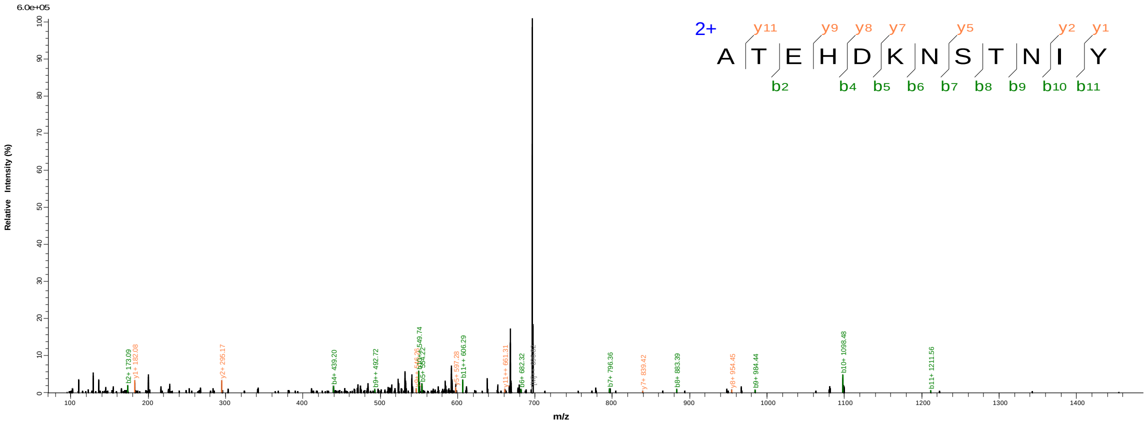

RVSHLTSSV

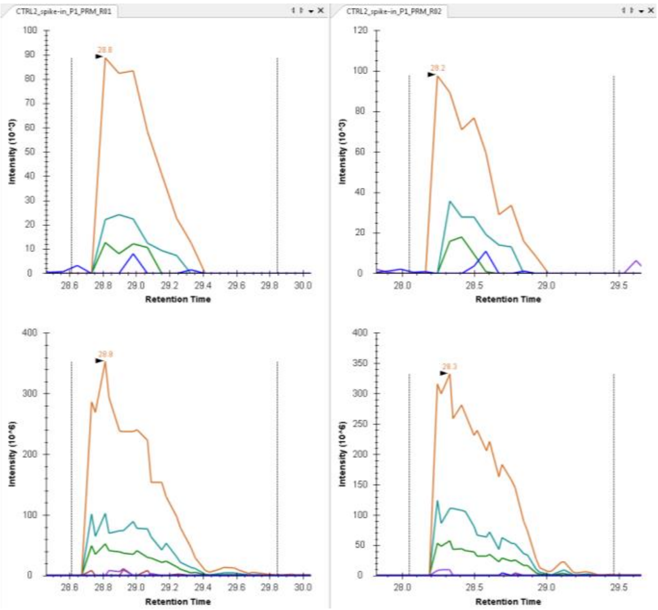

CTRL2_spike-in_P1_PRM_R01
CTRL2_spike-in_P1_PRM_R02
Intensity (10^3)
Intensity (10^6)
Retention Time

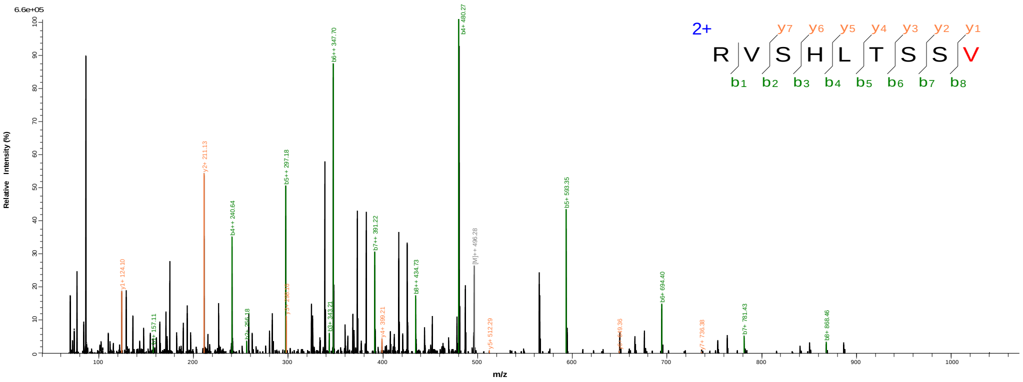

6.6e+05
Relative Intensity (%)
m/z
2+
R V S H L T S S V
y7 y6 y5 y4 y3 y2 y1
b1 b2 b3 b4 b5 b6 b7 b8

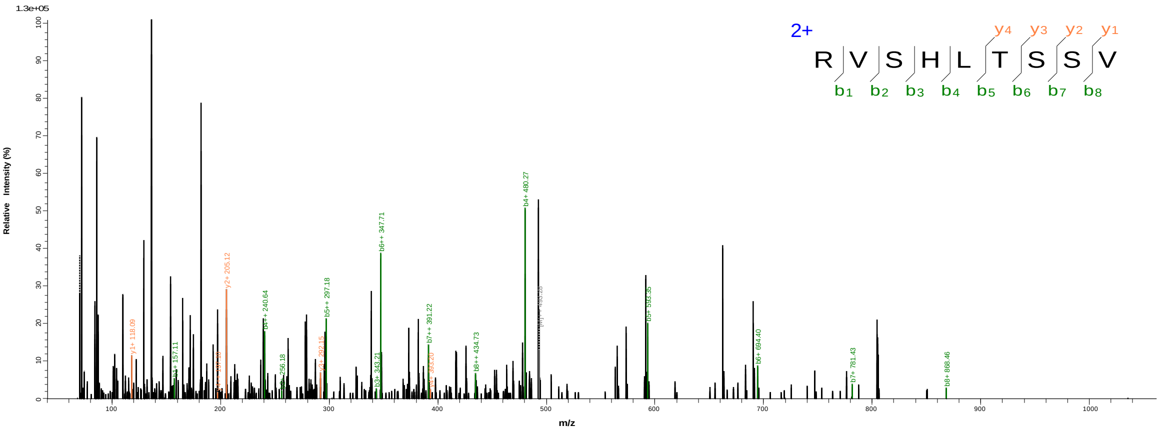

1.3e+05
Relative Intensity (%)
m/z
2+
R V S H L T S S V
y4 y3 y2 y1
b1 b2 b3 b4 b5 b6 b7 b8

IFNPVSKNF

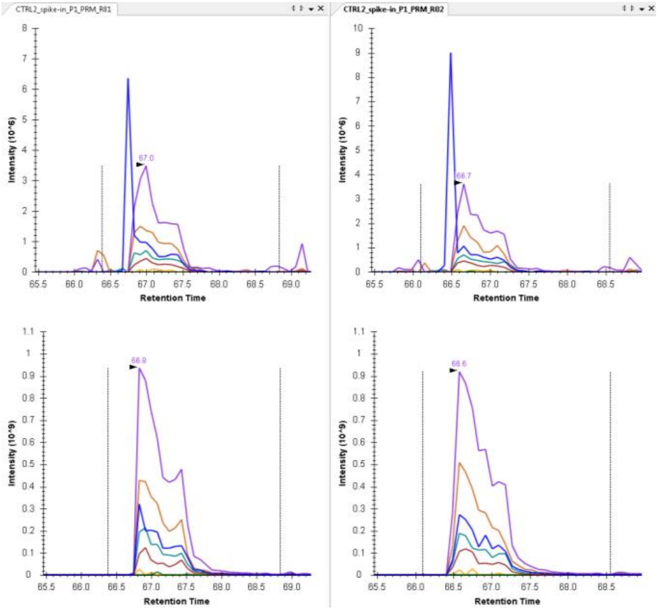

CTRL2_spike-in_P1_PRM_R01
CTRL2_spike-in_P1_PRM_R02
Intensity (10^6)
Intensity (10^9)
Retention Time

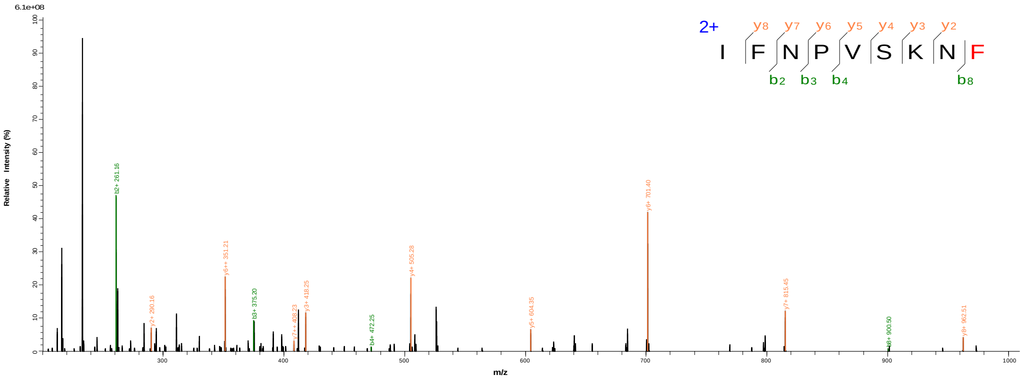

6.1e+08
Relative Intensity (%)
m/z
2+
y8 y7 y6 y5 y4 y3 y2
I F N P V S K N F
b2 b3 b4 b8

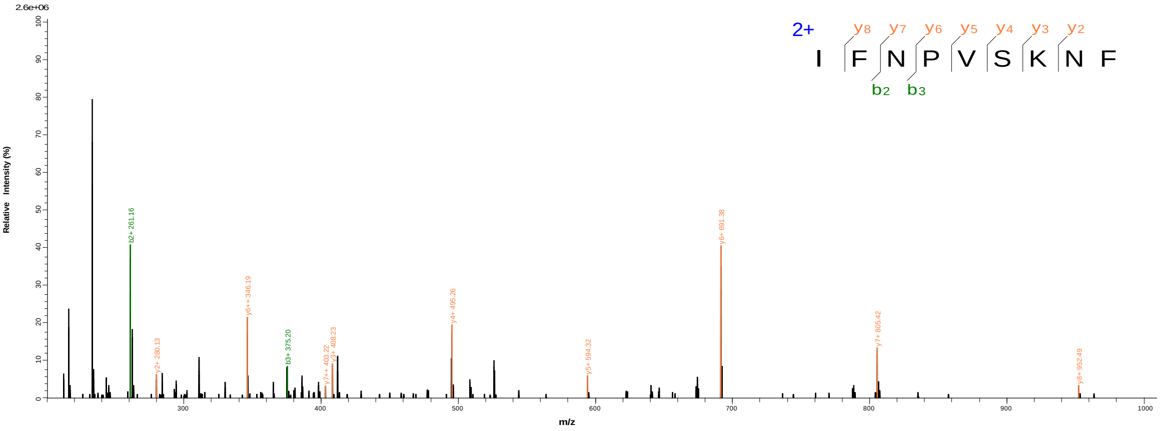

2.6e+06
Relative Intensity (%)
m/z
2+
y8 y7 y6 y5 y4 y3 y2
I F N P V S K N F
b2 b3

IWNGKGHFSF

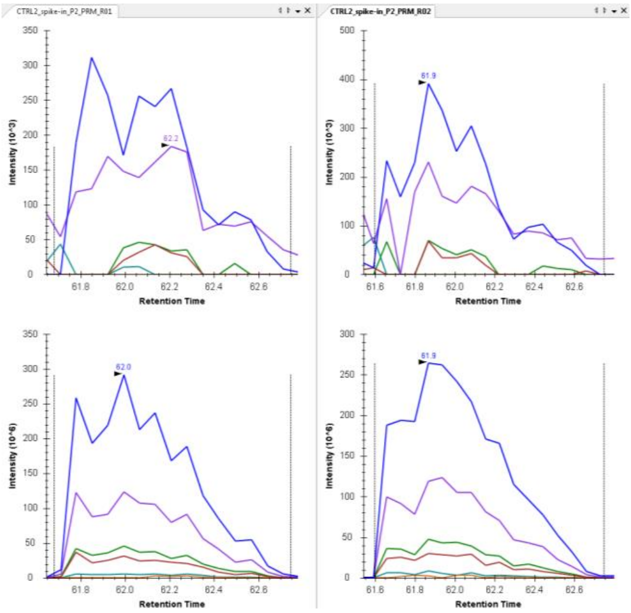

CTRL2_spike-in_P2_PRM_R01
CTRL2_spike-in_P2_PRM_R02
Intensity (10^3)
Intensity (10^6)
Retention Time
62.2
61.9
62.0
61.9

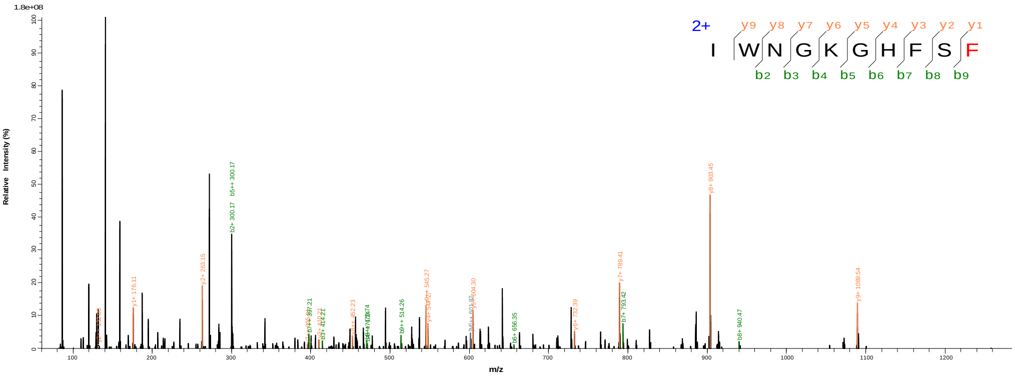

1.8e+08
2+
y9 y8 y7 y6 y5 y4 y3 y2 y1
I W N G K G H F S F
b2 b3 b4 b5 b6 b7 b8 b9
Relative Intensity (%)
m/z
b2+ 300.17
b5++ 300.17
y8+ 903.45
y9+ 1089.54
b7+ 793.42
b8+ 940.47

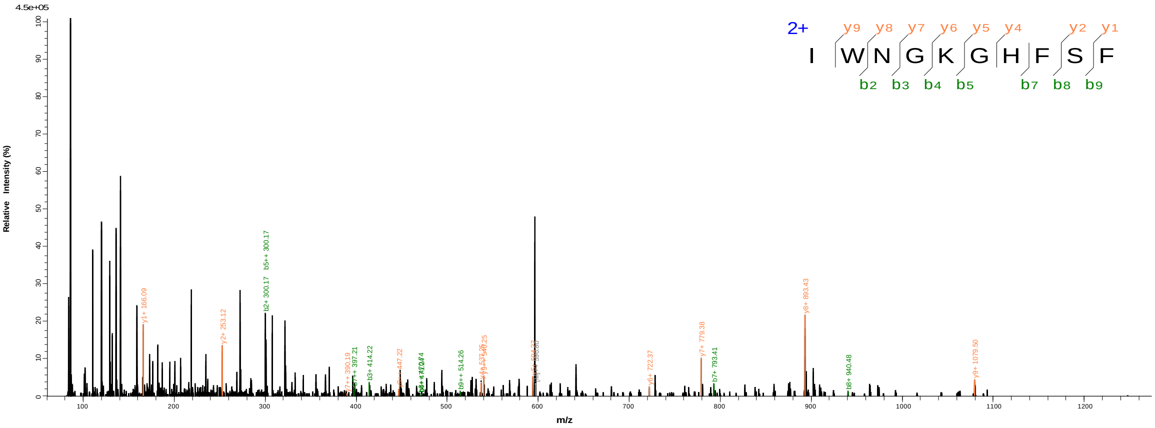

4.5e+05
2+
y9 y8 y7 y6 y5 y4 y2 y1
I W N G K G H F S F
b2 b3 b4 b5 b7 b8 b9
Relative Intensity (%)
m/z
y1+ 166.09
y2+ 253.12
b2+ 300.17
b5++ 300.17
b3+ 414.22
b9++ 514.26
y5+ 722.37
y7+ 779.38
b7+ 793.41
y8+ 893.43
b8+ 940.48
y9+ 1079.50

PLENAPIGHNRQY

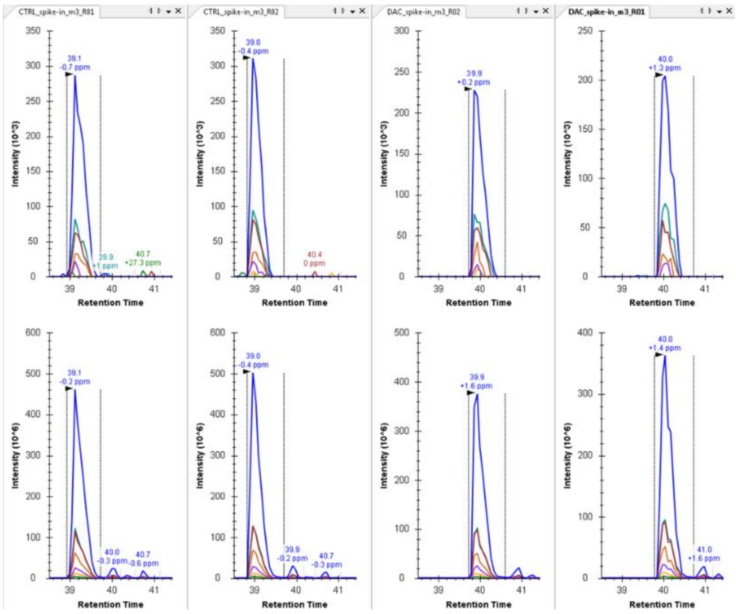

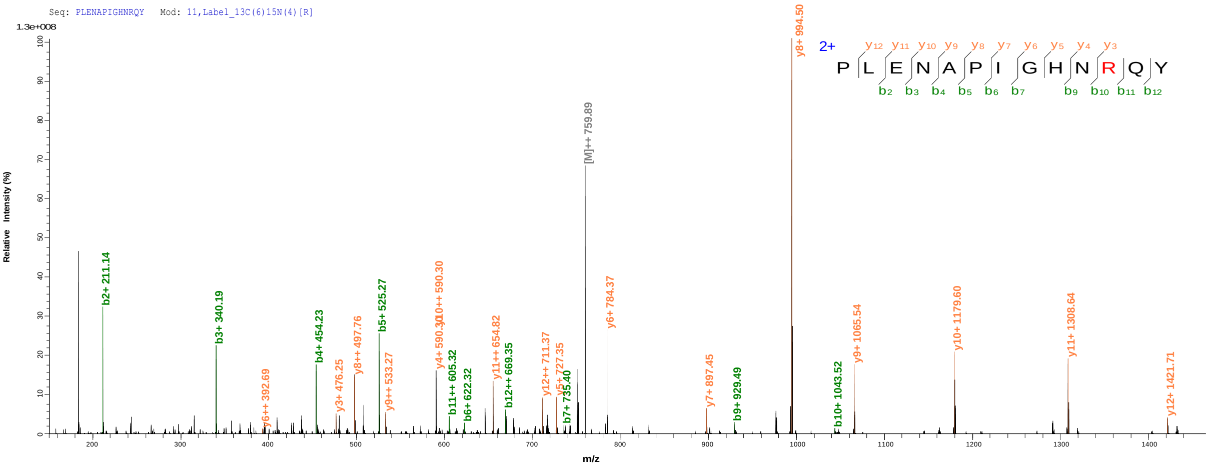

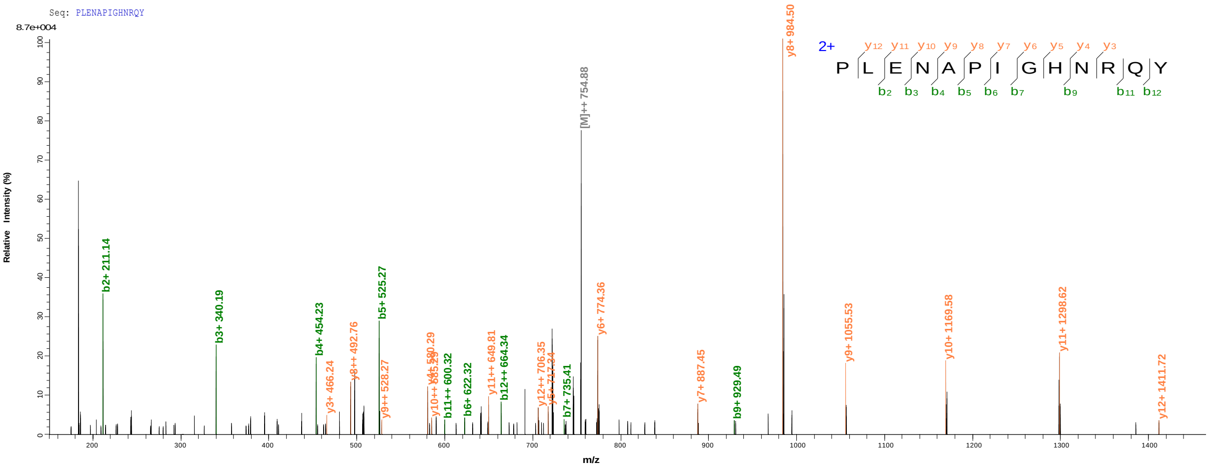

RYNADISTF

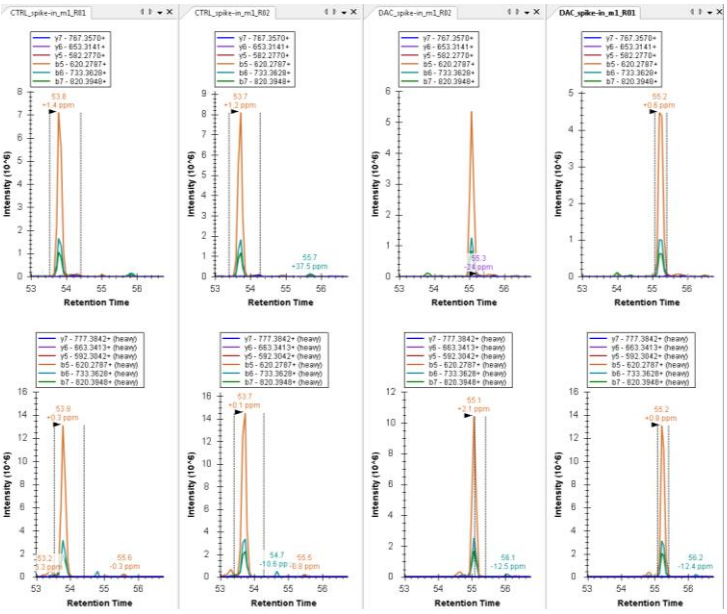
CTRL_spike-in_m1_R01
CTRL_spike-in_m1_R02
DAC_spike-in_m1_R02
DAC_spike-in_m1_R01
y7 - 767.3570+
y6 - 653.3141+
y5 - 582.2770+
b5 - 620.2787+
b6 - 733.3628+
b7 - 820.3948+
y7 - 777.3842+ (heavy)
y6 - 663.3413+ (heavy)
y5 - 592.3042+ (heavy)
b5 - 620.2787+ (heavy)
b6 - 733.3628+ (heavy)
b7 - 820.3948+ (heavy)
Intensity (10^6)
Retention Time

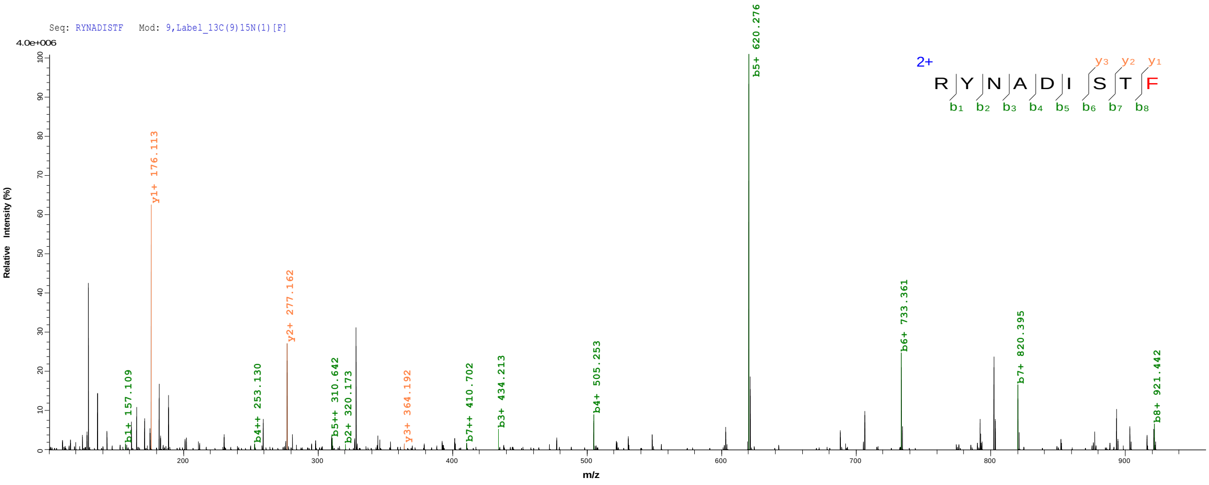
Seq: RYNADISTF Mod: 9,Label_13C(9)15N(1)[F]
4.0e+006
Relative Intensity (%)
m/z
b5+ 620.276
y1+ 176.113
y2+ 277.162
b6+ 733.361
b7+ 820.395
b8+ 921.442
b1+ 157.109
b4++ 253.130
b5++ 310.642
b2+ 320.173
y3+ 364.192
b7++ 410.702
b3+ 434.213
b4+ 505.253
2+
R Y N A D I S T F
b1 b2 b3 b4 b5 b6 b7 b8
y3 y2 y1

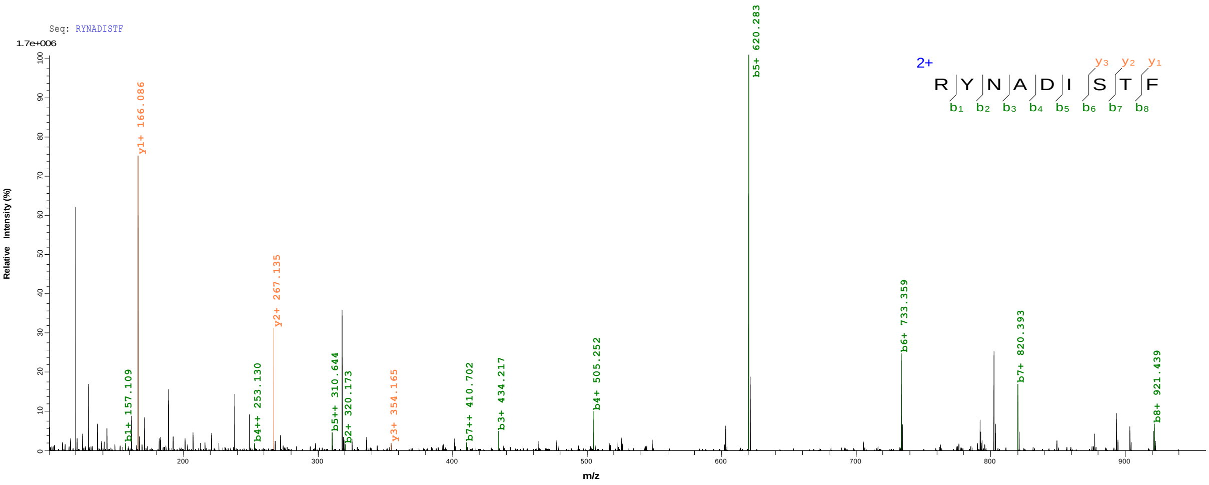
Seq: RYNADISTF
1.7e+006
Relative Intensity (%)
m/z
b5+ 620.283
y1+ 166.086
y2+ 267.135
b6+ 733.359
b7+ 820.393
b8+ 921.439
b1+ 157.109
b4++ 253.130
b5++ 310.644
b2+ 320.173
y3+ 354.165
b7++ 410.702
b3+ 434.217
b4+ 505.252
2+
R Y N A D I S T F
b1 b2 b3 b4 b5 b6 b7 b8
y3 y2 y1

ANRPPTQAL

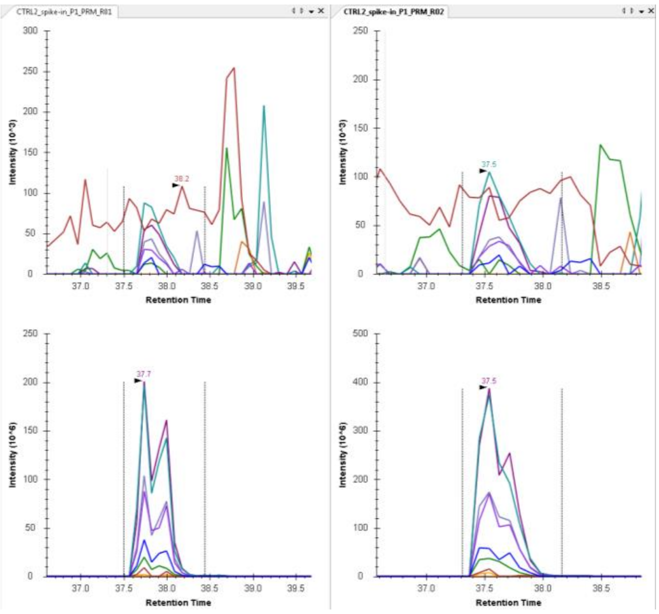

CTRL2_spike-in_P1_PRM_R01
CTRL2_spike-in_P1_PRM_R02
Intensity (10^3)
Intensity (10^6)
Retention Time

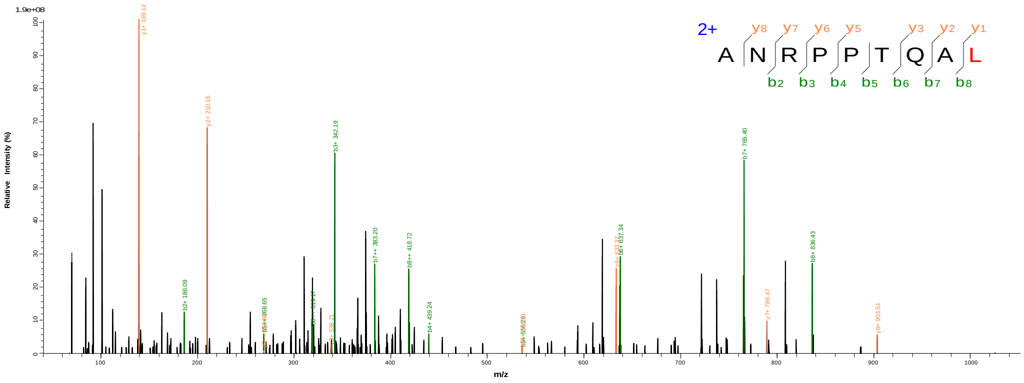

1.9e+08
Relative Intensity (%)
m/z
2+
A N R P P T Q A L
y8 y7 y6 y5 y3 y2 y1
b2 b3 b4 b5 b6 b7 b8

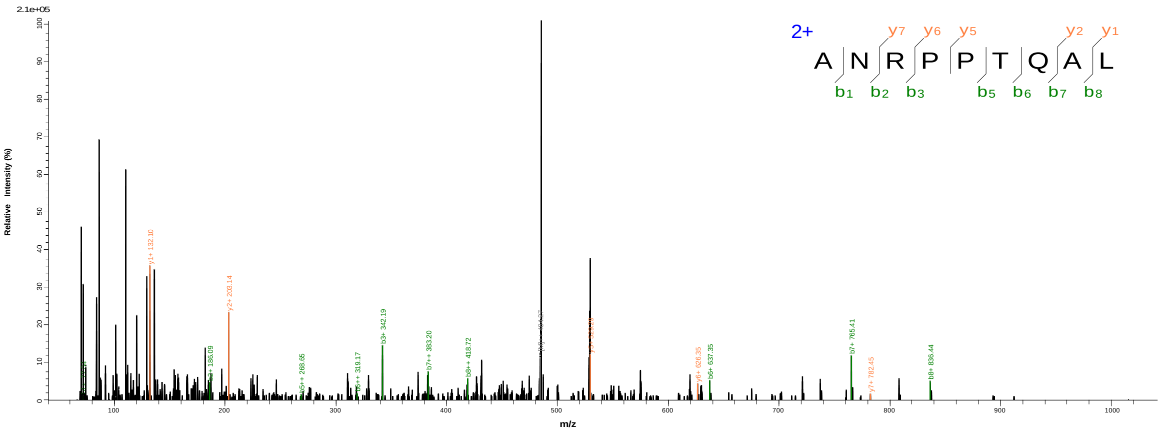

2.1e+05
Relative Intensity (%)
m/z
2+
A N R P P T Q A L
y7 y6 y5 y2 y1
b1 b2 b3 b5 b6 b7 b8

RPGPGTRKSL

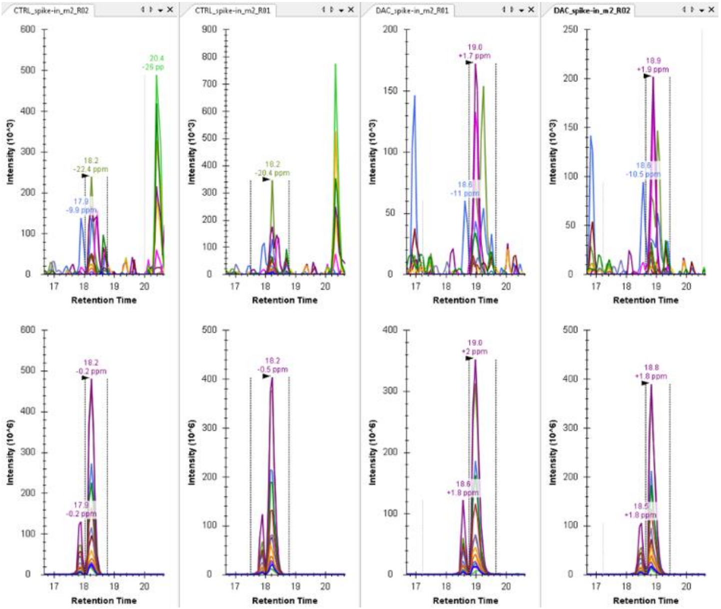

CTRL_spike-in_m2_R02
CTRL_spike-in_m2_R01
DAC_spike-in_m2_R01
DAC_spike-in_m2_R02
Intensity (10^3)
Intensity (10^6)
Retention Time

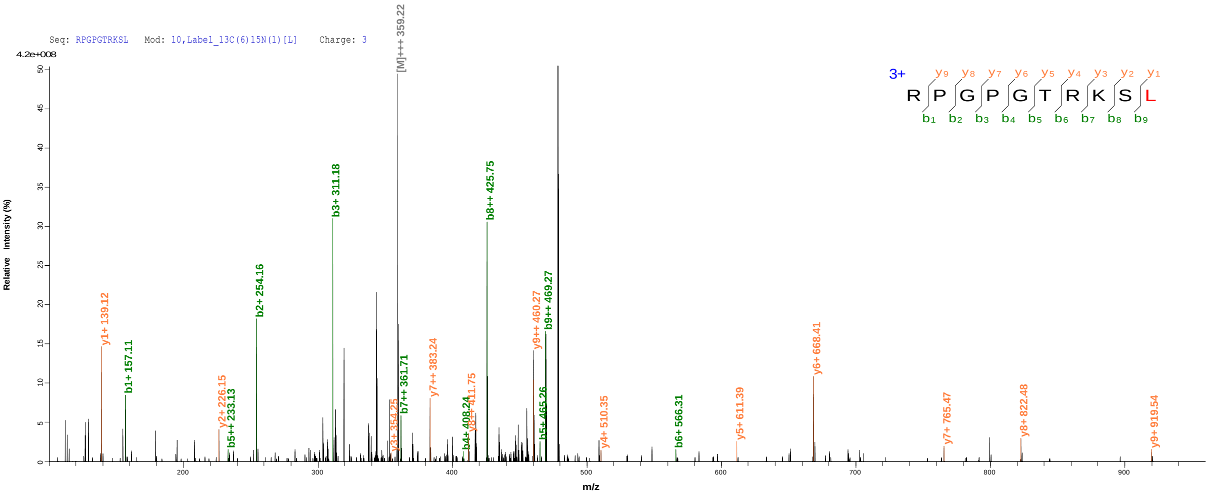

Seq: RPGPGTRKSL Mod: 10,Label_13C(6)15N(1)[L] Charge: 3
4.2e+008
Relative Intensity (%)
m/z
[M]+++ 359.22
3+
R P G P G T R K S L
y9 y8 y7 y6 y5 y4 y3 y2 y1
b1 b2 b3 b4 b5 b6 b7 b8 b9
y1+ 139.12
b1+ 157.11
y2+ 226.15
b5++ 233.13
b2+ 254.16
b3+ 311.18
b7++ 361.71
y7++ 383.24
b4+ 408.24
b8++ 425.75
y9++ 460.27
b9++ 469.27
y4+ 510.35
b6+ 566.31
y5+ 611.39
y6+ 668.41
y7+ 765.47
y8+ 822.48
y9+ 919.54

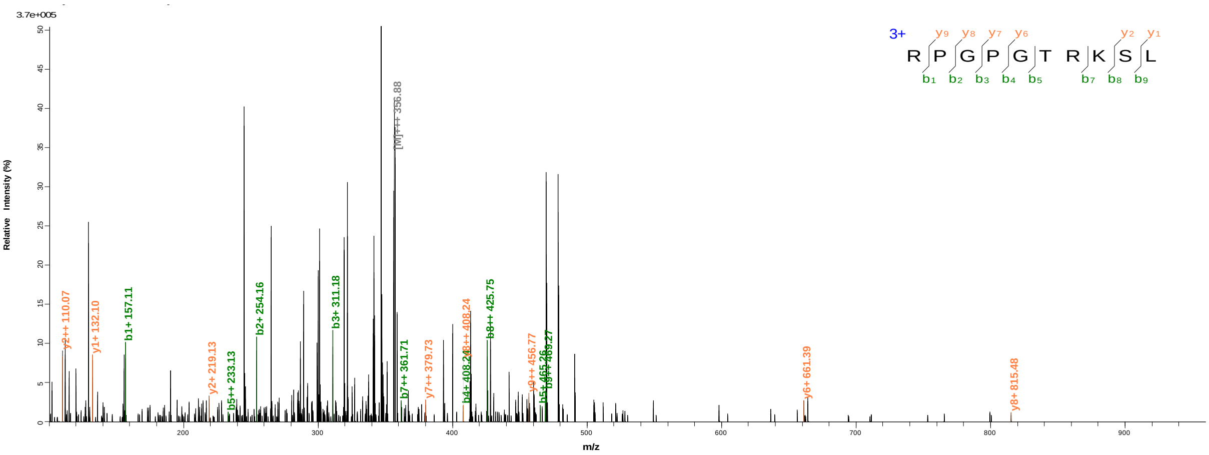

3.7e+005
Relative Intensity (%)
m/z
[M]+++ 356.88
3+
R P G P G T R K S L
y9 y8 y7 y6 y2 y1
b1 b2 b3 b4 b5 b7 b8 b9
y2++ 110.07
y1+ 132.10
b1+ 157.11
y2+ 219.13
b5++ 233.13
b2+ 254.16
b3+ 311.18
b7++ 361.71
y7++ 379.73
b4+ 408.24
b8++ 425.75
y9++ 456.77
y6+ 661.39
y8+ 815.48

FSDFPGGDKGKFY

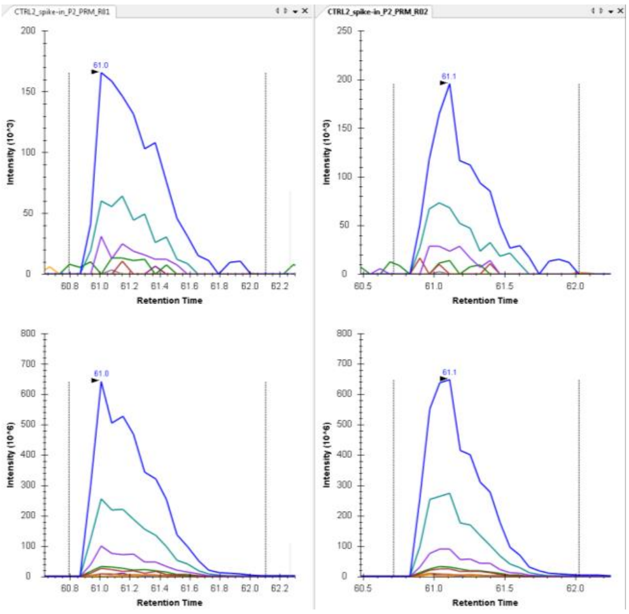

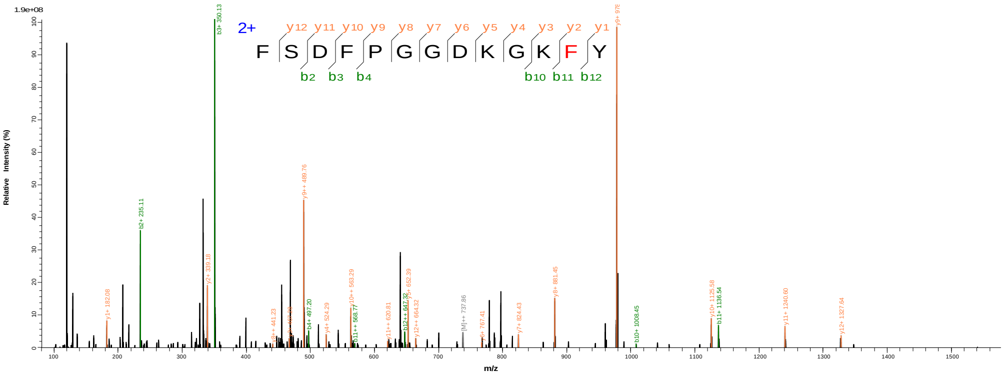

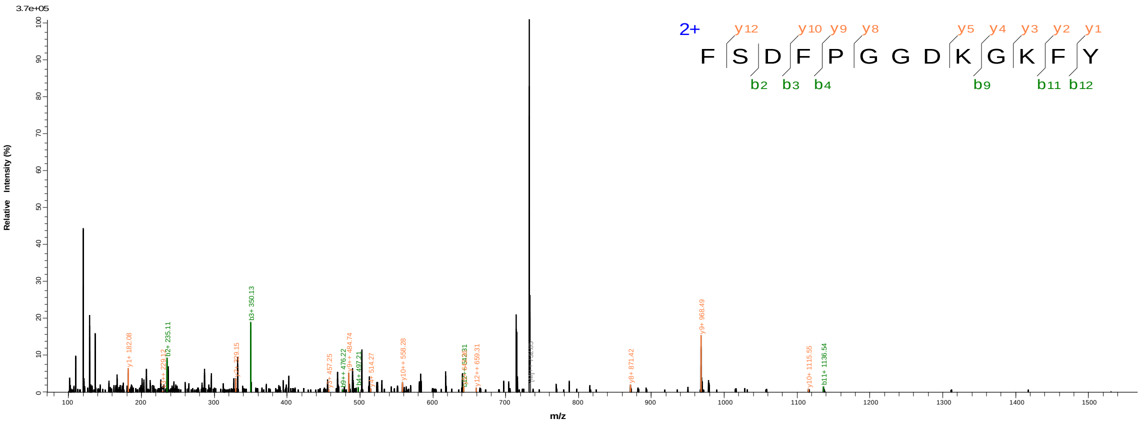

AAFDGRHSQTL

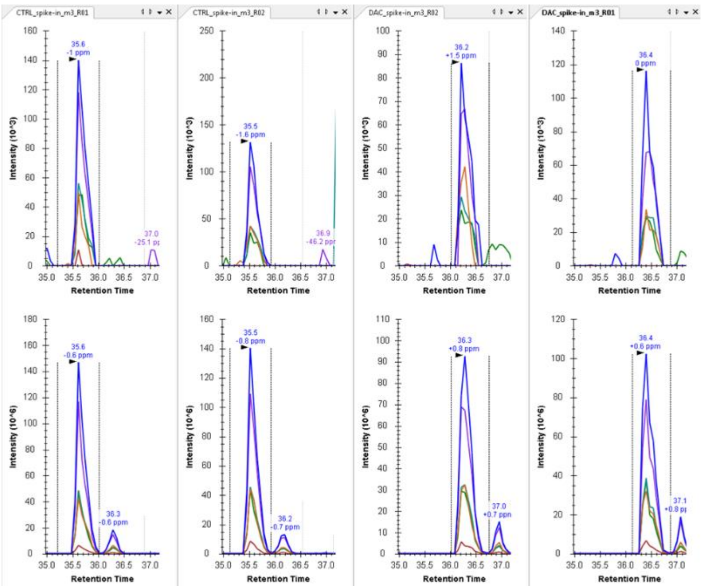

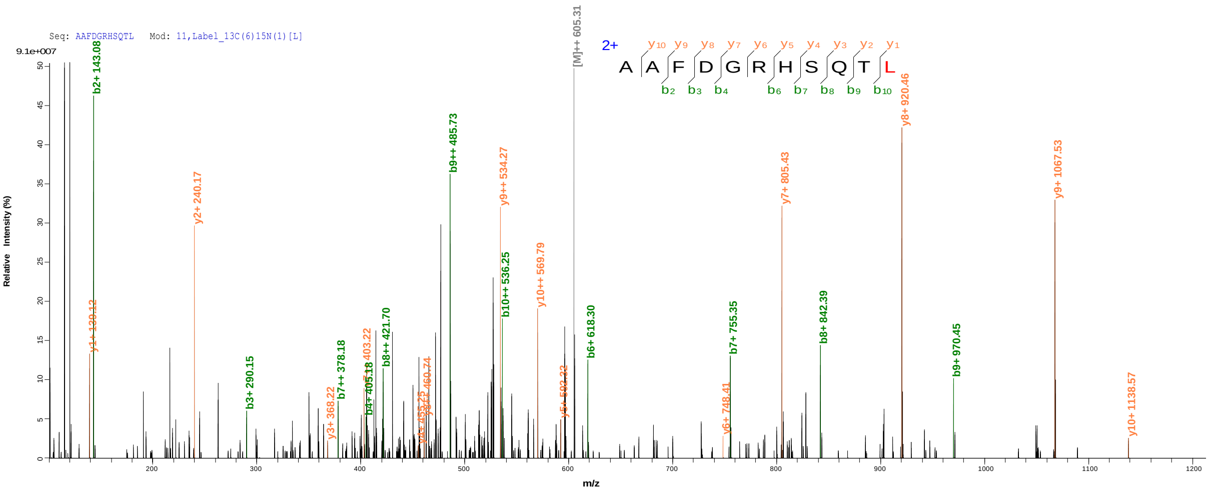

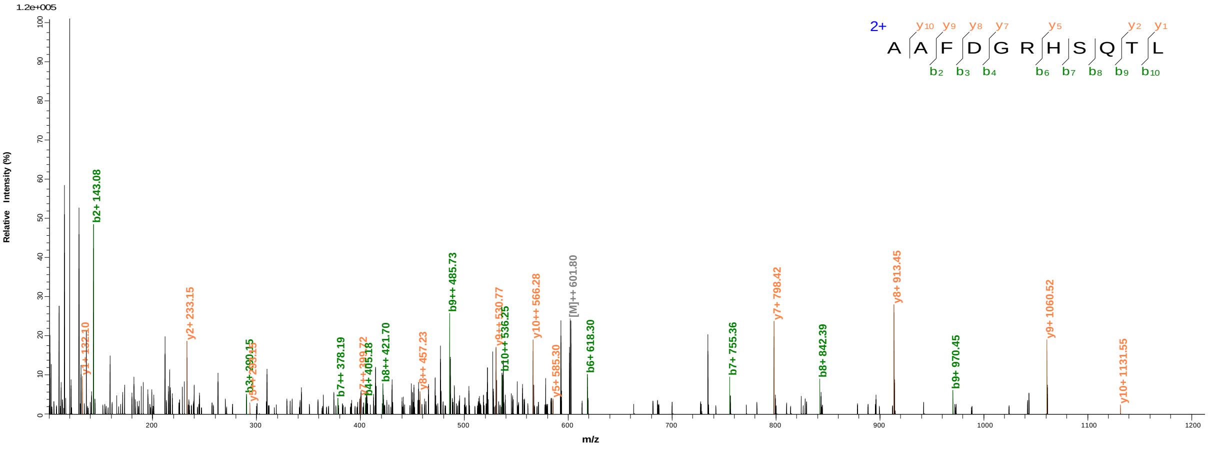

GQHLHLETF

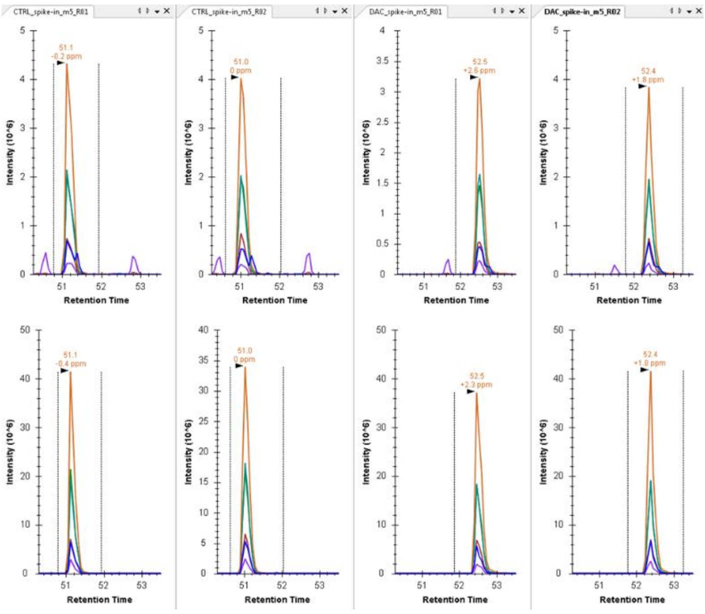

CTRL_spike-in_m5_R01
CTRL_spike-in_m5_R02
DAC_spike-in_m5_R01
DAC_spike-in_m5_R02
Intensity (10^6)
Retention Time
51.1 -0.2 ppm
51.0 0 ppm
52.5 +2.6 ppm
52.4 +1.8 ppm
51.1 -0.4 ppm
51.0 0 ppm
52.5 +2.3 ppm
52.4 +1.9 ppm

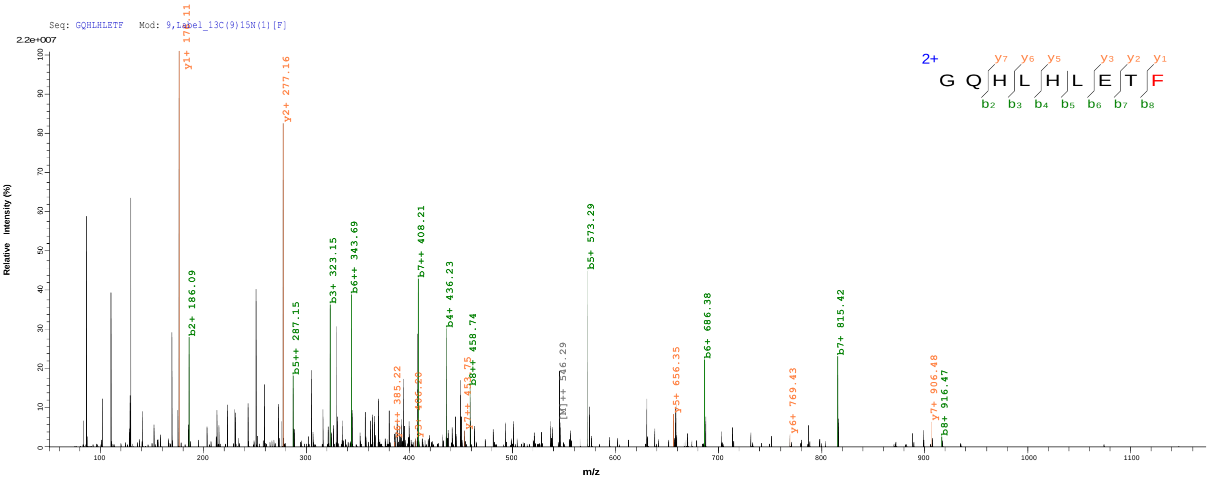

Seq: GQHLHLETF Mod: 9,Label_13C(9)15N(1)[F]
2.2e+007
Relative Intensity (%)
m/z
2+
G Q H L H L E T F
y1+ 176.11
y2+ 277.16
b2+ 186.09
b5++ 287.15
b3+ 323.15
b6++ 343.69
b7++ 408.21
b4+ 436.23
b8++ 458.74
[M]++ 546.29
b5+ 573.29
y5+ 656.35
b6+ 686.38
y6+ 769.43
b7+ 815.42
y7+ 906.48
b8+ 916.47

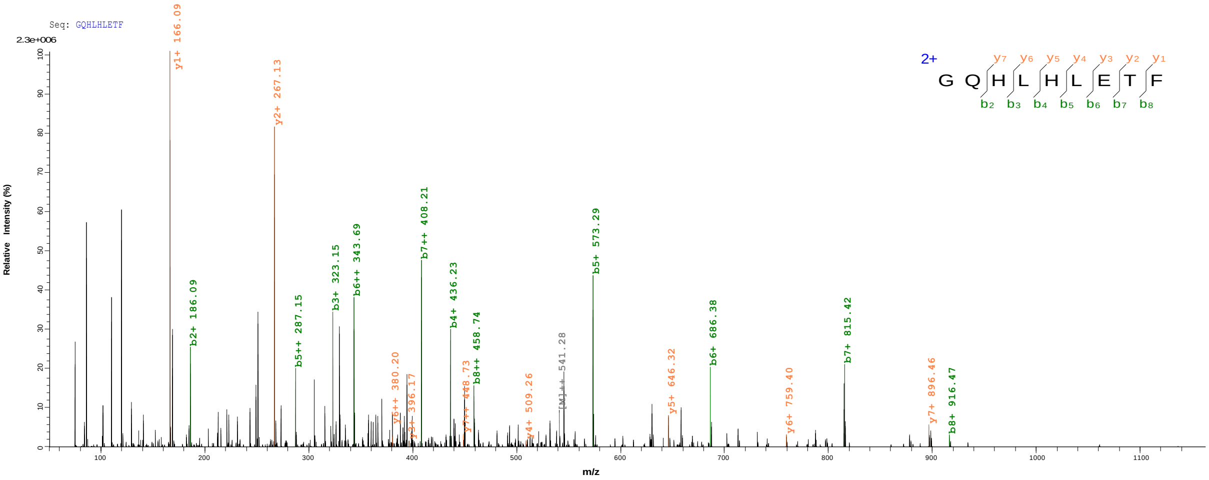

Seq: GQHLHLETF
2.3e+006
Relative Intensity (%)
m/z
2+
G Q H L H L E T F
y1+ 166.09
y2+ 267.13
b2+ 186.09
b5++ 287.15
b3+ 323.15
b6++ 343.69
y6++ 380.20
b7++ 408.21
b4+ 436.23
b8++ 458.74
y4+ 509.26
[M]++ 541.28
b5+ 573.29
y5+ 646.32
b6+ 686.38
y6+ 759.40
b7+ 815.42
y7+ 896.46
b8+ 916.47

SPAQRDRVVM

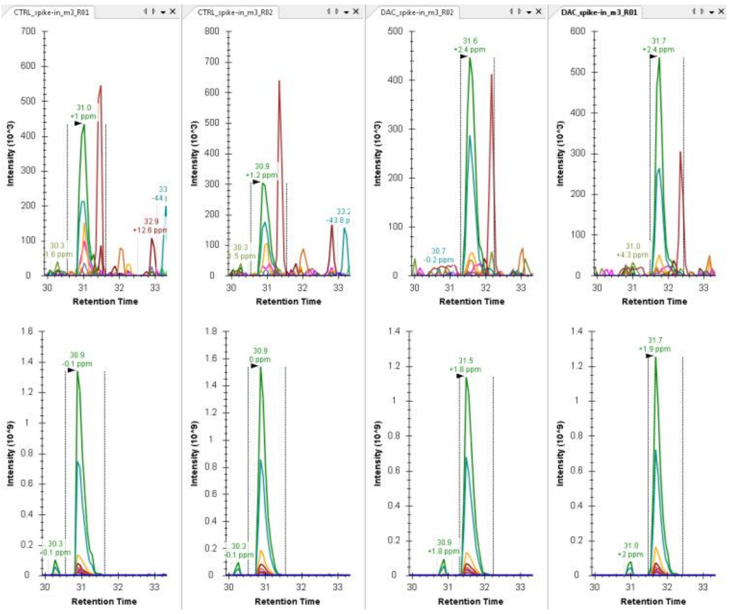
CTRL_spike-in_m3_R01
CTRL_spike-in_m3_R02
DAC_spike-in_m3_R02
DAC_spike-in_m3_R01
Intensity (10^3)
Intensity (10^9)
Retention Time

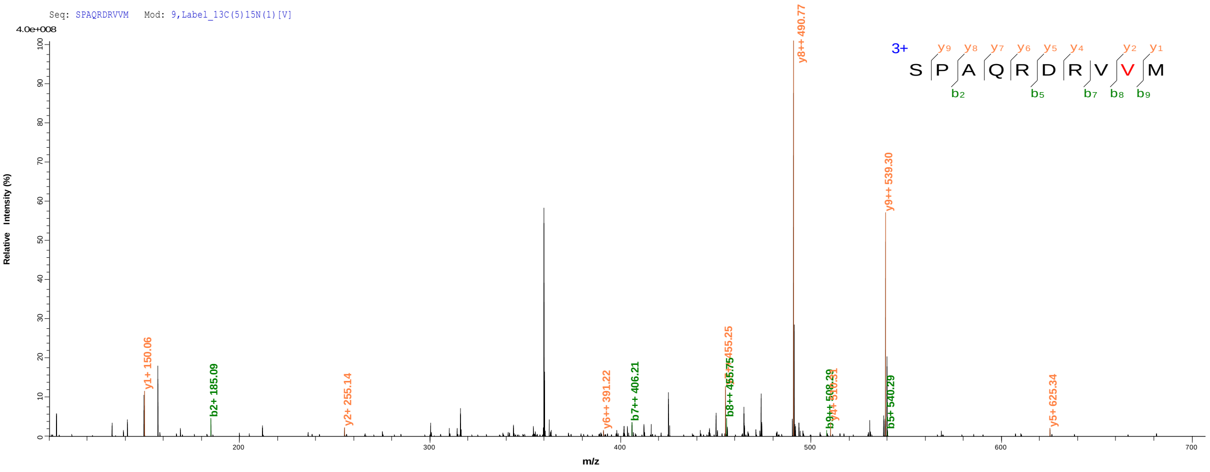
Seq: SPAQRDRVVM Mod: 9,Label_13C(5)15N(1)[V]
4.0e+008
Relative Intensity (%)
m/z
y8++ 490.77
y9++ 539.30
y1+ 150.06
b2+ 185.09
y2+ 255.14
y6++ 391.22
b7++ 406.21
b5+ 540.29
y5+ 625.34
3+
S P A Q R D R V V M

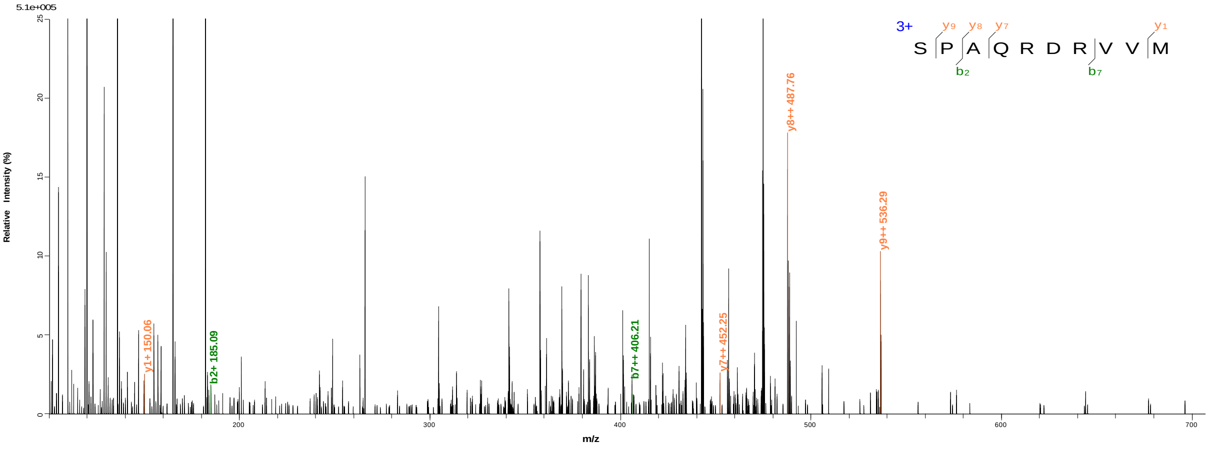
5.1e+005
Relative Intensity (%)
m/z
y8++ 487.76
y9++ 536.29
y1+ 150.06
b2+ 185.09
b7++ 406.21
y7++ 452.25
3+
S P A Q R D R V V M

AAEPDRAVL

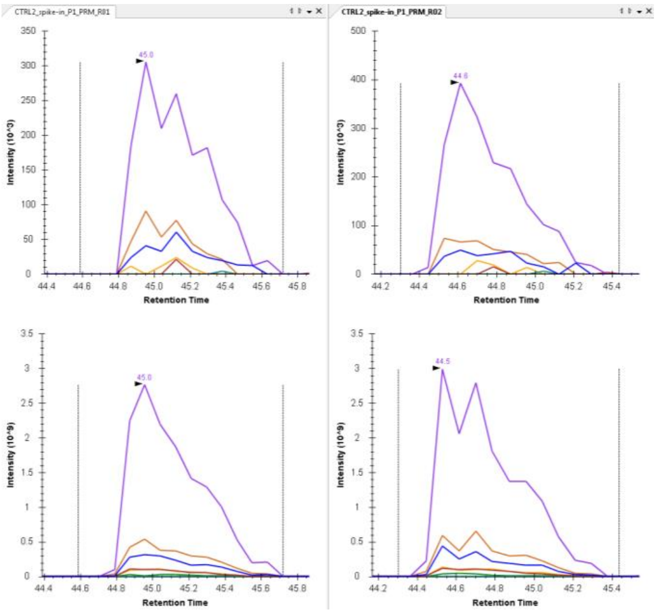

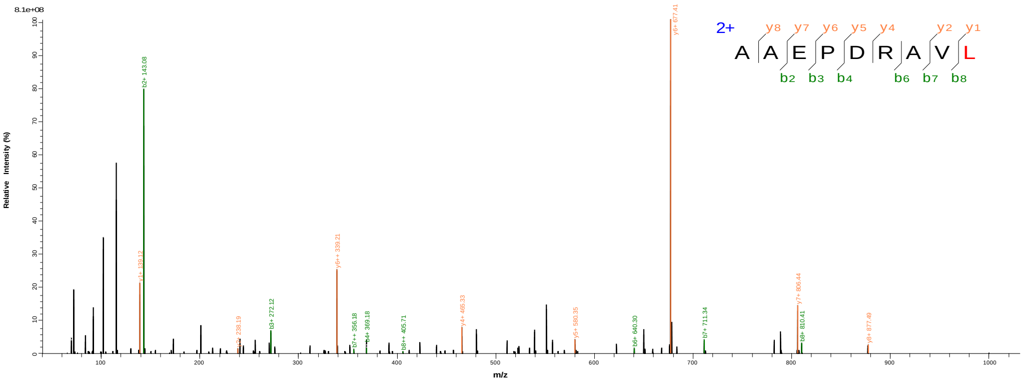
2+
A A E P D R A V L
y8 y7 y6 y5 y4 y2 y1
b2 b3 b4 b6 b7 b8
m/z
Relative Intensity (%)

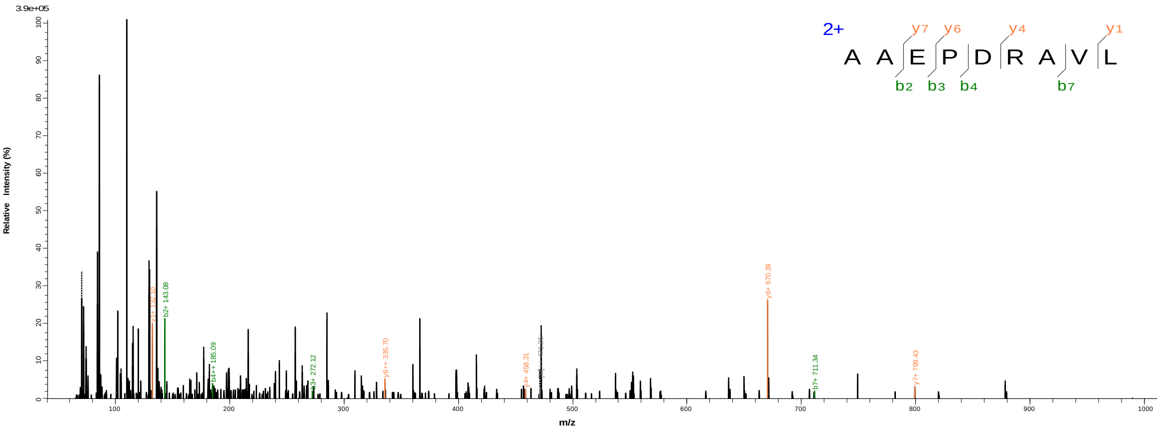
2+
A A E P D R A V L
y7 y6 y4 y1
b2 b3 b4 b7
m/z
Relative Intensity (%)

RQFLEPSGF

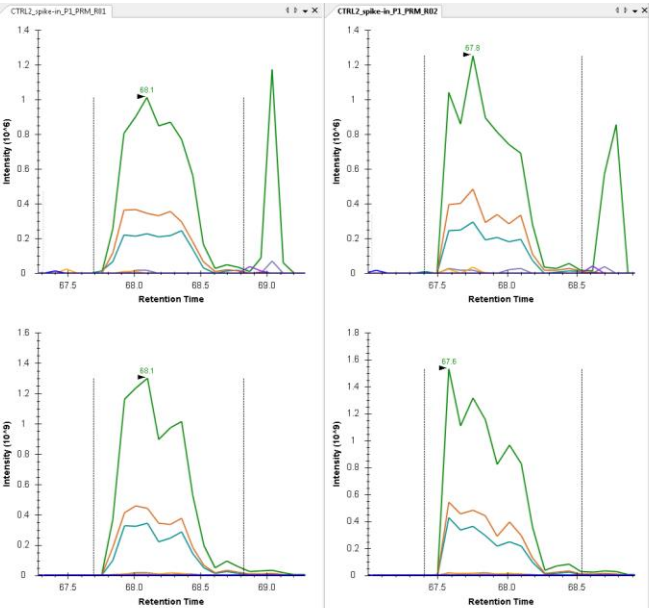
CTRL2_spike-in_P1_PRM_R01
CTRL2_spike-in_P1_PRM_R02
Intensity (10^6)
Intensity (10^9)
Retention Time
68.1
67.8
67.6

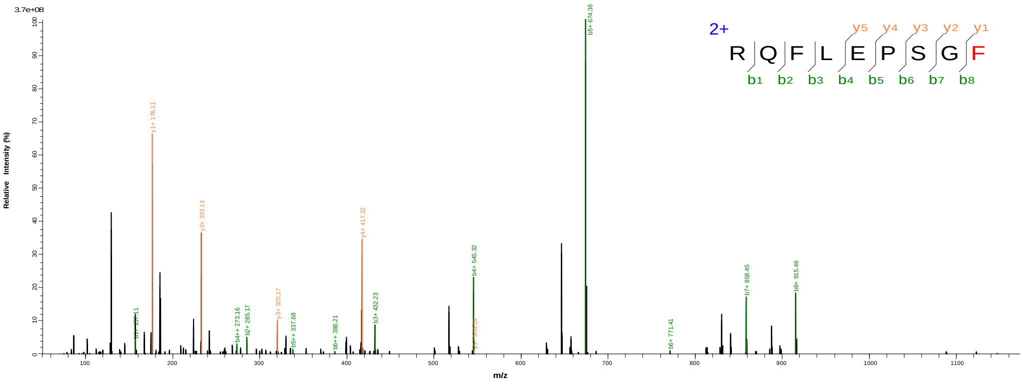
3.7e+08
Relative Intensity (%)
m/z
2+
R Q F L E P S G F
y5 y4 y3 y2 y1
b1 b2 b3 b4 b5 b6 b7 b8
b5+ 674.36
y1+ 176.11
y2+ 233.13
y4+ 417.22
b4+ 545.32
b7+ 858.45
b8+ 915.46

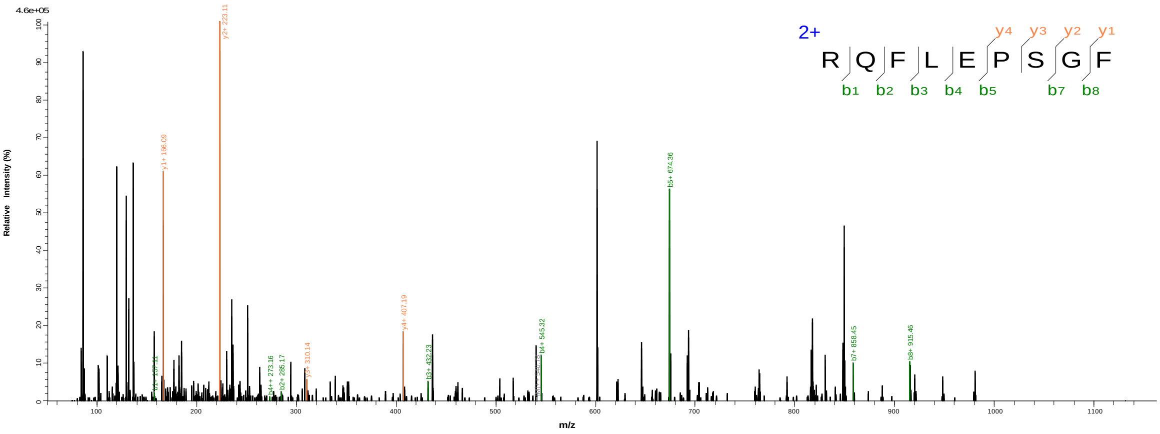
4.6e+05
Relative Intensity (%)
m/z
2+
R Q F L E P S G F
y4 y3 y2 y1
b1 b2 b3 b4 b5 b7 b8
y2+ 223.11
y1+ 166.09
b5+ 674.36
y4+ 407.19
b4+ 545.32
b7+ 858.45
b8+ 915.46

TPNSLAKPL

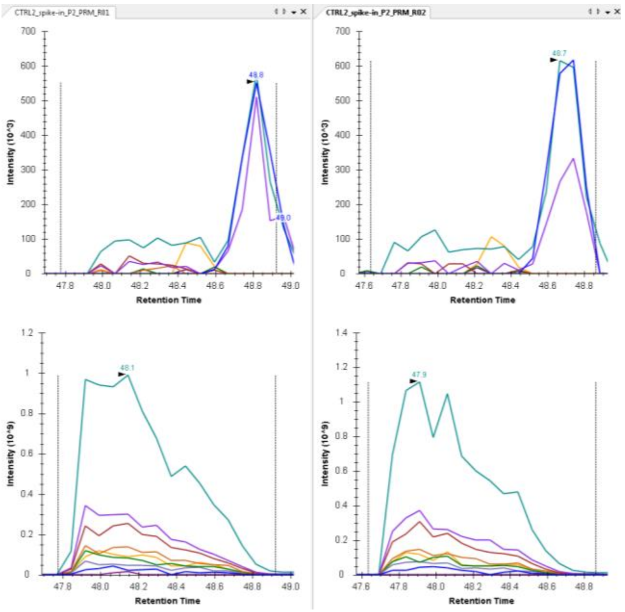
Intensity (10^3)
Retention Time
Intensity (10^9)
Retention Time

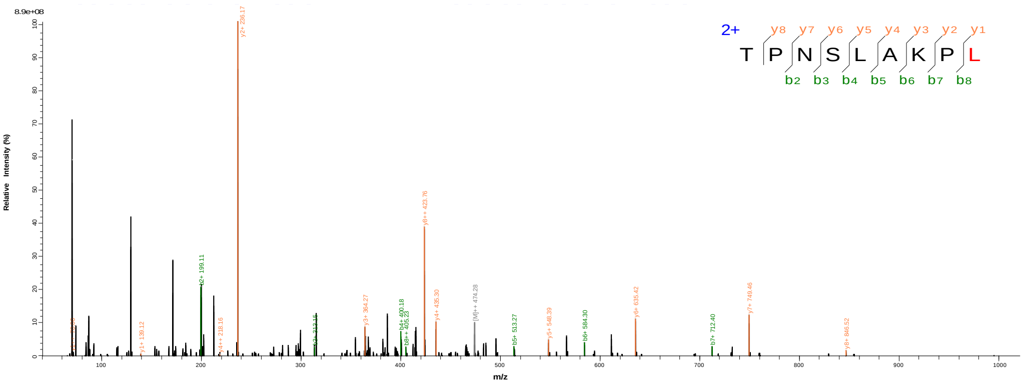
8.9e+08
Relative Intensity (%)
m/z
2+
y8 y7 y6 y5 y4 y3 y2 y1
T P N S L A K P L
b2 b3 b4 b5 b6 b7 b8

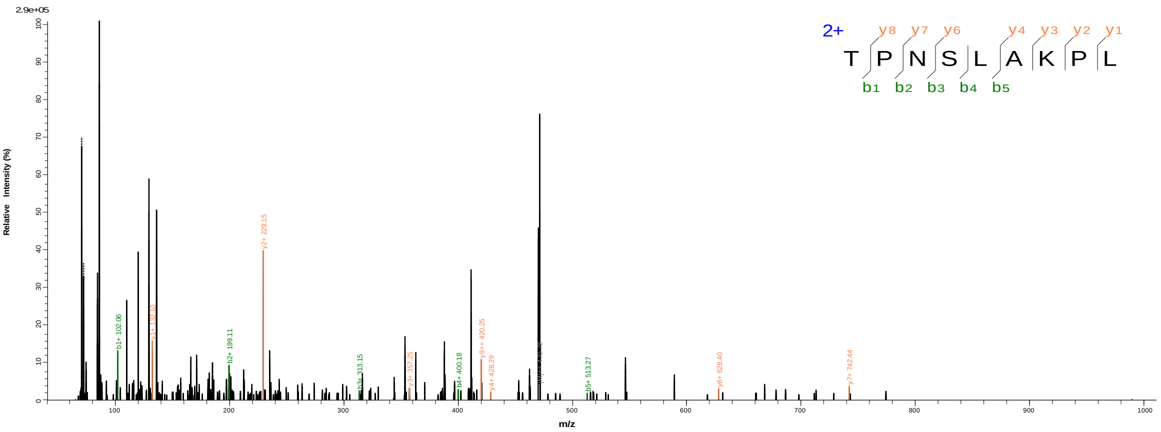
2.9e+05
Relative Intensity (%)
m/z
2+
y8 y7 y6 y4 y3 y2 y1
T P N S L A K P L
b1 b2 b3 b4 b5

KSDLSKPLSY

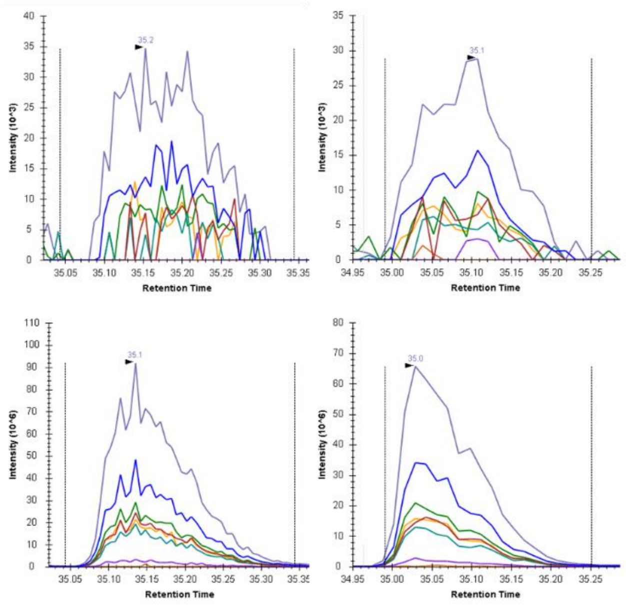
Intensity (10^3)
Intensity (10^6)
Retention Time

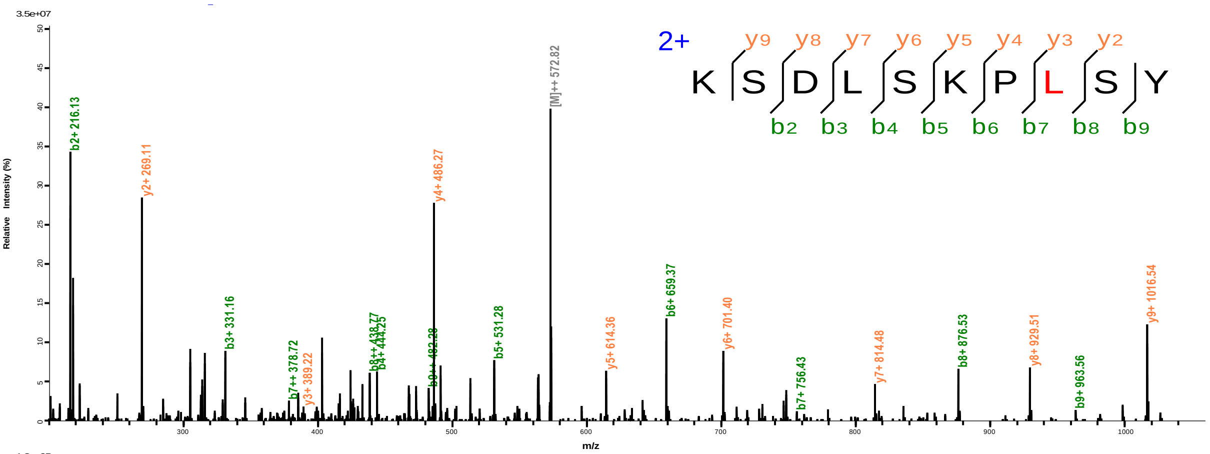
3.5e+07
Relative Intensity (%)
m/z
2+
y9 y8 y7 y6 y5 y4 y3 y2
K S D L S K P L S Y
b2 b3 b4 b5 b6 b7 b8 b9
b2+ 216.13
y2+ 269.11
b3+ 331.16
b7++ 378.72
y3+ 389.22
y4+ 486.27
[M]++ 572.82
b5+ 531.28
y5+ 614.36
b6+ 659.37
y6+ 701.40
b7+ 756.43
y7+ 814.48
b8+ 876.53
y8+ 929.51
b9+ 963.56
y9+ 1016.54

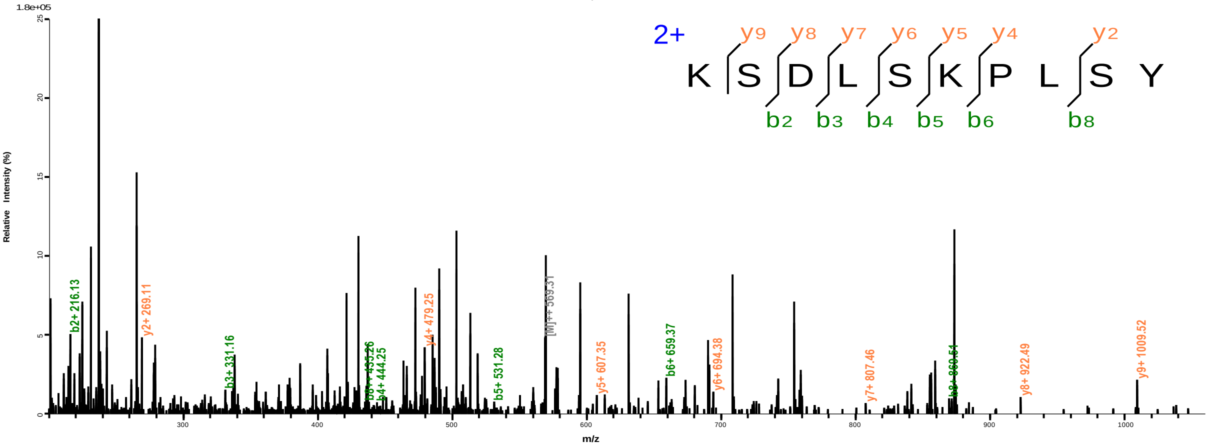
1.8e+05
Relative Intensity (%)
m/z
2+
y9 y8 y7 y6 y5 y4 y2
K S D L S K P L S Y
b2 b3 b4 b5 b6 b8
b2+ 216.13
y2+ 269.11
b3+ 331.16
b4+ 444.25
y4+ 479.25
b5+ 531.28
y5+ 607.35
b6+ 659.37
y6+ 694.38
y7+ 807.46
y8+ 922.49
y9+ 1009.52

NPRNDGIAL

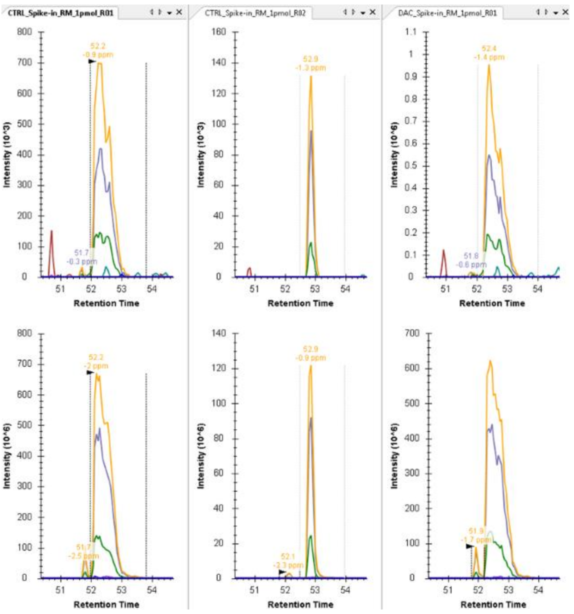
CTRL_Spike-in_RM_1pmol_R01
CTRL_Spike-in_RM_1pmol_R02
DAC_Spike-in_RM_1pmol_R01
Intensity (10^3)
Intensity (10^6)
Retention Time

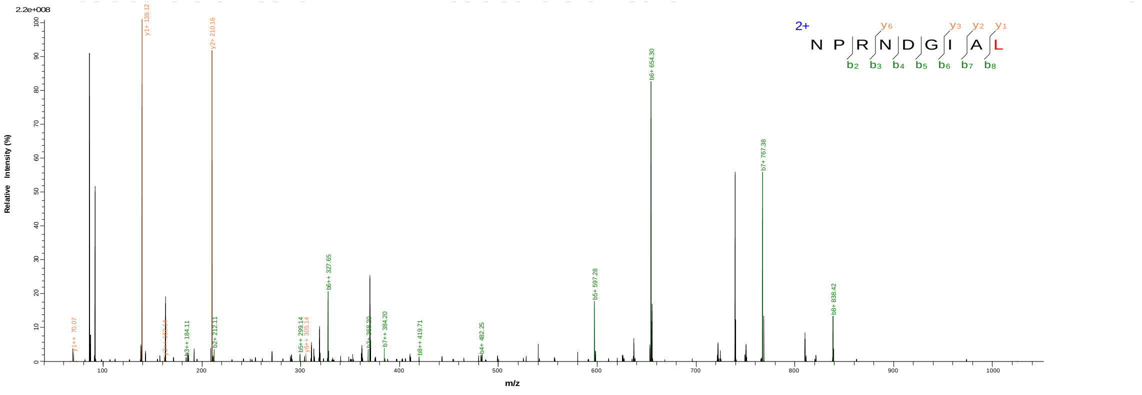
2.2e+008
Relative Intensity (%)
m/z
2+
N P R N D G I A L
b6+ 654.30
b7+ 767.38
b8+ 838.42
b5+ 597.28
b6++ 327.65
y1+ 132.10
y2+ 203.14

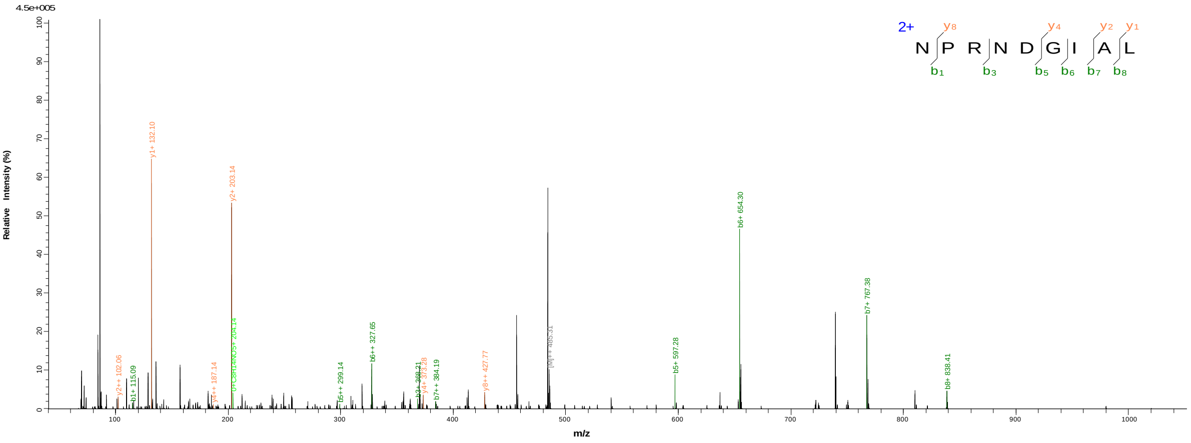
4.5e+005
Relative Intensity (%)
m/z
2+
N P R N D G I A L
b6+ 654.30
b7+ 767.38
b8+ 838.41
b5+ 597.28
b6++ 327.65
y1+ 132.10
y2+ 203.14

DVDIHKDLY

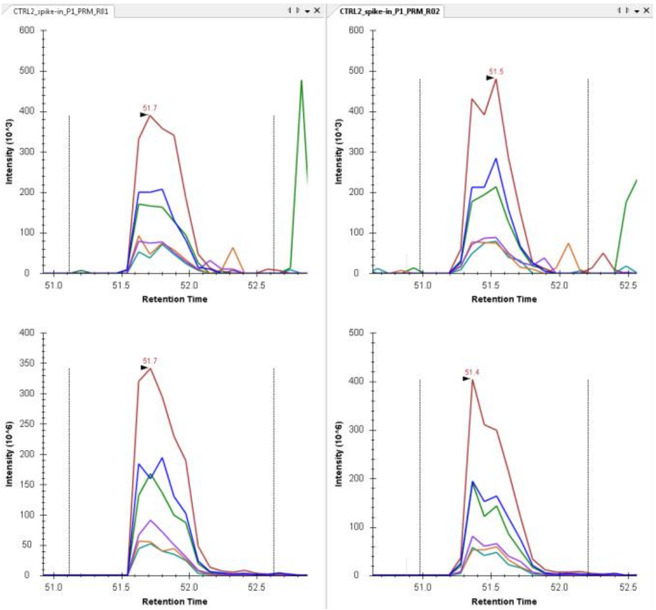
CTRL2_spike-in_P1_PRM_R01
CTRL2_spike-in_P1_PRM_R02
Intensity (10^3)
Intensity (10^6)
Retention Time
51.7
51.5
51.7
51.4

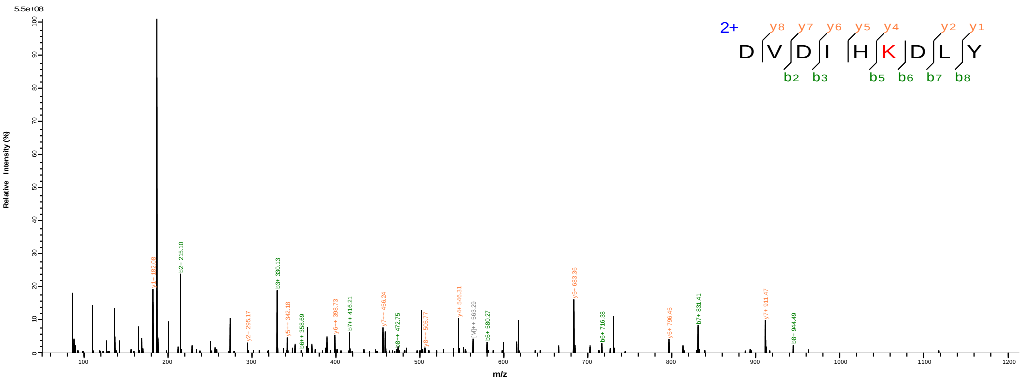
5.5e+08
Relative Intensity (%)
m/z
2+
D V D I H K D L Y
y8 y7 y6 y5 y4 y2 y1
b2 b3 b5 b6 b7 b8

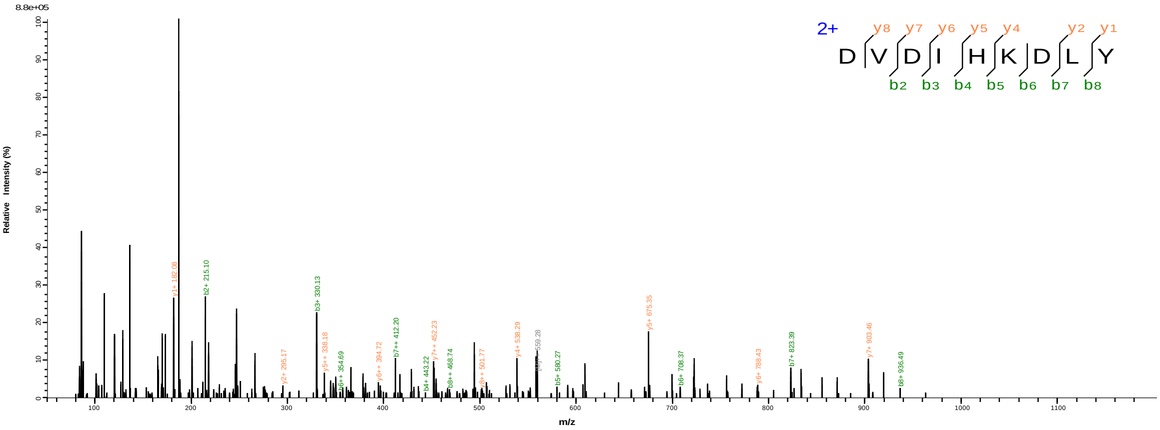
8.8e+05
Relative Intensity (%)
m/z
2+
D V D I H K D L Y
y8 y7 y6 y5 y4 y2 y1
b2 b3 b4 b5 b6 b7 b8

RIHDSRSSL

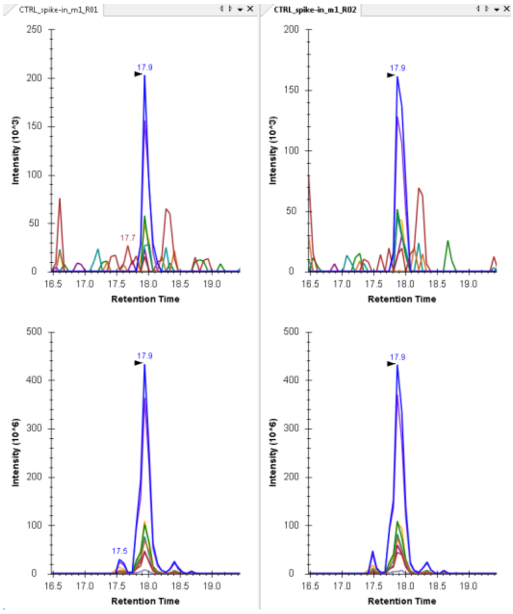

CTRL_spike-in_m1_R01
CTRL_spike-in_m1_R02
Intensity (10^3)
Intensity (10^6)
Retention Time
17.9
17.7
17.5

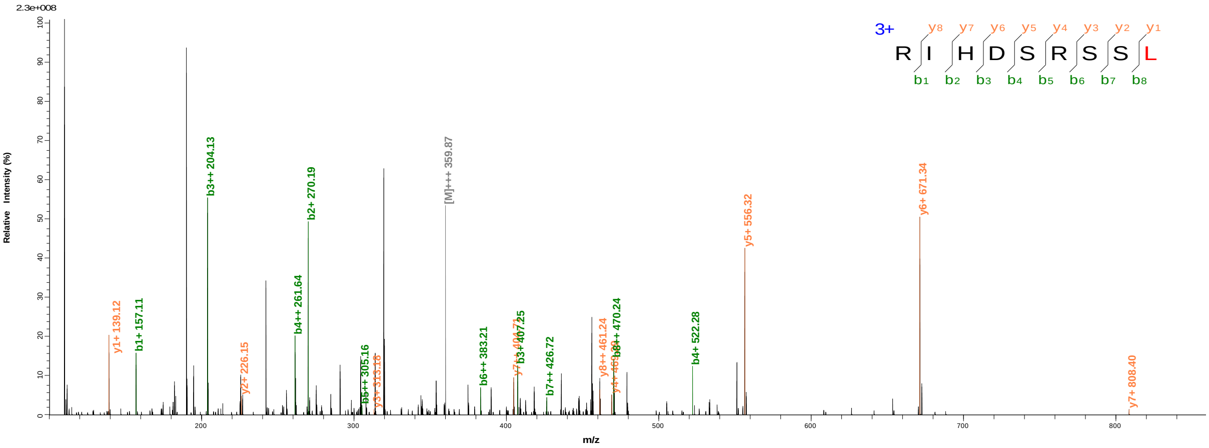

2.3e+008
Relative Intensity (%)
m/z
3+
R I H D S R S S L
y8 y7 y6 y5 y4 y3 y2 y1
b1 b2 b3 b4 b5 b6 b7 b8
y1+ 139.12
b1+ 157.11
b3++ 204.13
y2+ 226.15
b4++ 261.64
b2+ 270.19
[M]+++ 359.87
b6++ 383.21
b3+ 407.25
b7++ 426.72
y8++ 461.24
b8++ 470.24
b4+ 522.28
y5+ 556.32
y6+ 671.34
y7+ 808.40

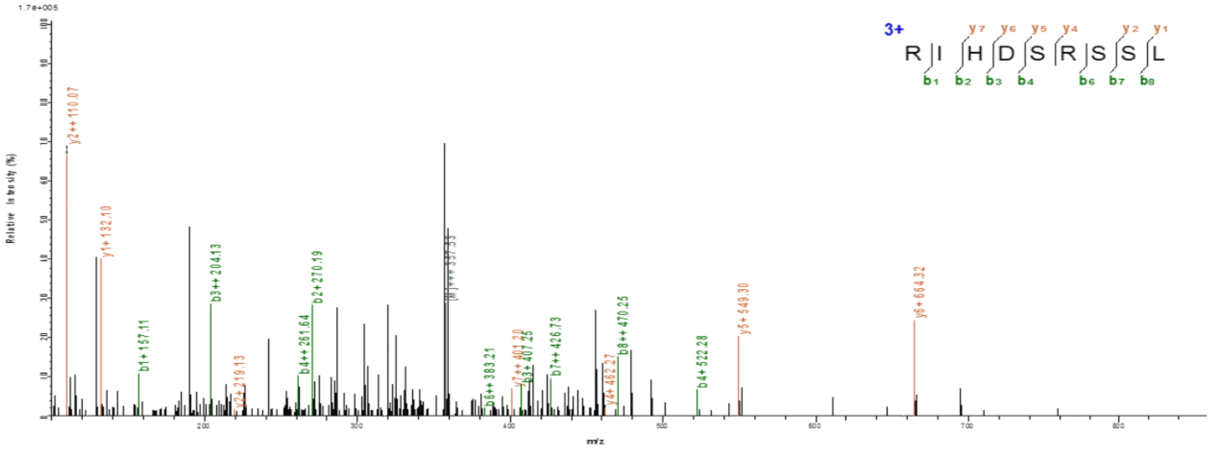

1.7e+005
Relative Intensity (%)
m/z
3+
R I H D S R S S L
y7 y6 y5 y4 y2 y1
b1 b2 b3 b4 b6 b7 b8
y2++ 110.07
y1+ 132.10
b1+ 157.11
b3++ 204.13
b4++ 261.64
b2+ 270.19
b6++ 383.21
b3+ 407.25
b7++ 426.73
b8++ 470.25
b4+ 522.28
y5+ 549.30
y6+ 664.32

ASKGAGVSF

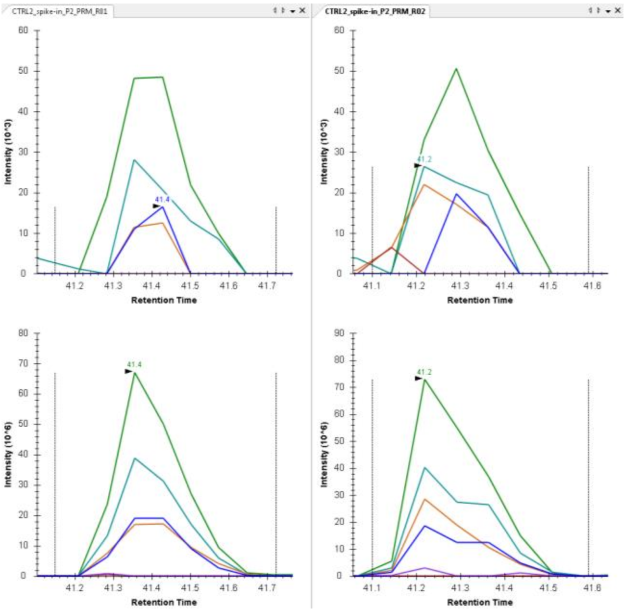
CTRL2_spike-in_P2_PRM_R01
CTRL2_spike-in_P2_PRM_R02
Intensity (10^3)
Intensity (10^6)
Retention Time

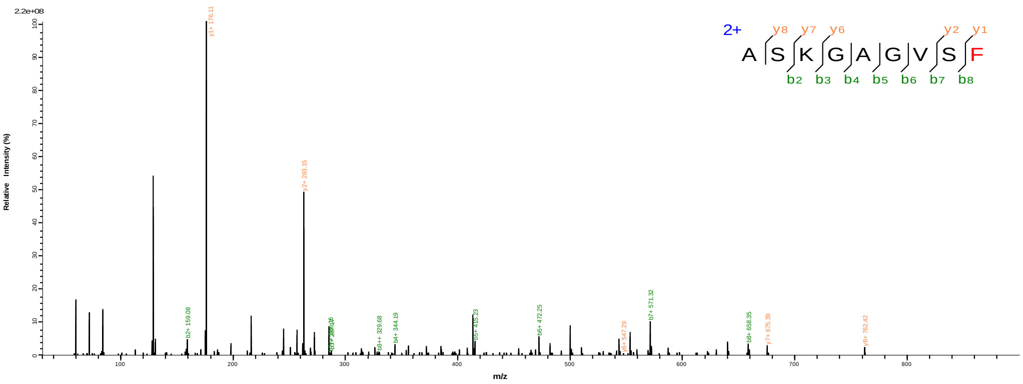
2.2e+08
Relative Intensity (%)
m/z
2+
y8 y7 y6 y2 y1
A S K G A G V S F
b2 b3 b4 b5 b6 b7 b8

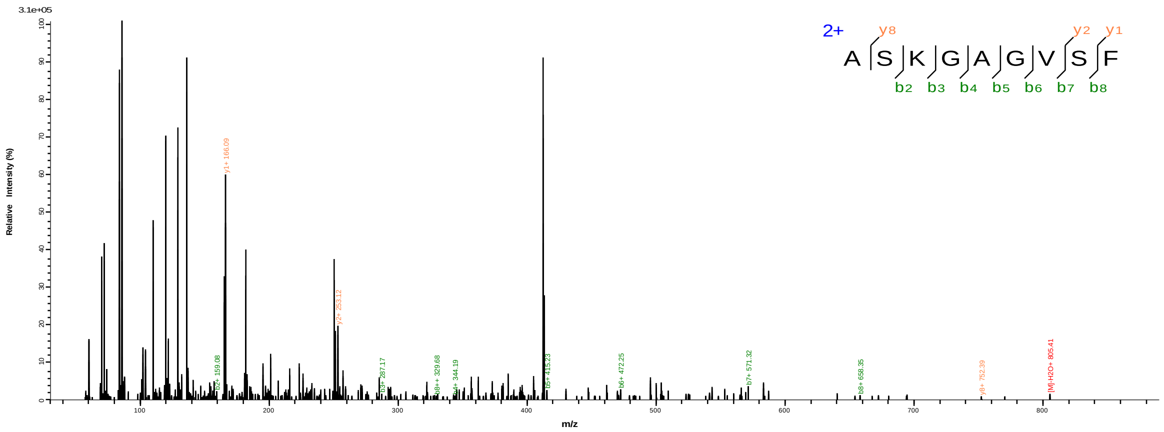
3.1e+05
Relative Intensity (%)
m/z
2+
y8 y2 y1
A S K G A G V S F
b2 b3 b4 b5 b6 b7 b8

KMEETTVTY

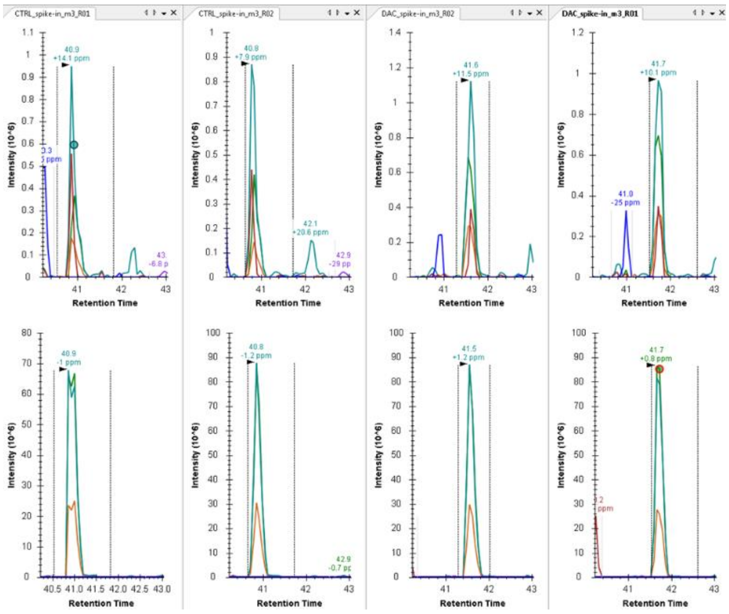

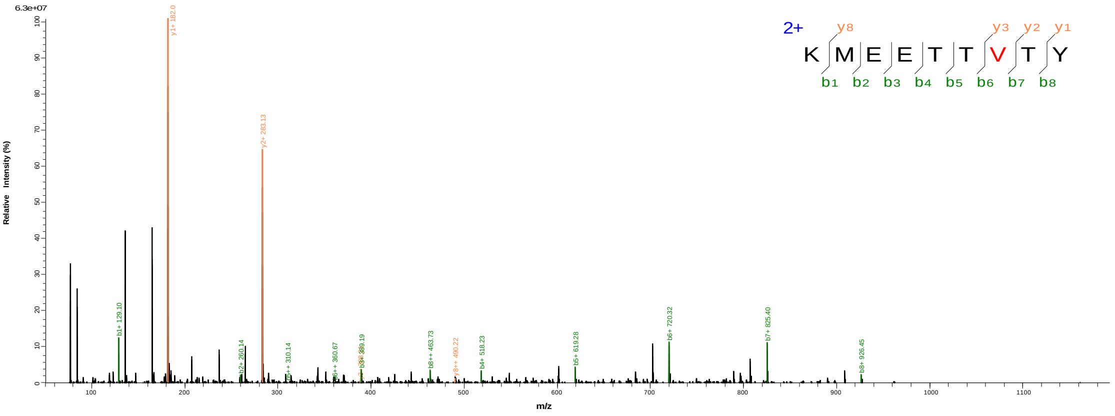

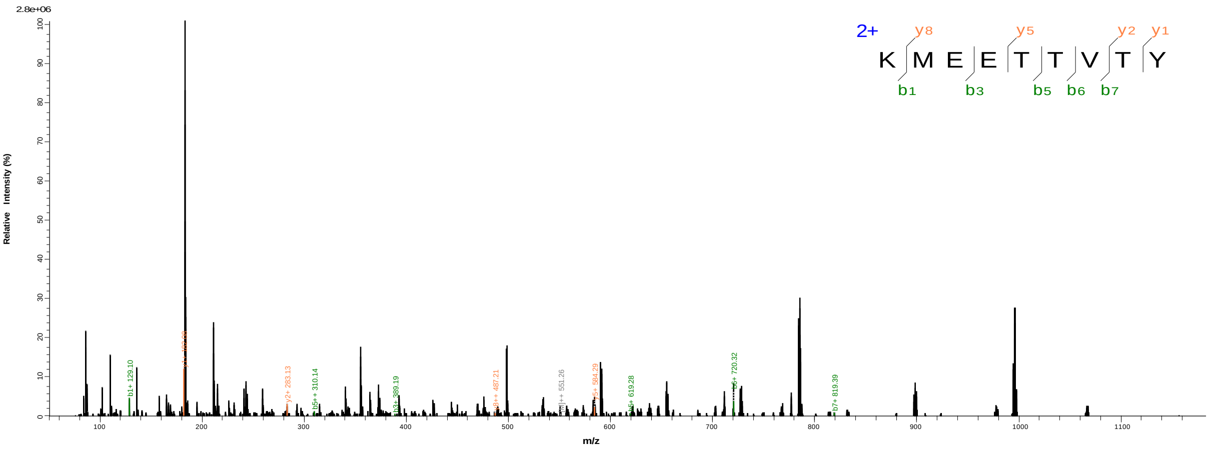

EYNNIFHTF

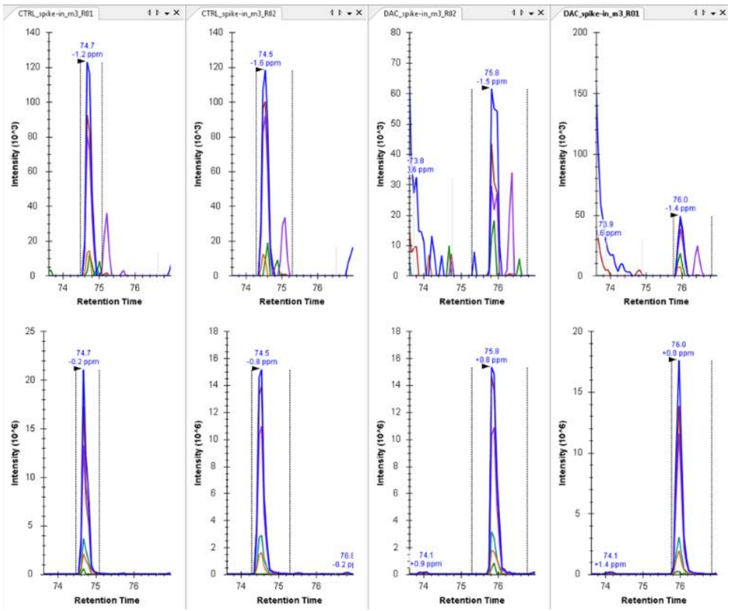
CTRL_spike-in_m3_R01
CTRL_spike-in_m3_R02
DAC_spike-in_m3_R02
DAC_spike-in_m3_R01
Intensity (10^3)
Intensity (10^6)
Retention Time

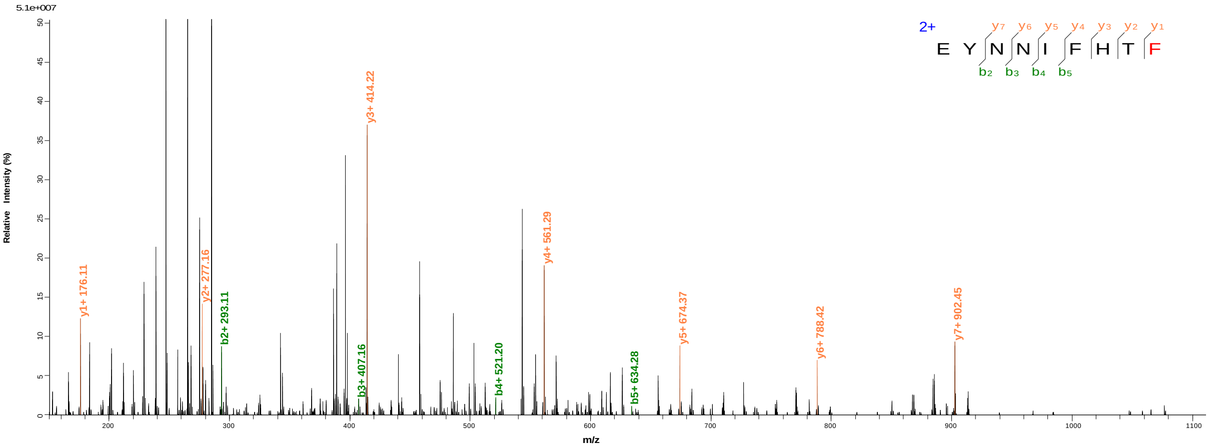
5.1e+007
2+
E Y N N I F H T F
y7 y6 y5 y4 y3 y2 y1
b2 b3 b4 b5
y1+ 176.11
y2+ 277.16
b2+ 293.11
b3+ 407.16
y3+ 414.22
b4+ 521.20
y4+ 561.29
b5+ 634.28
y5+ 674.37
y6+ 788.42
y7+ 902.45
Relative Intensity (%)
m/z

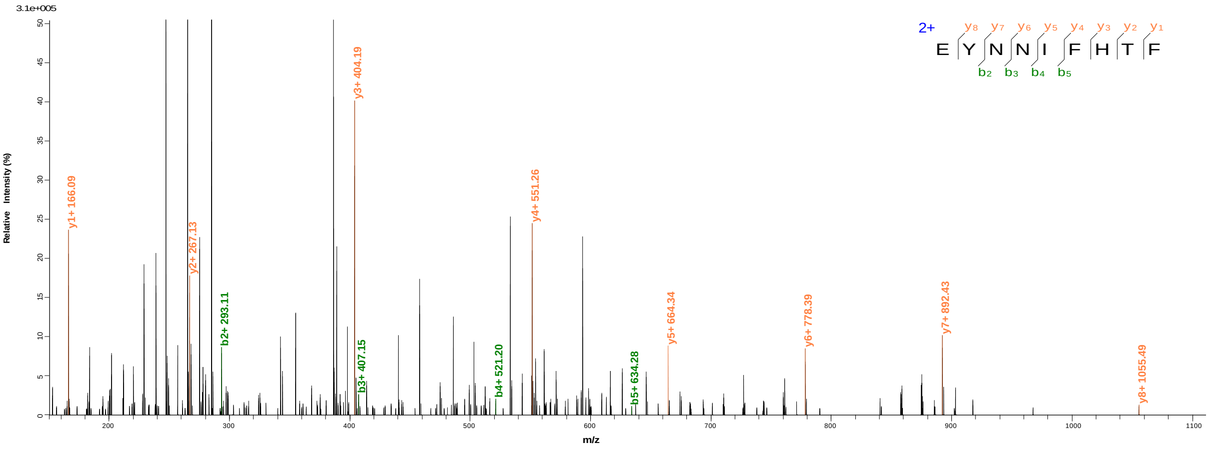
3.1e+005
2+
E Y N N I F H T F
y8 y7 y6 y5 y4 y3 y2 y1
b2 b3 b4 b5
y1+ 166.09
y2+ 267.13
b2+ 293.11
y3+ 404.19
b3+ 407.15
b4+ 521.20
y4+ 551.26
b5+ 634.28
y5+ 664.34
y6+ 778.39
y7+ 892.43
y8+ 1055.49
Relative Intensity (%)
m/z

ETDIEMETRY

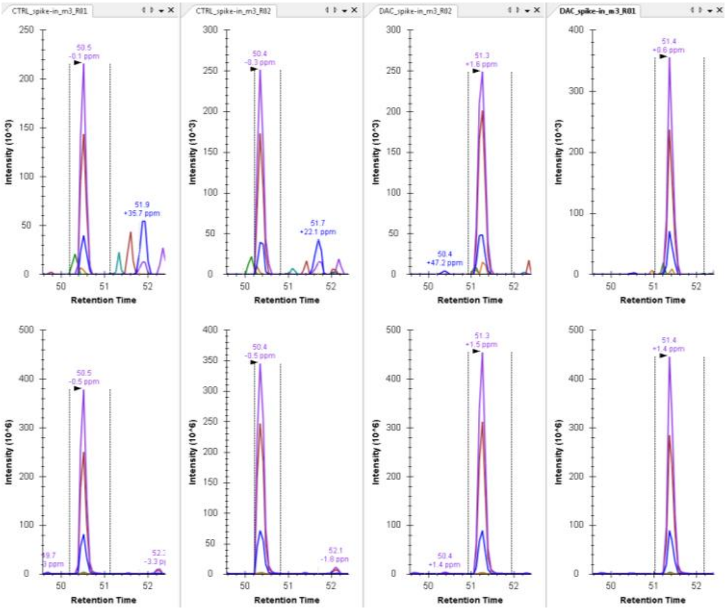

CTRL_spike-in_m3_R01
CTRL_spike-in_m3_R02
DAC_spike-in_m3_R02
DAC_spike-in_m3_R01
Intensity (10^3)
Intensity (10^6)
Retention Time

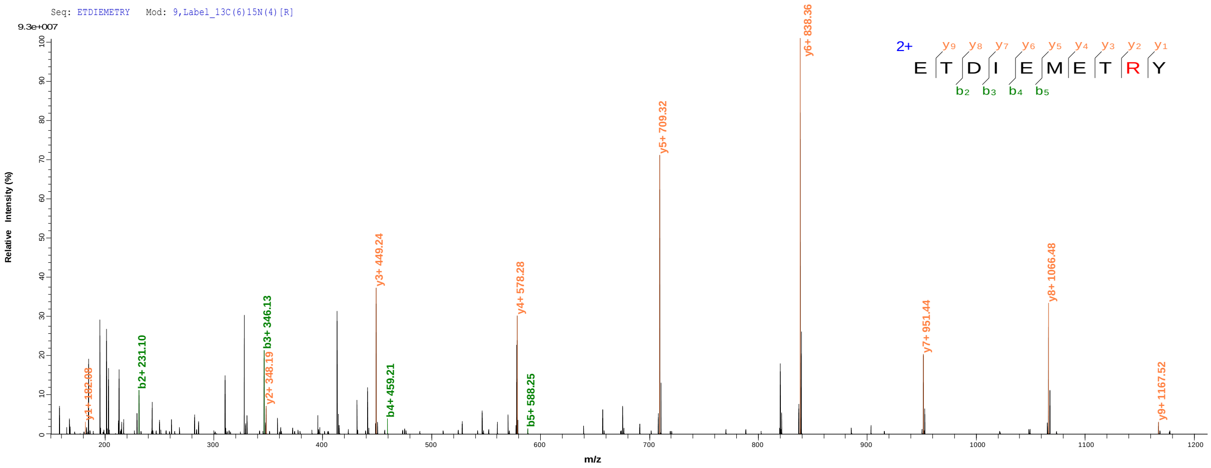

Seq: ETDIEMETRY Mod: 9,Label_13C(6)15N(4)[R]
9.3e+007
Relative Intensity (%)
m/z
y6+ 838.36
y5+ 709.32
y3+ 449.24
y4+ 578.28
y8+ 1066.48
y7+ 951.44
y9+ 1167.52
b3+ 346.13
b2+ 231.10
y2+ 348.19
b4+ 459.21
b5+ 588.25
2+
E T D I E M E T R Y

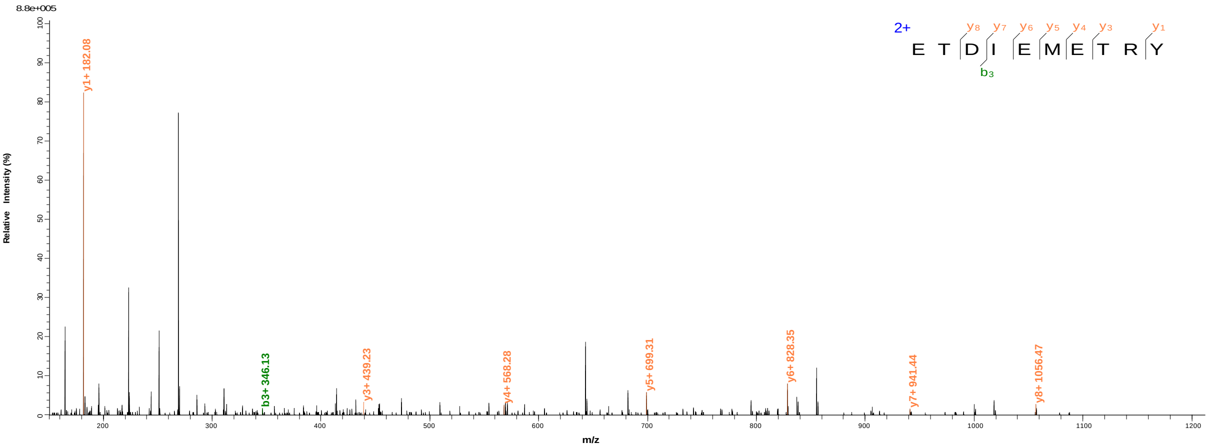

8.8e+005
Relative Intensity (%)
m/z
y1+ 182.08
b3+ 346.13
y3+ 439.23
y4+ 568.28
y5+ 699.31
y6+ 828.35
y7+ 941.44
y8+ 1056.47
2+
E T D I E M E T R Y

YLDPAQQNLY

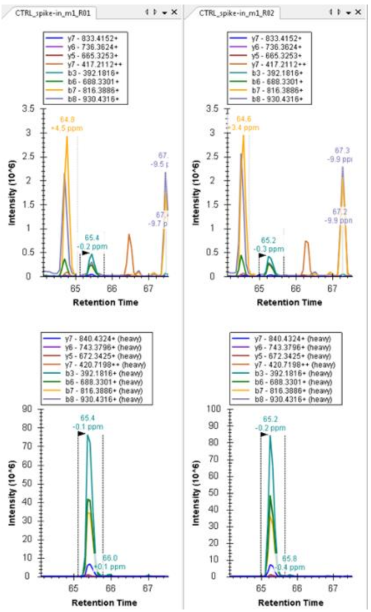

CTRL_spike-in_m1_R01
CTRL_spike-in_m1_R02
y7 - 833.4152+
y6 - 736.3624+
y5 - 665.3253+
y7 - 417.2112++
b3 - 392.1816+
b6 - 688.3301+
b7 - 816.3886+
b8 - 930.4316+
Intensity (10^6)
Retention Time
y7 - 840.4324+ (heavy)
y6 - 743.3796+ (heavy)
y5 - 672.3425+ (heavy)
y7 - 420.7198++ (heavy)
b3 - 392.1816+ (heavy)
b6 - 688.3301+ (heavy)
b7 - 816.3886+ (heavy)
b8 - 930.4316+ (heavy)

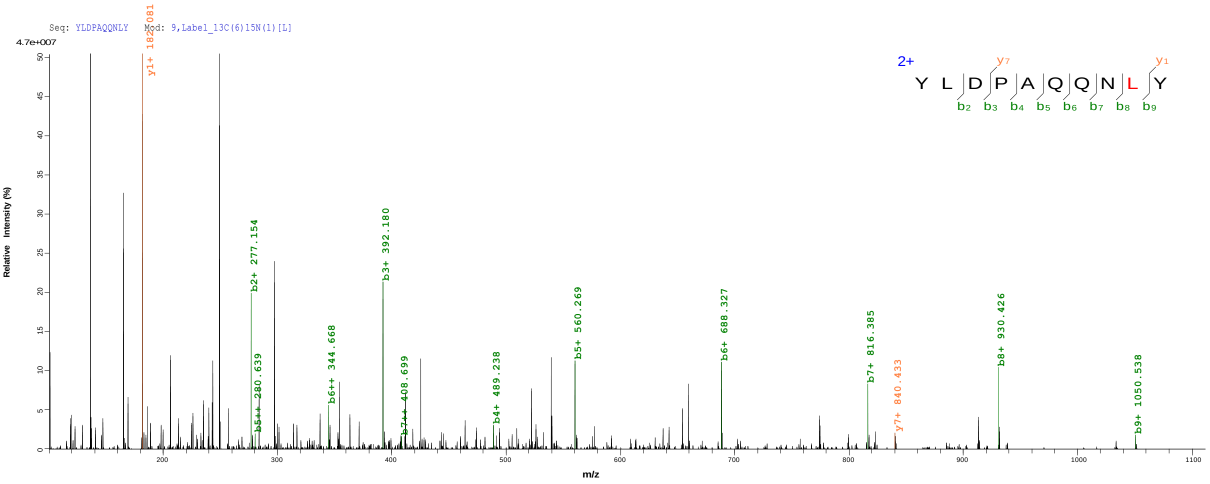

Seq: YLDPAQQNLY Mod: 9,Label_13C(6)15N(1)[L]
4.7e+007
2+
Y L D P A Q Q N L Y
y1+ 182.081
b2+ 277.154
b5++ 280.639
b6++ 344.668
b3+ 392.180
b7++ 408.699
b4+ 489.238
b5+ 560.269
b6+ 688.327
b7+ 816.385
y7+ 840.433
b8+ 930.426
b9+ 1050.538
Relative Intensity (%)
m/z

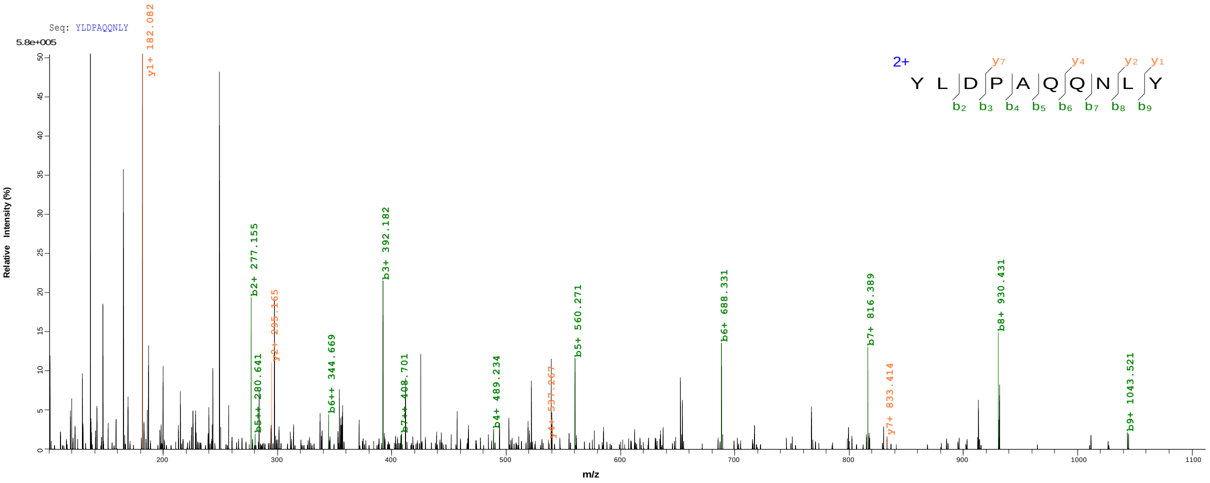

Seq: YLDPAQQNLY
5.8e+005
2+
Y L D P A Q Q N L Y
y1+ 182.082
b2+ 277.155
b5++ 280.641
y2+ 295.165
b6++ 344.669
b3+ 392.182
b7++ 408.701
b4+ 489.234
y4+ 537.267
b5+ 560.271
b6+ 688.331
b7+ 816.389
y7+ 833.414
b8+ 930.431
b9+ 1043.521
Relative Intensity (%)
m/z

VPHQPAPPF

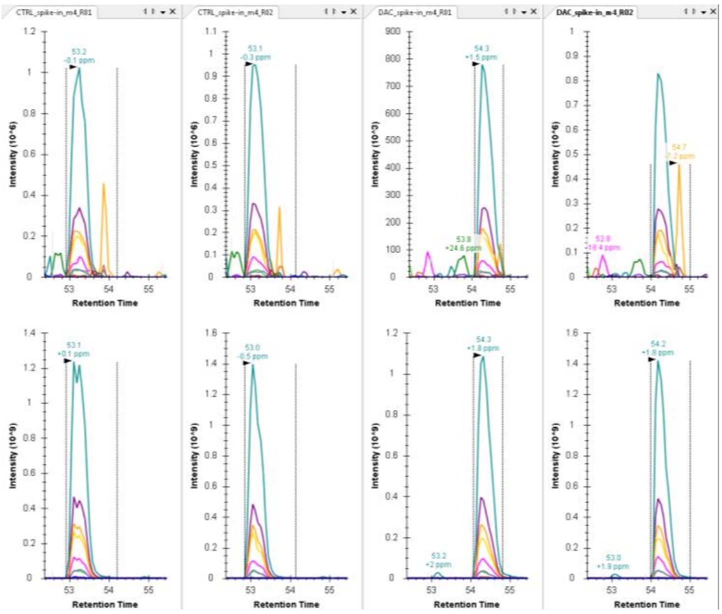

Intensity (10^6)
Intensity (10^9)
Retention Time

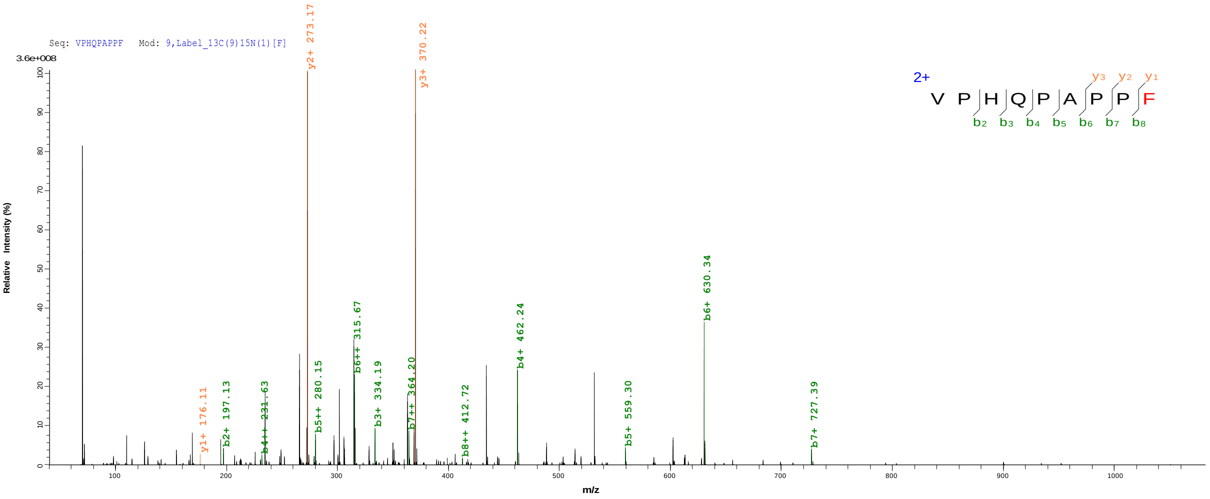

Seq: VPHQPAPPF Mod: 9,Label_13C(9)15N(1)[F]
3.6e+008
Relative Intensity (%)
m/z
y2+ 273.17
y3+ 370.22
y1+ 176.11
b2+ 197.13
b4++ 231.63
b5++ 280.15
b6++ 315.67
b3+ 334.19
b7++ 364.20
b8++ 412.72
b4+ 462.24
b5+ 559.30
b6+ 630.34
b7+ 727.39
2+
V P H Q P A P P F

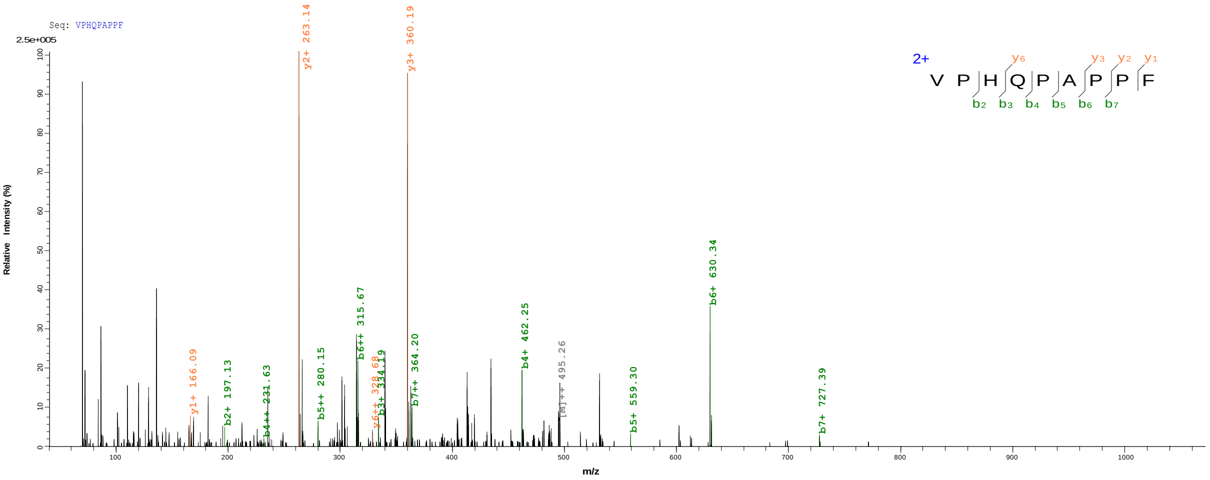

Seq: VPHQPAPPF
2.5e+005
Relative Intensity (%)
m/z
y2+ 263.14
y3+ 360.19
y1+ 166.09
b2+ 197.13
b4++ 231.63
b5++ 280.15
b6++ 315.67
b7++ 364.20
b4+ 462.25
495.26
b5+ 559.30
b6+ 630.34
b7+ 727.39
2+
V P H Q P A P P F

TFQKKTKEM

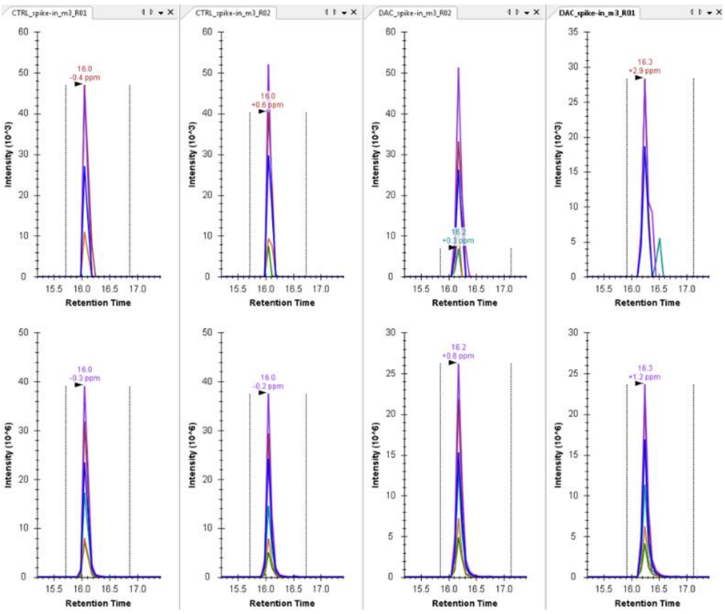

CTRL_spike-in_m3_R01
CTRL_spike-in_m3_R02
DAC_spike-in_m3_R02
DAC_spike-in_m3_R01
Intensity (10^3)
Intensity (10^6)
Retention Time

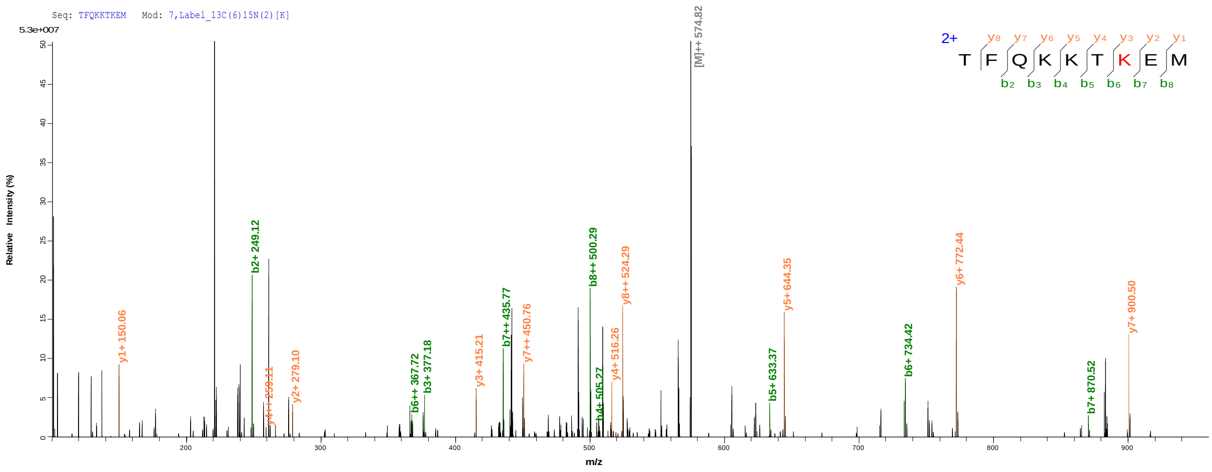

Seq: TFQKKTKEM Mod: 7,Label_13C(6)15N(2)[K]
5.3e+007
Relative Intensity (%)
m/z
[M]++ 574.82
2+
T F Q K K T K E M
y1+ 150.06
b2+ 249.12
y2+ 279.10
b6++ 367.72
b3+ 377.18
y3+ 415.21
b7++ 435.77
y7++ 450.76
b8++ 500.29
b4+ 505.27
y4+ 516.26
y8++ 524.29
b5+ 633.37
y5+ 644.35
b6+ 734.42
y6+ 772.44
b7+ 870.52
y7+ 900.50

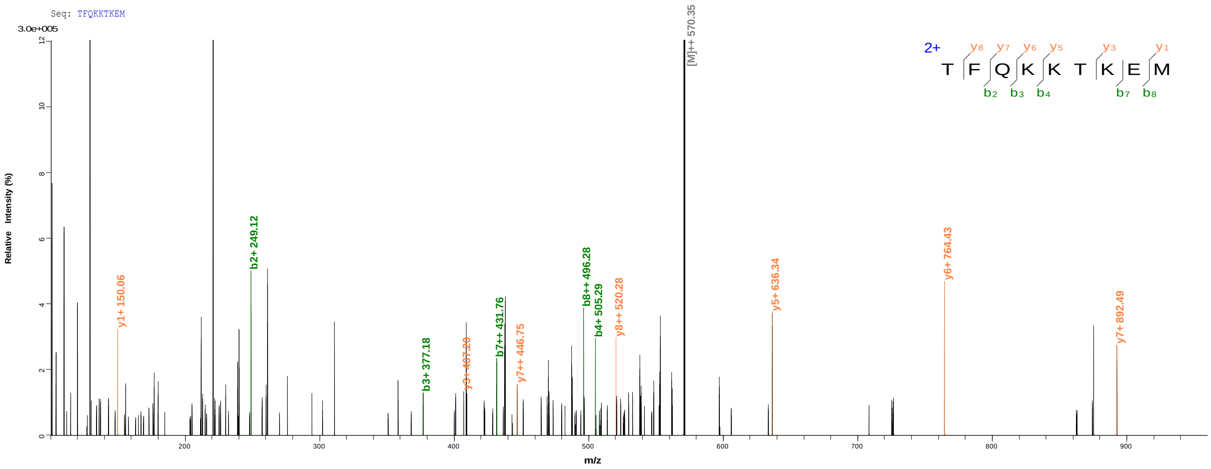

Seq: TFQKKTKEM
3.0e+005
Relative Intensity (%)
m/z
[M]++ 570.35
2+
T F Q K K T K E M
y1+ 150.06
b2+ 249.12
b3+ 377.18
b7++ 431.76
y7++ 446.75
b8++ 496.28
b4+ 505.29
y8++ 520.28
y5+ 636.34
y6+ 764.43
y7+ 892.49

QTEKENSKY

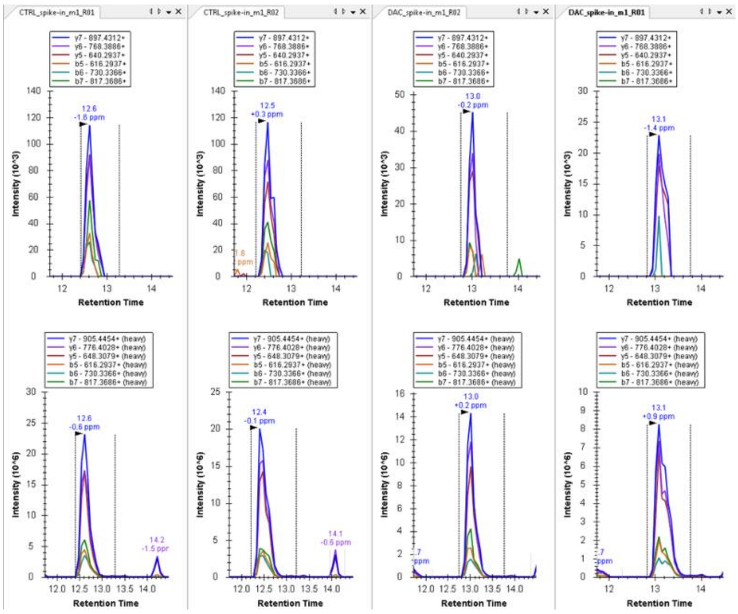

CTRL_spike-in_m1_R01
CTRL_spike-in_m1_R02
DAC_spike-in_m1_R02
DAC_spike-in_m1_R01
y7 - 897.4312+
y6 - 768.3886+
y5 - 640.2937+
b5 - 616.2937+
b6 - 730.3366+
b7 - 817.3686+
y7 - 905.4454+ (heavy)
y6 - 776.4028+ (heavy)
y5 - 648.3079+ (heavy)
b5 - 616.2937+ (heavy)
b6 - 730.3366+ (heavy)
b7 - 817.3686+ (heavy)
Intensity (10^3)
Intensity (10^6)
Retention Time

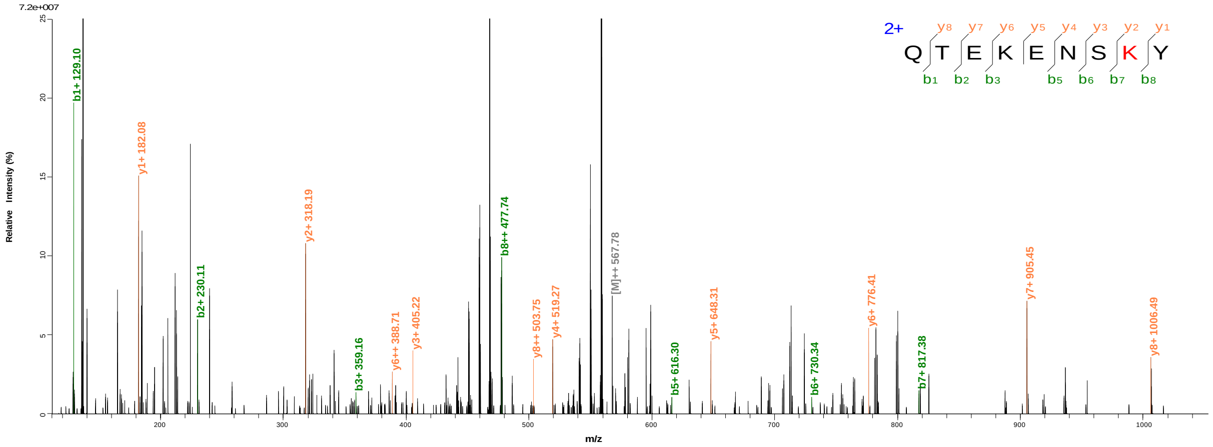

7.2e+007
2+
Q T E K E N S K Y
b1+ 129.10
y1+ 182.08
b2+ 230.11
y2+ 318.19
b3+ 359.16
y6++ 388.71
y3+ 405.22
b8++ 477.74
y8++ 503.75
y4+ 519.27
[M]++ 567.78
b5+ 616.30
y5+ 648.31
b6+ 730.34
y6+ 776.41
b7+ 817.38
y7+ 905.45
y8+ 1006.49
Relative Intensity (%)
m/z

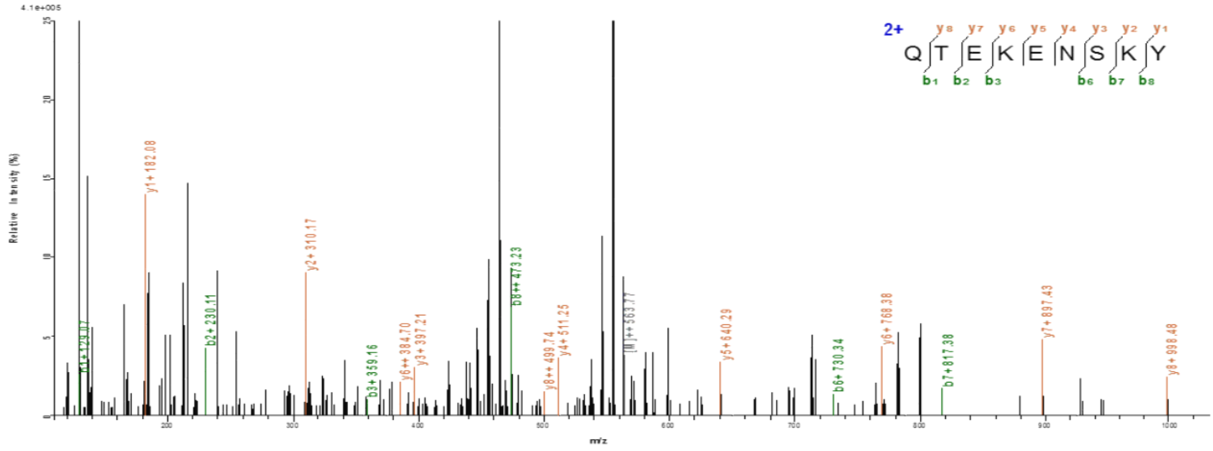

4.1e+005
2+
Q T E K E N S K Y
y1+ 182.08
b2+ 230.11
y2+ 310.17
b3+ 359.16
b8++ 473.23
b6+ 730.34
y6+ 768.38
b7+ 817.38
y7+ 897.43
y8+ 998.48
Relative Intensity (%)
m/z

KPRHQPGPAL

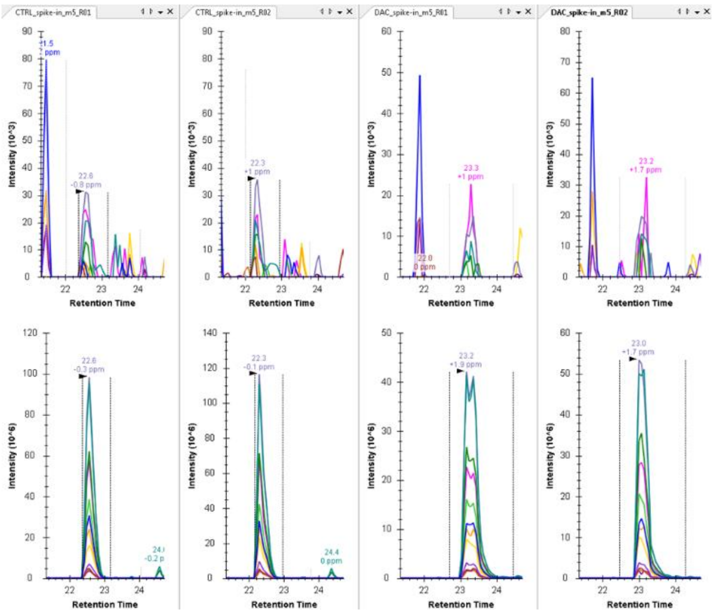
CTRL_spike-in_m5_R81
CTRL_spike-in_m5_R82
DAC_spike-in_m5_R81
DAC_spike-in_m5_R82
Intensity (10^3)
Intensity (10^6)
Retention Time

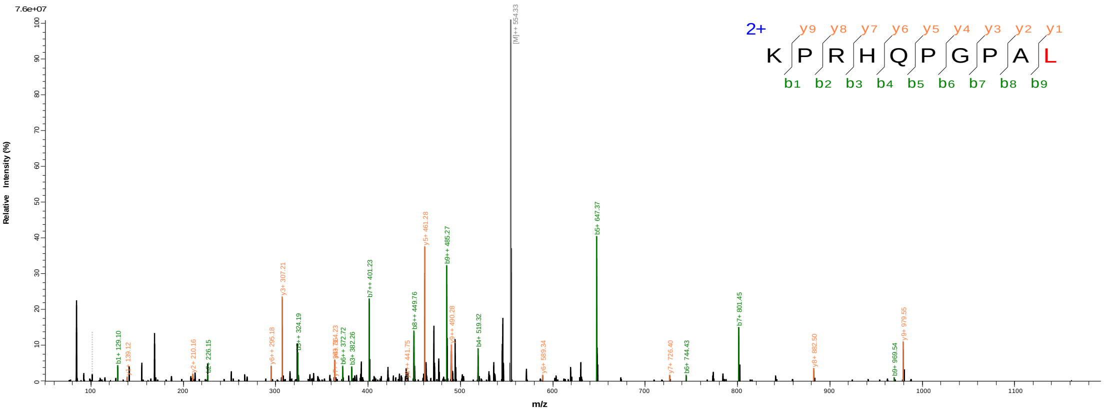
7.6e+07
Relative Intensity (%)
m/z
2+
y9 y8 y7 y6 y5 y4 y3 y2 y1
K P R H Q P G P A L
b1 b2 b3 b4 b5 b6 b7 b8 b9

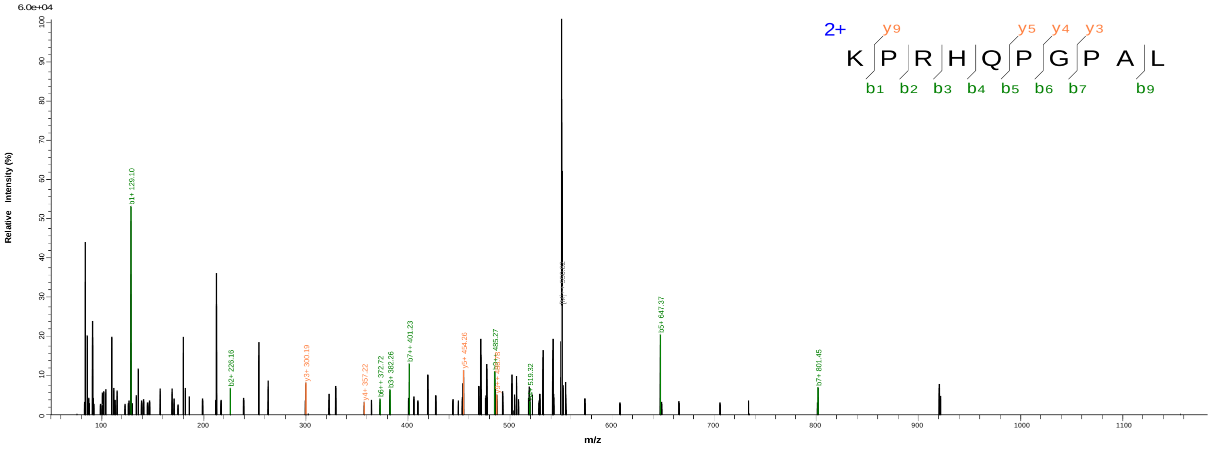
6.0e+04
Relative Intensity (%)
m/z
2+
y9 y5 y4 y3
K P R H Q P G P A L
b1 b2 b3 b4 b5 b6 b7 b9

APKSSSGFSL

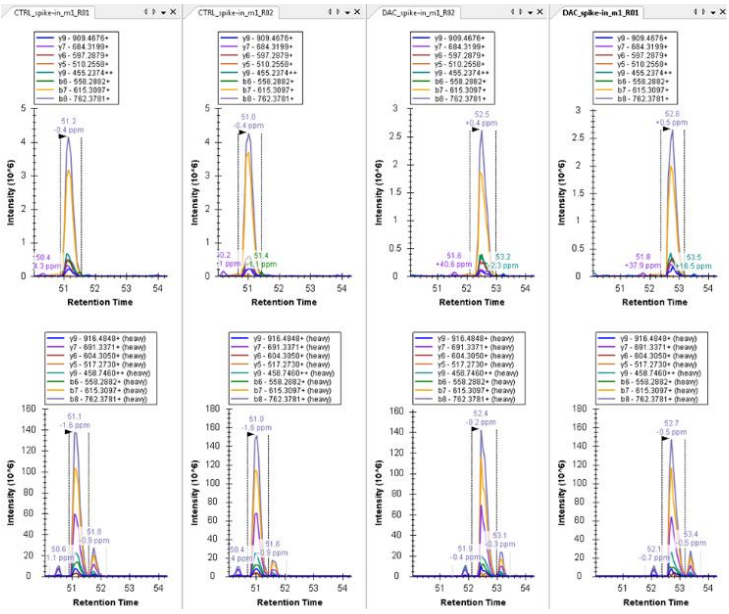

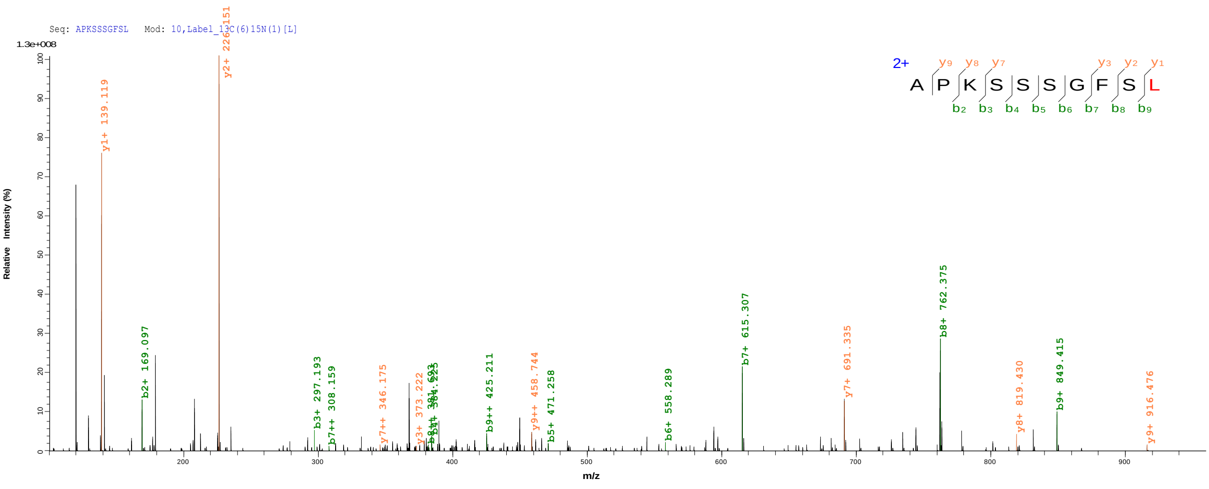
Seq: APKSSSGFSL Mod: 10,Label_13C(6)15N(1)[L]
Relative Intensity (%)
m/z
2+
A P K S S S G F S L

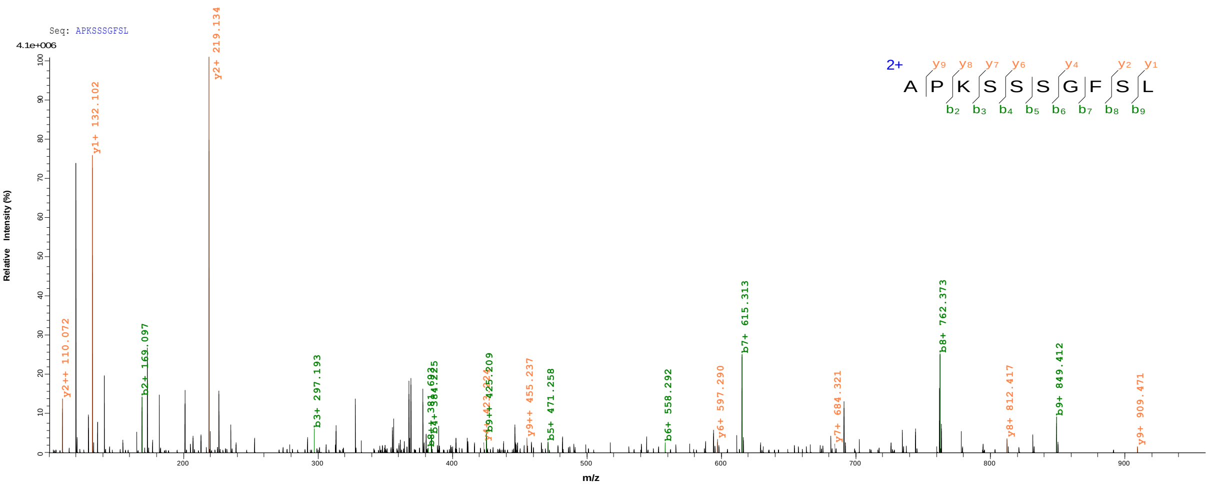
Seq: APKSSSGFSL
Relative Intensity (%)
m/z
2+
A P K S S S G F S L

YSETSLSNGGKY

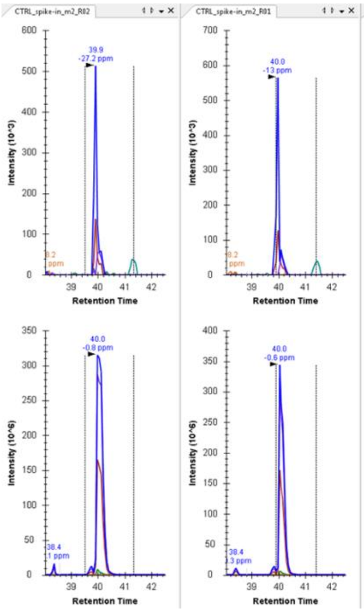

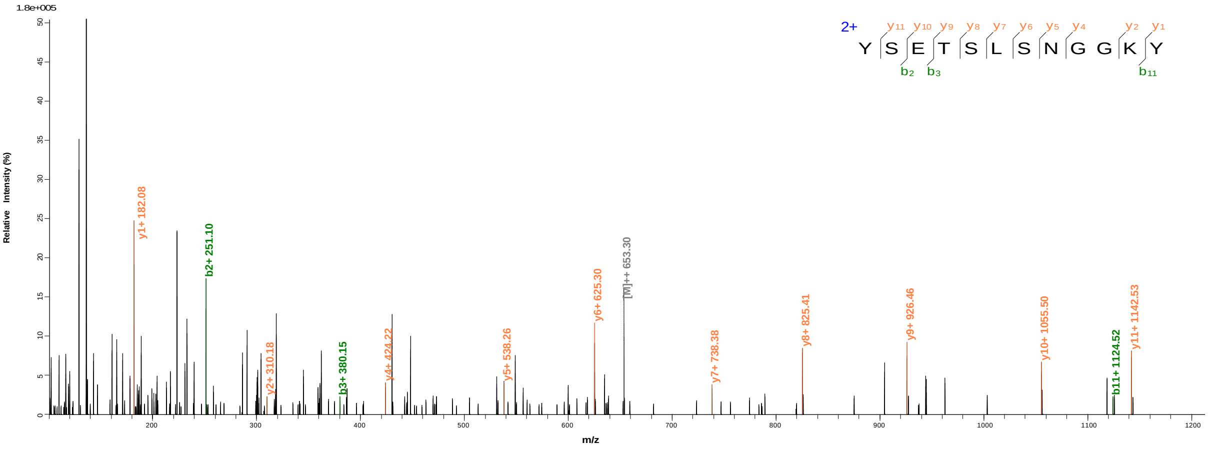

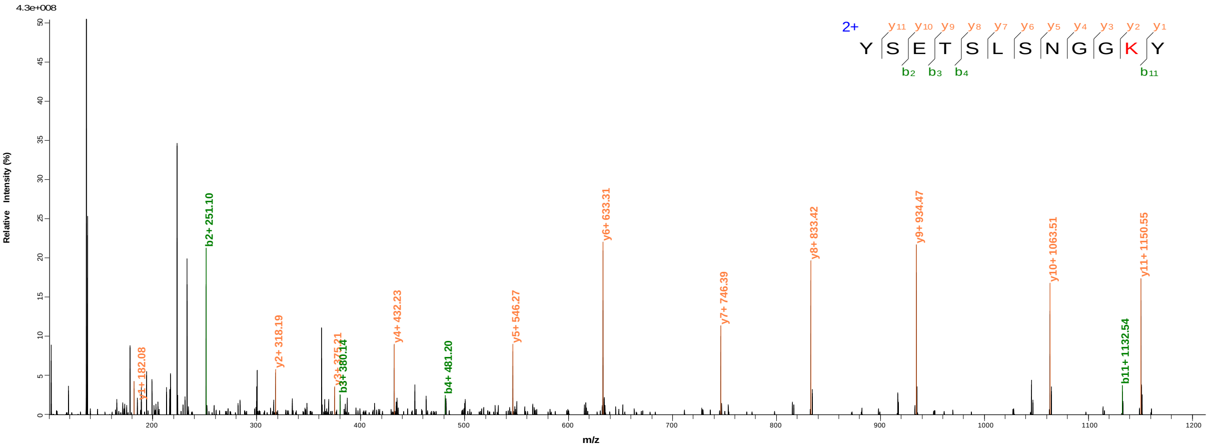

RPPHPRPSL

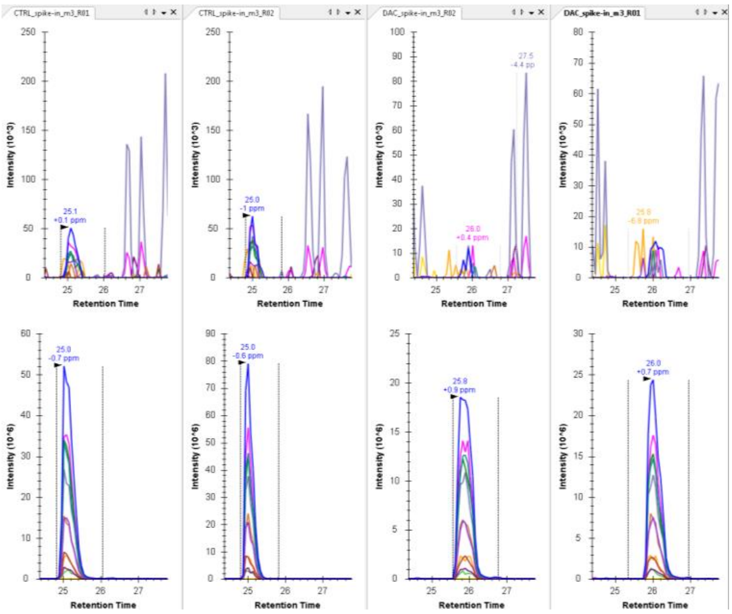

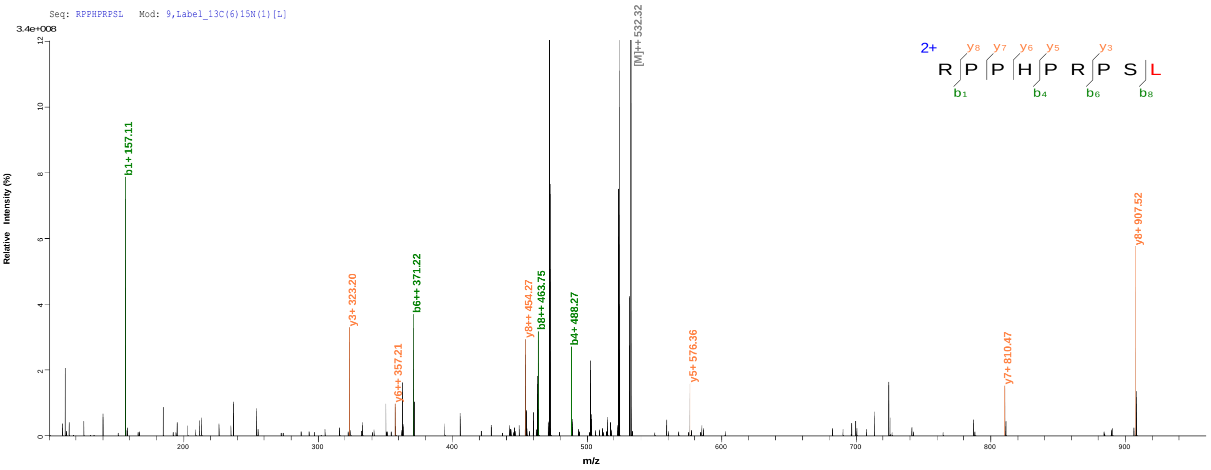
Seq: RPPHPRPSL Mod: 9,Label_13C(6)15N(1)[L]
2+
R P P H P R P S L
m/z
Relative Intensity (%)

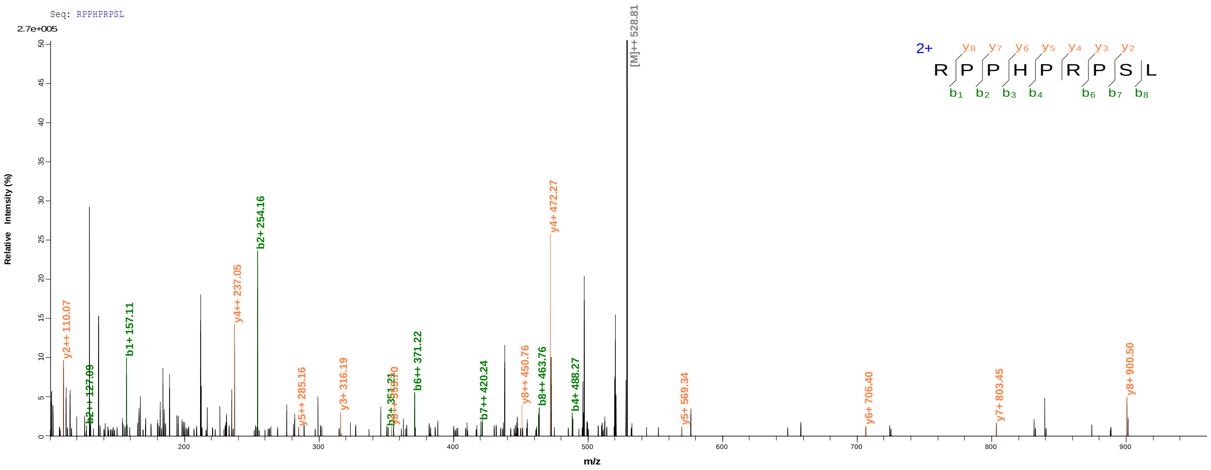
Seq: RPPHPRPSL
2+
R P P H P R P S L
m/z
Relative Intensity (%)

KFLVQNNVF

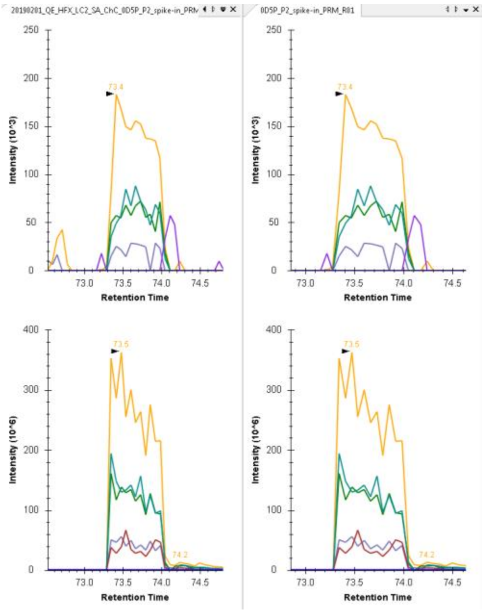
Intensity (10^3)
Intensity (10^6)
Retention Time

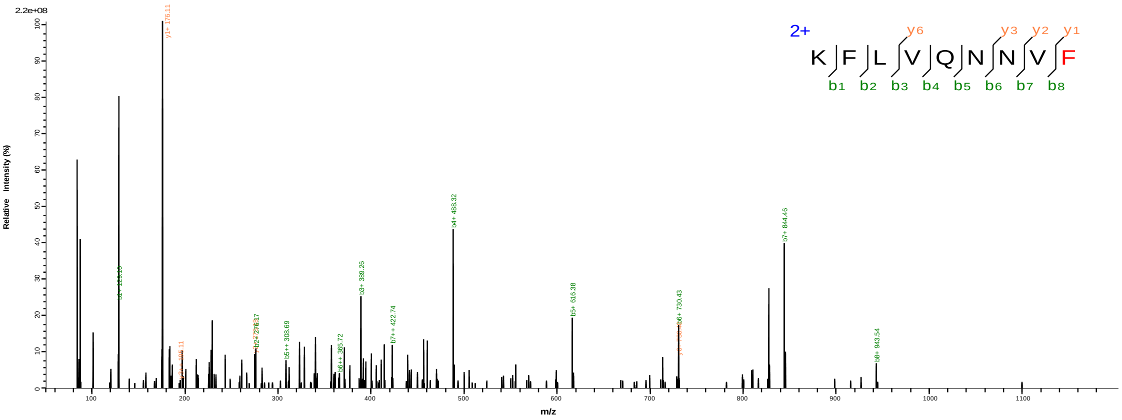
2.2e+08
Relative Intensity (%)
m/z
2+
K F L V Q N N V F
y6 y3 y2 y1
b1 b2 b3 b4 b5 b6 b7 b8

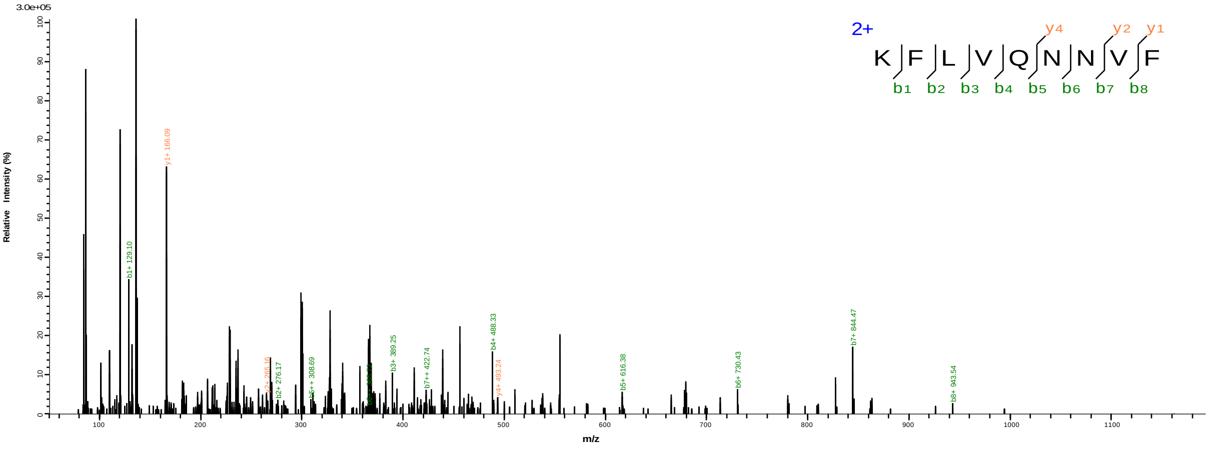
3.0e+05
Relative Intensity (%)
m/z
2+
K F L V Q N N V F
y4 y2 y1
b1 b2 b3 b4 b5 b6 b7 b8

HIKTQDTGY

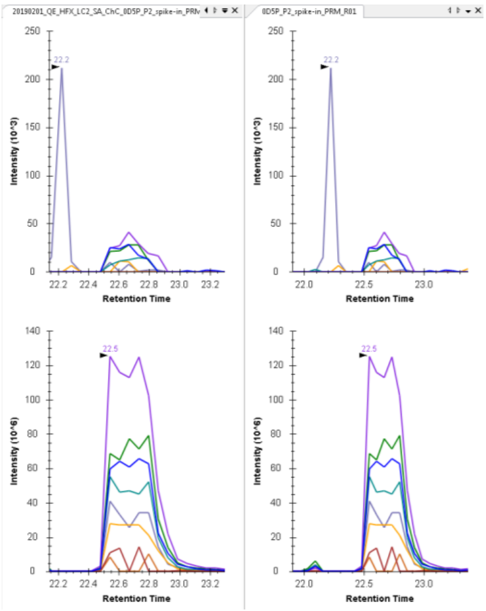

Intensity (10^3)
Intensity (10^6)
Retention Time

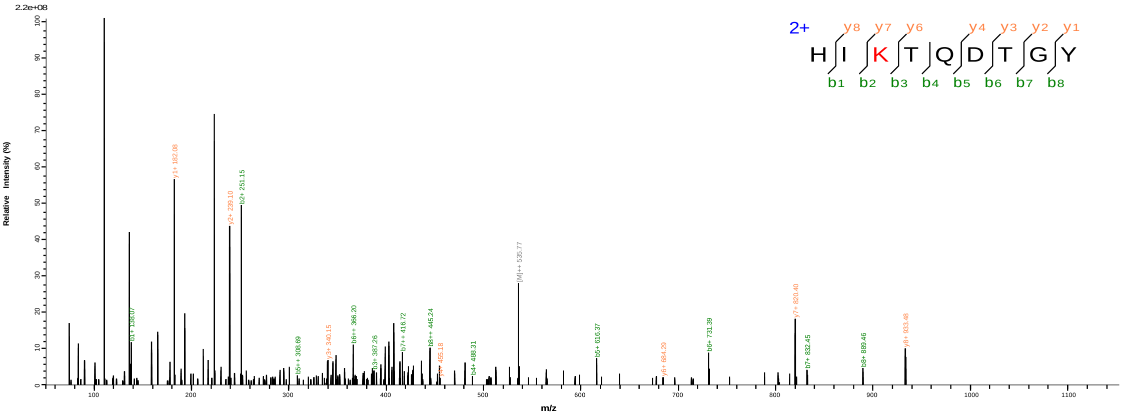

2.2e+08
Relative Intensity (%)
m/z
2+
H I K T Q D T G Y

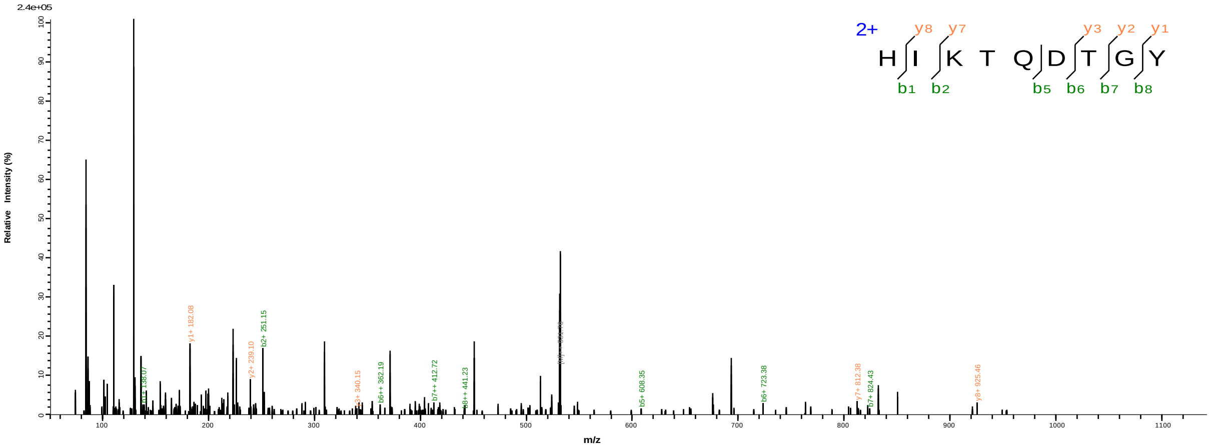

2.4e+05
Relative Intensity (%)
m/z
2+
H I K T Q D T G Y

VMKGINQGY

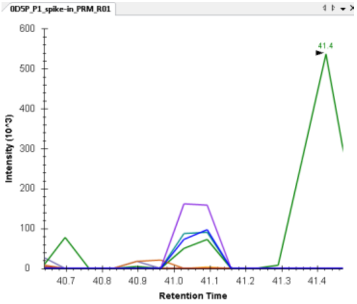

ODSP_P1_spike-in_PRM_R01
Intensity (10^3)
Retention Time
41.4

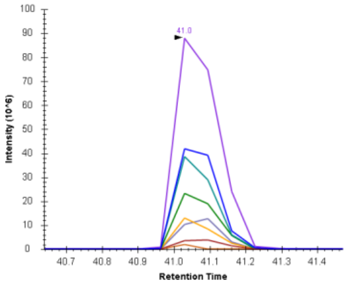

Intensity (10^6)
Retention Time
41.0

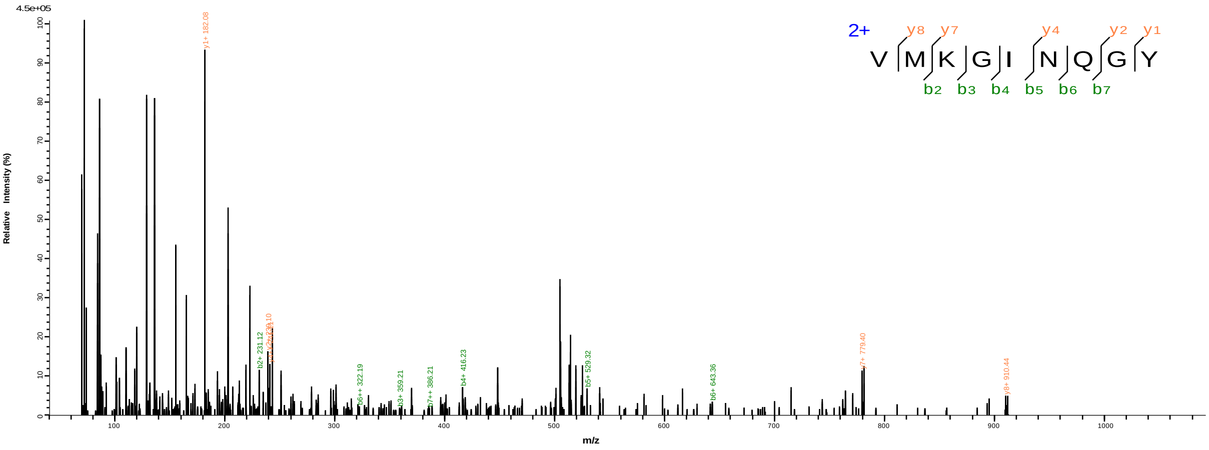

4.5e+05
Relative Intensity (%)
m/z
2+
V M K G I N Q G Y
y8 y7 y4 y2 y1
b2 b3 b4 b5 b6 b7

TSDQLGYSY

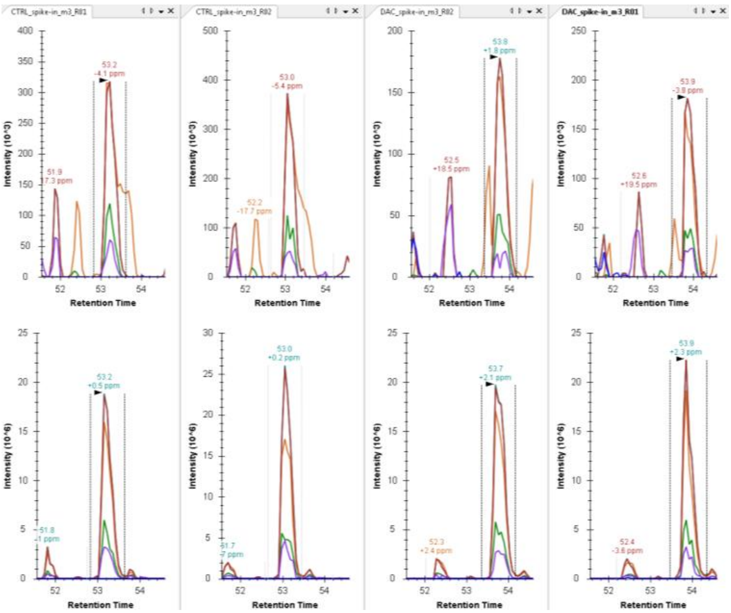
CTRL_spike-in_m3_R81
CTRL_spike-in_m3_R82
DAC_spike-in_m3_R82
DAC_spike-in_m3_R81
Intensity (10^3)
Intensity (10^6)
Retention Time

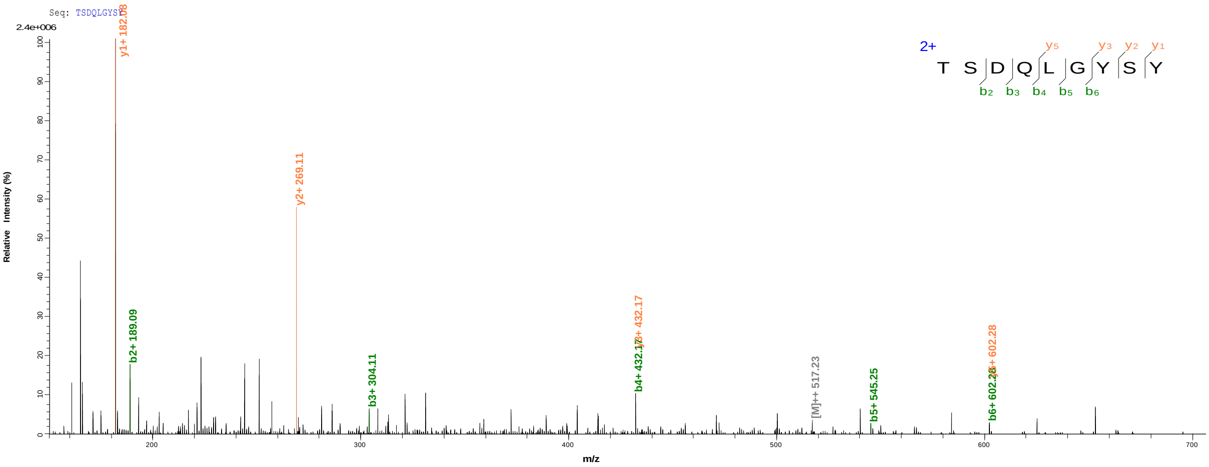
Seq: TSDQLGYSY
2.4e+006
Relative Intensity (%)
m/z
y1+ 182.08
y2+ 269.11
b2+ 189.09
b3+ 304.11
b4+ 432.17
b5+ 545.25
b6+ 602.26
[M]++ 517.23
2+
T S D Q L G Y S Y
y5 y3 y2 y1
b2 b3 b4 b5 b6

SMDPVTGYQY

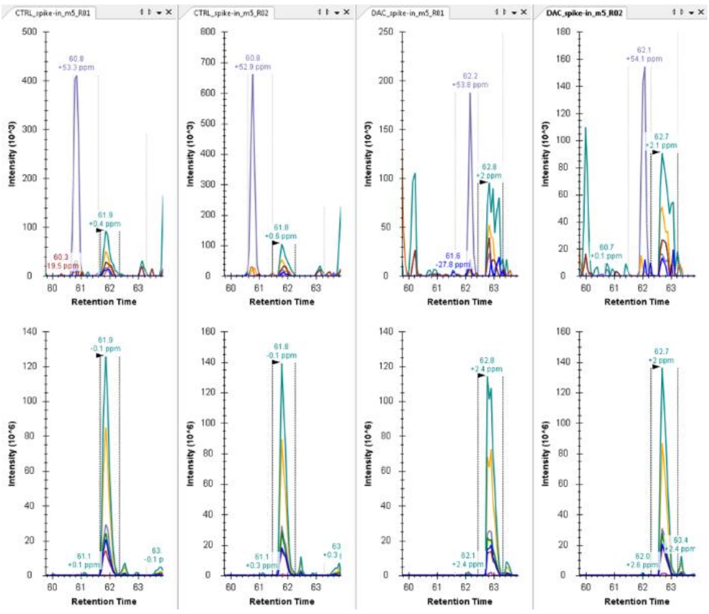
CTRL_spike-in_m5_R01
CTRL_spike-in_m5_R02
DAC_spike-in_m5_R01
DAC_spike-in_m5_R02
Intensity (10^3)
Intensity (10^6)
Retention Time

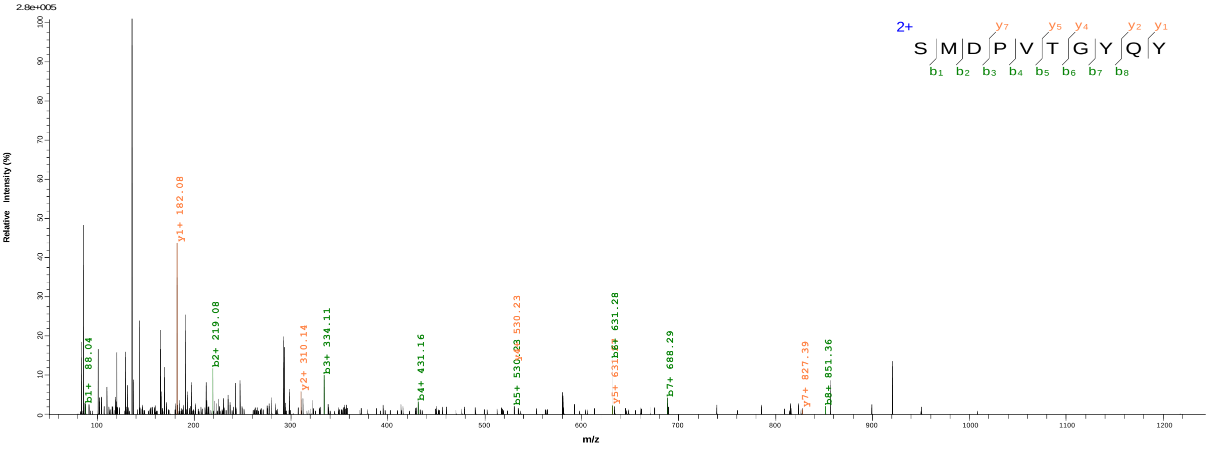
2.8e+005
Relative Intensity (%)
m/z
2+
S M D P V T G Y Q Y
y1+ 182.08
b1+ 88.04
b2+ 219.08
y2+ 310.14
b3+ 334.11
b4+ 431.16
b7+ 688.29
y7+ 827.39
b8+ 851.36

SSDYVIPIGTY

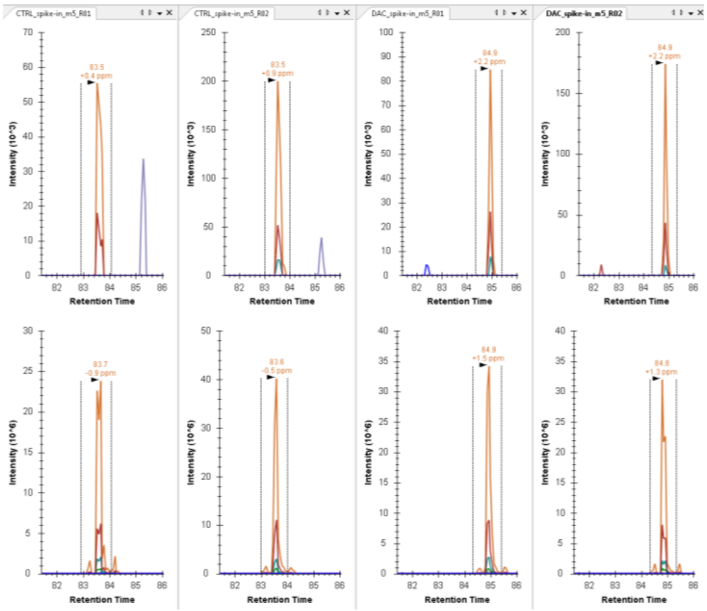

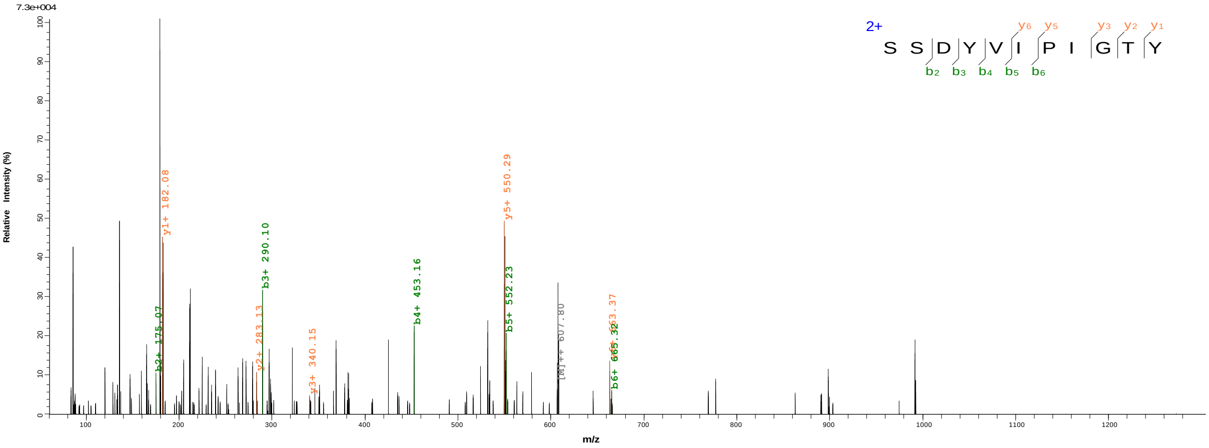

APAVIHAL

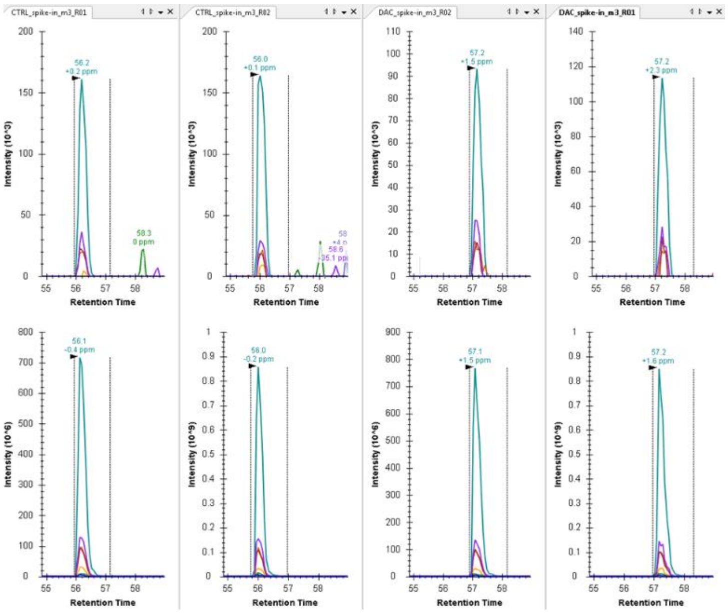

CTRL_spike-in_m3_R01
CTRL_spike-in_m3_R02
DAC_spike-in_m3_R02
DAC_spike-in_m3_R01
Intensity (10^3)
Intensity (10^6)
Intensity (10^9)
Retention Time

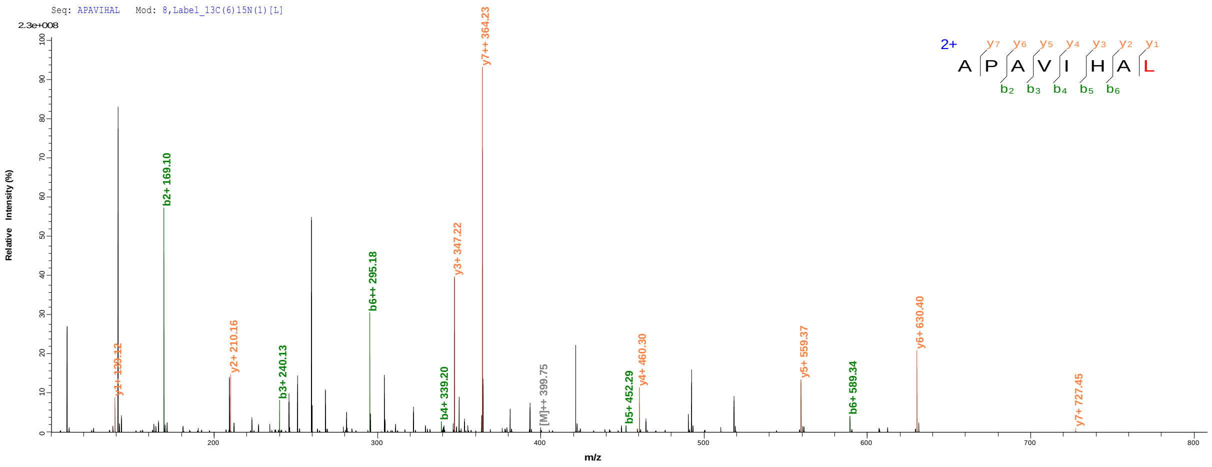

Seq: APAVIHAL Mod: 8,Label_13C(6)15N(1)[L]
2.3e+008
Relative Intensity (%)
m/z
2+
A P A V I H A L
b2+ 169.10
b3+ 240.13
b4+ 339.20
b5+ 452.29
b6+ 589.34
b6++ 295.18
y2+ 210.16
y3+ 347.22
y4+ 460.30
y5+ 559.37
y6+ 630.40
y7+ 727.45
y7++ 364.23
[M]++ 399.75

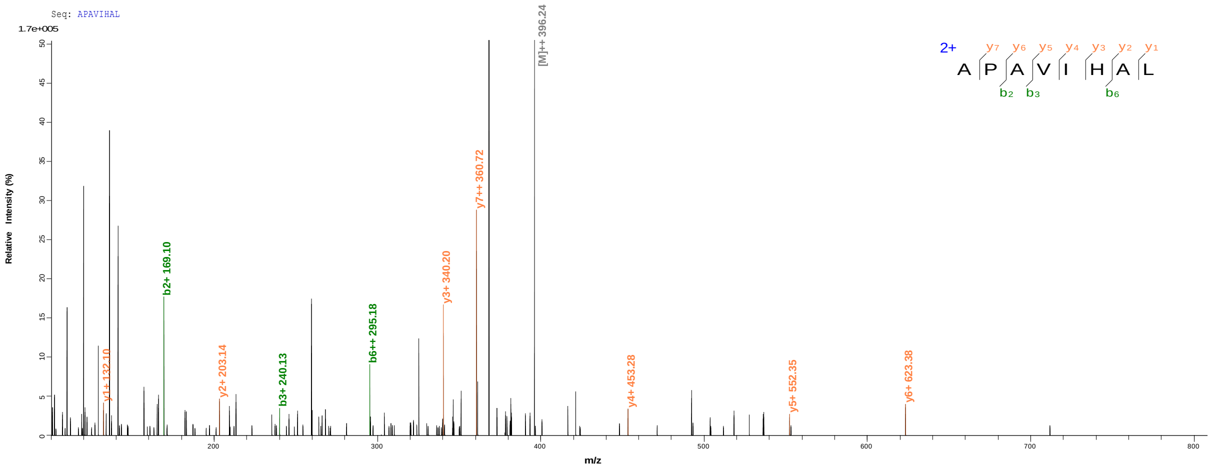

Seq: APAVIHAL
1.7e+005
Relative Intensity (%)
m/z
2+
A P A V I H A L
b2+ 169.10
b3+ 240.13
b6++ 295.18
y1+ 132.10
y2+ 203.14
y3+ 340.20
y4+ 453.28
y5+ 552.35
y6+ 623.38
y7++ 360.72
[M]++ 396.24

APKLMVRPF

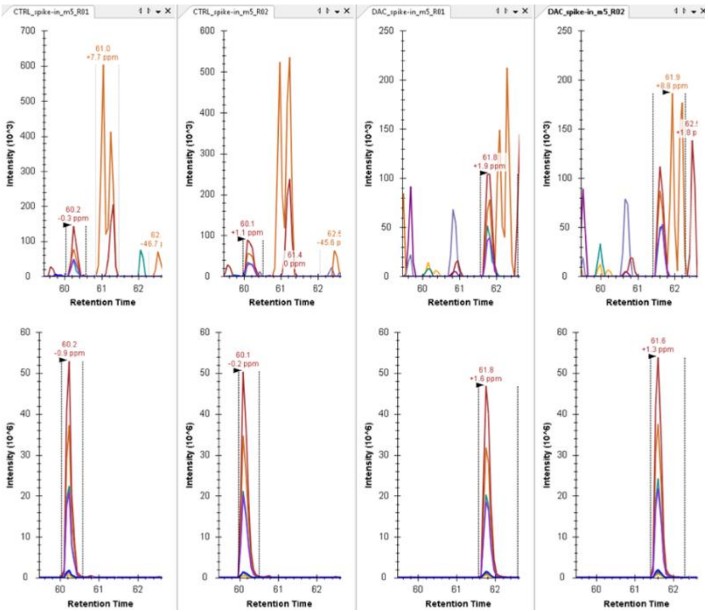

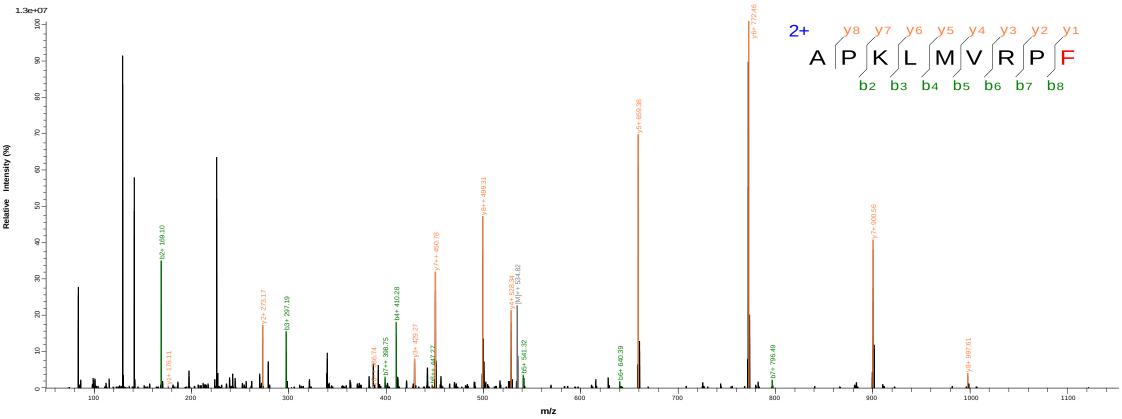

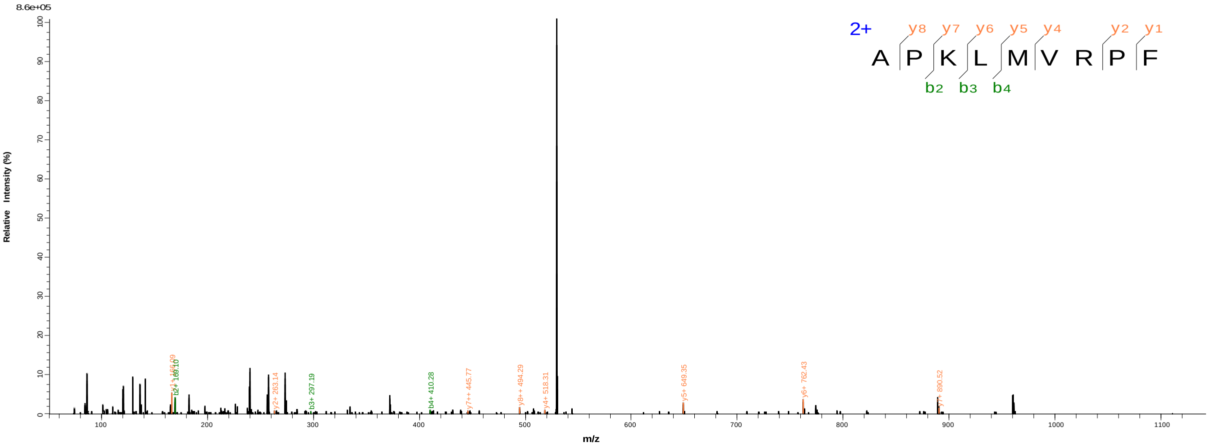

AYAIIKEEL

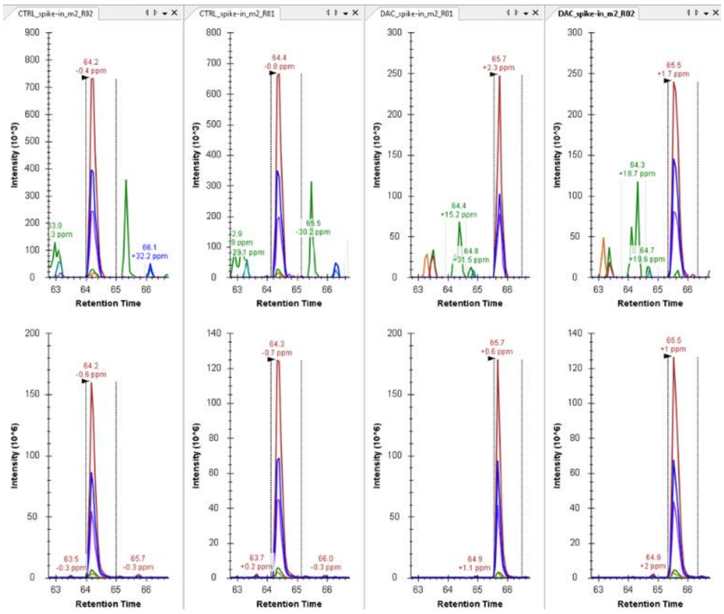
CTRL_spike-in_m2_R02
CTRL_spike-in_m2_R01
DAC_spike-in_m2_R01
DAC_spike-in_m2_R02
Intensity (10^3)
Intensity (10^6)
Retention Time

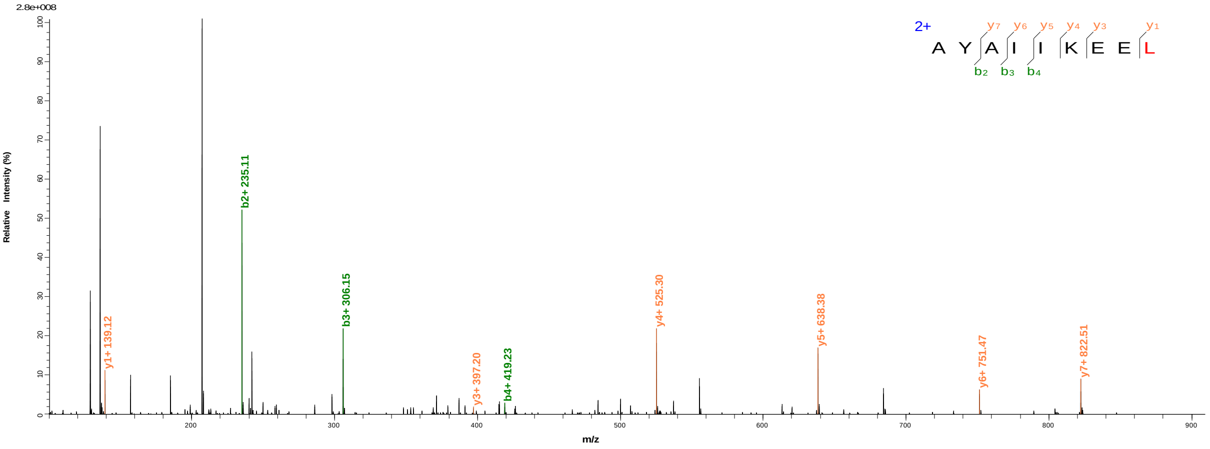
2.8e+008
Relative Intensity (%)
m/z
2+
A Y A I I K E E L
y7 y6 y5 y4 y3 y1
b2 b3 b4
b2+ 235.11
b3+ 306.15
b4+ 419.23
y1+ 139.12
y3+ 397.20
y4+ 525.30
y5+ 638.38
y6+ 751.47
y7+ 822.51

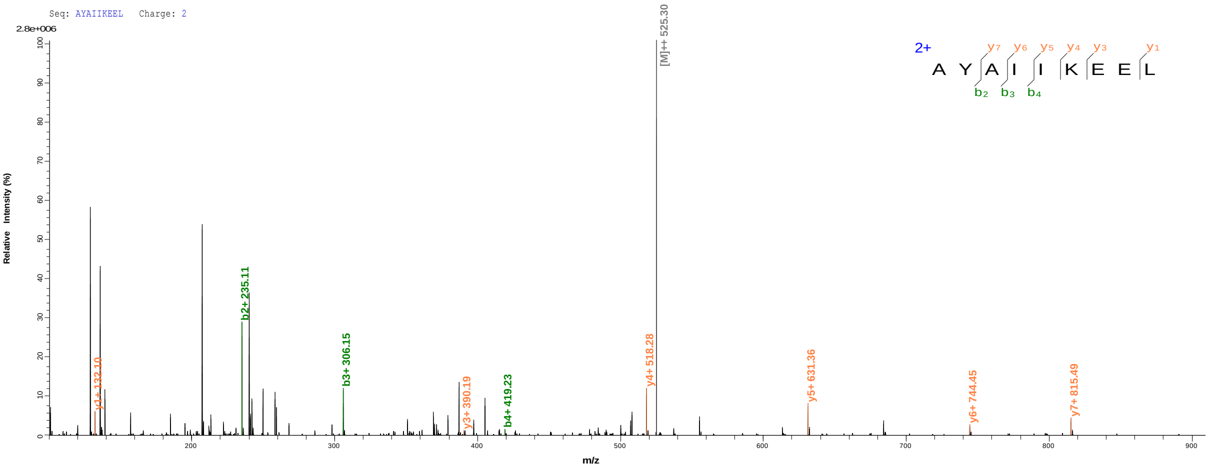
Seq: AYAIIKEEL Charge: 2
2.8e+006
Relative Intensity (%)
m/z
2+
A Y A I I K E E L
y7 y6 y5 y4 y3 y1
b2 b3 b4
[M]++ 525.30
b2+ 235.11
b3+ 306.15
b4+ 419.23
y1+ 132.10
y3+ 390.19
y4+ 518.28
y5+ 631.36
y6+ 744.45
y7+ 815.49

KYLTVKDYL

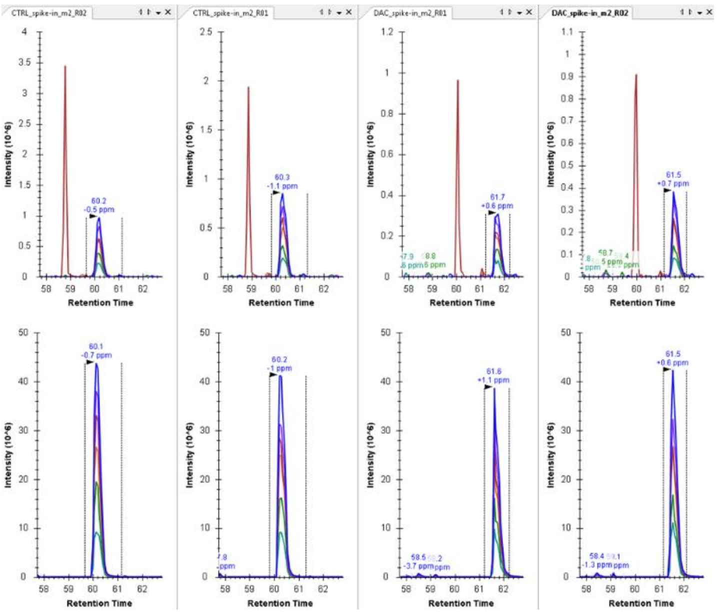

CTRL_spike-in_m2_R02
CTRL_spike-in_m2_R01
DAC_spike-in_m2_R01
DAC_spike-in_m2_R02
Intensity (10^6)
Retention Time
60.2
-0.5 ppm
60.3
-1.1 ppm
61.7
+0.6 ppm
61.5
+0.7 ppm
60.1
-0.7 ppm
60.2
-1 ppm
61.6
+1.1 ppm
61.5
+0.6 ppm

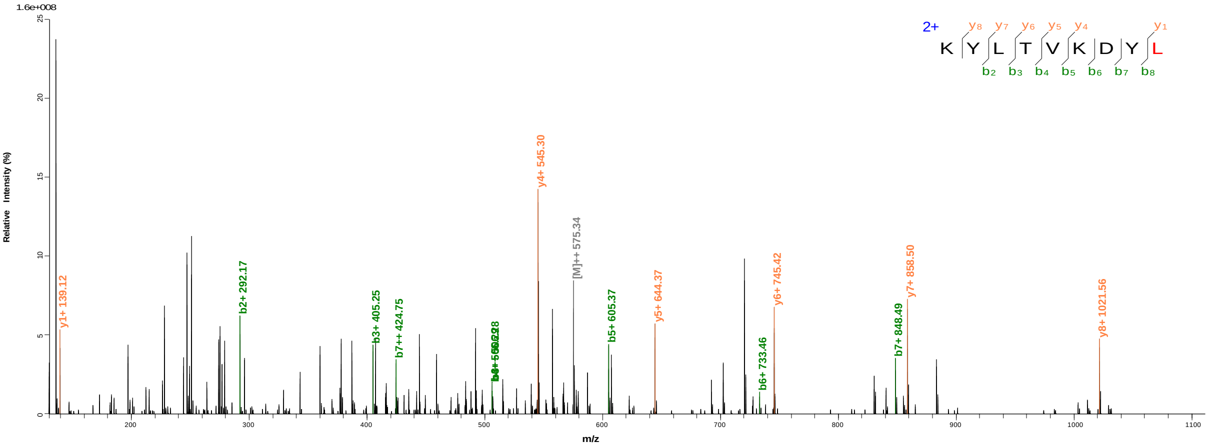

1.6e+008
Relative Intensity (%)
m/z
2+
K Y L T V K D Y L
y1+ 139.12
b2+ 292.17
b3+ 405.25
b7++ 424.75
y4+ 545.30
[M]++ 575.34
b5+ 605.37
y5+ 644.37
b6+ 733.46
y6+ 745.42
b7+ 848.49
y7+ 858.50
y8+ 1021.56

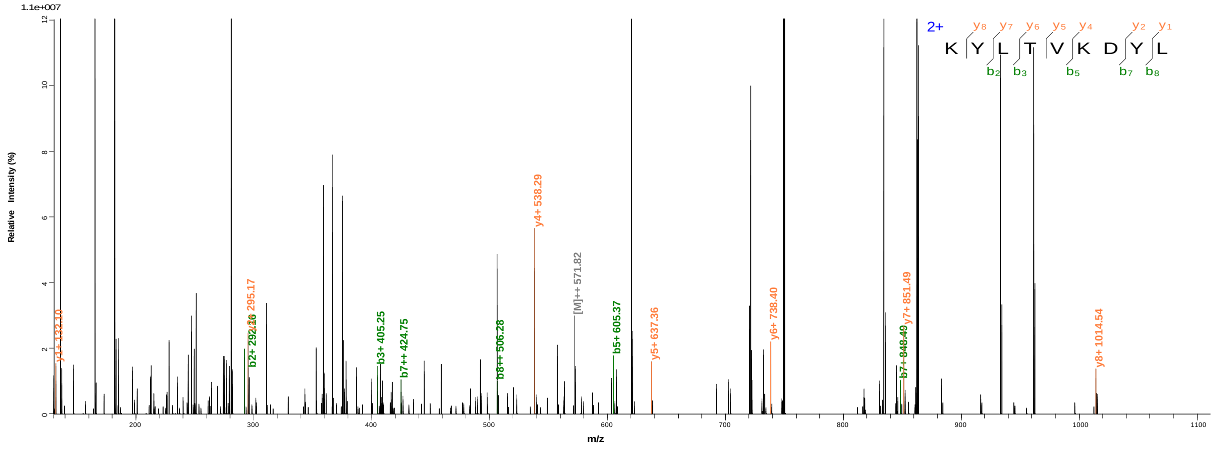

1.1e+007
Relative Intensity (%)
m/z
2+
K Y L T V K D Y L
b3+ 405.25
b7++ 424.75
b8++ 506.28
y4+ 538.29
[M]++ 571.82
b5+ 605.37
y5+ 637.36
y6+ 738.40
y7+ 851.49
y8+ 1014.54

RYSGVNQSMLF

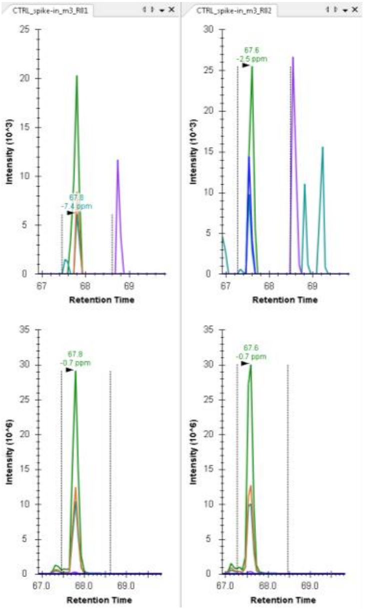

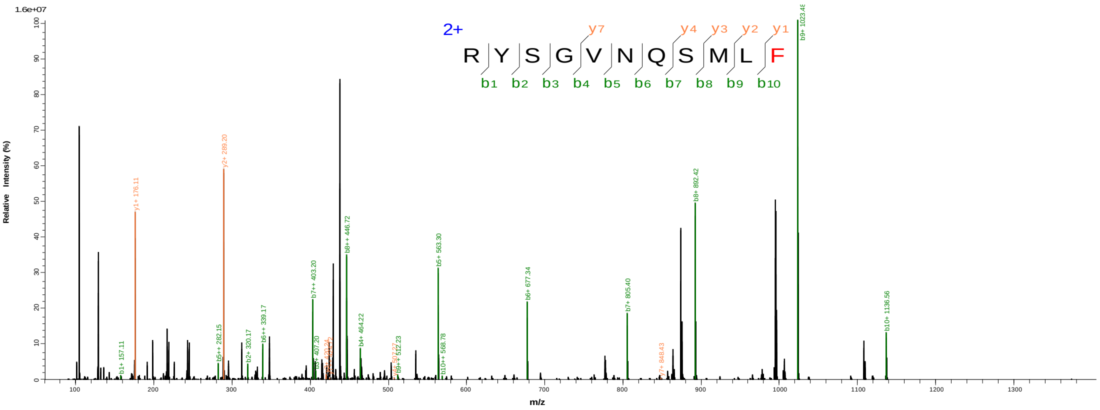

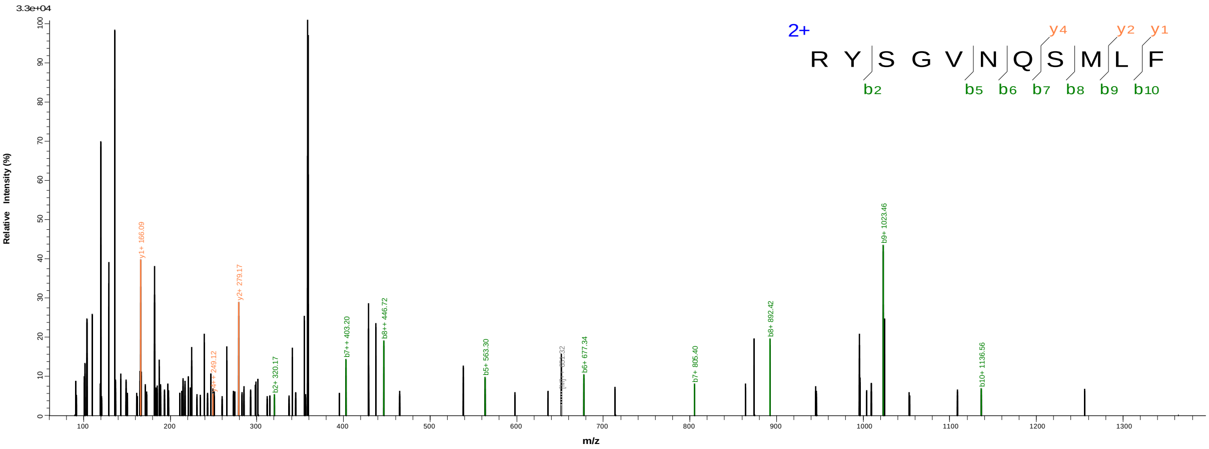

QTDPDVHRNGKY

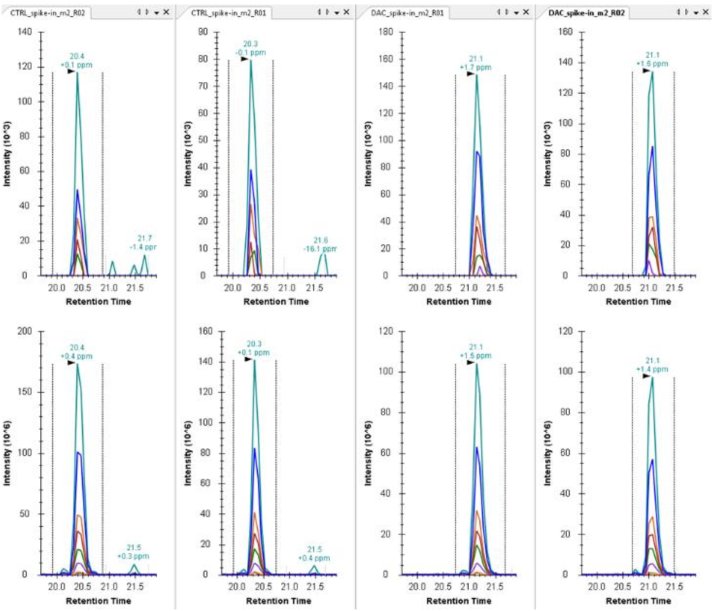

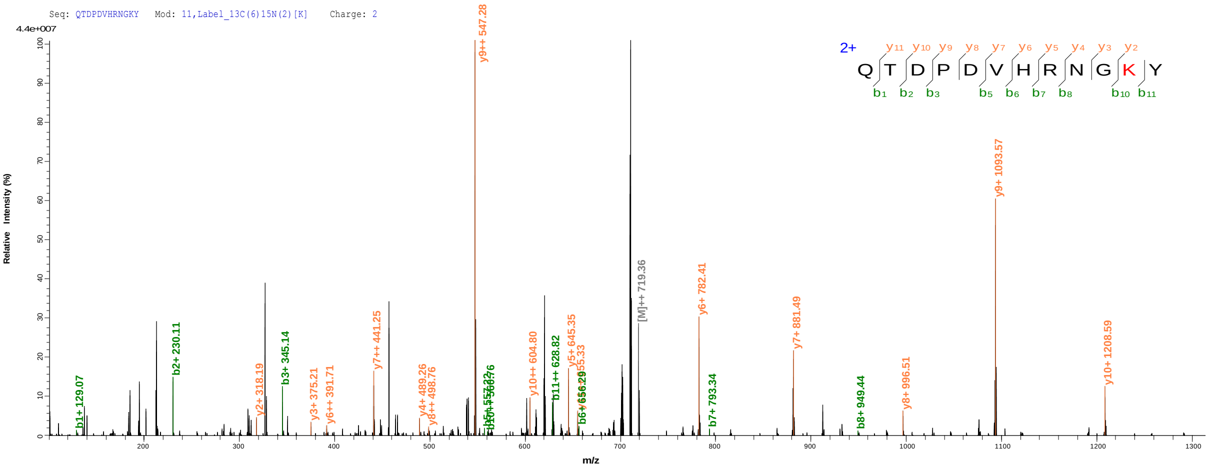

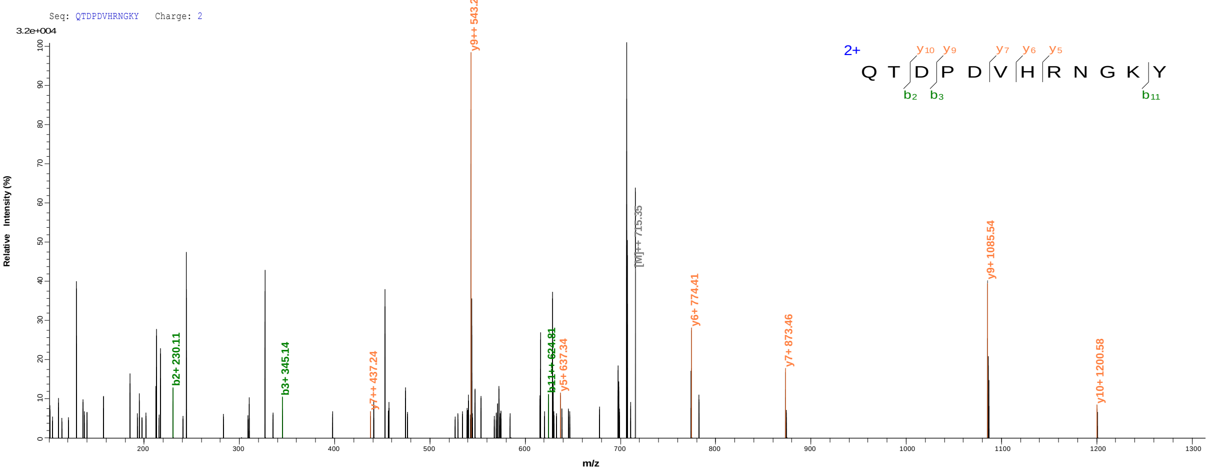

ETDRVTAHF

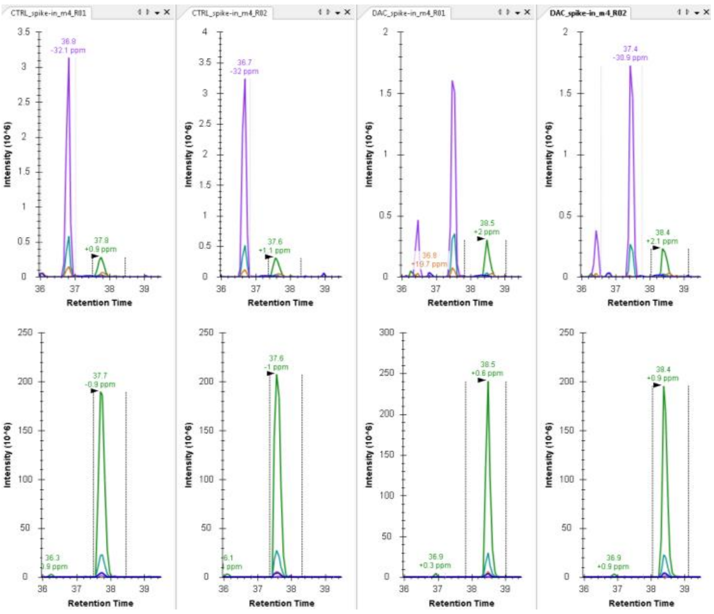

CTRL_spike-in_m4_R01
CTRL_spike-in_m4_R02
DAC_spike-in_m4_R01
DAC_spike-in_m4_R02
Intensity (10^6)
Retention Time

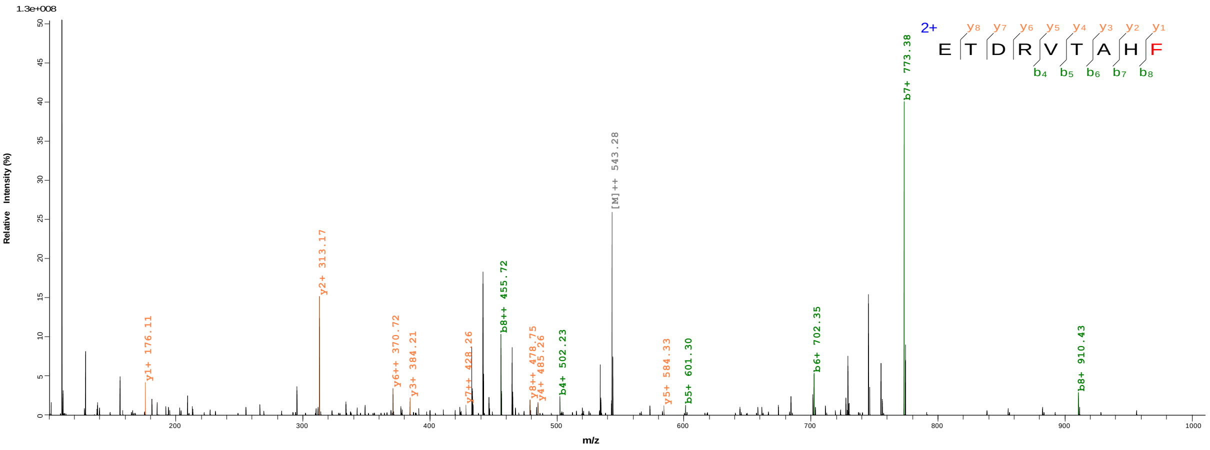

1.3e+008
Relative Intensity (%)
m/z
2+
E T D R V T A H F
b7+ 773.38
[M]++ 543.28
y2+ 313.17
b8++ 455.72
y1+ 176.11
b6+ 702.35
b8+ 910.43
b4+ 502.23
b5+ 601.30
y5+ 584.33

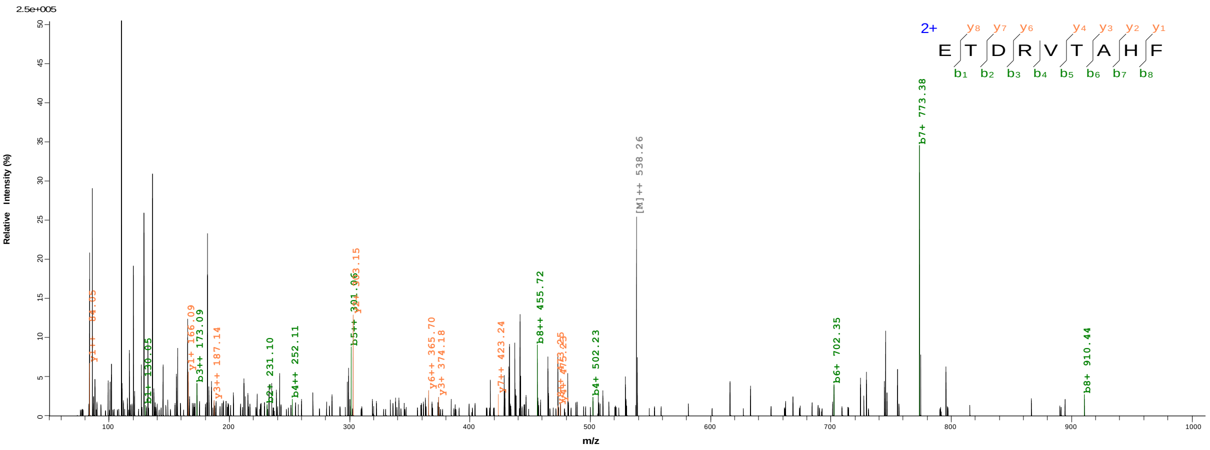

2.5e+005
Relative Intensity (%)
m/z
2+
E T D R V T A H F
b7+ 773.38
[M]++ 538.26
b8++ 455.72
b6+ 702.35
b8+ 910.44
b4+ 502.23
b4++ 252.11
b2+ 231.10
b3++ 173.09
y3++ 187.14
y6++ 365.70
y3+ 374.18
y7++ 423.24

RYIHPQQEAF

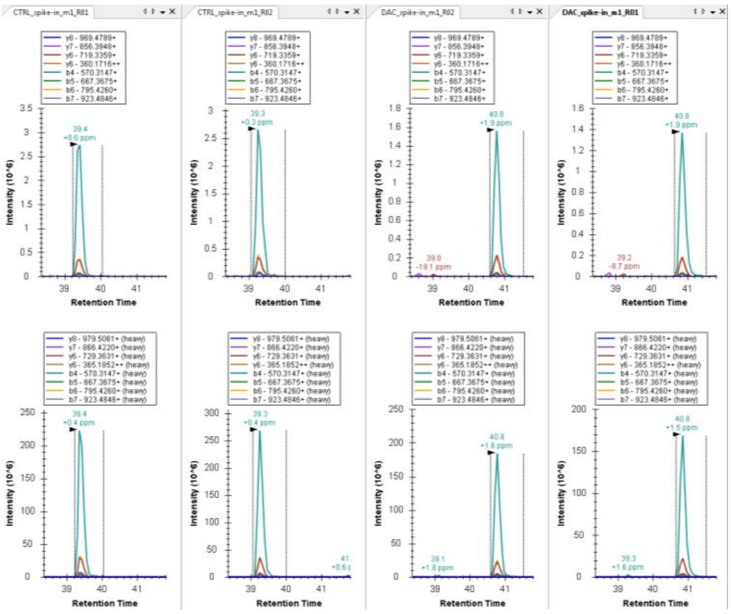

Retention Time
Intensity (10^6)

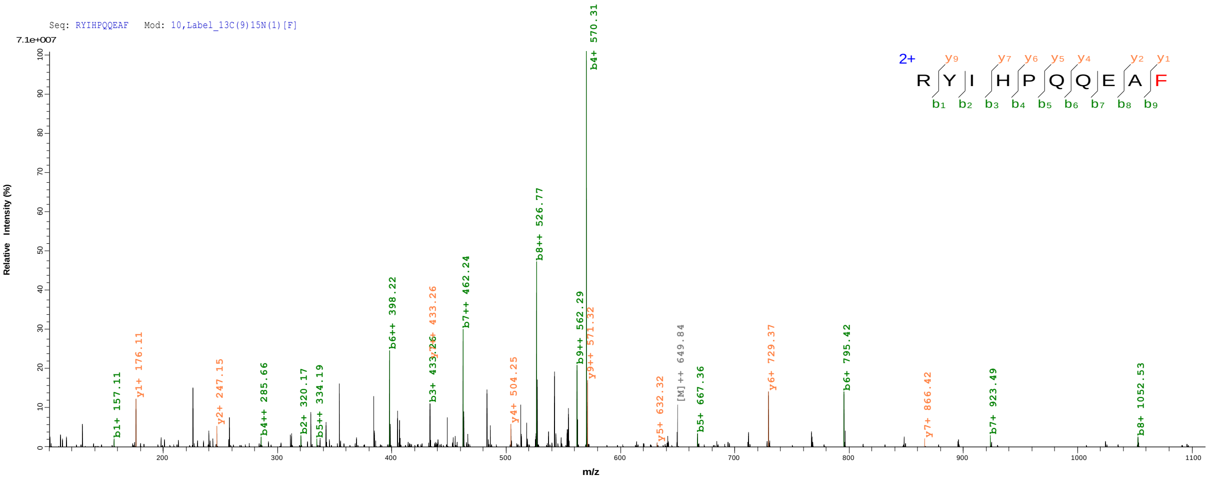

Seq: RYIHPQQEAF Mod: 10,Label_13C(9)15N(1)[F]
7.1e+007
Relative Intensity (%)
m/z
2+
R Y I H P Q Q E A F
b4+ 570.31
b8++ 526.77
b7++ 462.24
b6++ 398.22
b9++ 562.29
y9++ 571.32
y6+ 729.37
b6+ 795.42
y1+ 176.11
b1+ 157.11
y2+ 247.15
b4++ 285.66
b2+ 320.17
b5++ 334.19
b3+ 433.26
y4+ 504.25
y5+ 632.32
[M]++ 649.84
b5+ 667.36
y7+ 866.42
b7+ 923.49
b8+ 1052.53

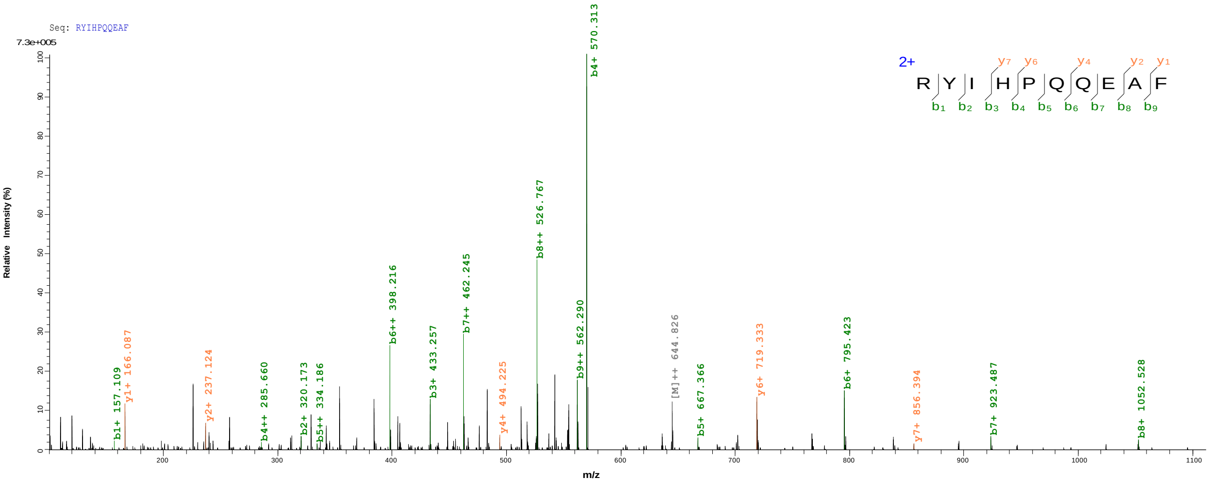

Seq: RYIHPQQEAF
7.3e+005
Relative Intensity (%)
m/z
2+
R Y I H P Q Q E A F
b4+ 570.313
b8++ 526.767
b7++ 462.245
b6++ 398.216
b3+ 433.257
b9++ 562.290
[M]++ 644.826
b6+ 795.423
y6+ 719.333
y1+ 166.087
b1+ 157.109
y2+ 237.124
b4++ 285.660
b2+ 320.173
b5++ 334.186
y4+ 494.225
b5+ 667.366
y7+ 856.394
b7+ 923.487
b8+ 1052.528

FIETESQLY

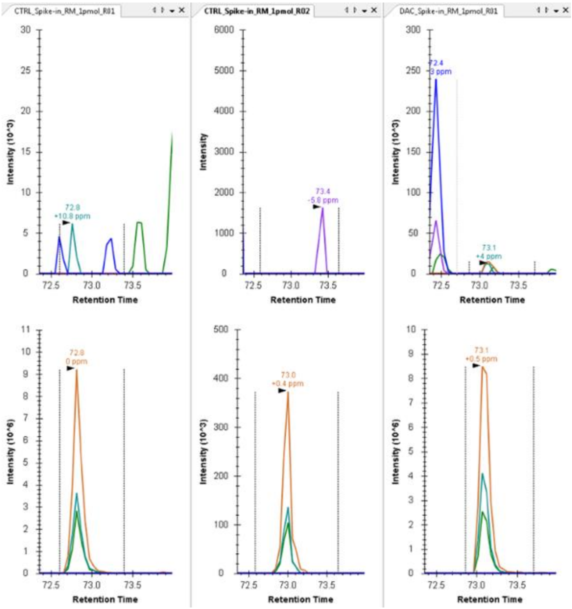

CTRL_Spike-in_RM_1pmol_R01
CTRL_Spike-in_RM_1pmol_R02
DAC_Spike-in_RM_1pmol_R01
Retention Time
Intensity (10^3)
Intensity
Intensity (10^6)

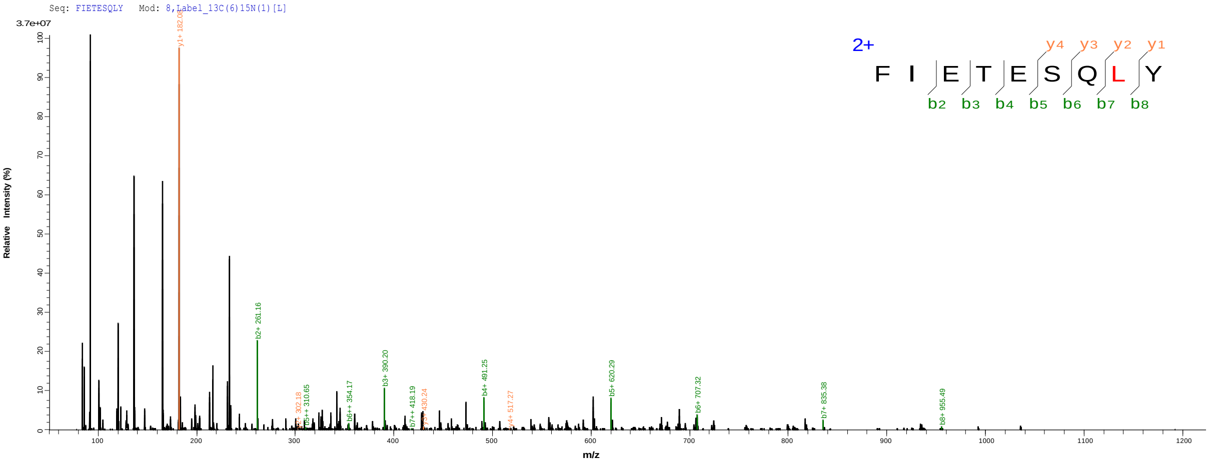

Seq: FIETESQLY Mod: 8,Label_13C(6)15N(1)[L]
3.7e+07
Relative Intensity (%)
m/z
2+
F I E T E S Q L Y
y4 y3 y2 y1
b2 b3 b4 b5 b6 b7 b8

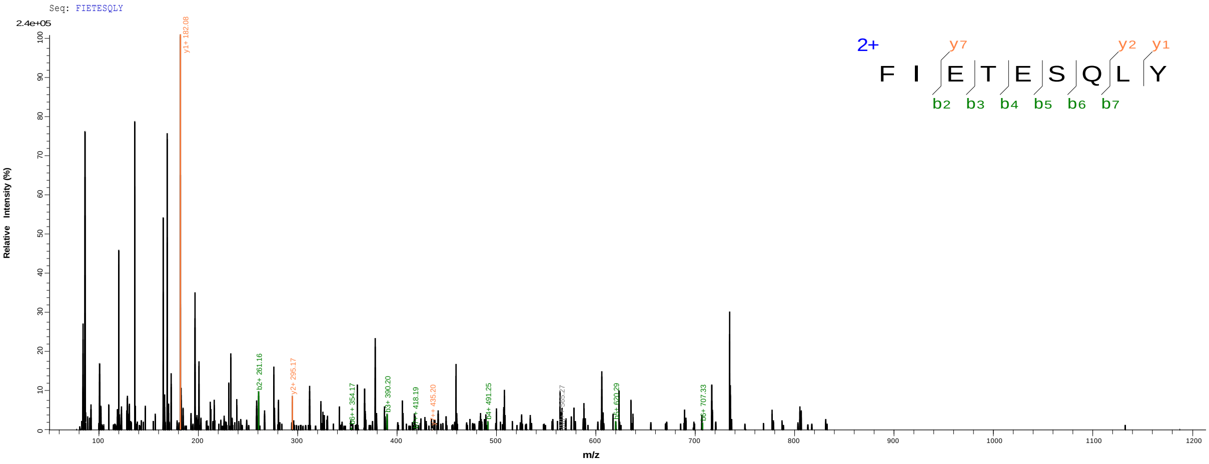

Seq: FIETESQLY
2.4e+05
Relative Intensity (%)
m/z
2+
F I E T E S Q L Y
y7 y2 y1
b2 b3 b4 b5 b6 b7

HYYSVRDTL

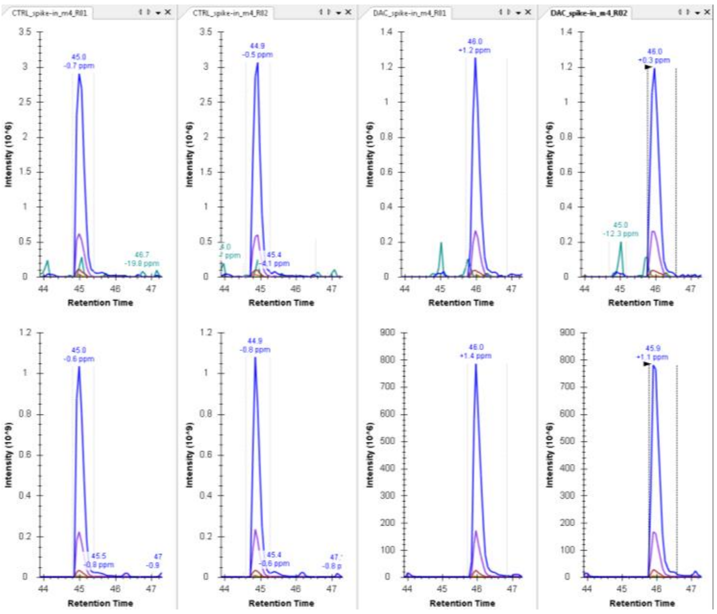

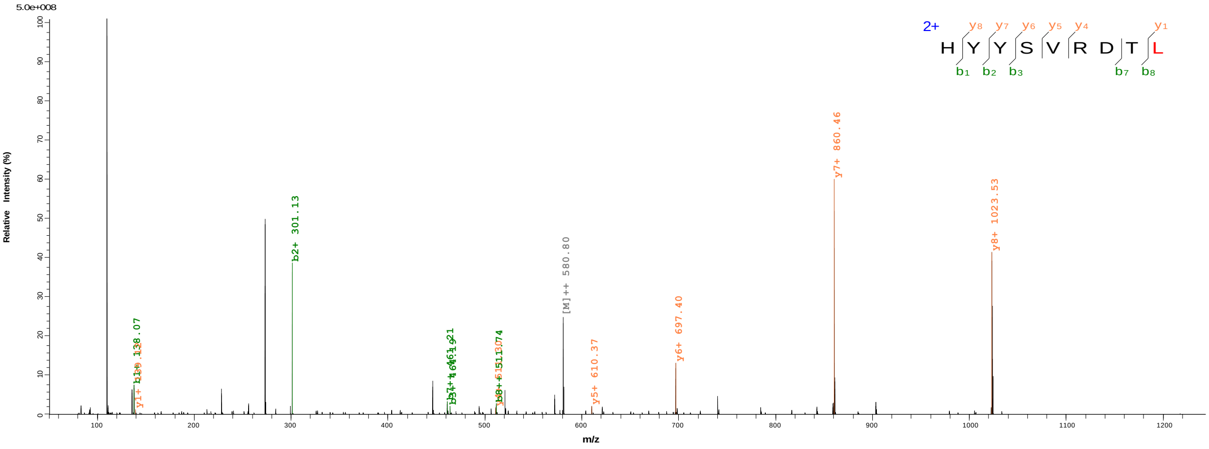

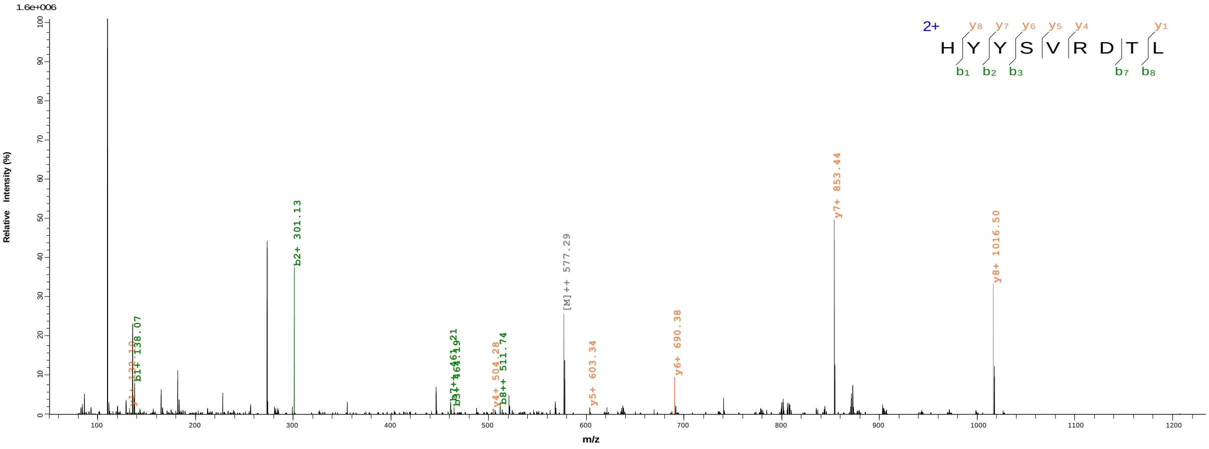

ESDYSKLWRY

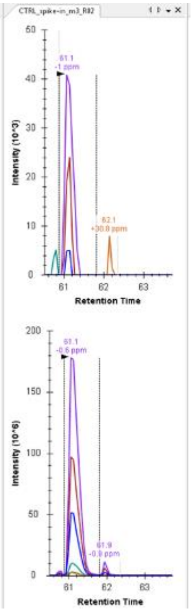

CTRL_spike-in_m3_R82
61.1
-1 ppm
62.1
+30.8 ppm
Intensity (10^3)
Retention Time
61.1
-0.6 ppm
61.9
-0.8 ppm
Intensity (10^6)
Retention Time

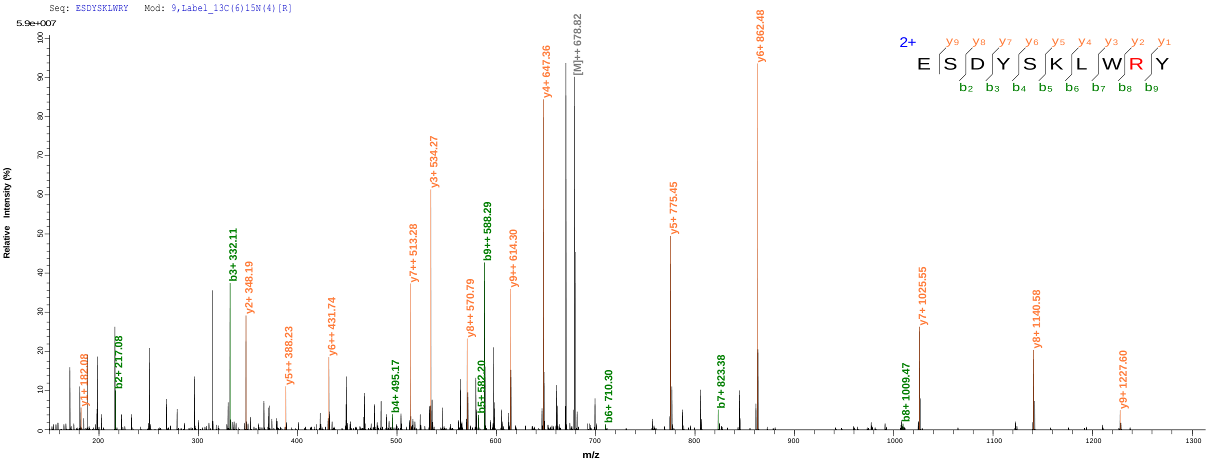

Seq: ESDYSKLWRY Mod: 9,Label_13C(6)15N(4)[R]
5.9e+007
Relative Intensity (%)
m/z
2+
E S D Y S K L W R Y
y1+ 182.08
b2+ 217.08
b3+ 332.11
y2+ 348.19
y5++ 388.23
y6++ 431.74
b4+ 495.17
y7++ 513.28
y3+ 534.27
y8++ 570.79
b5+ 582.20
b9++ 588.29
y9++ 614.30
y4+ 647.36
[M]++ 678.82
b6+ 710.30
y5+ 775.45
b7+ 823.38
y6+ 862.48
b8+ 1009.47
y7+ 1025.55
y8+ 1140.58
y9+ 1227.60

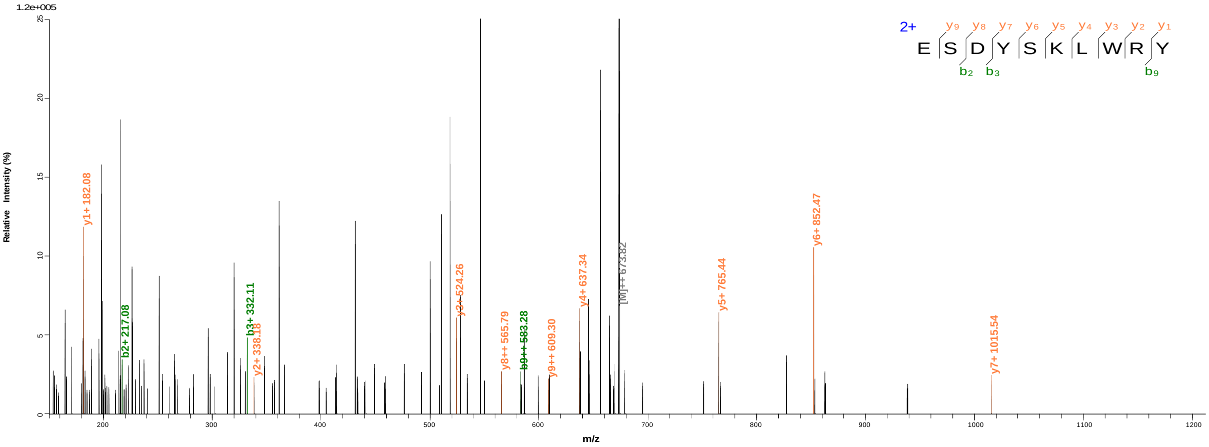

1.2e+005
Relative Intensity (%)
m/z
2+
E S D Y S K L W R Y
y1+ 182.08
b2+ 217.08
b3+ 332.11
y2+ 338.18
y3+ 524.26
y8++ 565.79
b9++ 583.28
y9++ 609.30
y4+ 637.34
[M]++ 673.82
y5+ 765.44
y6+ 852.47
y7+ 1015.54

HAIPHYVTM

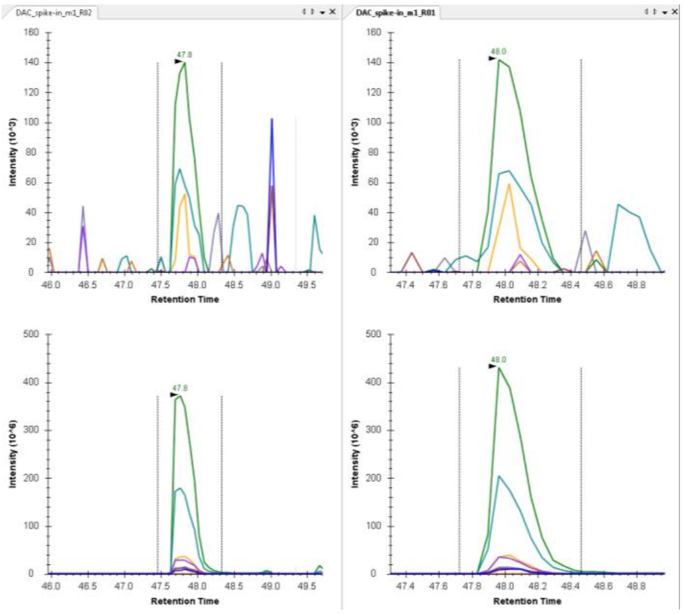

DAC_spike-in_m1_R02
DAC_spike-in_m1_R01
Intensity (10^3)
Intensity (10^6)
Retention Time
47.8
48.0

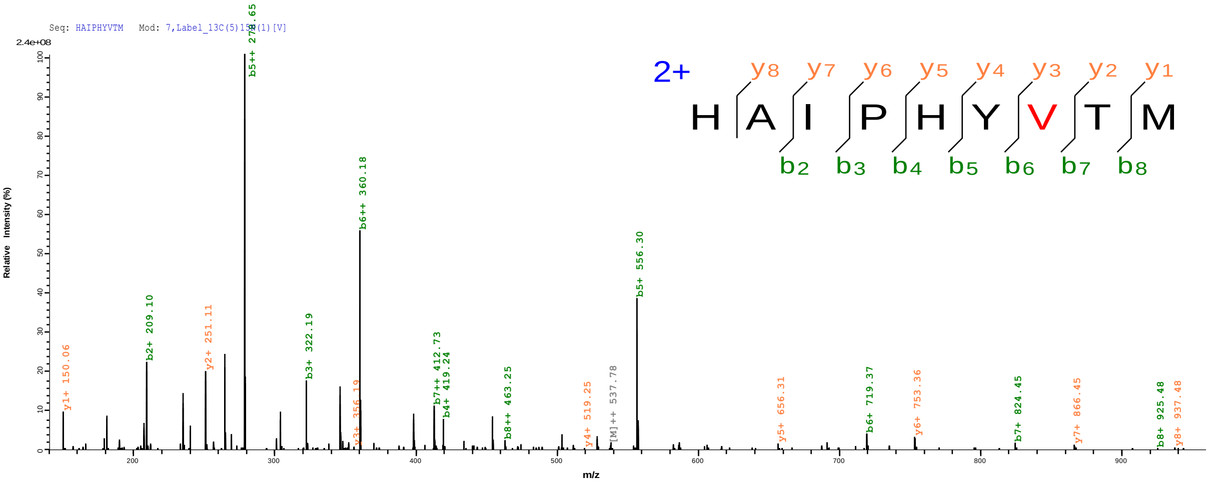

Seq: HAIPHYVTM Mod: 7,Label_13C(5)15N(1)[V]
2.4e+08
Relative Intensity (%)
m/z
2+
H A I P H Y V T M
y8 y7 y6 y5 y4 y3 y2 y1
b2 b3 b4 b5 b6 b7 b8
b5++ 278.65
b6++ 360.18
b5+ 556.30
b2+ 209.10
y2+ 251.11
b3+ 322.19
b7++ 412.73
b4+ 419.24
b8++ 463.25
y4+ 519.25
[M]++ 537.78
y5+ 656.31
b6+ 719.37
y6+ 753.36
b7+ 824.45
y7+ 866.45
b8+ 925.48
y8+ 937.48
y1+ 150.06

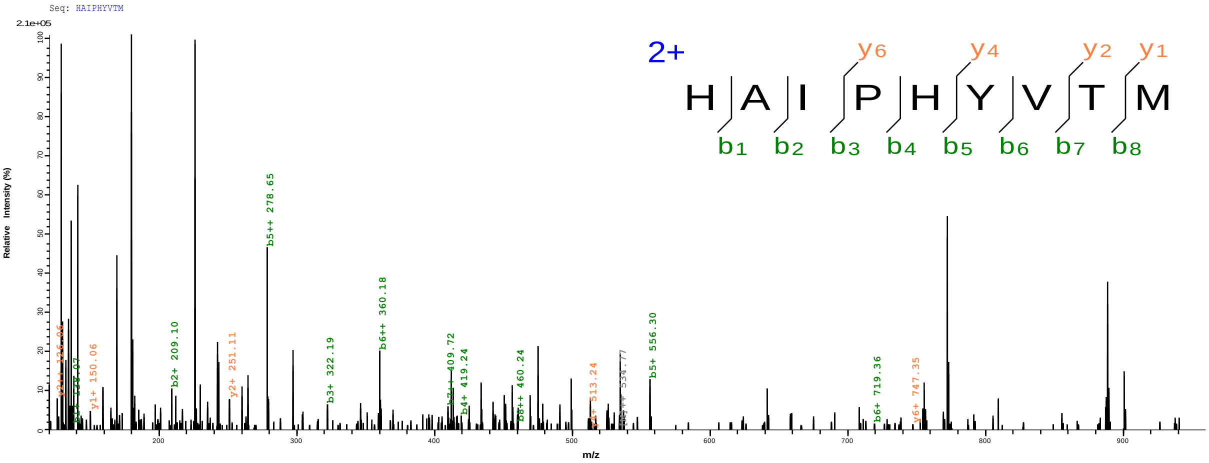

Seq: HAIPHYVTM
2.1e+05
Relative Intensity (%)
m/z
2+
H A I P H Y V T M
y6 y4 y2 y1
b1 b2 b3 b4 b5 b6 b7 b8
b5++ 278.65
b6++ 360.18
b5+ 556.30
b2+ 209.10
y2+ 251.11
b3+ 322.19
b4+ 419.24
460.24
513.24
534.77
b6+ 719.36
y6+ 747.35
y1+ 150.06

QTDINGGSLK

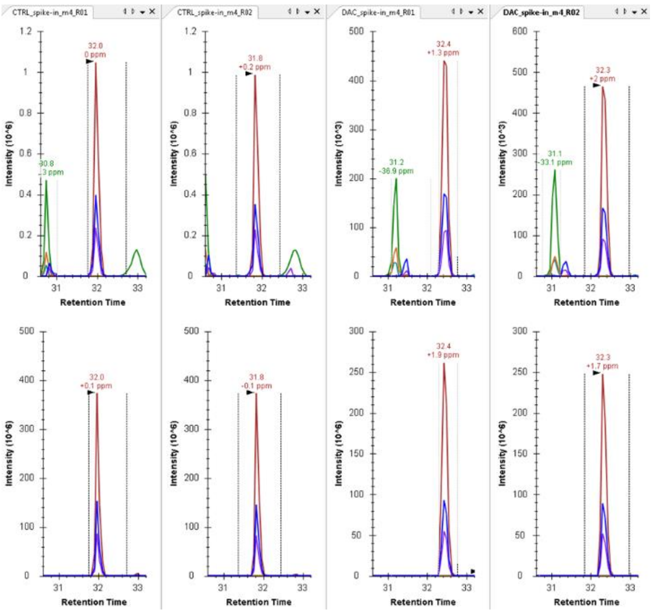

CTRL_spike-in_m4_R01
CTRL_spike-in_m4_R02
DAC_spike-in_m4_R01
DAC_spike-in_m4_R02
Intensity (10^6)
Intensity (10^3)
Retention Time
32.0
0 ppm
31.8
+0.2 ppm
32.4
+1.3 ppm
32.3
+2 ppm
31.2
-36.9 ppm
31.1
-33.1 ppm
32.0
+0.1 ppm
31.8
-0.1 ppm
32.4
+1.9 ppm
32.3
+1.7 ppm

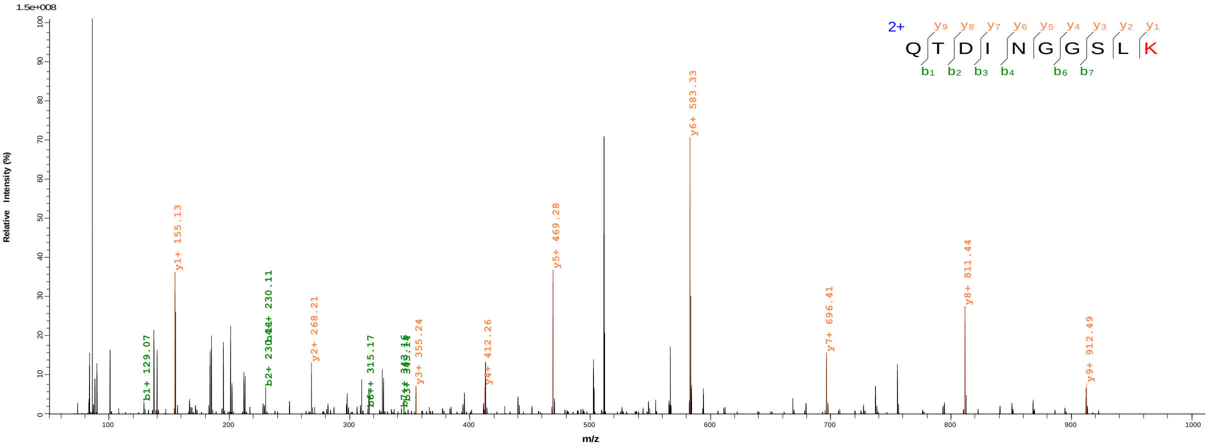

1.5e+008
Relative Intensity (%)
m/z
2+
Q T D I N G G S L K
y1+ 155.13
b1+ 129.07
b2+ 230.11
y2+ 268.21
b3+ 315.17
y3+ 355.24
y4+ 412.26
y5+ 469.28
y6+ 583.33
y7+ 696.41
y8+ 811.44
y9+ 912.49

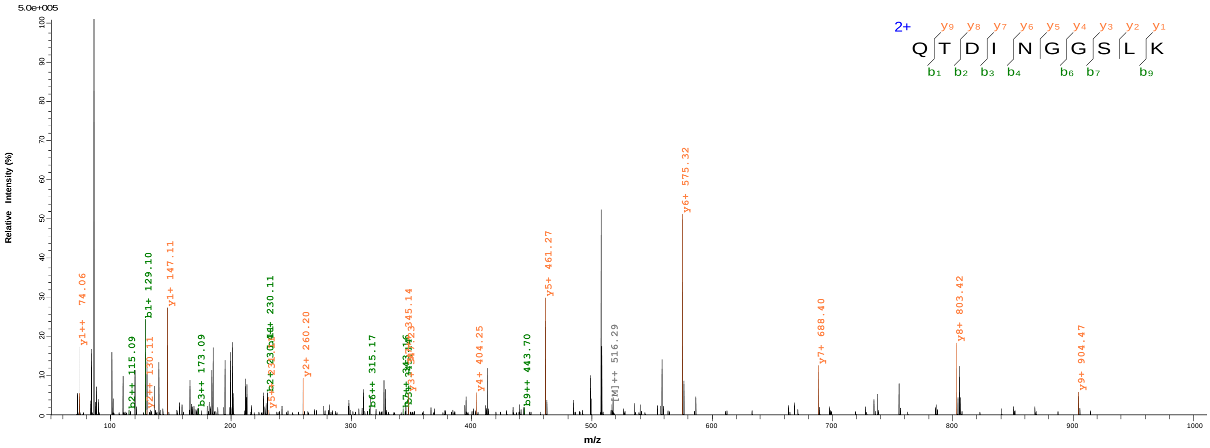

5.0e+005
Relative Intensity (%)
m/z
2+
Q T D I N G G S L K
y1++ 74.06
b2++ 115.09
b1+ 129.10
y1+ 147.11
b3++ 173.09
b2+ 230.11
y2+ 260.20
b3+ 315.17
y3+ 345.14
y4+ 404.25
b9++ 443.70
y5+ 461.27
[M]++ 516.29
y6+ 575.32
y7+ 688.40
y8+ 803.42
y9+ 904.47

RFTDVRVQF

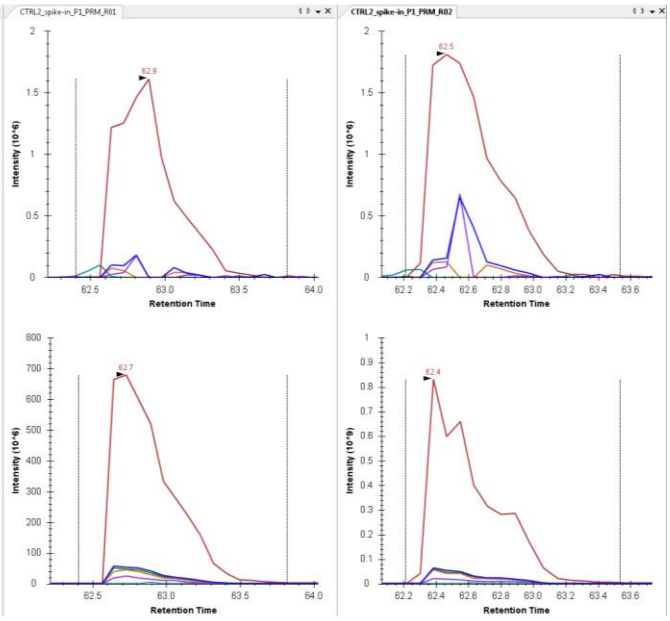

CTRL2_spike-in_P1_PRM_R01
CTRL2_spike-in_P1_PRM_R02
Intensity (10^6)
Intensity (10^9)
Retention Time
62.9
62.5
62.7
62.4

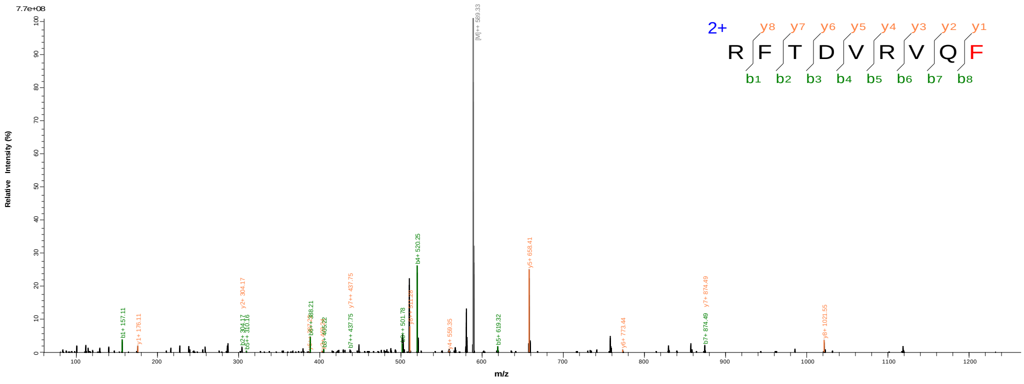

7.7e+08
Relative Intensity (%)
m/z
2+
y8 y7 y6 y5 y4 y3 y2 y1
R F T D V R V Q F
b1 b2 b3 b4 b5 b6 b7 b8

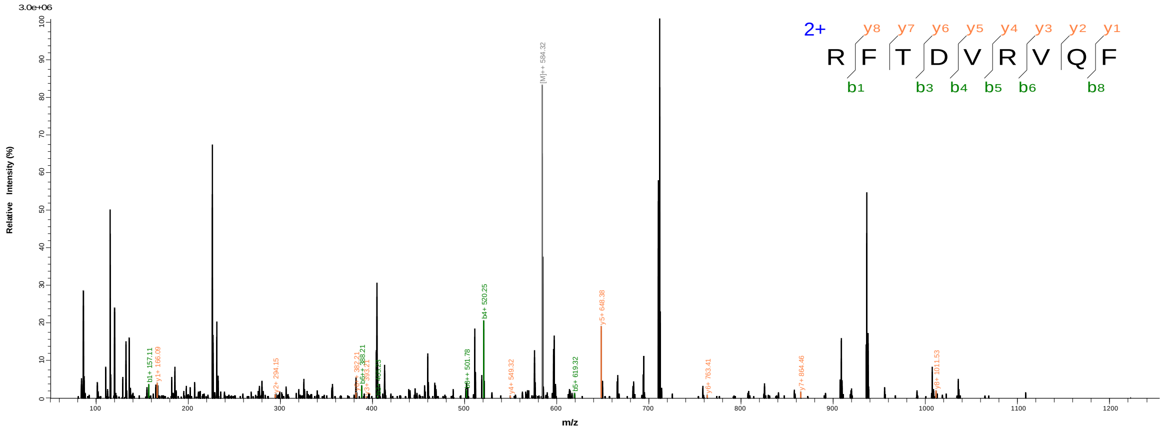

3.0e+06
Relative Intensity (%)
m/z
2+
y8 y7 y6 y5 y4 y3 y2 y1
R F T D V R V Q F
b1 b3 b4 b5 b6 b8

SPRKEIQWESL

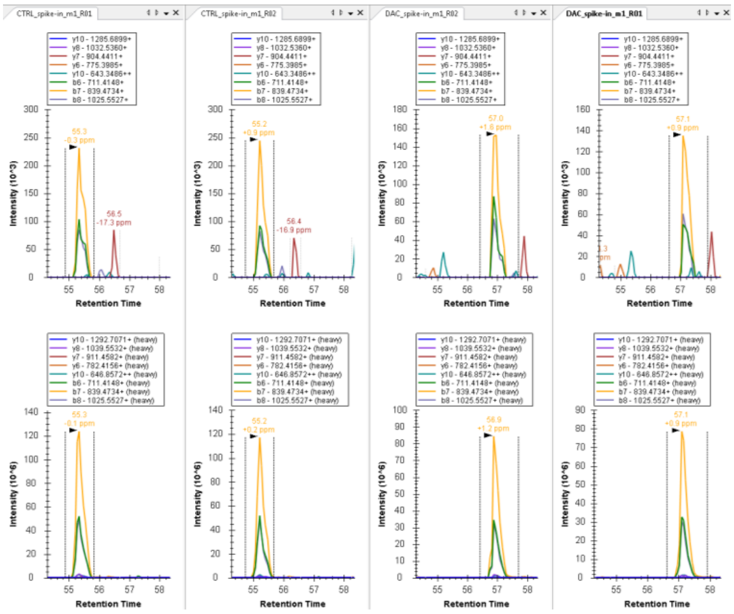

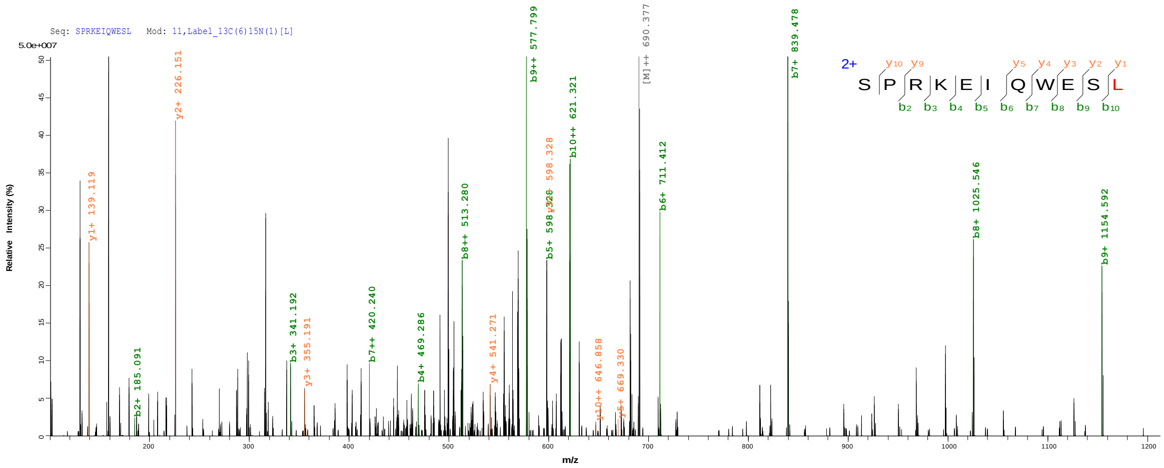

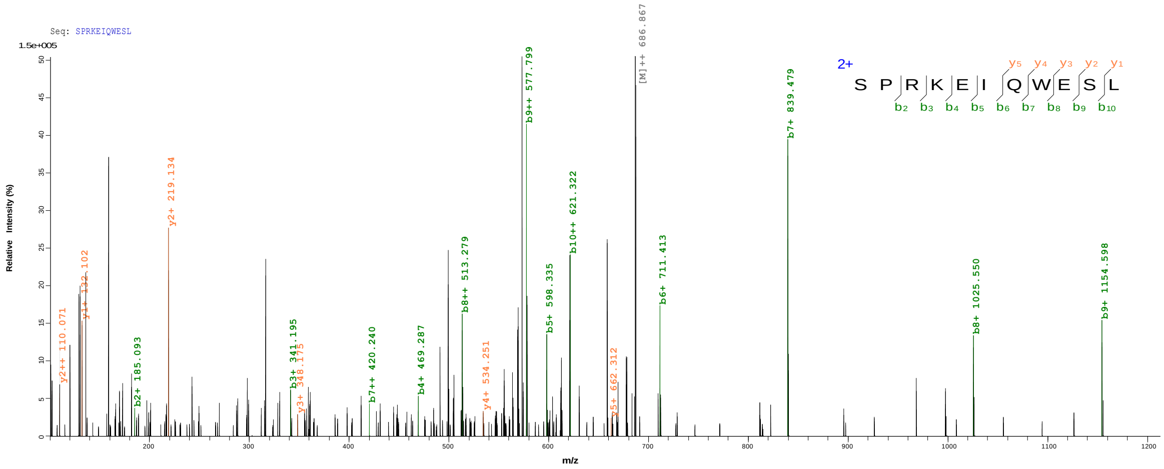

SPQPRNIGL

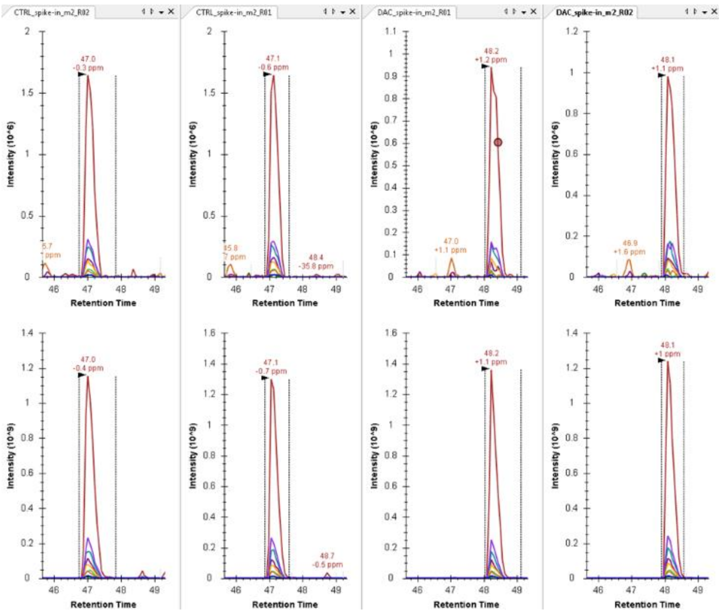

CTRL_spike-in_m2_R02
CTRL_spike-in_m2_R01
DAC_spike-in_m2_R01
DAC_spike-in_m2_R02
Intensity (10^6)
Intensity (10^9)
Retention Time

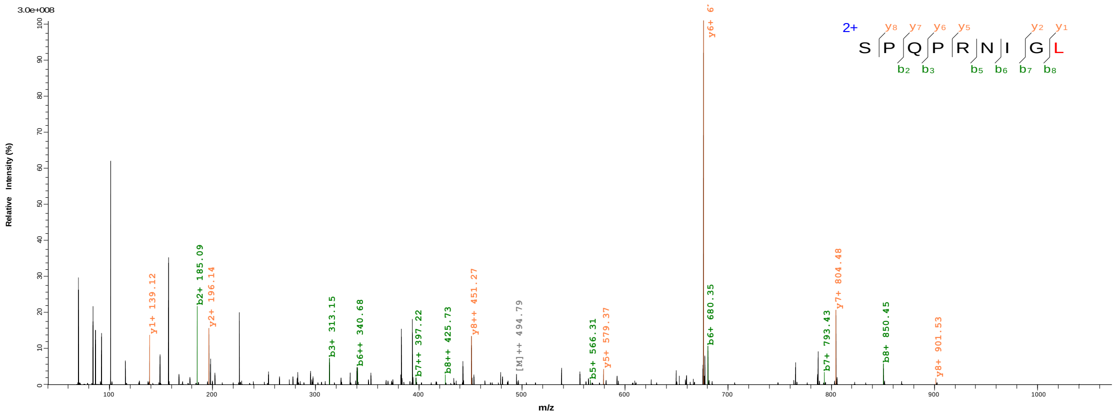

3.0e+008
Relative Intensity (%)
m/z
2+
S P Q P R N I G L
y1+ 139.12
b2+ 185.09
y2+ 196.14
b3+ 313.15
b6++ 340.68
b7++ 397.22
b8++ 425.73
y8++ 451.27
[M]++ 494.79
b5+ 566.31
y5+ 579.37
b6+ 680.35
b7+ 793.43
y7+ 804.48
b8+ 850.45
y8+ 901.53

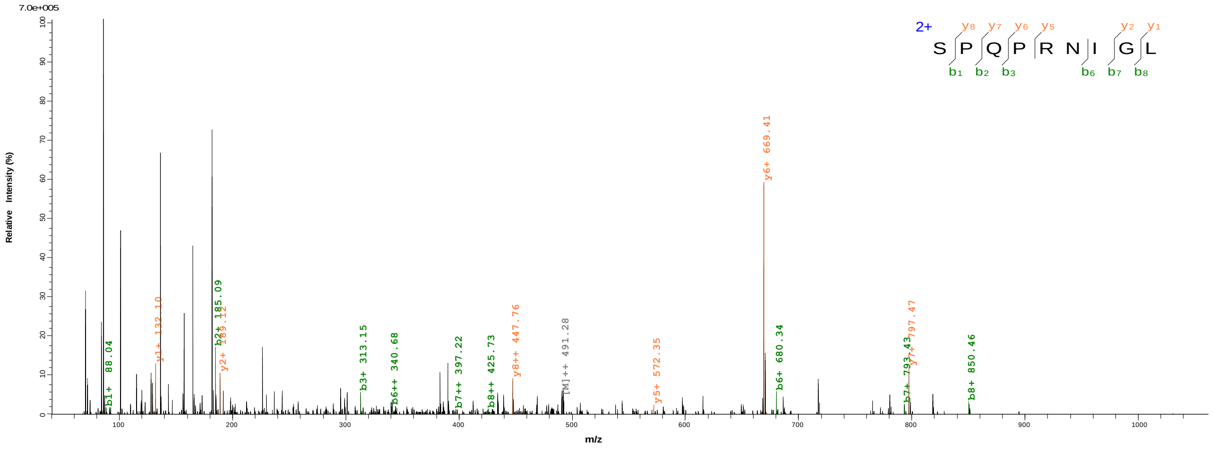

7.0e+005
Relative Intensity (%)
m/z
2+
S P Q P R N I G L
b1+ 88.04
b2+ 185.09
b3+ 313.15
b6++ 340.68
b7++ 397.22
b8++ 425.73
y8++ 447.76
[M]++ 491.28
y5+ 572.35
y6+ 669.41
b6+ 680.34
y7+ 797.47
b8+ 850.46

GQYGQSAFHY

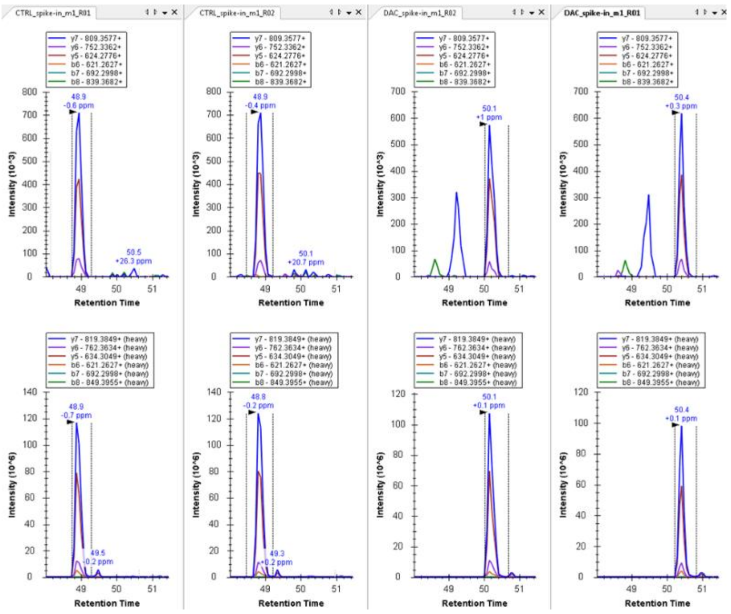

CTRL_spike-in_m1_R01
CTRL_spike-in_m1_R02
DAC_spike-in_m1_R02
DAC_spike-in_m1_R01
y7 - 809.3577+
y6 - 752.3362+
y5 - 624.2776+
b6 - 621.2627+
b7 - 692.2998+
b8 - 839.3682+
y7 - 819.3849+ (heavy)
y6 - 762.3634+ (heavy)
y5 - 634.3049+ (heavy)
b6 - 621.2627+ (heavy)
b7 - 692.2998+ (heavy)
b8 - 849.3955+ (heavy)
Intensity (10^3)
Intensity (10^6)
Retention Time

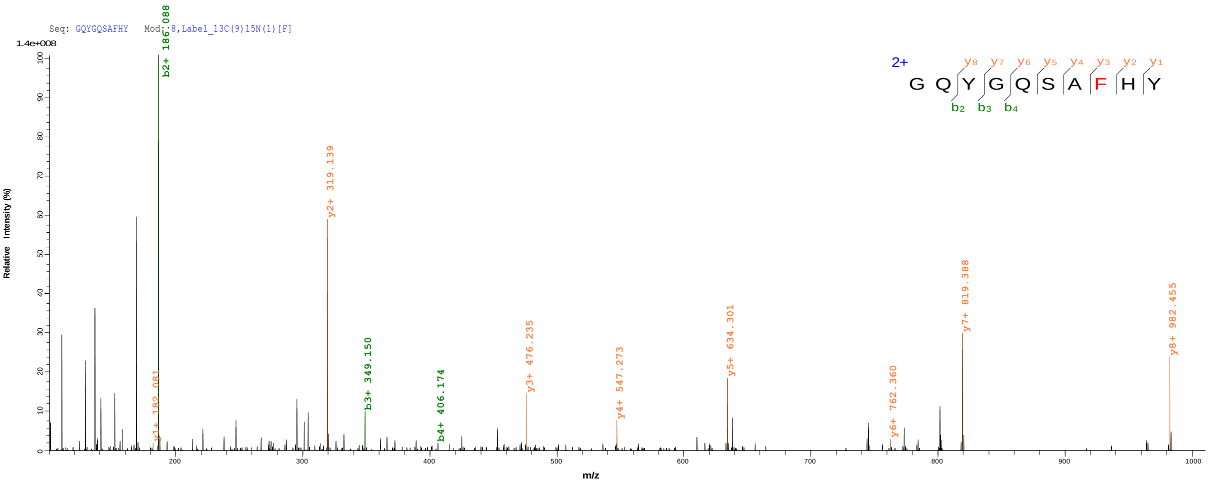

Seq: GQYGQSAFHY Mod: 8,Label_13C(9)15N(1)[F]
1.4e+008
Relative Intensity (%)
m/z
b2+ 186.088
y2+ 319.139
b3+ 349.150
b4+ 406.174
y3+ 476.235
y4+ 547.273
y5+ 634.301
y6+ 762.360
y7+ 819.388
y8+ 982.455
2+
G Q Y G Q S A F H Y

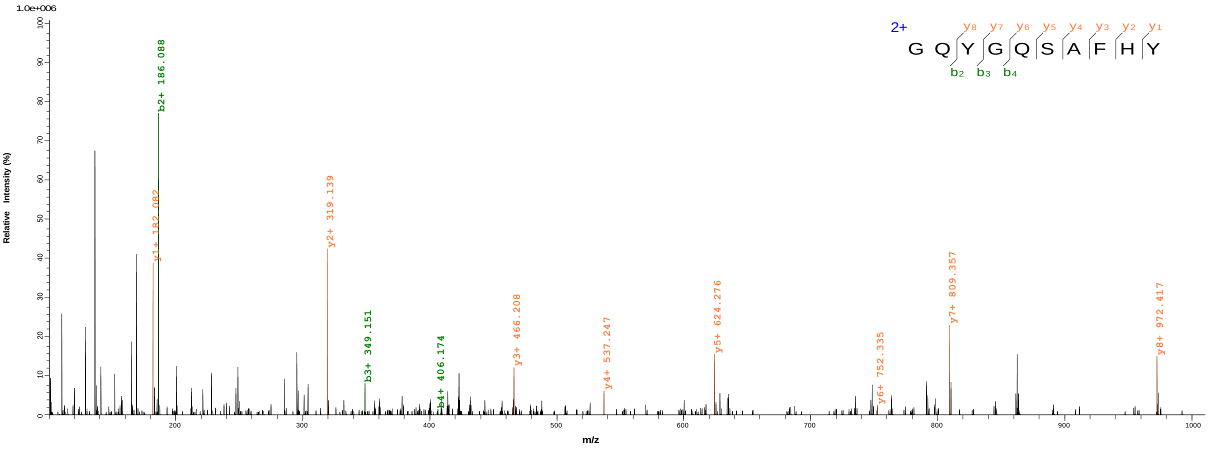

1.0e+006
Relative Intensity (%)
m/z
b2+ 186.088
y1+ 182.082
y2+ 319.139
b3+ 349.151
b4+ 406.174
y3+ 466.208
y4+ 537.247
y5+ 624.276
y6+ 752.335
y7+ 809.357
y8+ 972.417
2+
G Q Y G Q S A F H Y

HQILKGGSGTY

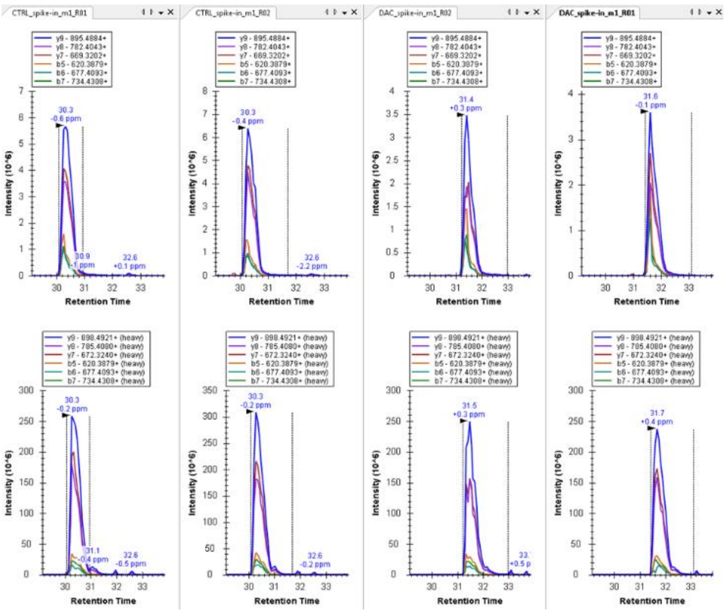

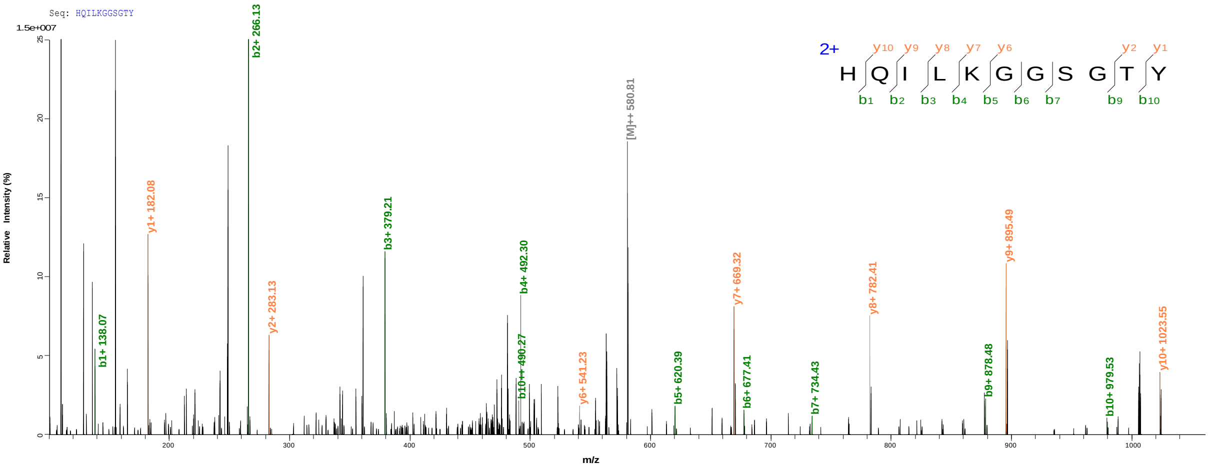

LYPEWTEAQRL

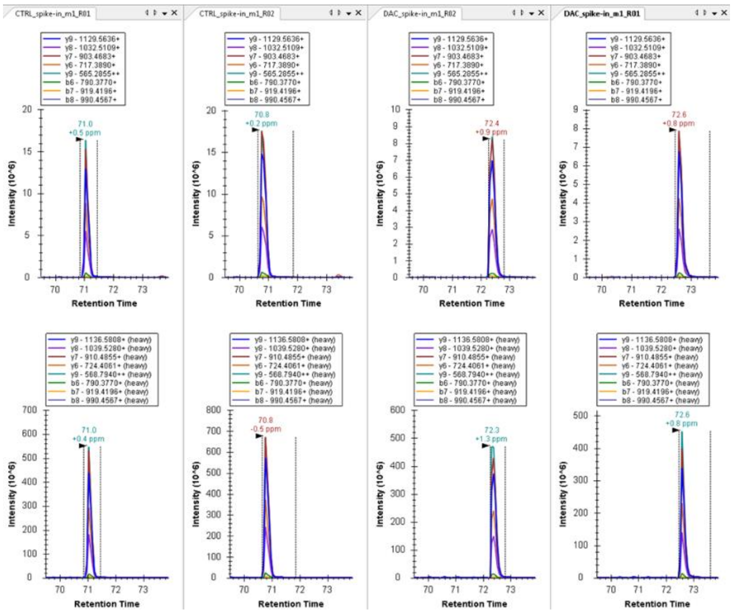

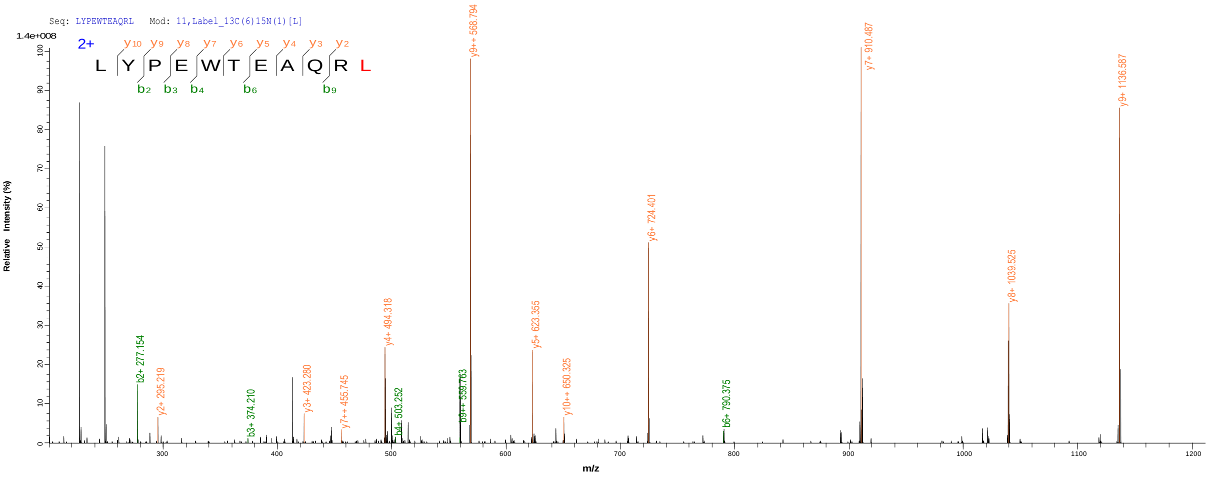

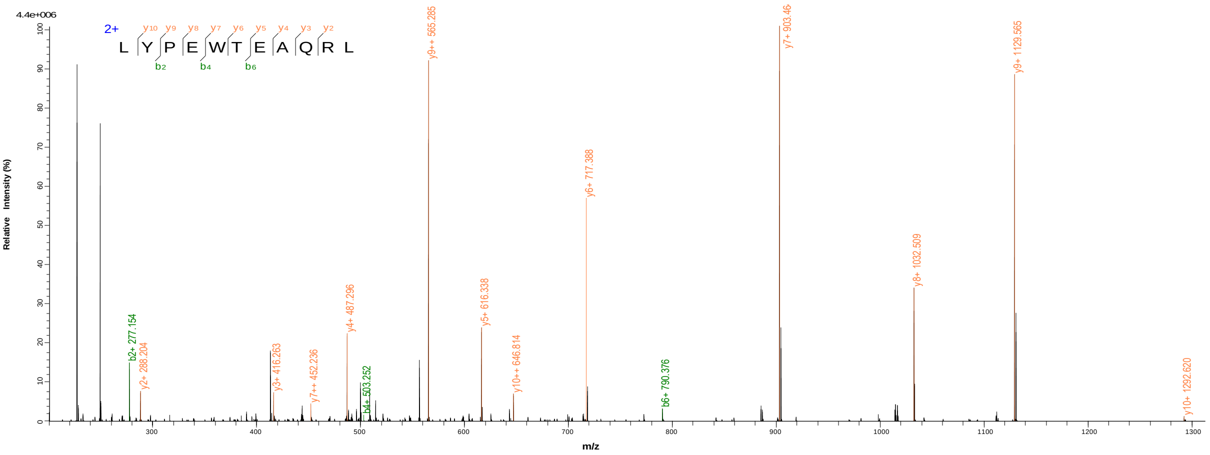

PIGENSPL

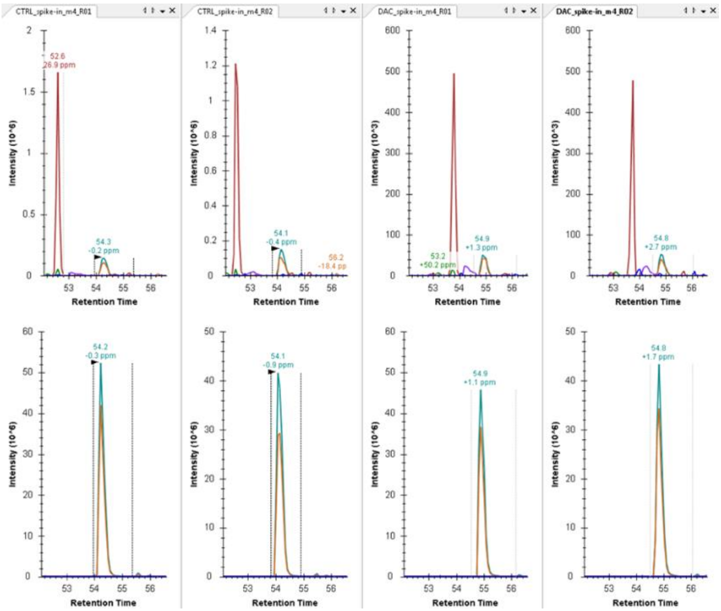
CTRL_spike-in_m4_R01
CTRL_spike-in_m4_R02
DAC_spike-in_m4_R01
DAC_spike-in_m4_R02
Intensity (10^6)
Intensity (10^3)
Retention Time

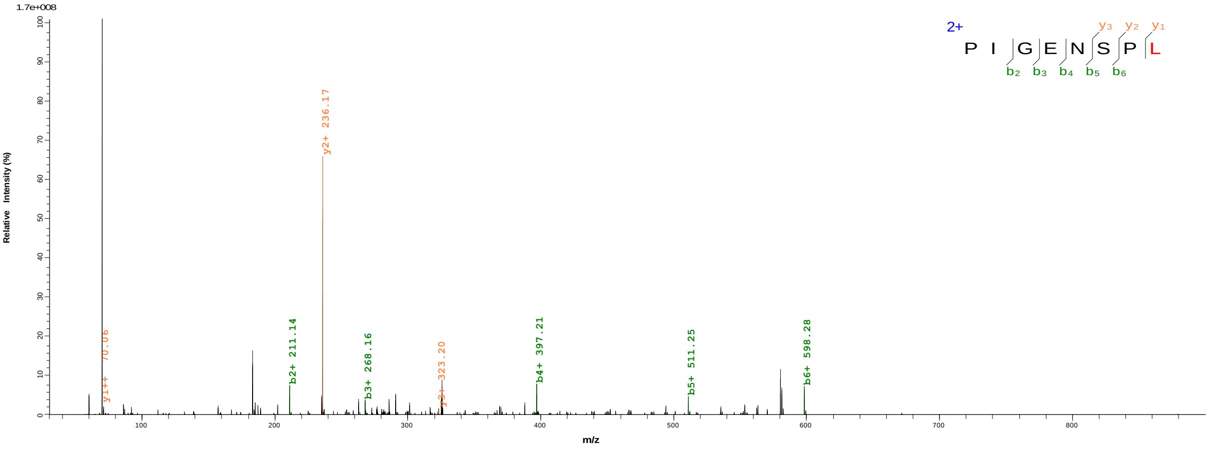
1.7e+008
2+
P I G E N S P L
y3 y2 y1
b2 b3 b4 b5 b6
Relative Intensity (%)
y1++ 70.06
b2+ 211.14
y2+ 236.17
b3+ 268.16
323.20
b4+ 397.21
b5+ 511.25
b6+ 598.28
m/z

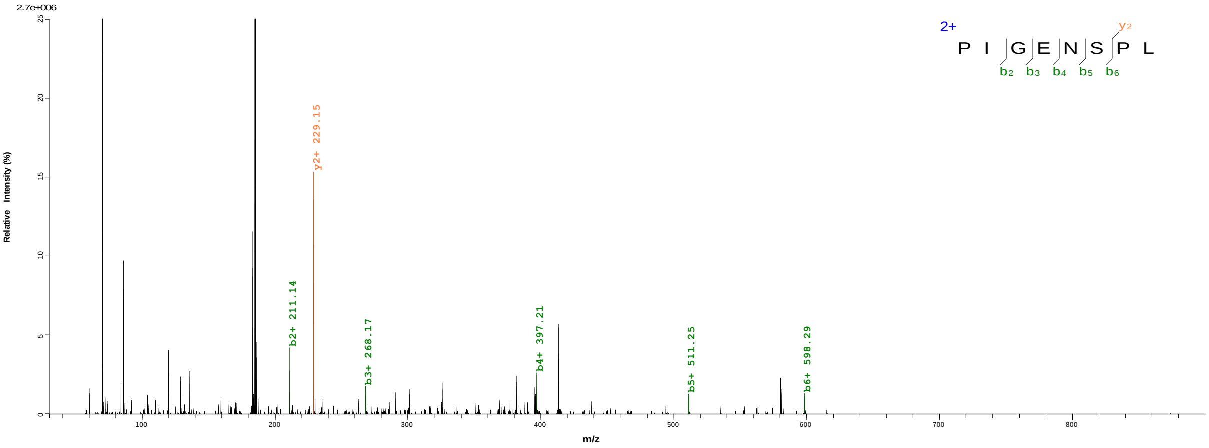
2.7e+006
2+
P I G E N S P L
y2
b2 b3 b4 b5 b6
Relative Intensity (%)
b2+ 211.14
y2+ 229.15
b3+ 268.17
b4+ 397.21
b5+ 511.25
b6+ 598.29
m/z

LPQETRIPL

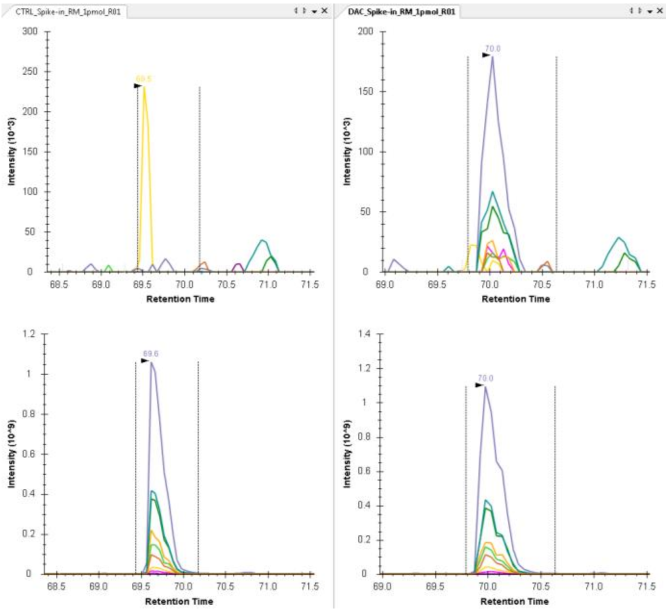

CTRL_Spike-in_RM_1pmol_R01
DAC_Spike-in_RM_1pmol_R01
Intensity (10^3)
Intensity (10^9)
Retention Time

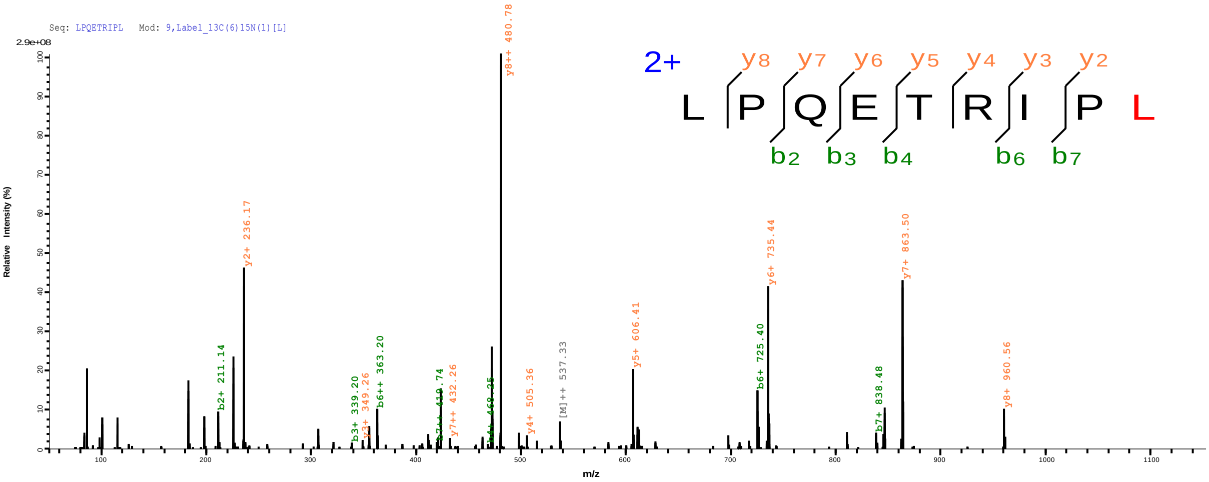

Seq: LPQETRIPL Mod: 9,Label_13C(6)15N(1)[L]
2.9e+08
Relative Intensity (%)
m/z
y8++ 480.78
y2+ 236.17
y6+ 735.44
y7+ 863.50
y5+ 606.41
b6+ 725.40
b2+ 211.14
b6++ 363.20
b3+ 339.20
y4+ 505.36
[M]++ 537.33
b7+ 838.48
y8+ 960.56
y7++ 432.26
2+
y8 y7 y6 y5 y4 y3 y2
L P Q E T R I P L
b2 b3 b4 b6 b7

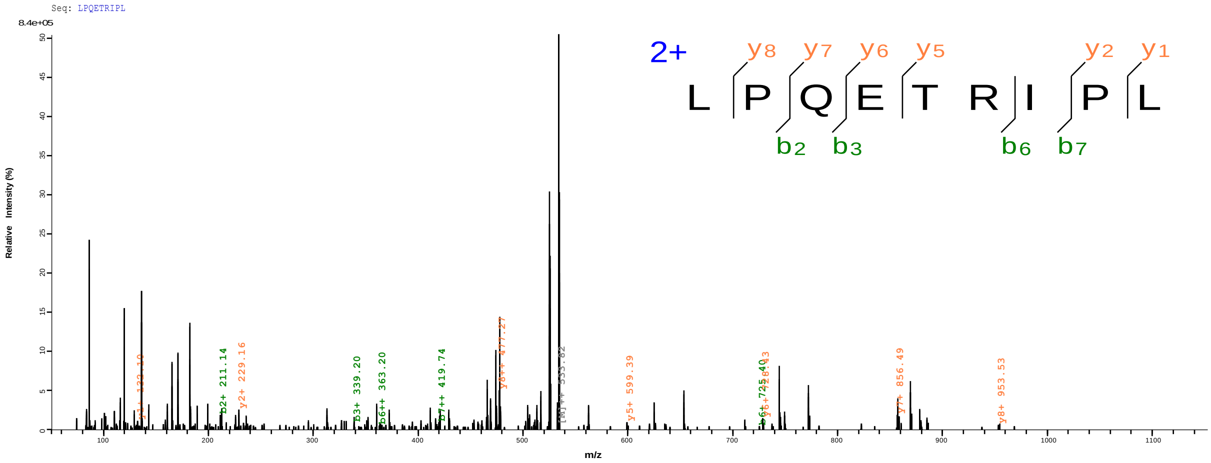

Seq: LPQETRIPL
8.4e+05
Relative Intensity (%)
m/z
b2+ 211.14
y2+ 229.16
b3+ 339.20
b6++ 363.20
b7++ 419.74
y5+ 599.39
y7+ 856.49
y8+ 953.53
2+
y8 y7 y6 y5 y2 y1
L P Q E T R I P L
b2 b3 b6 b7

GTAAIQAHY

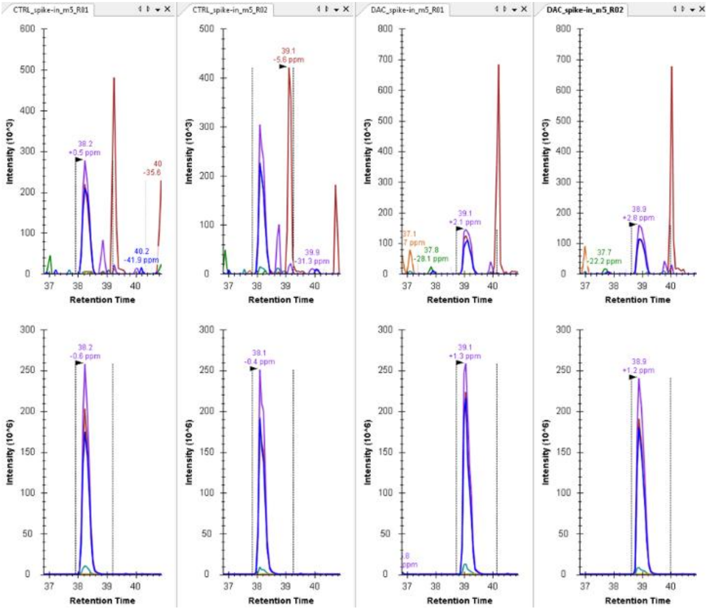
CTRL_spike-in_m5_R01
CTRL_spike-in_m5_R02
DAC_spike-in_m5_R01
DAC_spike-in_m5_R02
Intensity (10^3)
Intensity (10^6)
Retention Time

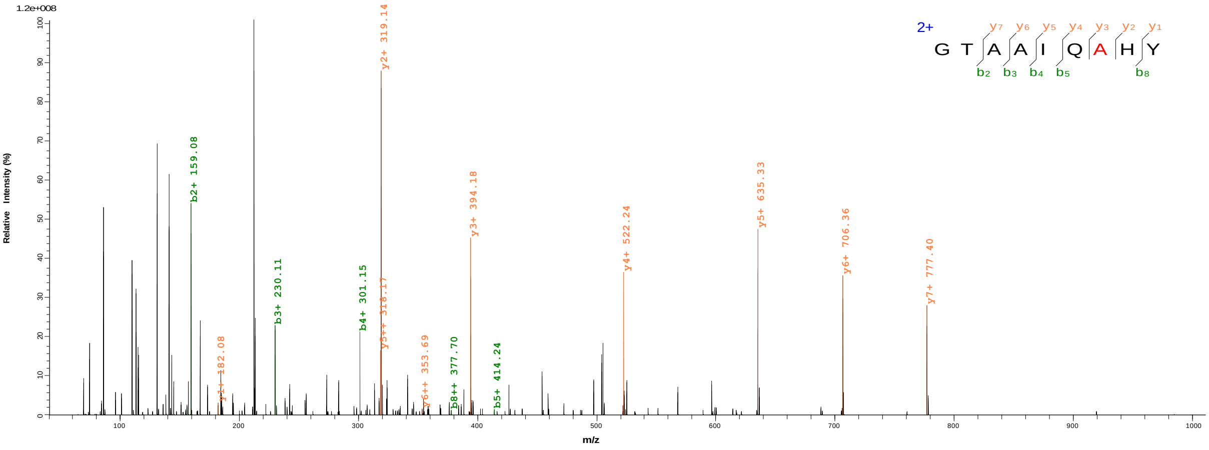
1.2e+008
Relative Intensity (%)
m/z
2+
G T A A I Q A H Y
y7 y6 y5 y4 y3 y2 y1
b2 b3 b4 b5 b8
y2+ 319.14
b2+ 159.08
y3+ 394.18
y5+ 635.33
y4+ 522.24
y6+ 706.36
y7+ 777.40
b3+ 230.11
b4+ 301.15
y1+ 182.08
y6++ 353.69
b8++ 377.70
b5+ 414.24

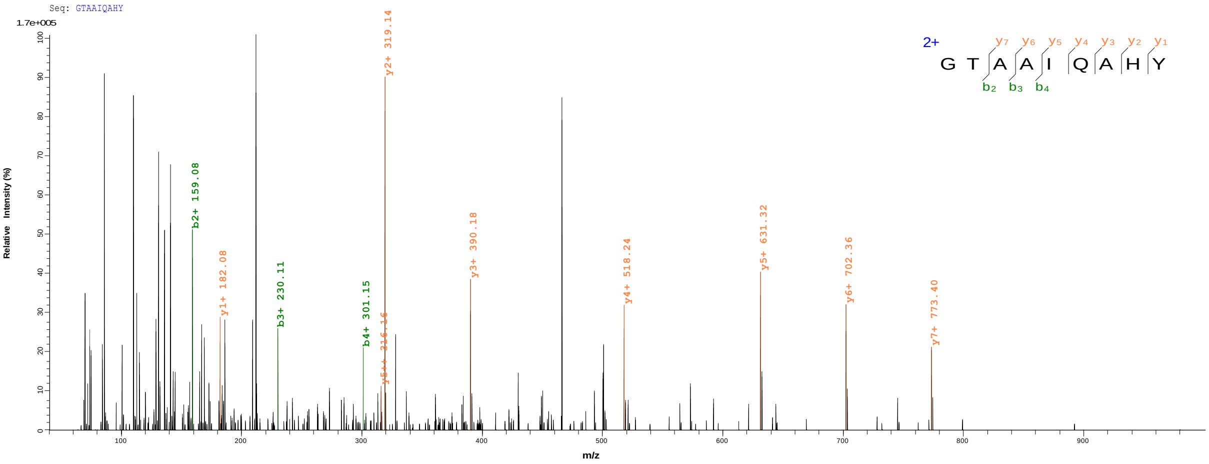
Seq: GTAAIQAHY
1.7e+005
Relative Intensity (%)
m/z
2+
G T A A I Q A H Y
y7 y6 y5 y4 y3 y2 y1
b2 b3 b4
y2+ 319.14
b2+ 159.08
y3+ 390.18
y5+ 631.32
y4+ 518.24
y6+ 702.36
y7+ 773.40
y1+ 182.08
b3+ 230.11
b4+ 301.15

HYGSAFPSI

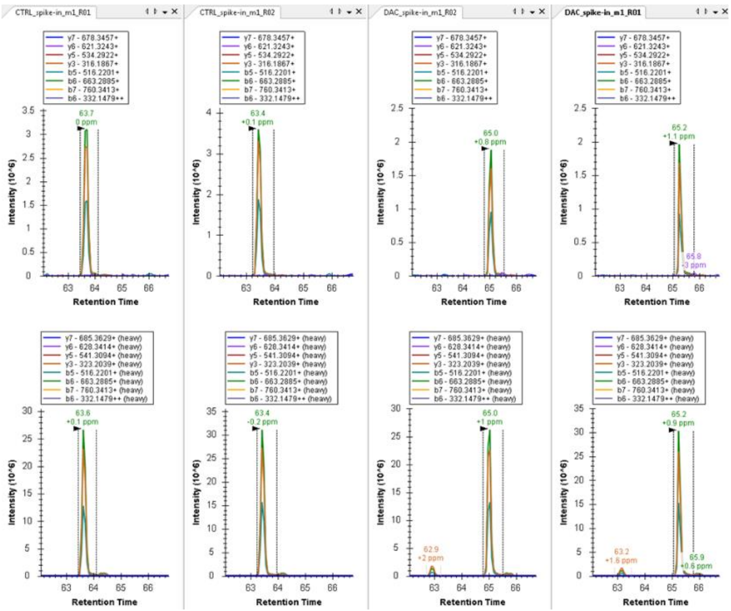
CTRL_spike-in_m1_R01
CTRL_spike-in_m1_R02
DAC_spike-in_m1_R02
DAC_spike-in_m1_R01
Intensity (10^6)
Retention Time

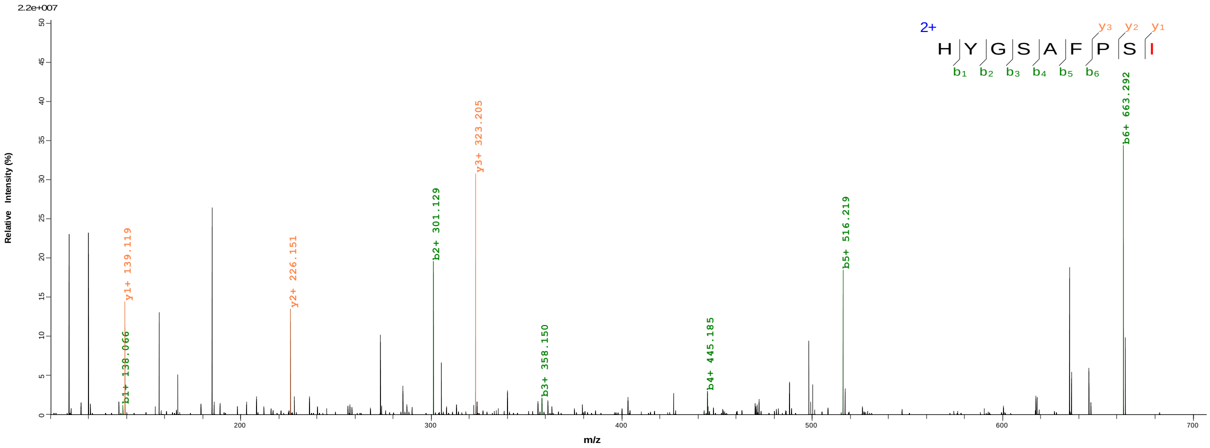
2.2e+007
2+
H Y G S A F P S I
y3 y2 y1
b1 b2 b3 b4 b5 b6
b6+ 663.292
y3+ 323.205
b5+ 516.219
b2+ 301.129
y1+ 139.119
y2+ 226.151
b3+ 358.150
b4+ 445.185
b1+ 138.066
Relative Intensity (%)
m/z

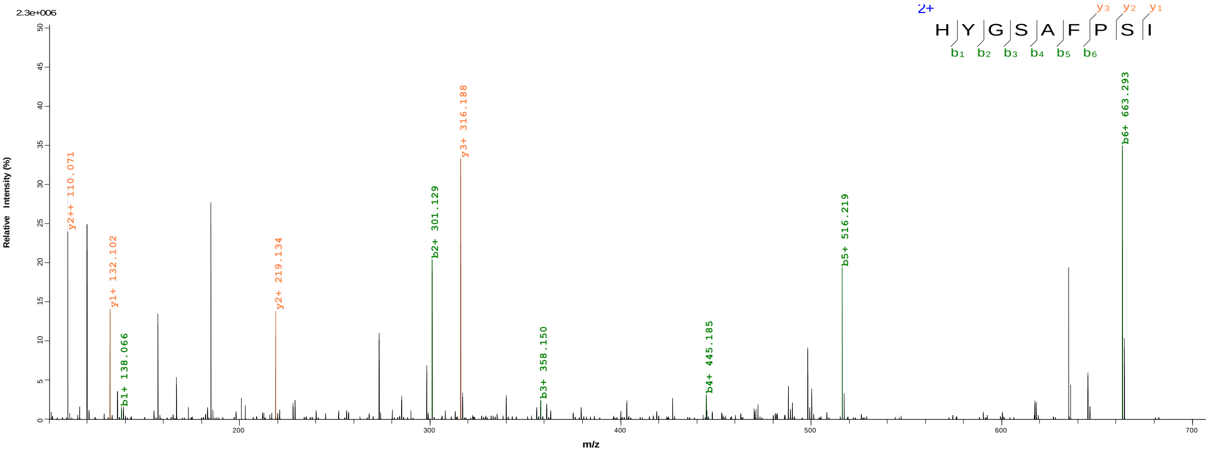
2.3e+006
2+
H Y G S A F P S I
y3 y2 y1
b1 b2 b3 b4 b5 b6
b6+ 663.293
y3+ 316.188
y2++ 110.071
b2+ 301.129
b5+ 516.219
y1+ 132.102
y2+ 219.134
b1+ 138.066
b3+ 358.150
b4+ 445.185
Relative Intensity (%)
m/z

ISKPPGVAL

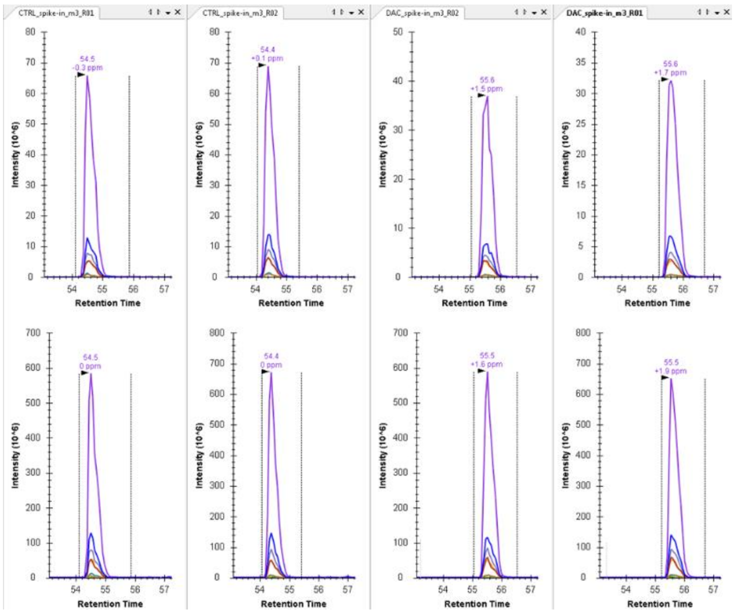
CTRL_spike-in_m3_R01
CTRL_spike-in_m3_R02
DAC_spike-in_m3_R02
DAC_spike-in_m3_R01
Intensity (10^6)
Retention Time
54.5 -0.3 ppm
54.4 +0.1 ppm
55.6 +1.5 ppm
55.6 +1.7 ppm
54.5 0 ppm
54.4 0 ppm
55.5 +1.6 ppm
55.5 +1.9 ppm

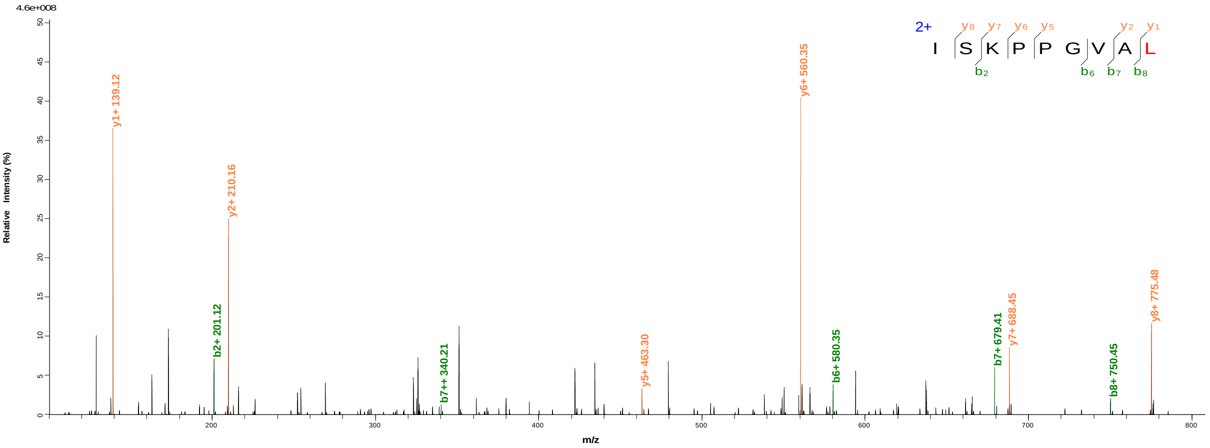
4.6e+008
Relative Intensity (%)
m/z
2+
I S K P P G V A L
y8 y7 y6 y5 y2 y1
b2 b6 b7 b8
y1+ 139.12
y2+ 210.16
b2+ 201.12
b7++ 340.21
y5+ 463.30
y6+ 560.35
b6+ 580.35
b7+ 679.41
y7+ 688.45
b8+ 750.45
y8+ 775.48

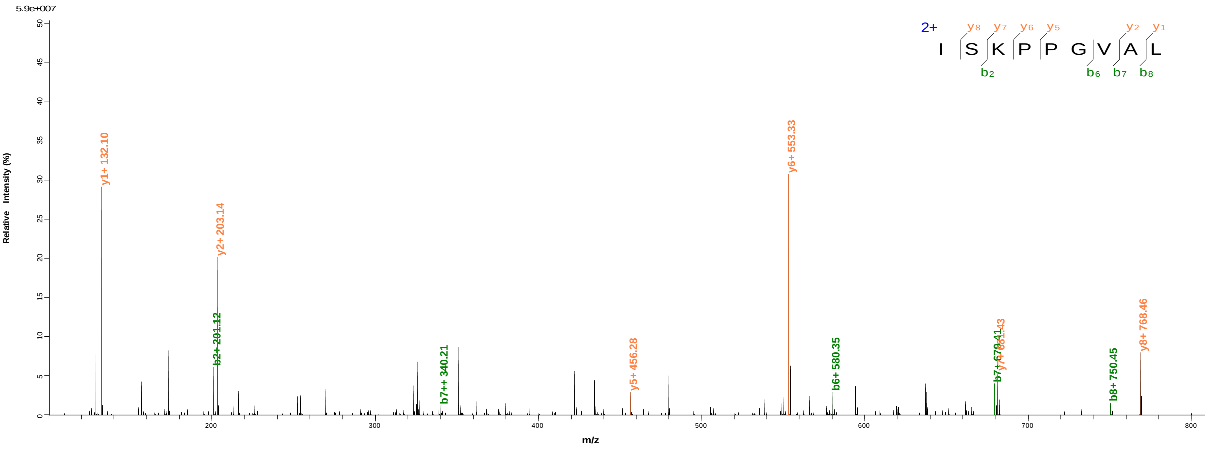
5.9e+007
Relative Intensity (%)
m/z
2+
I S K P P G V A L
y8 y7 y6 y5 y2 y1
b2 b6 b7 b8
y1+ 132.10
y2+ 203.14
b2+ 201.12
b7++ 340.21
y5+ 456.28
y6+ 553.33
b6+ 580.35
b7+ 679.41
y7+ 681.43
b8+ 750.45
y8+ 768.46

SPRSPSPSL

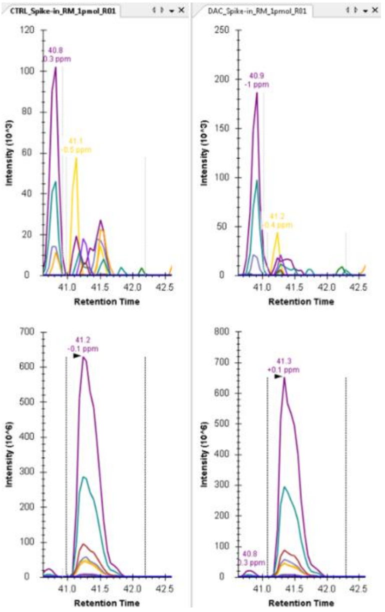
CTRL_Spike-in_RM_1pmol_R01
DAC_Spike-in_RM_1pmol_R01
Intensity (10^3)
Intensity (10^6)
Retention Time
40.8
0.3 ppm
41.1
-0.5 ppm
40.9
-1 ppm
41.2
-0.4 ppm
41.2
-0.1 ppm
41.3
+0.1 ppm
40.8
0.3 ppm

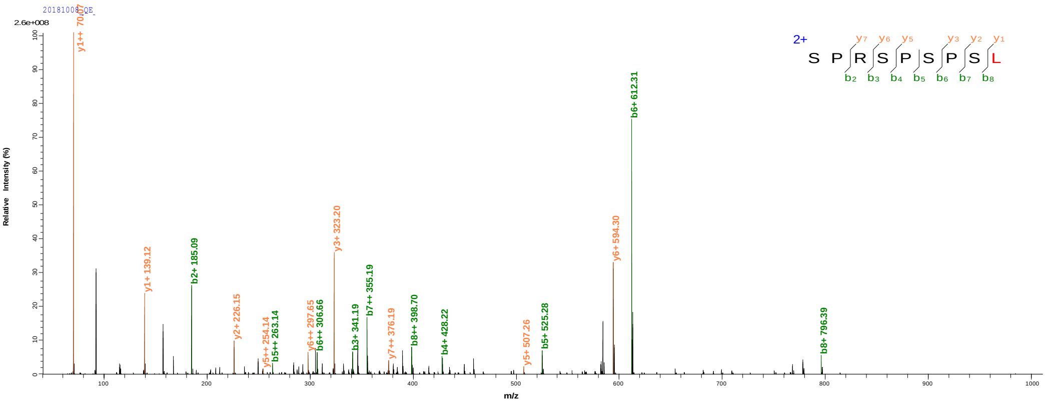
2.6e+008
Relative Intensity (%)
m/z
2+
S P R S P S P S L
y7 y6 y5 y3 y2 y1
b2 b3 b4 b5 b6 b7 b8
y1++ 70.07
y1+ 139.12
b2+ 185.09
y2+ 226.15
y5++ 254.14
b5++ 263.14
y6++ 297.65
b6++ 306.66
y3+ 323.20
b3+ 341.19
b7++ 355.19
y7++ 376.19
b8++ 398.70
b4+ 428.22
y5+ 507.26
b5+ 525.28
y6+ 594.30
b6+ 612.31
b8+ 796.39

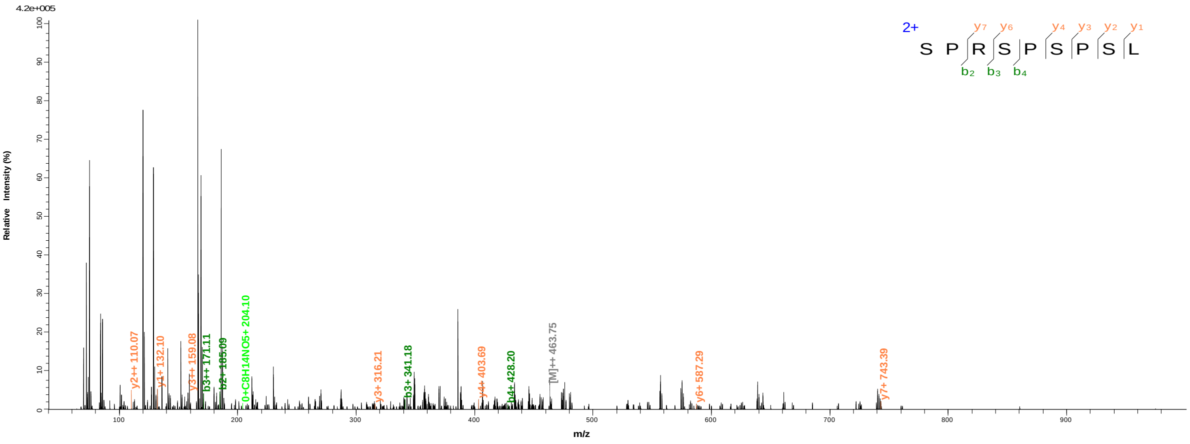
4.2e+005
Relative Intensity (%)
m/z
2+
S P R S P S P S L
y7 y6 y4 y3 y2 y1
b2 b3 b4
y2++ 110.07
y1+ 132.10
y3++ 159.08
b3++ 171.11
b2+ 185.09
y3+ 316.21
b3+ 341.18
y4+ 403.69
b4+ 428.20
[M]++ 463.75
y6+ 587.29
y7+ 743.39

YPHLHNAEL

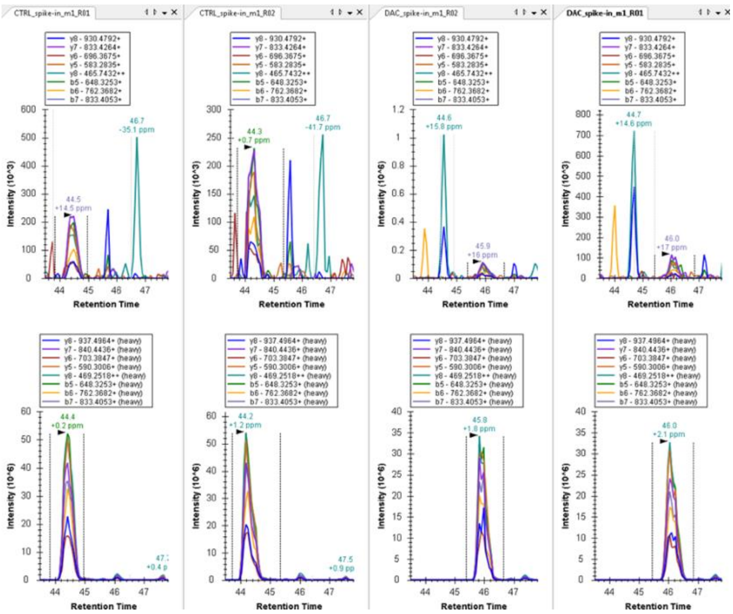

CTRL_spike-in_m1_R01
CTRL_spike-in_m1_R02
DAC_spike-in_m1_R02
DAC_spike-in_m1_R01
Retention Time

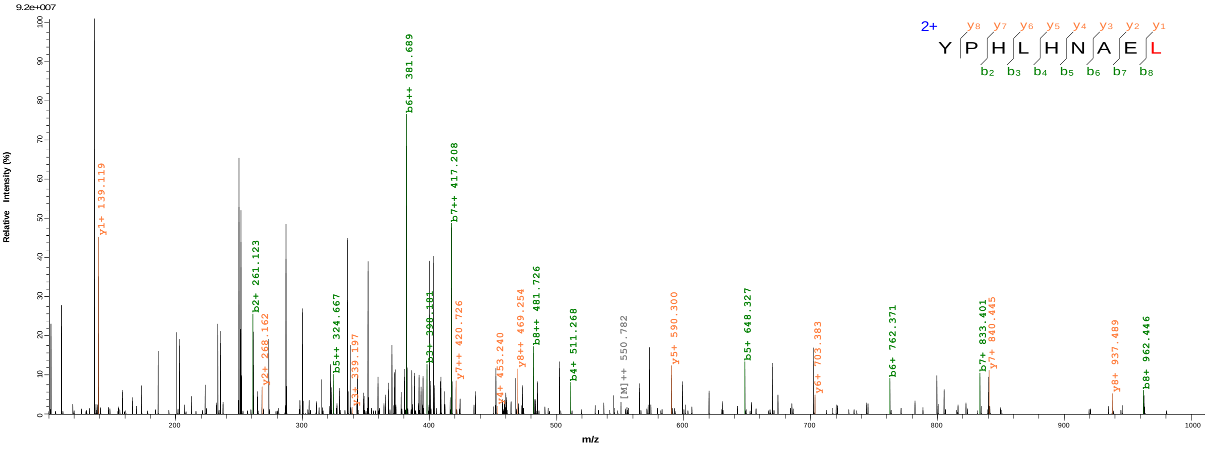

9.2e+007
Relative Intensity (%)
m/z
2+
Y P H L H N A E L
y8 y7 y6 y5 y4 y3 y2 y1
b2 b3 b4 b5 b6 b7 b8
y1+ 139.119
b2+ 261.123
y2+ 268.162
b5++ 324.667
y3+ 339.197
b6++ 381.689
b3+ 399.181
b7++ 417.208
y7++ 420.726
y4+ 453.240
y8++ 469.254
b8++ 481.726
b4+ 511.268
[M]++ 550.782
y5+ 590.300
b5+ 648.327
y6+ 703.383
b6+ 762.371
b7+ 833.401
y7+ 840.445
y8+ 937.489
b8+ 962.446

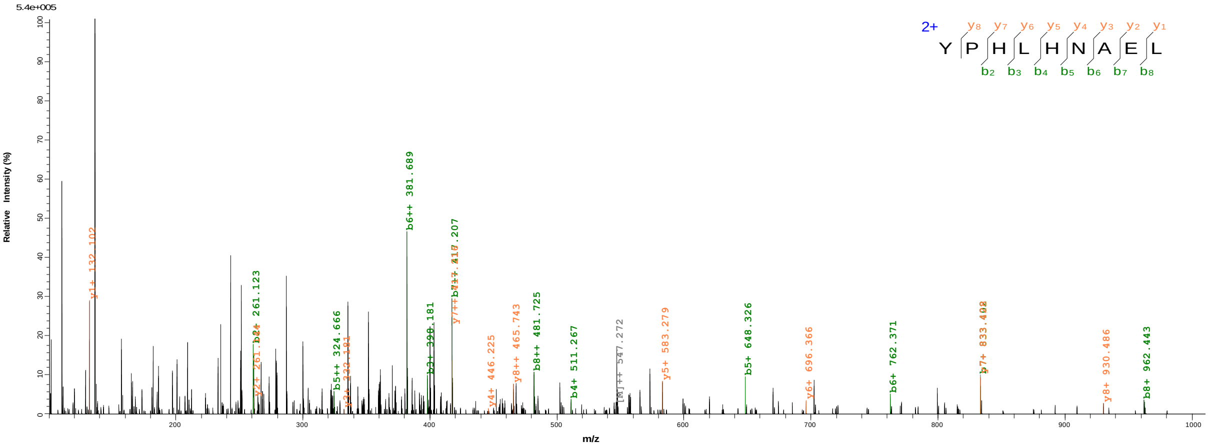

5.4e+005
Relative Intensity (%)
m/z
2+
Y P H L H N A E L
y8 y7 y6 y5 y4 y3 y2 y1
b2 b3 b4 b5 b6 b7 b8
y1+ 132.102
b2+ 261.123
b5++ 324.666
b6++ 381.689
b3+ 399.181
b7++ 417.207
y8++ 465.743
b8++ 481.725
b4+ 511.267
y4+ 446.225
[M]++ 547.272
y5+ 583.279
b5+ 648.326
y6+ 696.366
b6+ 762.371
b7+ 833.402
y8+ 930.486
b8+ 962.443

SYLEPQVTF

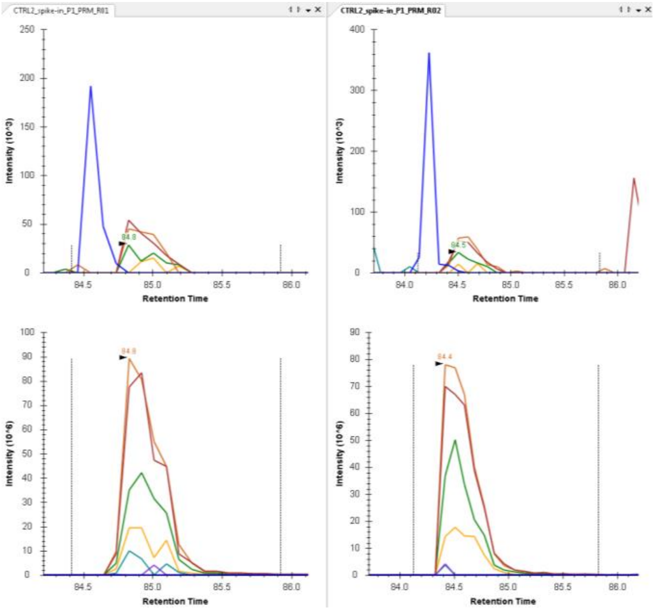

CTRL2_spike-in_P1_PRM_R01
CTRL2_spike-in_P1_PRM_R02
Intensity (10^3)
Intensity (10^6)
Retention Time

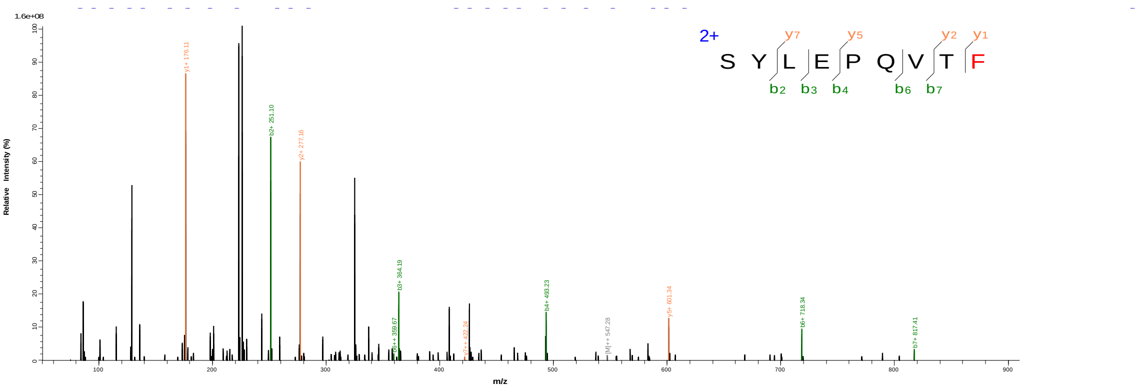

1.6e+08
Relative Intensity (%)
m/z
2+
S Y L E P Q V T F
y7 y5 y2 y1
b2 b3 b4 b6 b7

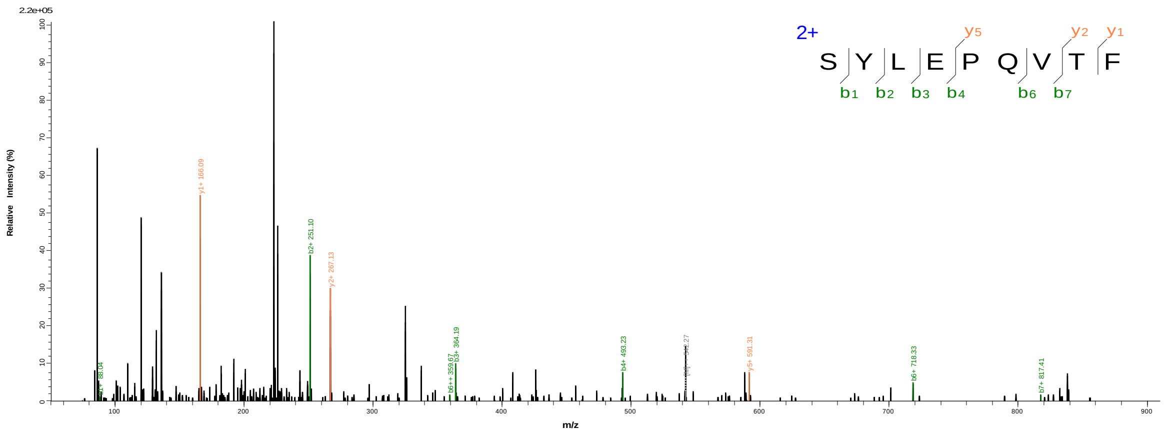

2.2e+05
Relative Intensity (%)
m/z
2+
S Y L E P Q V T F
y5 y2 y1
b1 b2 b3 b4 b6 b7

YTDQPSTSQIAY

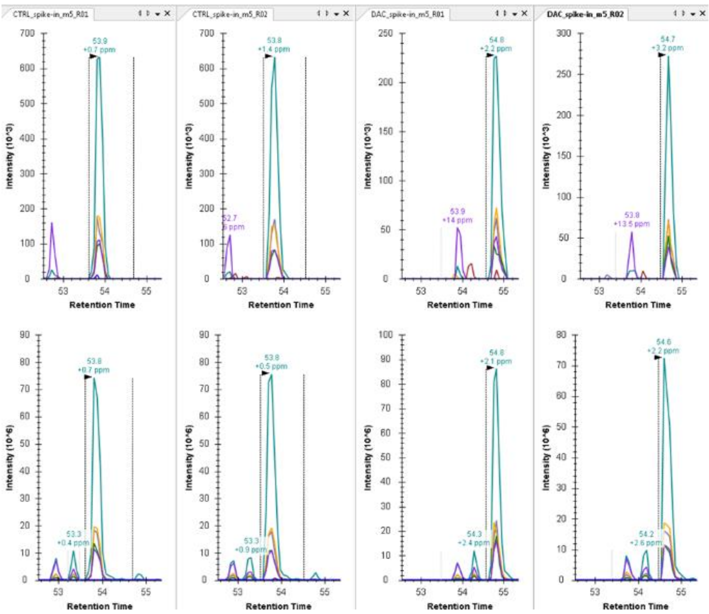

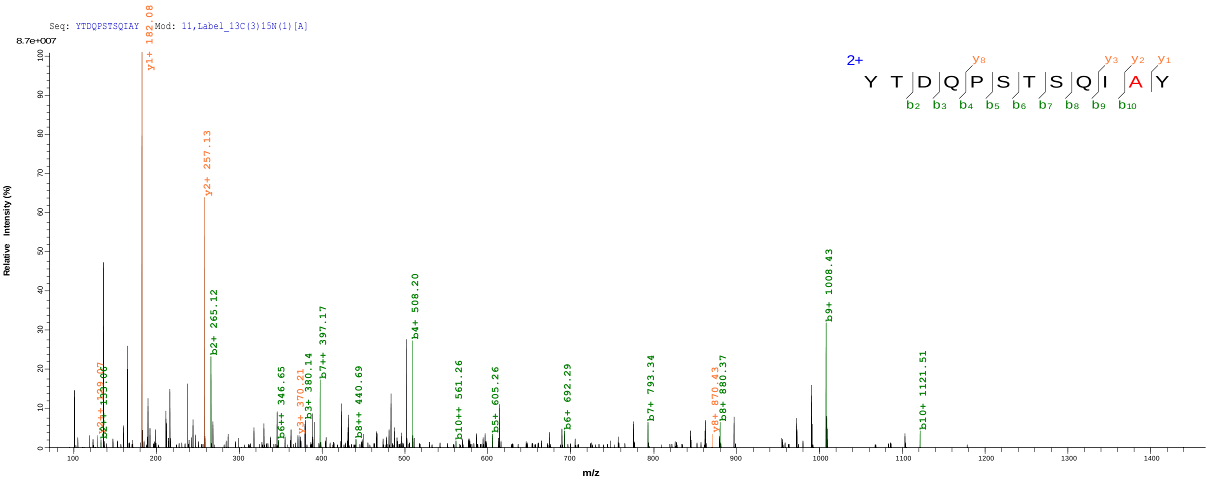

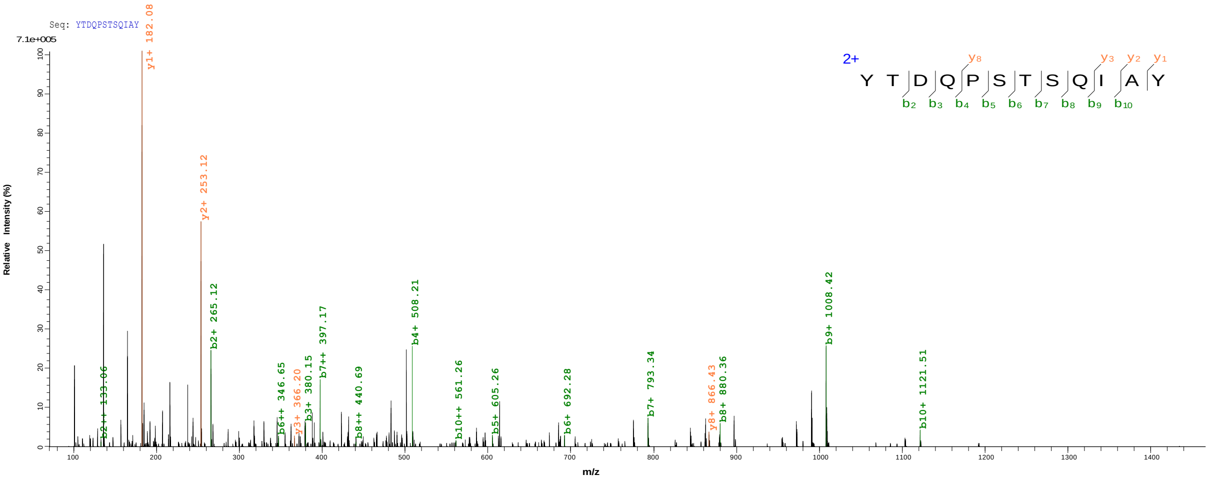

DSDPDSFQDY

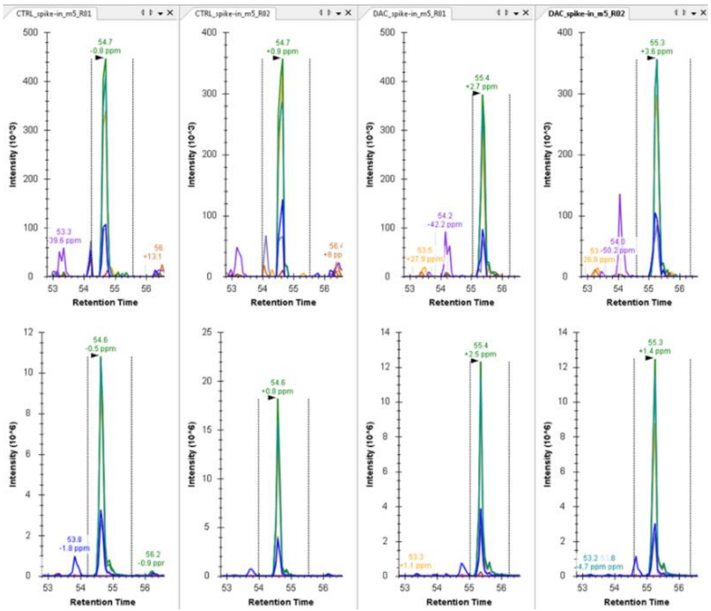

CTRL_spike-in_m5_R01
CTRL_spike-in_m5_R02
DAC_spike-in_m5_R01
DAC_spike-in_m5_R02
Intensity (10^3)
Intensity (10^6)
Retention Time

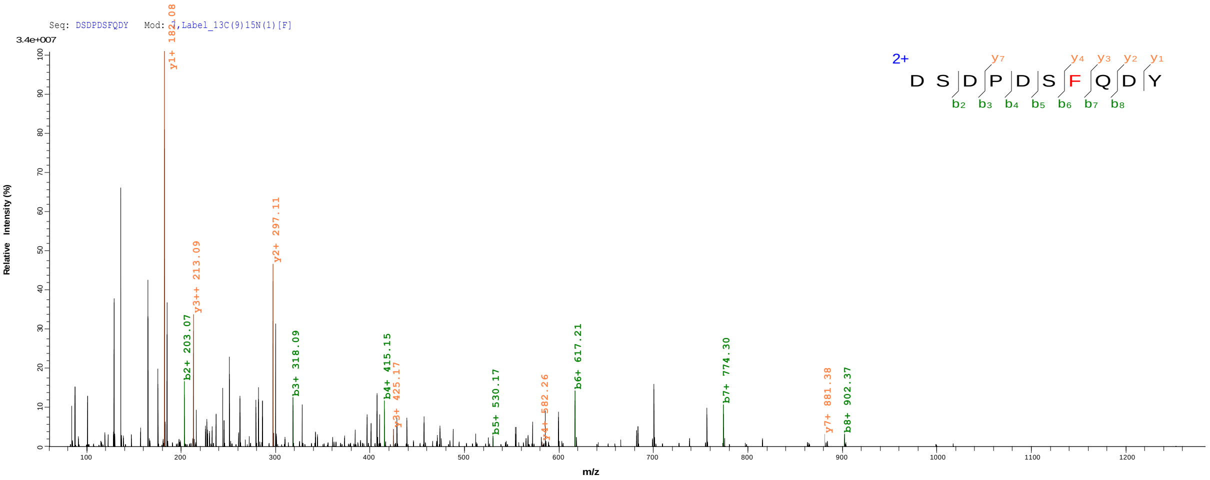

Seq: DSDPDSFQDY Mod: 7,Label_13C(9)15N(1)[F]
3.4e+007
Relative Intensity (%)
m/z
2+
D S D P D S F Q D Y
y1+ 182.08
y2+ 297.11
y3++ 213.09
b2+ 203.07
b3+ 318.09
b4+ 415.15
y3+ 425.17
b5+ 530.17
b6+ 617.21
b7+ 774.30
y7+ 881.38
b8+ 902.37

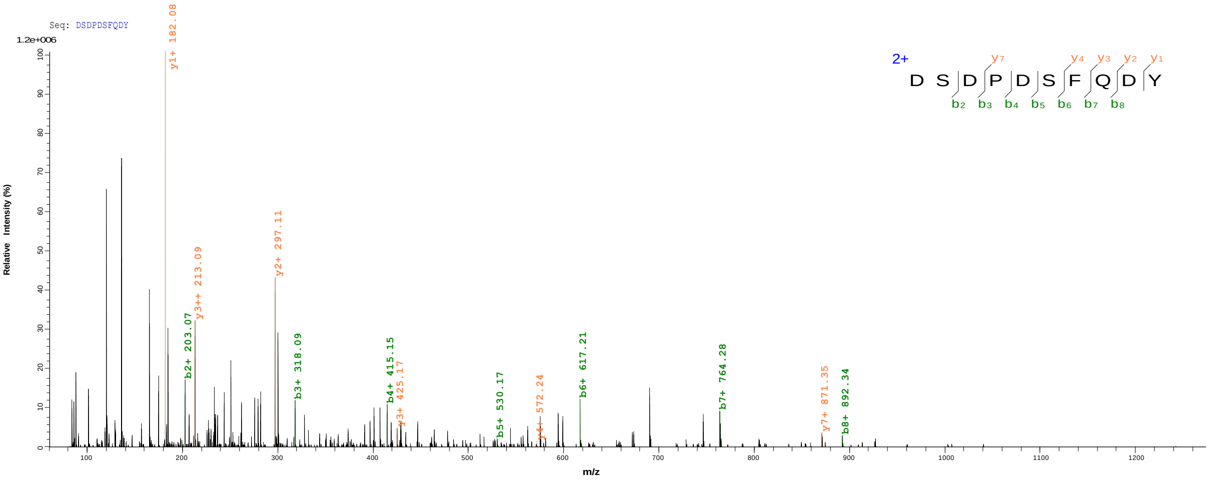

Seq: DSDPDSFQDY
1.2e+006
Relative Intensity (%)
m/z
2+
D S D P D S F Q D Y
y1+ 182.08
y2+ 297.11
y3++ 213.09
b2+ 203.07
b3+ 318.09
b4+ 415.15
y3+ 425.17
b5+ 530.17
y4+ 572.24
b6+ 617.21
b7+ 764.28
y7+ 871.35
b8+ 892.34

FASPLTGIA

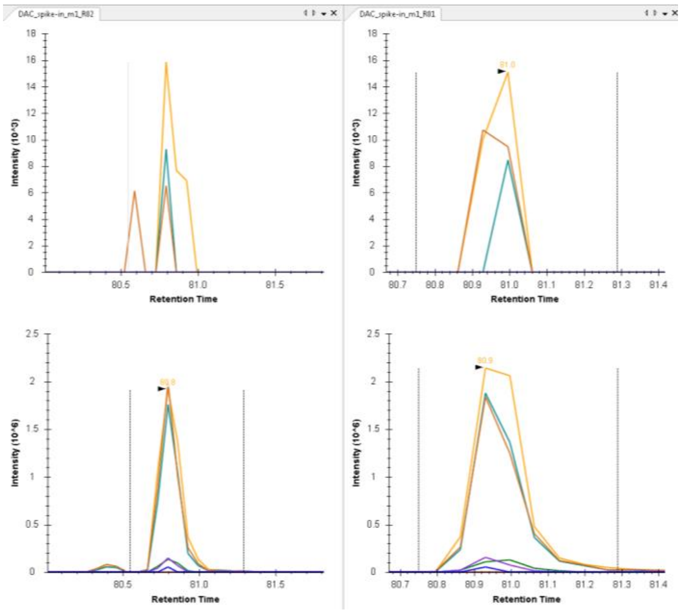

Intensity (10^3)
Intensity (10^6)
Retention Time

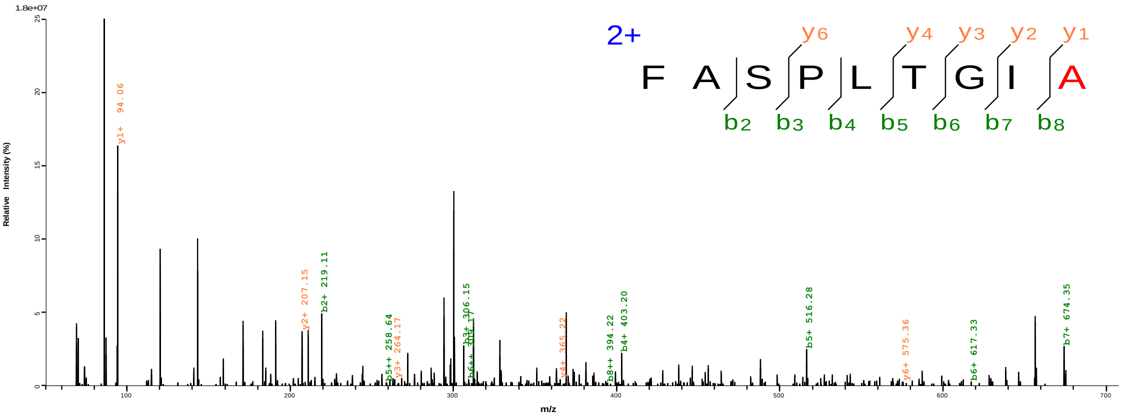

1.8e+07
2+
y6 y4 y3 y2 y1
F A S P L T G I A
b2 b3 b4 b5 b6 b7 b8
y1+ 94.06
y2+ 207.15
b2+ 219.11
b3+ 306.15
b4+ 403.20
b5+ 516.28
y6+ 575.36
b6+ 617.33
b7+ 674.35
Relative Intensity (%)
m/z

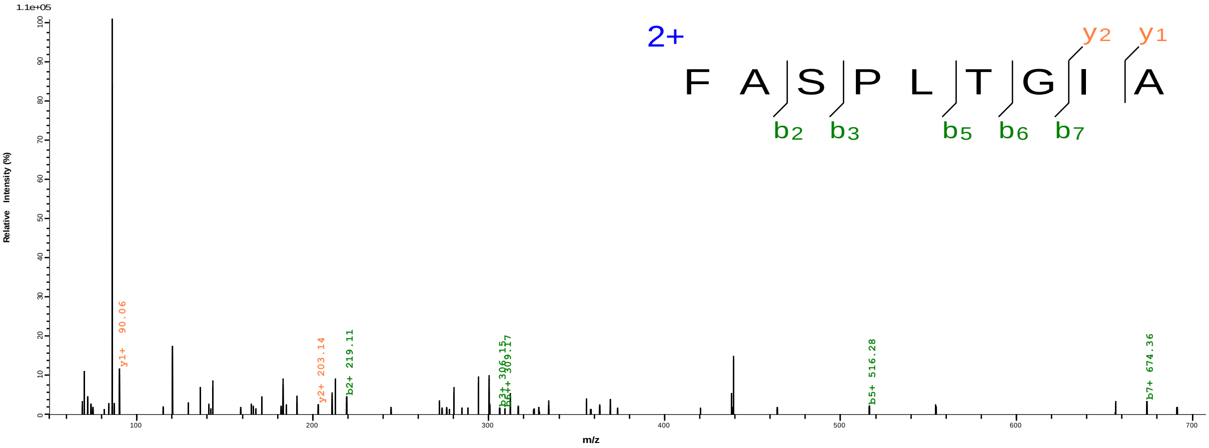

1.1e+05
2+
y2 y1
F A S P L T G I A
b2 b3 b5 b6 b7
y1+ 90.06
y2+ 203.14
b2+ 219.11
b5+ 516.28
b7+ 674.36
Relative Intensity (%)
m/z

ISSKDLGYDYSY

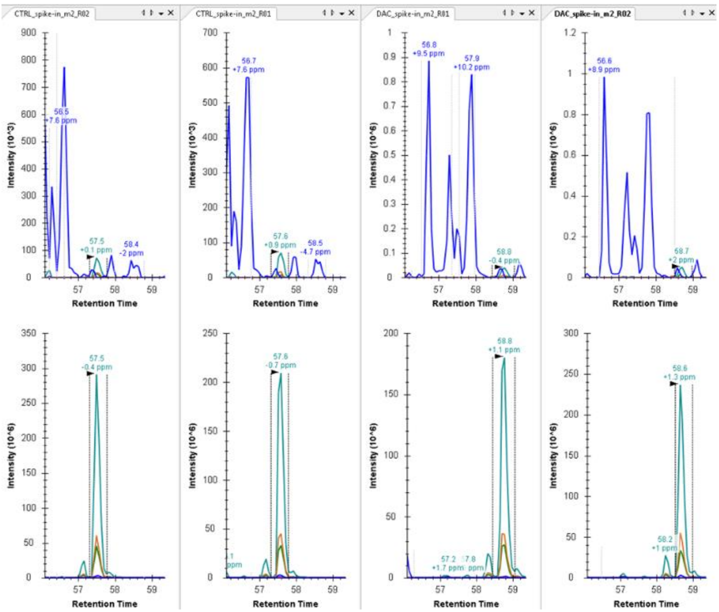

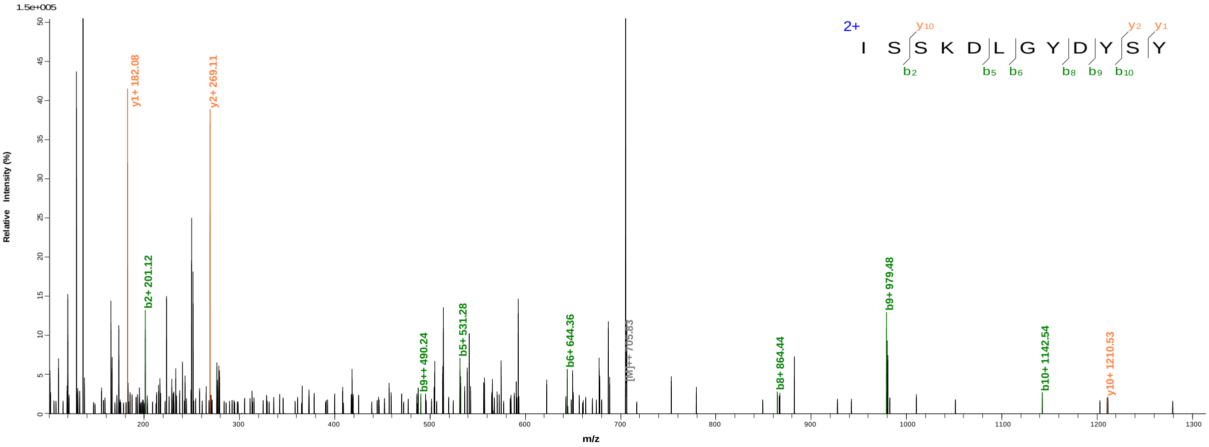

LMEKEDYHSLY

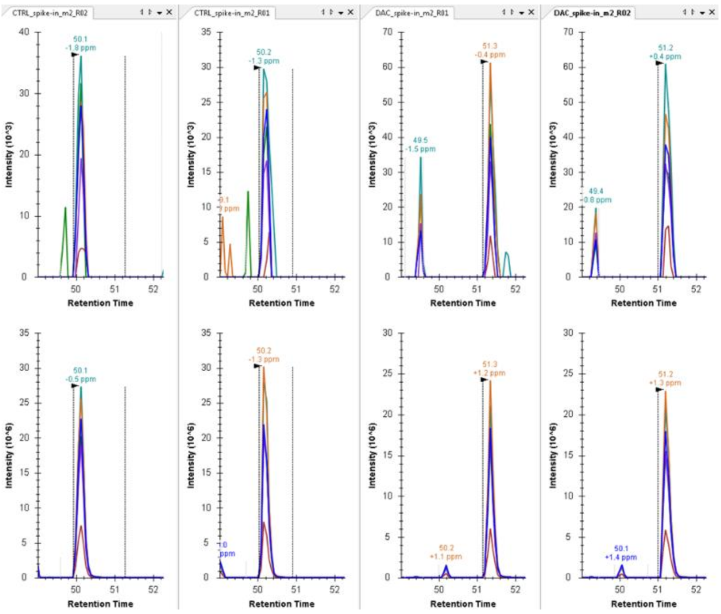

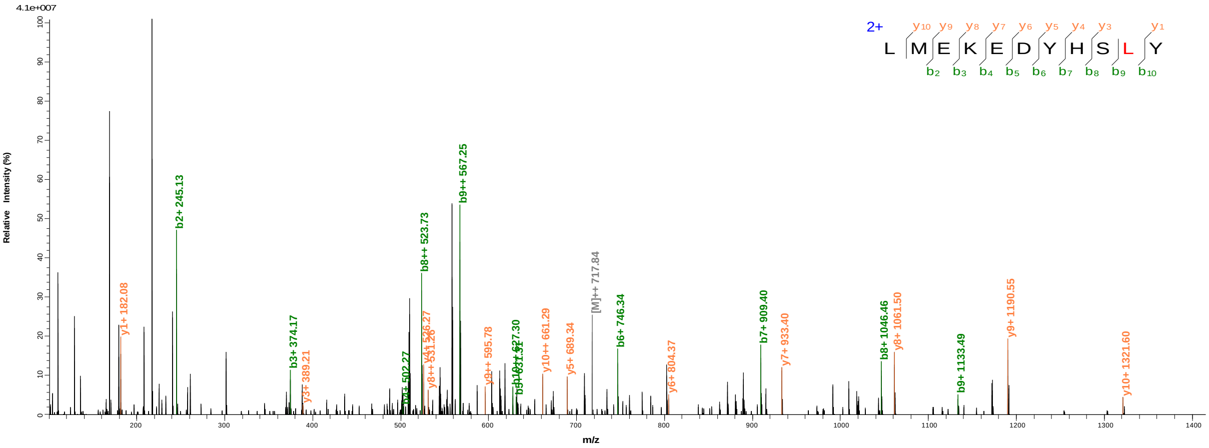

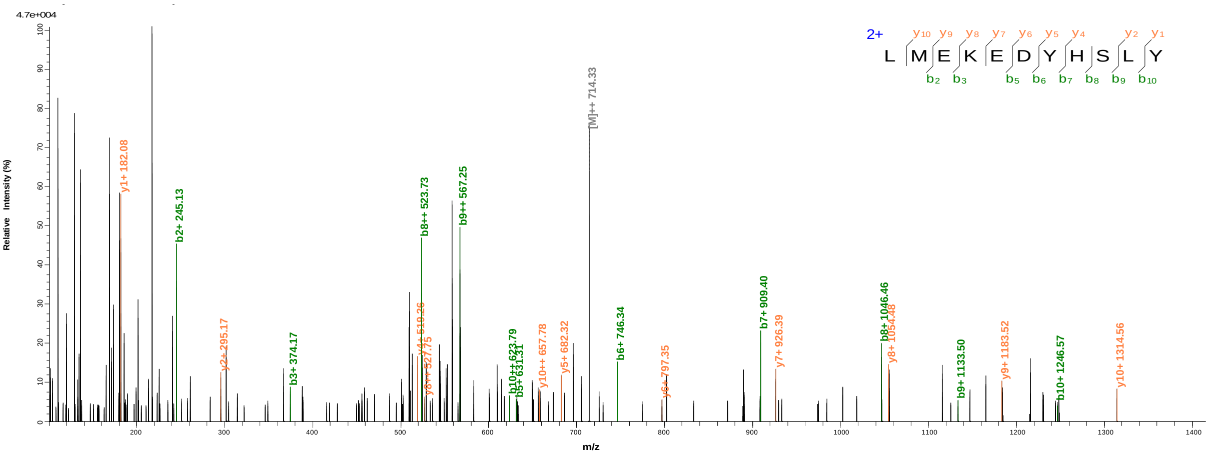

RLPSSADVEF

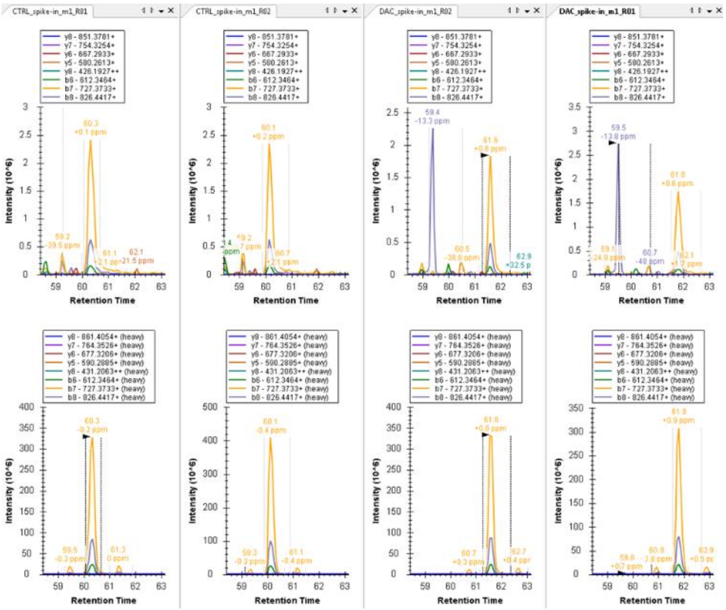

CTRL_spike-in_m1_R01
CTRL_spike-in_m1_R02
DAC_spike-in_m1_R02
DAC_spike-in_m1_R01
Intensity (10^6)
Retention Time

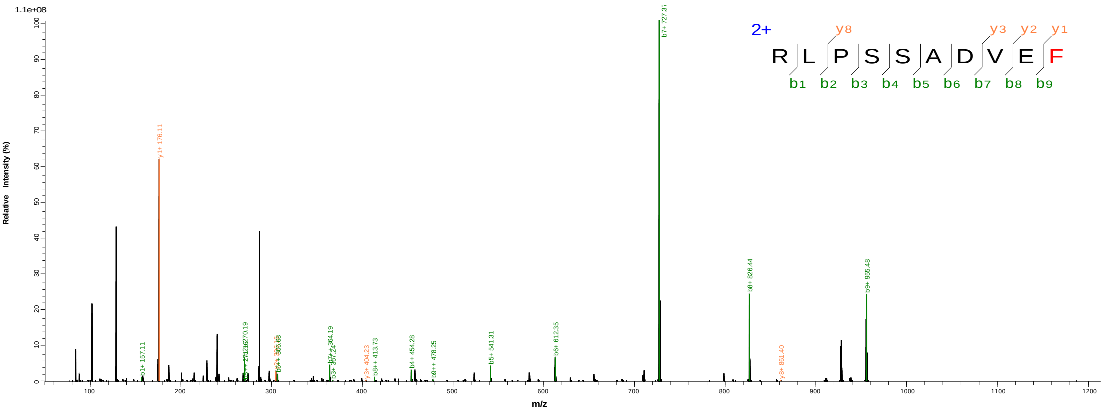

1.1e+08
2+
R L P S S A D V E F
y8 y3 y2 y1
b1 b2 b3 b4 b5 b6 b7 b8 b9
Relative Intensity (%)
m/z

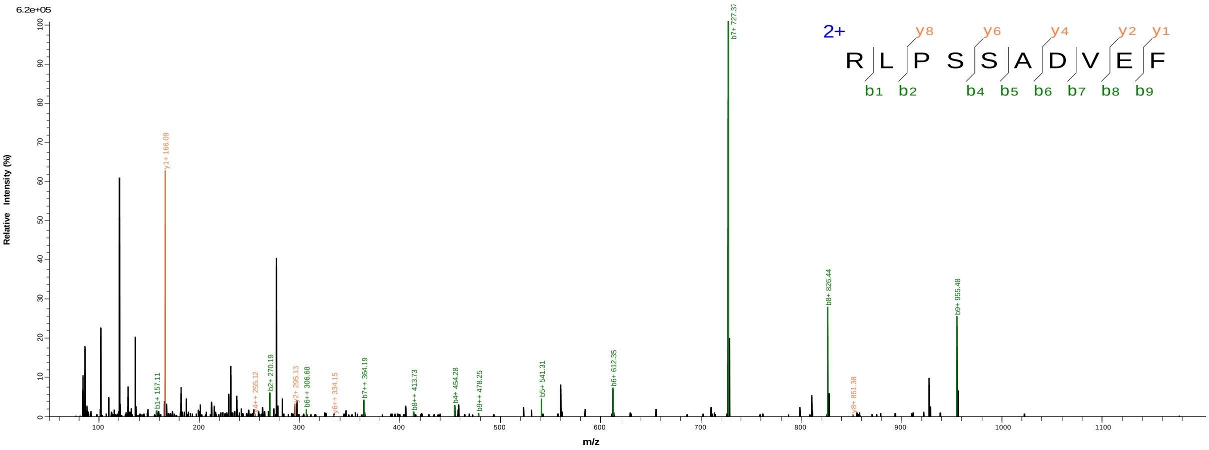

6.2e+05
2+
R L P S S A D V E F
y8 y6 y4 y2 y1
b1 b2 b4 b5 b6 b7 b8 b9
Relative Intensity (%)
m/z

ALIFGTASY

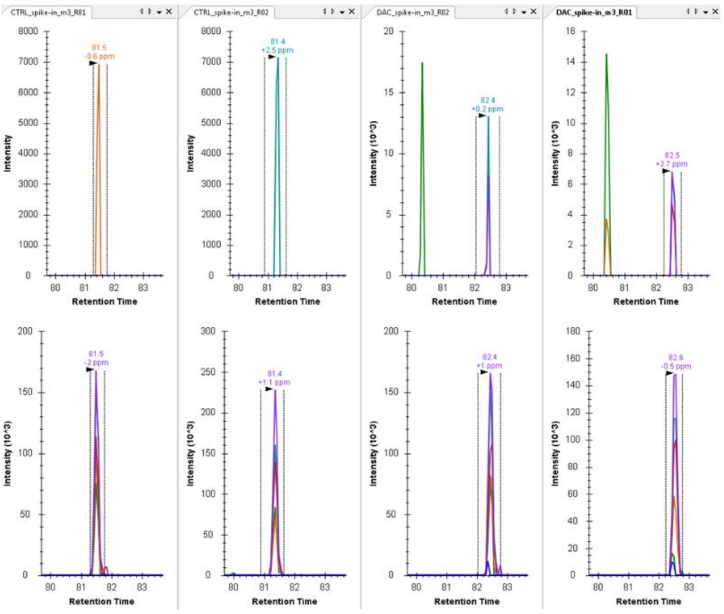

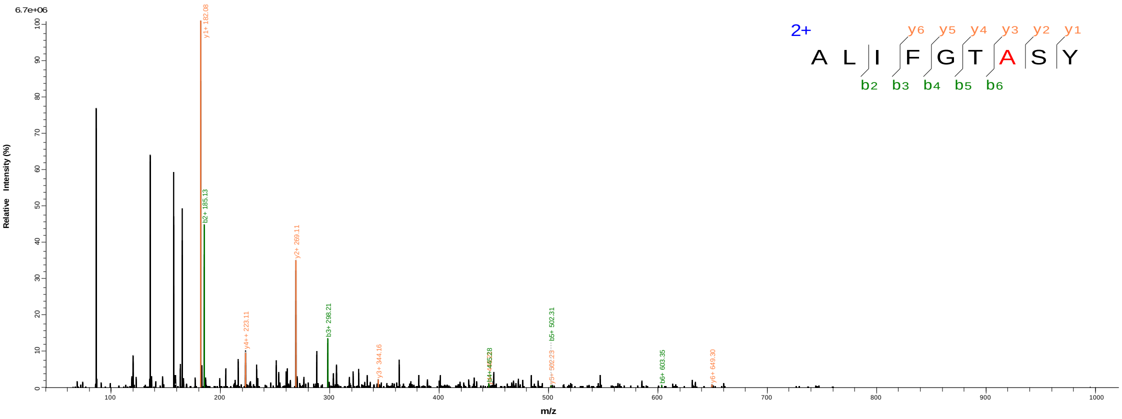

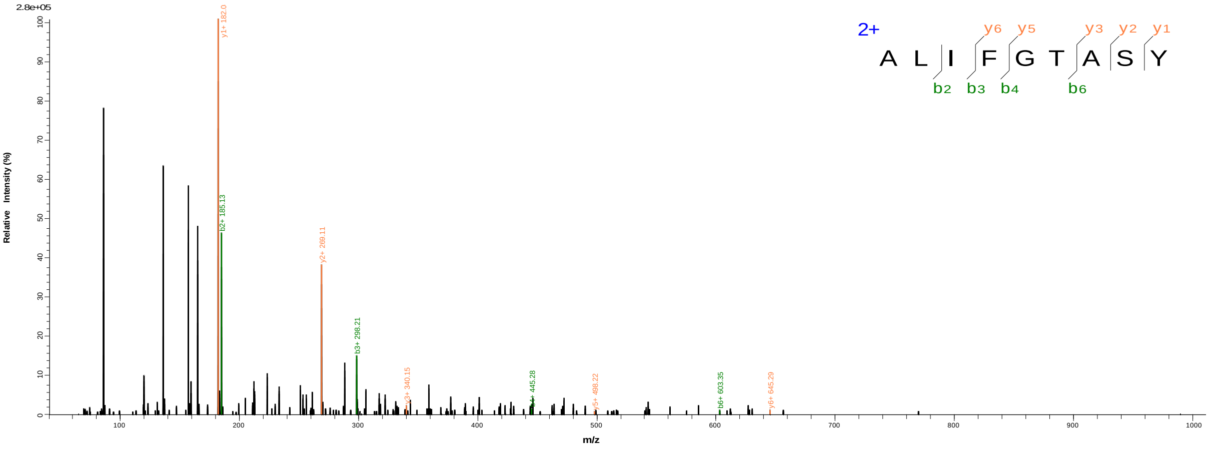

FSHEGPAFL

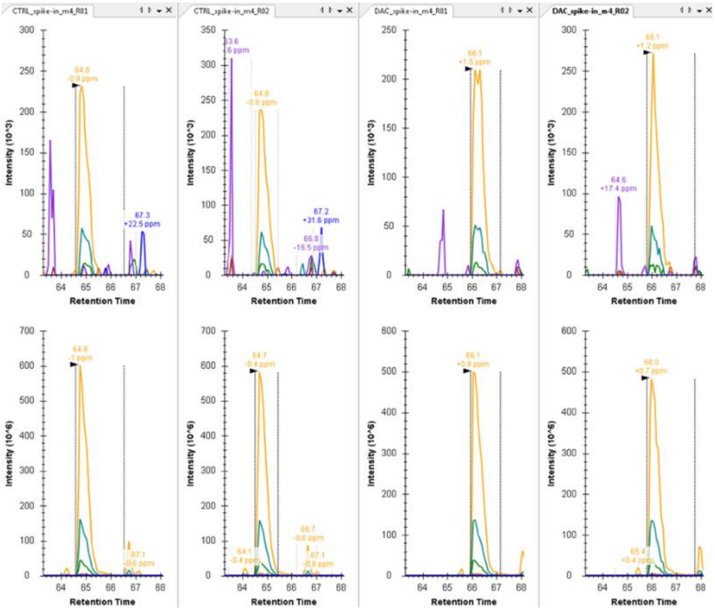

CTRL_spike-in_m4_R01
CTRL_spike-in_m4_R02
DAC_spike-in_m4_R01
DAC_spike-in_m4_R02
Intensity (10^3)
Intensity (10^6)
Retention Time

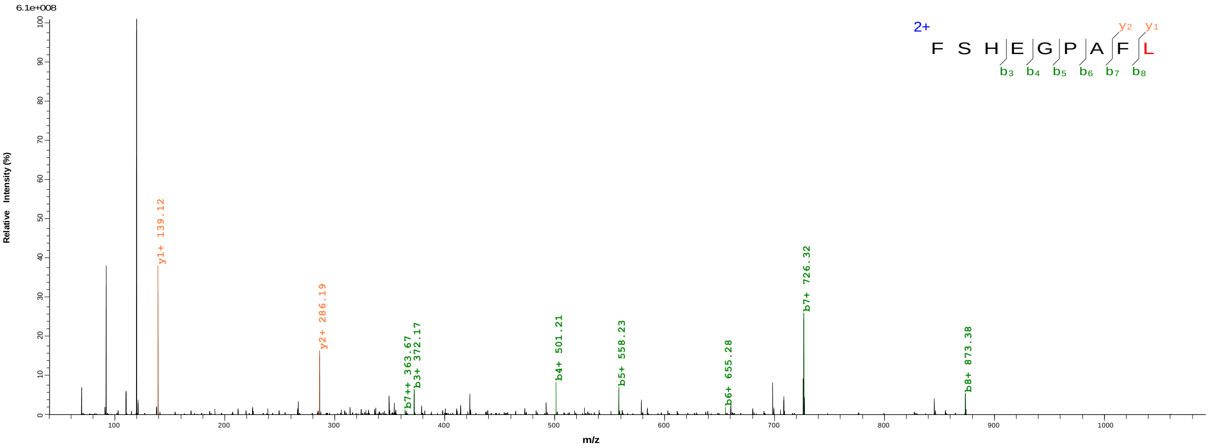

6.1e+008
Relative Intensity (%)
m/z
2+
F S H E G P A F L
y2 y1
b3 b4 b5 b6 b7 b8
y1+ 139.12
y2+ 286.19
b7++ 363.67
b3+ 372.17
b4+ 501.21
b5+ 558.23
b6+ 655.28
b7+ 726.32
b8+ 873.38

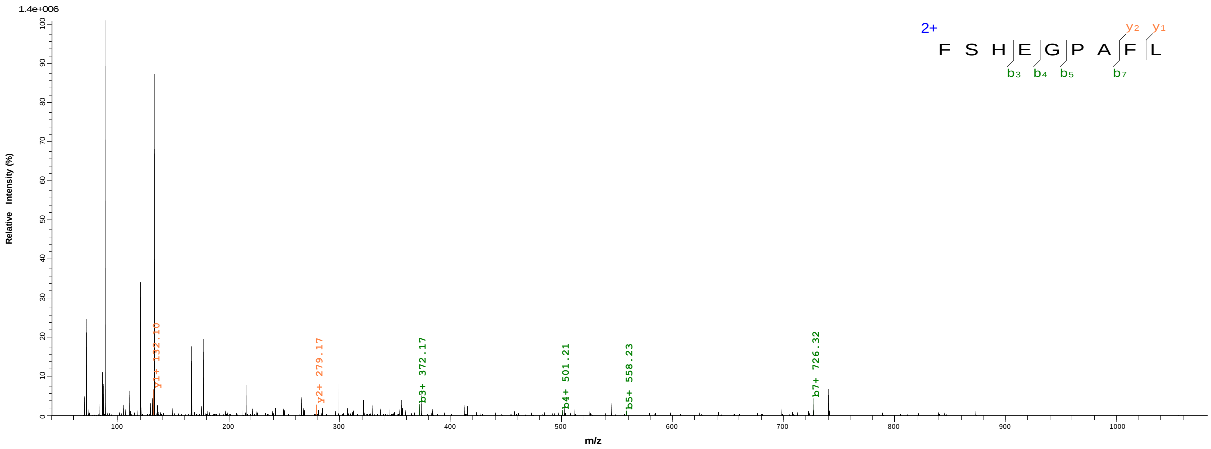

1.4e+006
Relative Intensity (%)
m/z
2+
F S H E G P A F L
y2 y1
b3 b4 b5 b7
y2+ 279.17
b3+ 372.17
b4+ 501.21
b5+ 558.23
b7+ 726.32

IQWPSREF

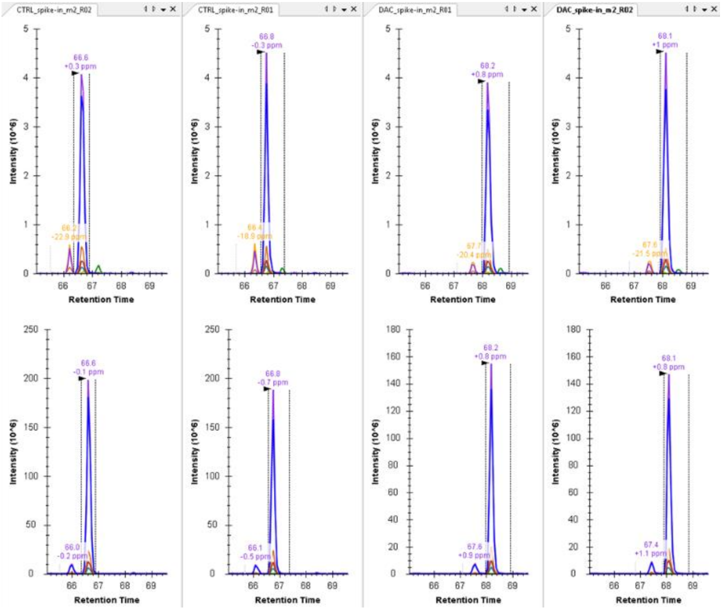

CTRL_spike-in_m2_R02
CTRL_spike-in_m2_R01
DAC_spike-in_m2_R01
DAC_spike-in_m2_R02
Intensity (10^6)
Retention Time

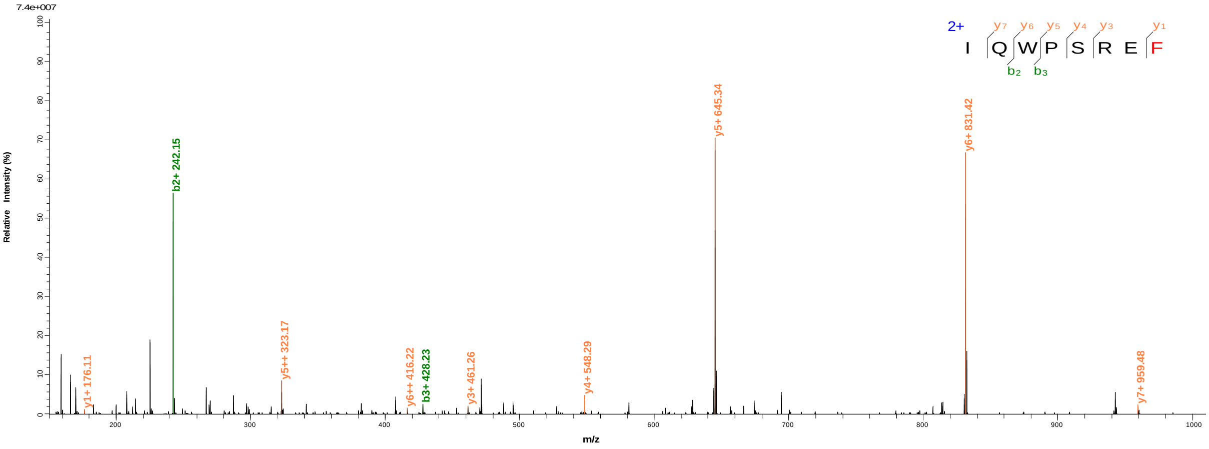

7.4e+007
Relative Intensity (%)
m/z
2+
I Q W P S R E F
y7 y6 y5 y4 y3 y1
b2 b3
y1+ 176.11
b2+ 242.15
y5++ 323.17
y6++ 416.22
b3+ 428.23
y3+ 461.26
y4+ 548.29
y5+ 645.34
y6+ 831.42
y7+ 959.48

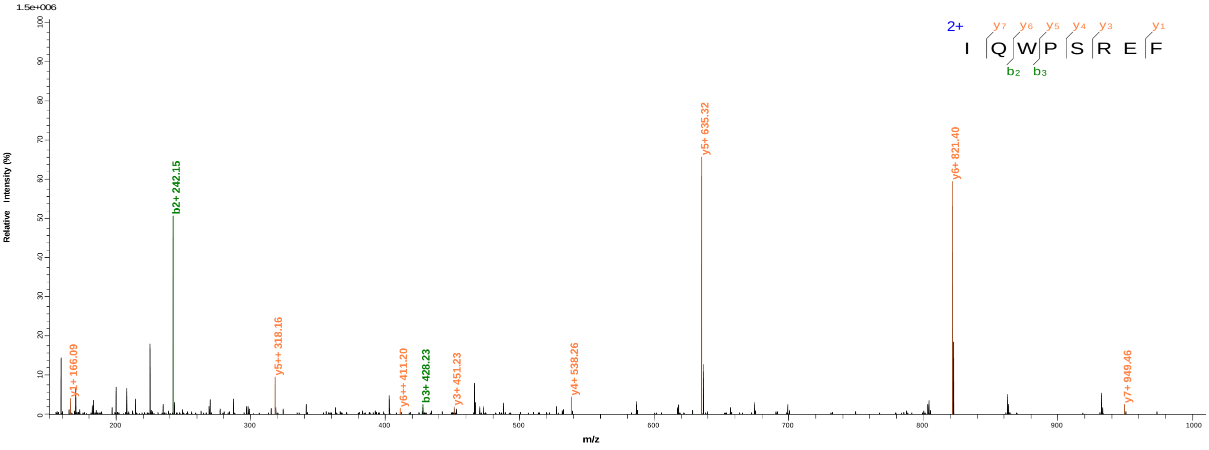

1.5e+006
Relative Intensity (%)
m/z
2+
I Q W P S R E F
y7 y6 y5 y4 y3 y1
b2 b3
y1+ 166.09
b2+ 242.15
y5++ 318.16
y6++ 411.20
b3+ 428.23
y3+ 451.23
y4+ 538.26
y5+ 635.32
y6+ 821.40
y7+ 949.46

LQNPNQSVV

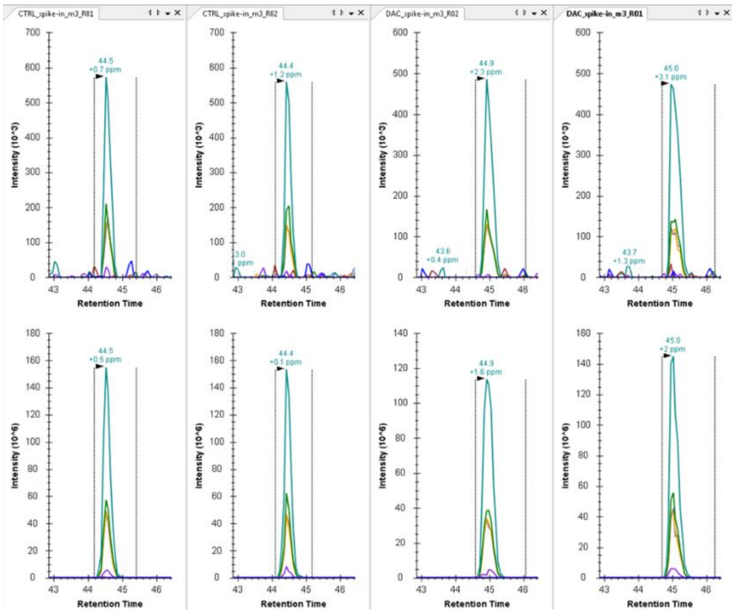

CTRL_spike-in_m3_R01
CTRL_spike-in_m3_R02
DAC_spike-in_m3_R02
DAC_spike-in_m3_R01
Intensity (10^3)
Intensity (10^6)
Retention Time
44.5 +0.7 ppm
44.4 +1.2 ppm
44.9 +2.3 ppm
45.0 +3.1 ppm
43.6 +0.4 ppm
43.7 +1.3 ppm
44.5 +0.5 ppm
44.4 +0.1 ppm
44.9 +1.6 ppm
45.0 +2 ppm

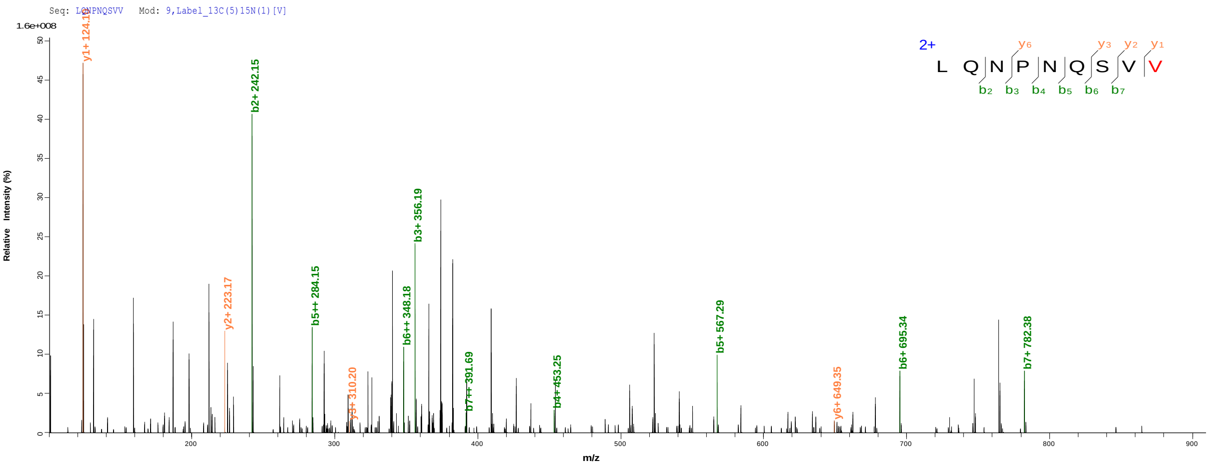

Seq: LQNPNQSVV Mod: 9,Label_13C(5)15N(1)[V]
1.6e+008
Relative Intensity (%)
m/z
2+
L Q N P N Q S V V
y6 y3 y2 y1
b2 b3 b4 b5 b6 b7
y1+ 124.10
b2+ 242.15
y2+ 223.17
b5++ 284.15
y3+ 310.20
b6++ 348.18
b3+ 356.19
b7++ 391.69
b4+ 453.25
b5+ 567.29
y6+ 649.35
b6+ 695.34
b7+ 782.38

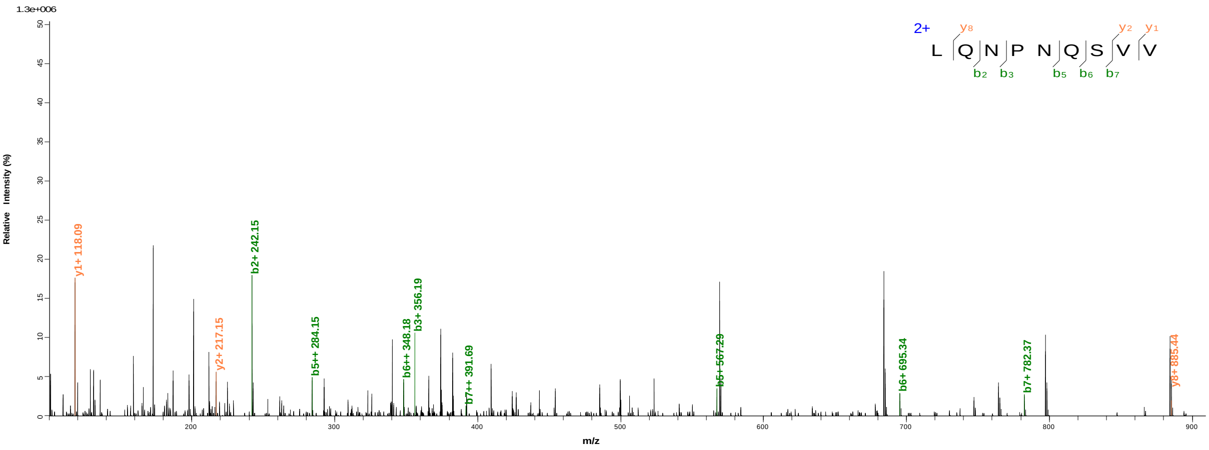

1.3e+006
Relative Intensity (%)
m/z
2+
L Q N P N Q S V V
y8 y2 y1
b2 b3 b5 b6 b7
y1+ 118.09
b2+ 242.15
y2+ 217.15
b5++ 284.15
b6++ 348.18
b3+ 356.19
b7++ 391.69
b5+ 567.29
b6+ 695.34
b7+ 782.37
y8+ 885.44

TLISPNSVF

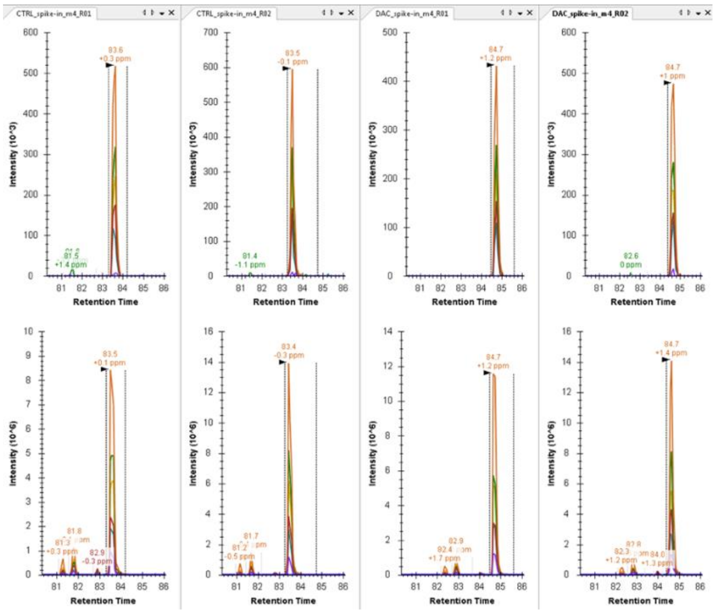

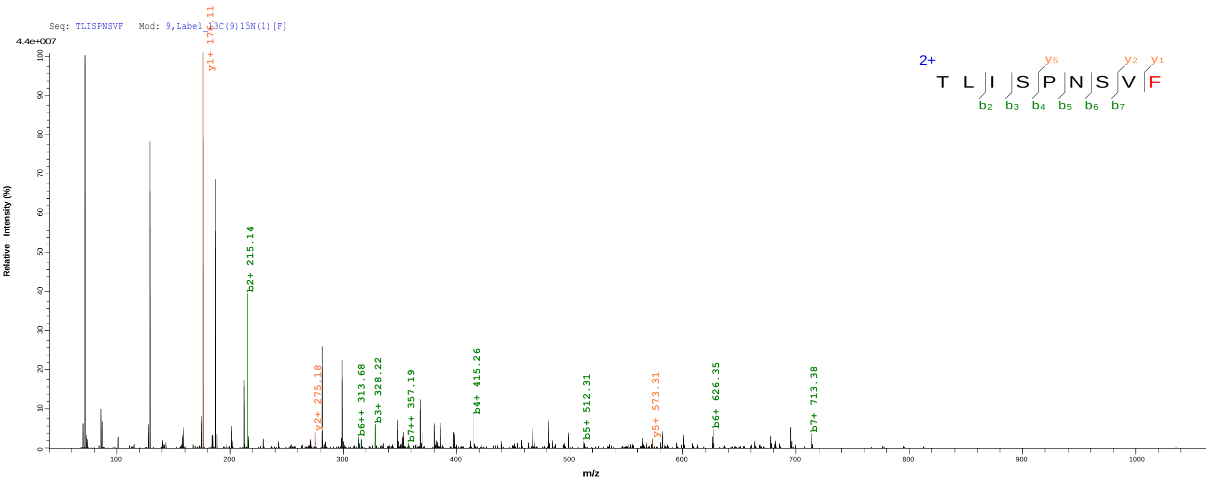
Seq: TLISPNSVF Mod: 9,Label 13C(9)15N(1)[F]
4.4e+007
Relative Intensity (%)
m/z
2+
T L I S P N S V F

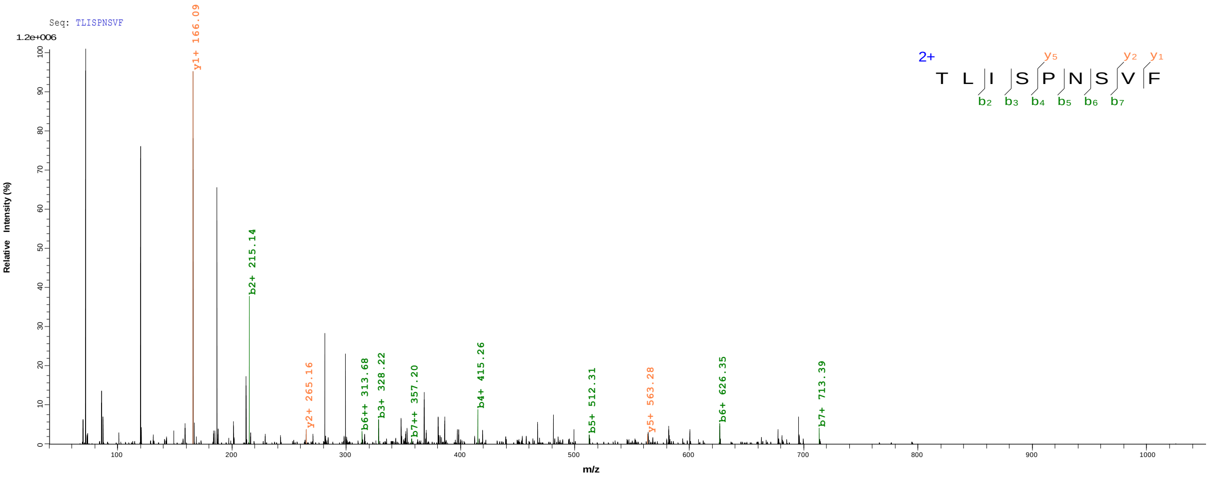
Seq: TLISPNSVF
1.2e+006
Relative Intensity (%)
m/z
2+
T L I S P N S V F

VTAPDNLGY

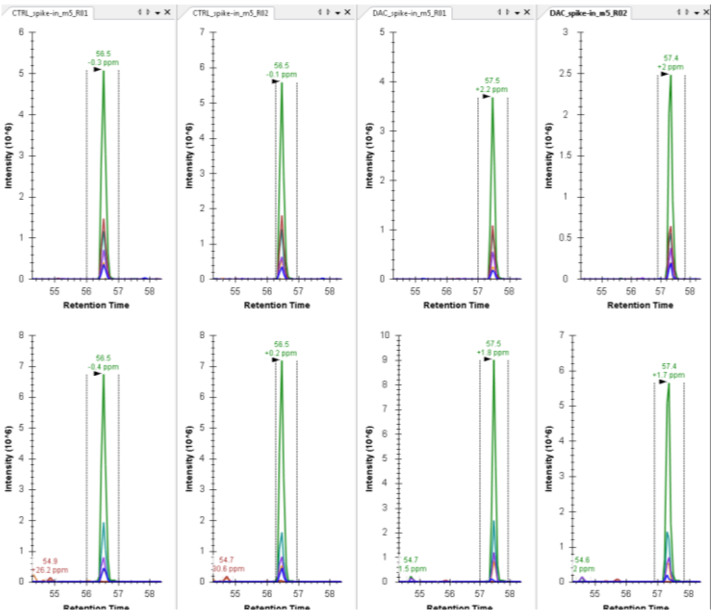
CTRL_spike-in_m5_R01
CTRL_spike-in_m5_R02
DAC_spike-in_m5_R01
DAC_spike-in_m5_R02
Intensity (10^6)
Retention Time

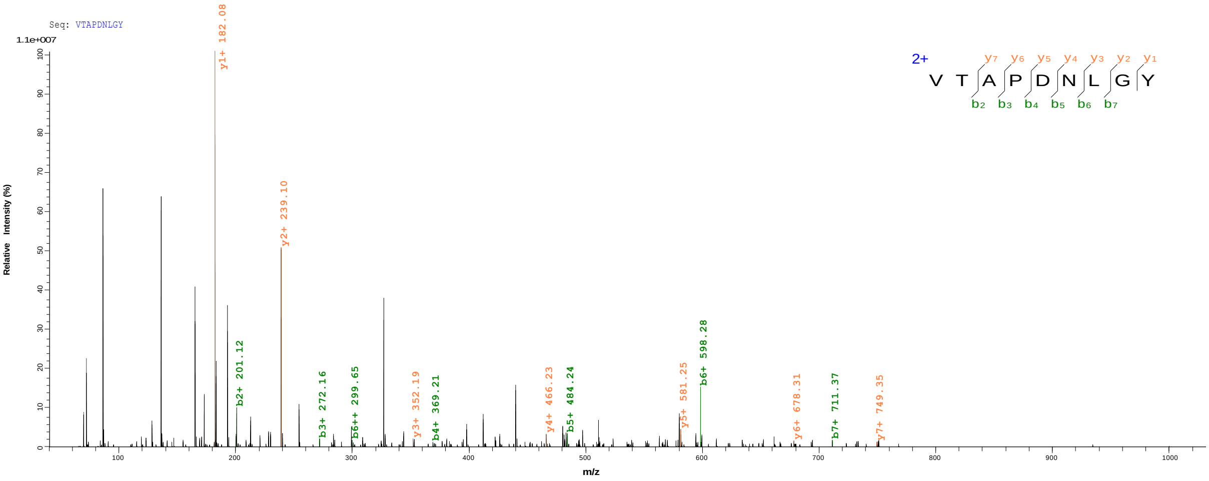
Seq: VTAPDNLGY
1.1e+007
Relative Intensity (%)
m/z
y1+ 182.08
y2+ 239.10
b2+ 201.12
b3+ 272.16
b6++ 299.65
y3+ 352.19
b4+ 369.21
y4+ 466.23
b5+ 484.24
y5+ 581.25
b6+ 598.28
y6+ 678.31
b7+ 711.37
y7+ 749.35
2+
V T A P D N L G Y

YSDPTGKYDPAVRSL

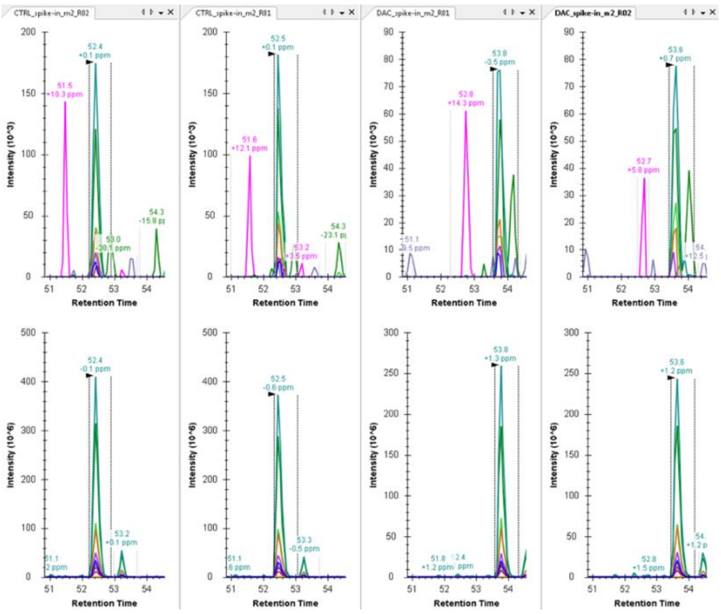

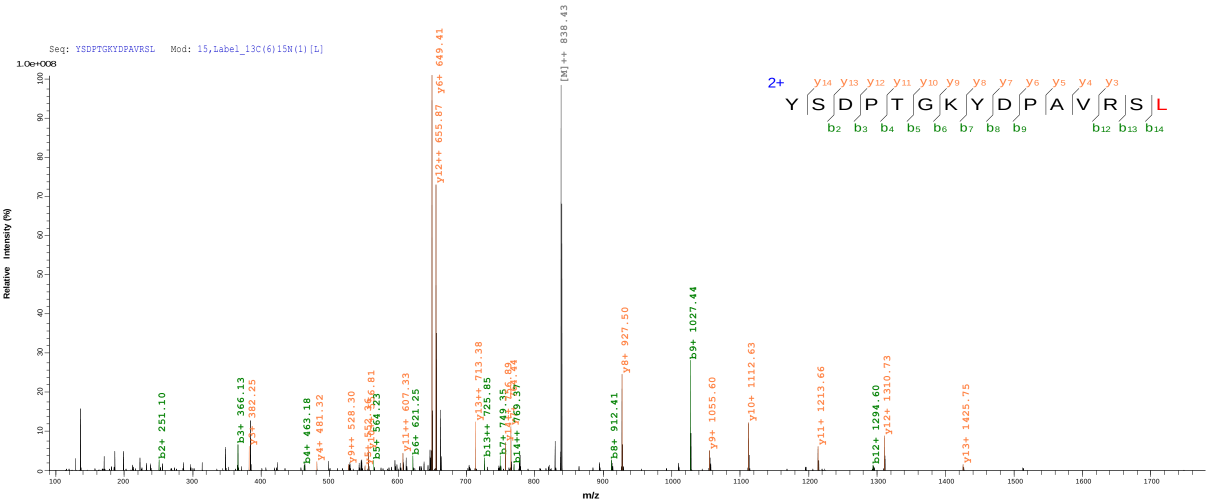

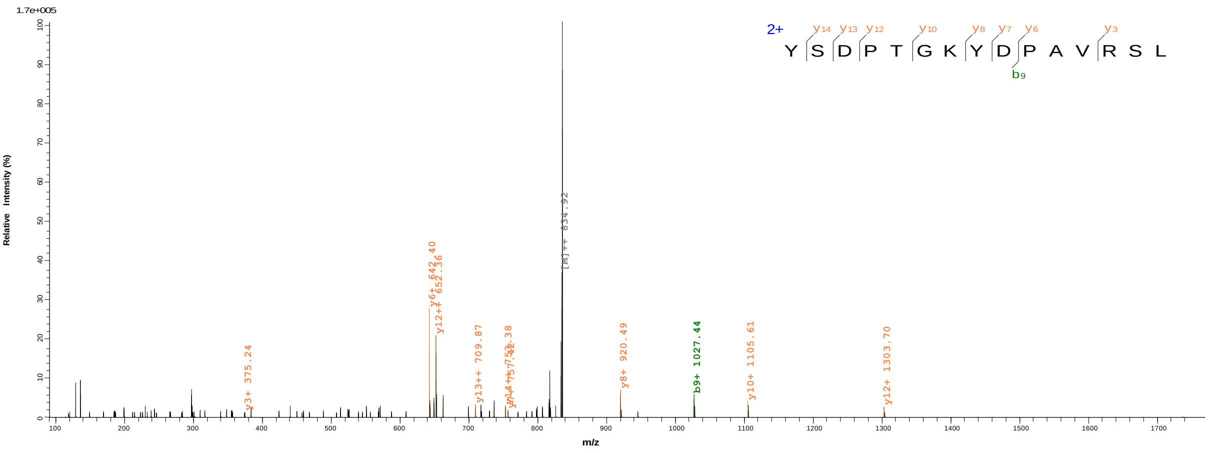

LYPEVFEKF

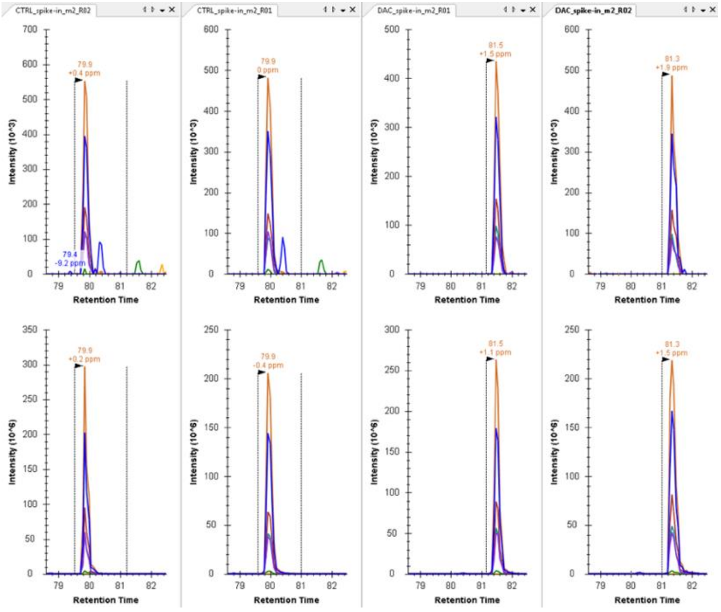
CTRL_spike-in_m2_R02
CTRL_spike-in_m2_R01
DAC_spike-in_m2_R01
DAC_spike-in_m2_R02
Intensity (10^3)
Intensity (10^6)
Retention Time

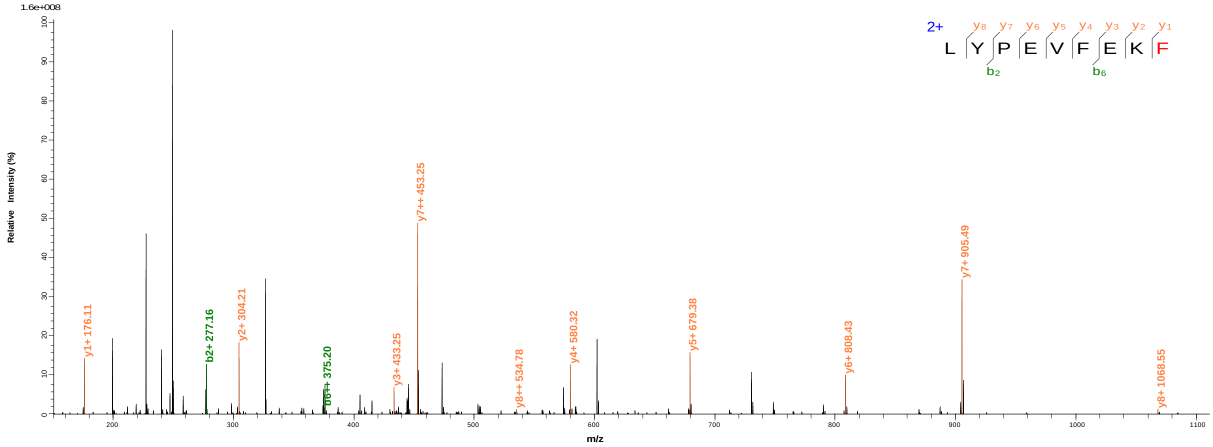
1.6e+008
Relative Intensity (%)
m/z
2+ L Y P E V F E K F
y1+ 176.11
b2+ 277.16
y2+ 304.21
b6++ 375.20
y3+ 433.25
y7++ 453.25
y8++ 534.78
y4+ 580.32
y5+ 679.38
y6+ 808.43
y7+ 905.49
y8+ 1068.55

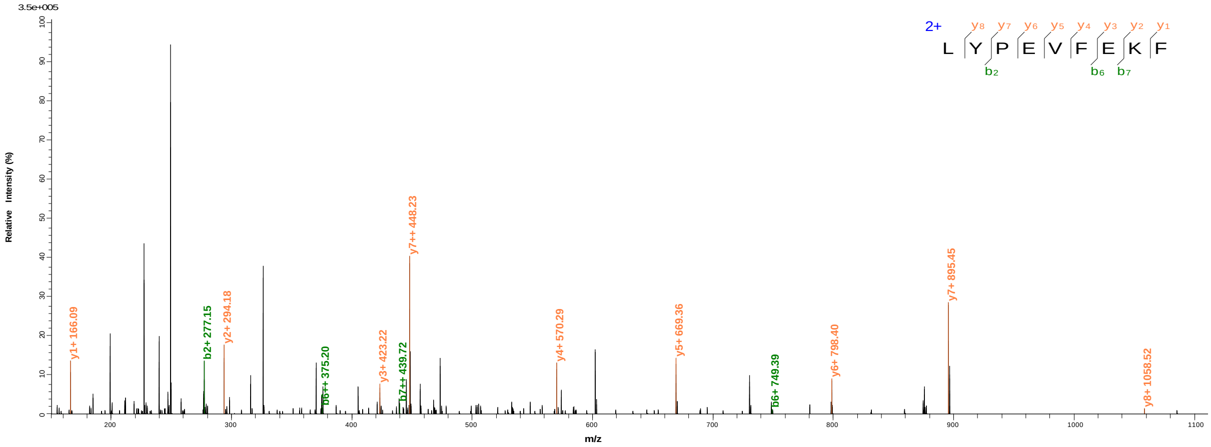
3.5e+005
Relative Intensity (%)
m/z
2+ L Y P E V F E K F
y1+ 166.09
b2+ 277.15
y2+ 294.18
b6++ 375.20
y3+ 423.22
b7++ 439.72
y7++ 448.23
y4+ 570.29
y5+ 669.36
b6+ 749.39
y6+ 798.40
y7+ 895.45
y8+ 1058.52

SPQEREQFL

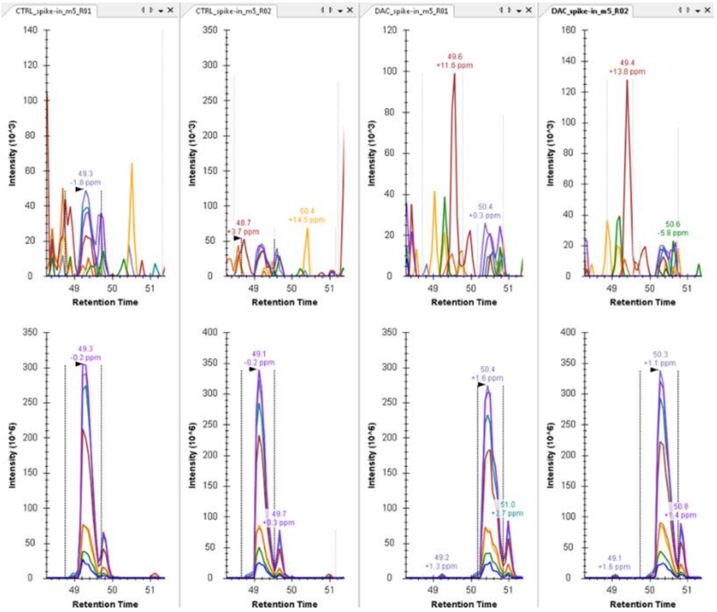
CTRL_spike-in_m5_R01
CTRL_spike-in_m5_R02
DAC_spike-in_m5_R01
DAC_spike-in_m5_R02
Intensity (10^3)
Intensity (10^6)
Retention Time
49.3
-1.8 ppm
48.7
+3.7 ppm
50.4
+14.5 ppm
49.6
+11.6 ppm
50.4
+0.3 ppm
49.4
+13.8 ppm
50.6
-5.8 ppm
49.3
-0.2 ppm
49.1
-0.2 ppm
49.7
+0.3 ppm
50.4
+1.6 ppm
51.0
+2.7 ppm
49.2
+1.3 ppm
50.3
+1.1 ppm
50.8
+1.4 ppm
49.1
+1.6 ppm

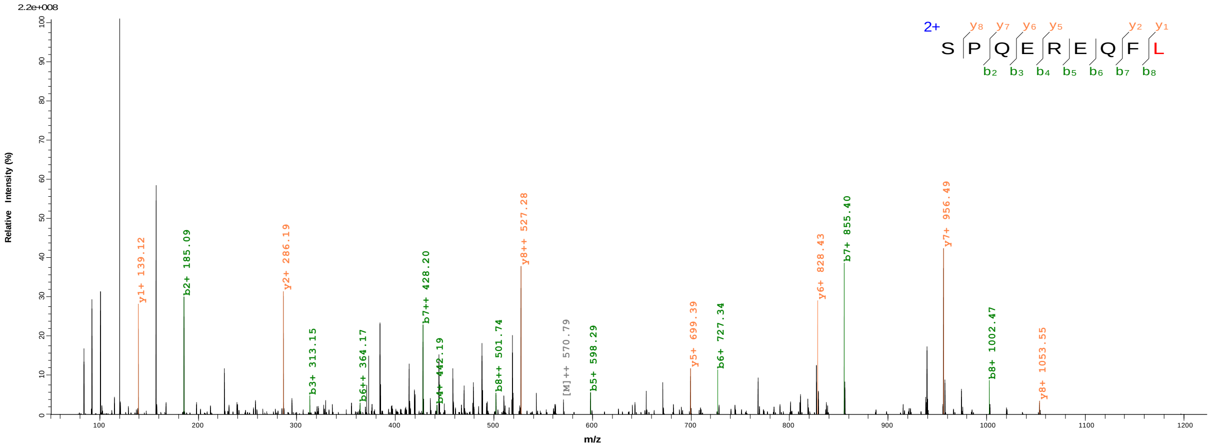
2.2e+008
Relative Intensity (%)
m/z
2+
S P Q E R E Q F L
y8 y7 y6 y5 y2 y1
b2 b3 b4 b5 b6 b7 b8
y1+ 139.12
b2+ 185.09
y2+ 286.19
b3+ 313.15
b6++ 364.17
b7++ 428.20
b4+ 442.19
b8++ 501.74
y8++ 527.28
[M]++ 570.79
b5+ 598.29
y5+ 699.39
b6+ 727.34
y6+ 828.43
b7+ 855.40
y7+ 956.49
b8+ 1002.47
y8+ 1053.55

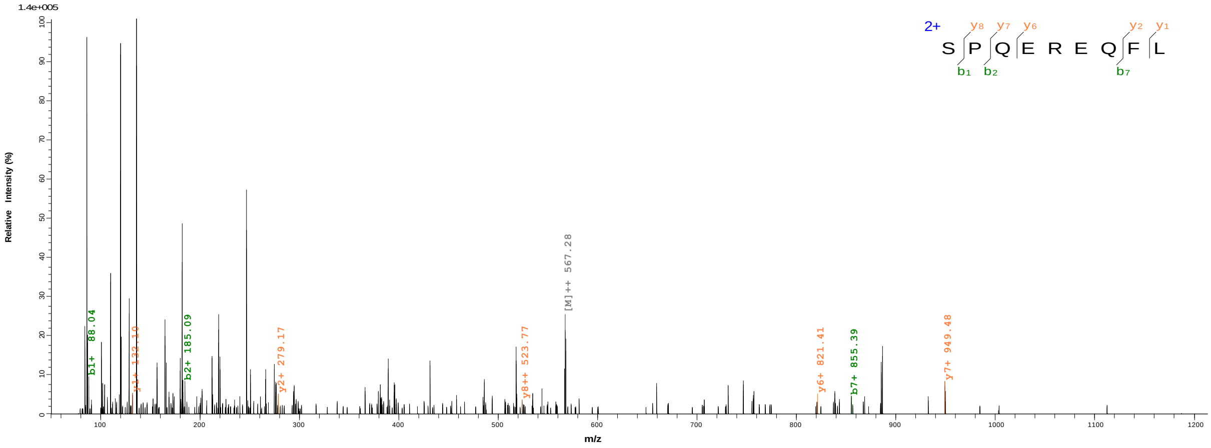
1.4e+005
Relative Intensity (%)
m/z
2+
S P Q E R E Q F L
y8 y7 y6 y2 y1
b1 b2 b7
b1+ 88.04
y1+ 132.10
b2+ 185.09
y2+ 279.17
y8++ 523.77
[M]++ 567.28
y6+ 821.41
b7+ 855.39
y7+ 949.48

TFSFRNAL

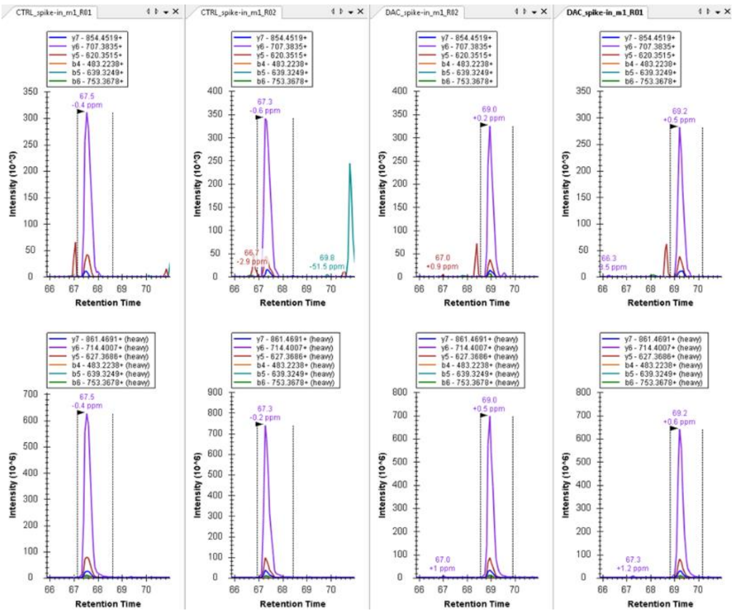

CTRL_spike-in_m1_R01
CTRL_spike-in_m1_R02
DAC_spike-in_m1_R02
DAC_spike-in_m1_R01
y7 - 854.4519+
y6 - 707.3835+
y5 - 620.3515+
b4 - 483.2238+
b5 - 639.3249+
b6 - 753.3678+
y7 - 861.4691+ (heavy)
y6 - 714.4007+ (heavy)
y5 - 627.3686+ (heavy)
b4 - 483.2238+ (heavy)
b5 - 639.3249+ (heavy)
b6 - 753.3678+ (heavy)
Intensity (10^3)
Intensity (10^6)
Retention Time

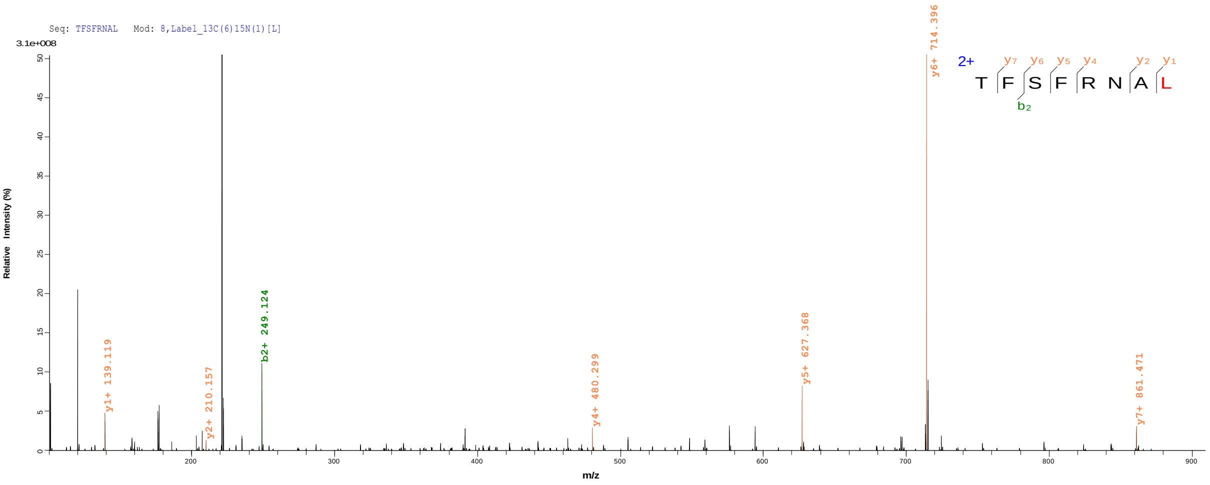

Seq: TFSFRNAL Mod: 8,Label_13C(6)15N(1)[L]
3.1e+008
Relative Intensity (%)
m/z
y6+ 714.396
y1+ 139.119
y2+ 210.157
b2+ 249.124
y4+ 480.299
y5+ 627.368
y7+ 861.471
2+
T F S F R N A L

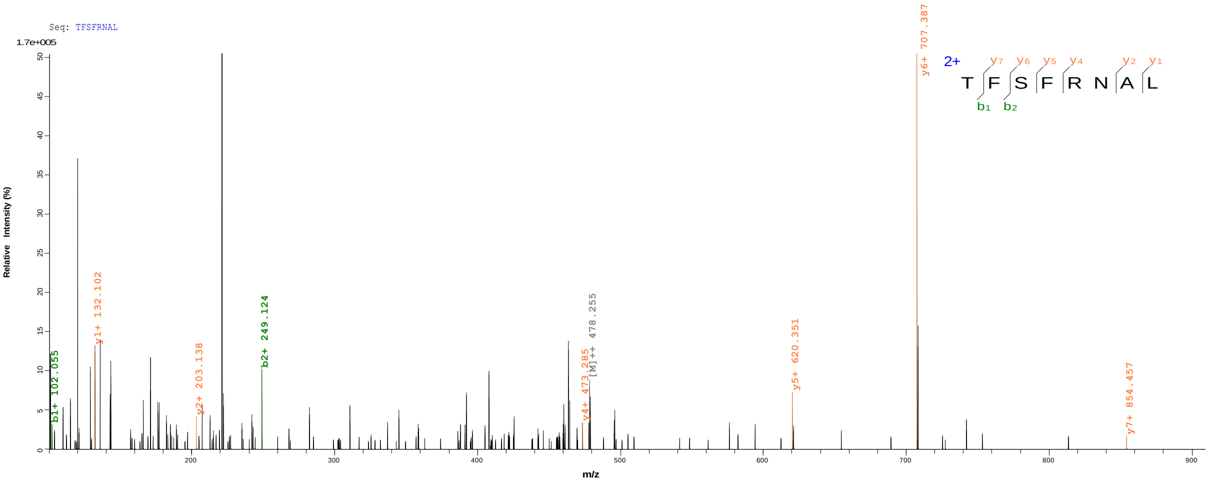

Seq: TFSFRNAL
1.7e+005
Relative Intensity (%)
m/z
y6+ 707.387
b1+ 102.055
y1+ 132.102
y2+ 203.138
b2+ 249.124
y4+ 473.285
[M]++ 478.255
y5+ 620.351
y7+ 854.457
2+
T F S F R N A L

EVDPIGHVY

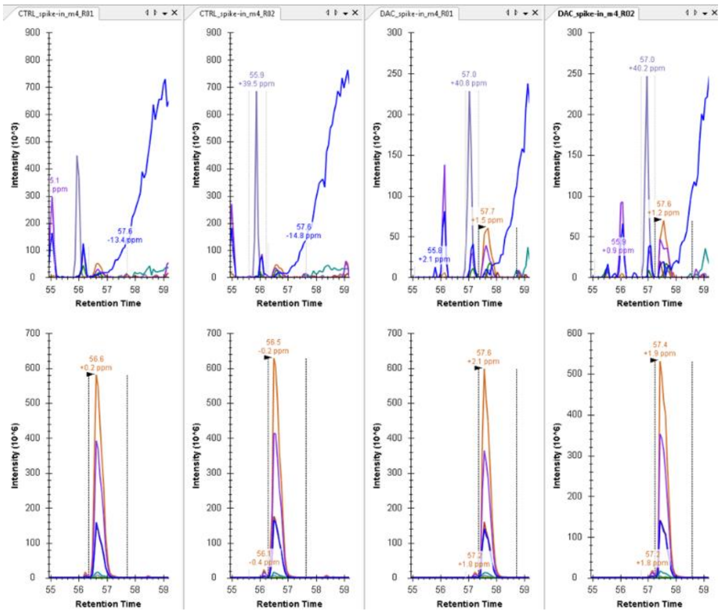

CTRL_spike-in_m4_R01
CTRL_spike-in_m4_R02
DAC_spike-in_m4_R01
DAC_spike-in_m4_R02
Intensity (10^3)
Intensity (10^6)
Retention Time

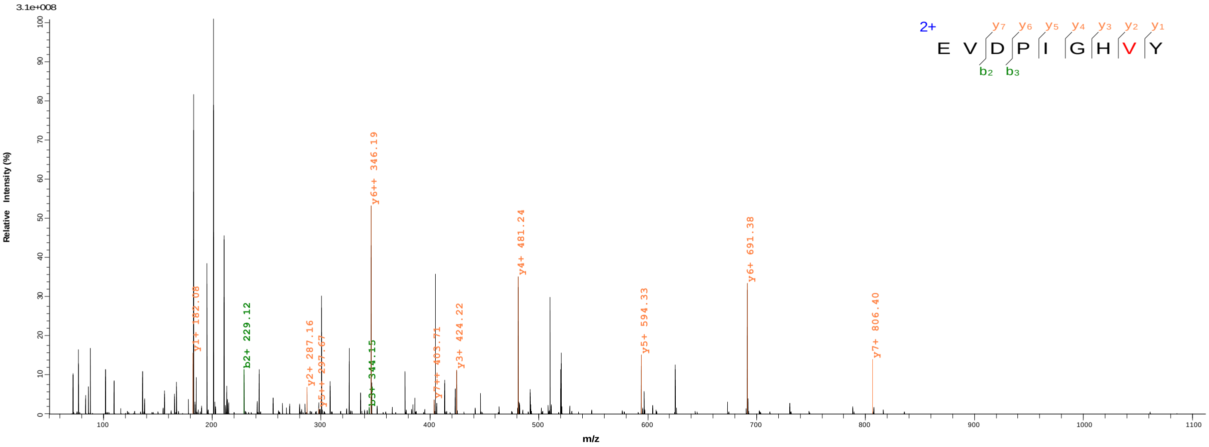

3.1e+008
Relative Intensity (%)
m/z
2+
E V D P I G H V Y
y1+ 182.08
b2+ 229.12
y2+ 287.16
b3+ 344.15
y6++ 346.19
y3+ 424.22
y4+ 481.24
y5+ 594.33
y6+ 691.38
y7+ 806.40

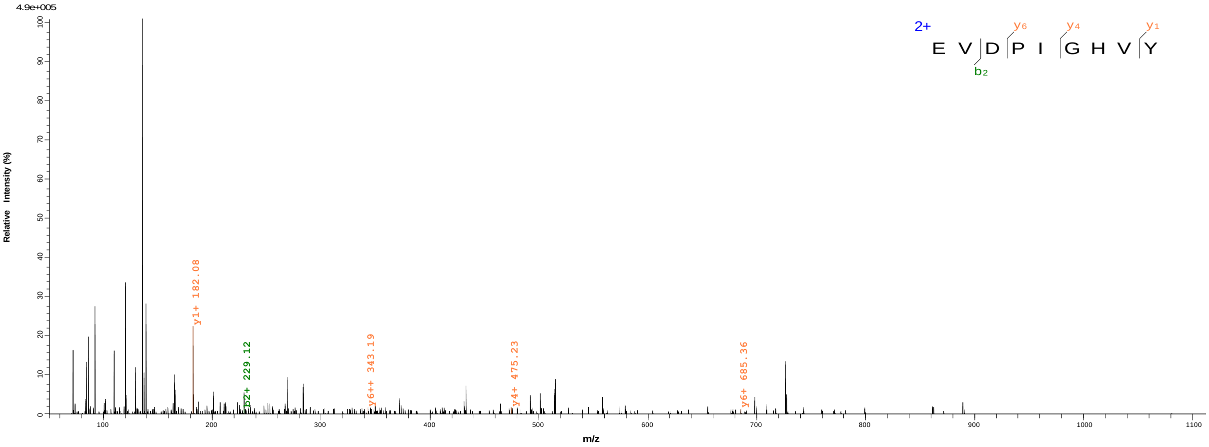

4.9e+005
Relative Intensity (%)
m/z
2+
E V D P I G H V Y
y1+ 182.08
b2+ 229.12
y6++ 343.19
y4+ 475.23
y6+ 685.36

HTMEVTVY

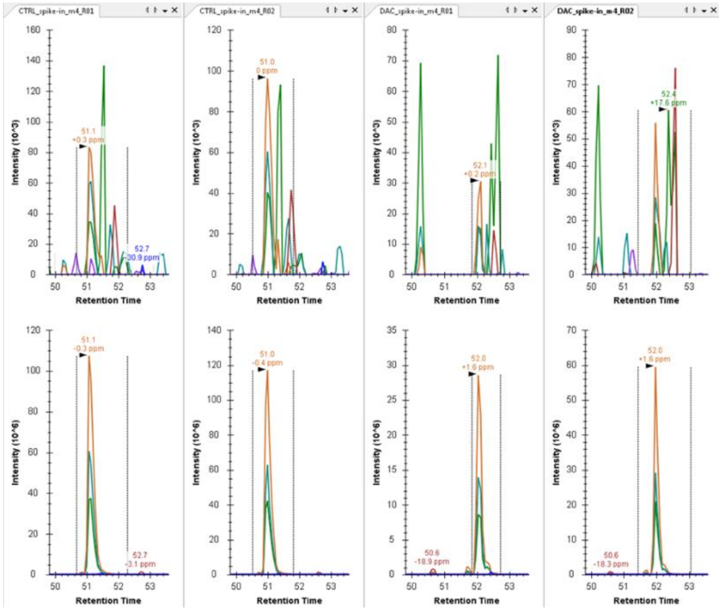
CTRL_spike-in_m4_R01
CTRL_spike-in_m4_R02
DAC_spike-in_m4_R01
DAC_spike-in_m4_R02
Intensity (10^3)
Intensity (10^6)
Retention Time

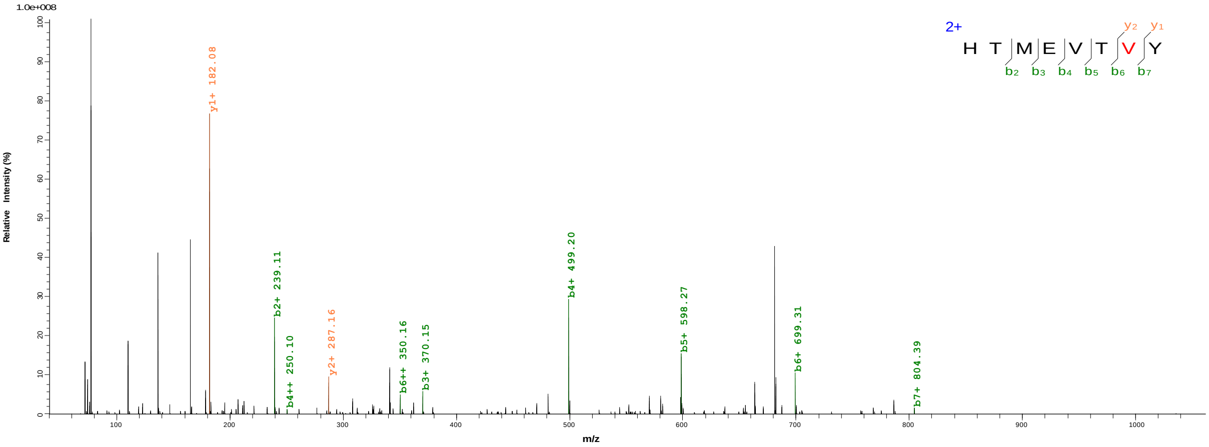
1.0e+008
Relative Intensity (%)
m/z
2+
H T M E V T V Y
y2 y1
b2 b3 b4 b5 b6 b7
y1+ 182.08
b2+ 239.11
b4++ 250.10
y2+ 287.16
b6++ 350.16
b3+ 370.15
b4+ 499.20
b5+ 598.27
b6+ 699.31
b7+ 804.39

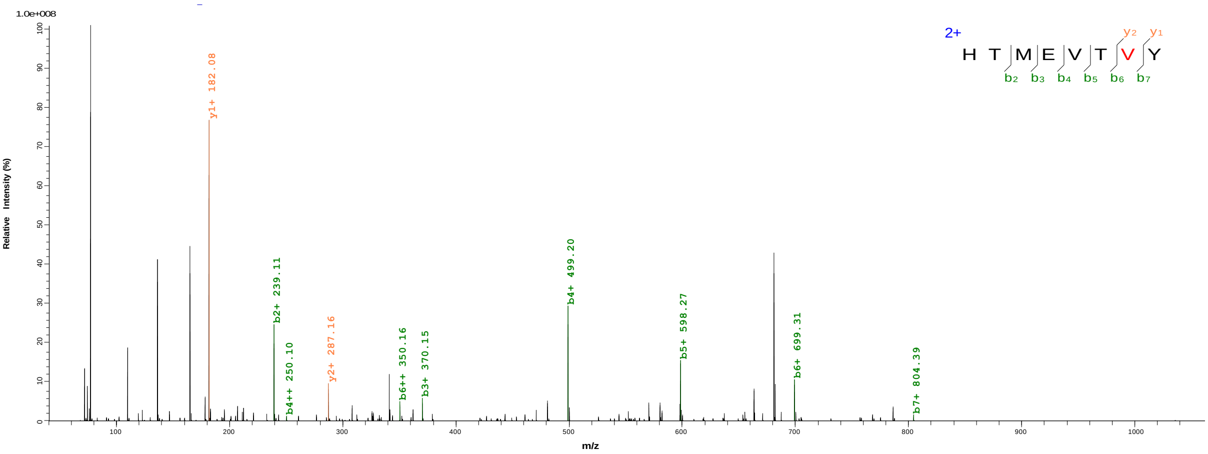
1.0e+008
Relative Intensity (%)
m/z
2+
H T M E V T V Y
y2 y1
b2 b3 b4 b5 b6 b7
y1+ 182.08
b2+ 239.11
b4++ 250.10
y2+ 287.16
b6++ 350.16
b3+ 370.15
b4+ 499.20
b5+ 598.27
b6+ 699.31
b7+ 804.39

FAHEAPAFL

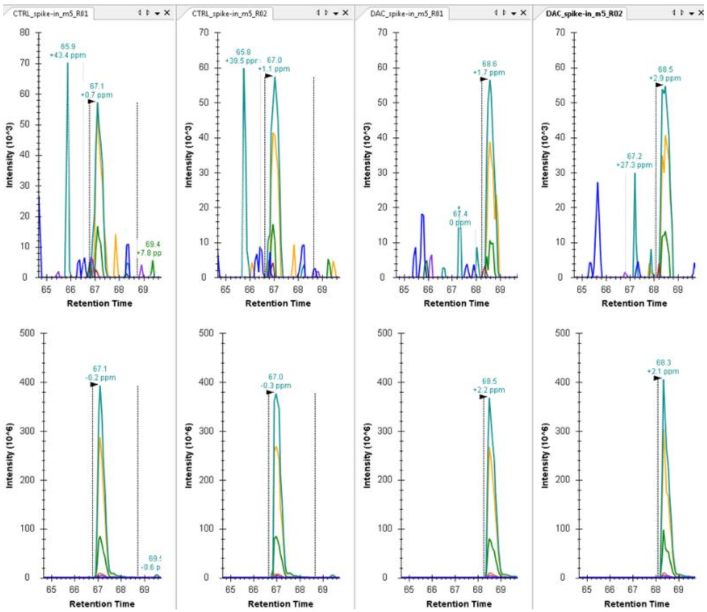

CTRL_spike-in_m5_R01
CTRL_spike-in_m5_R02
DAC_spike-in_m5_R01
DAC_spike-in_m5_R02
Intensity (10^3)
Intensity (10^6)
Retention Time

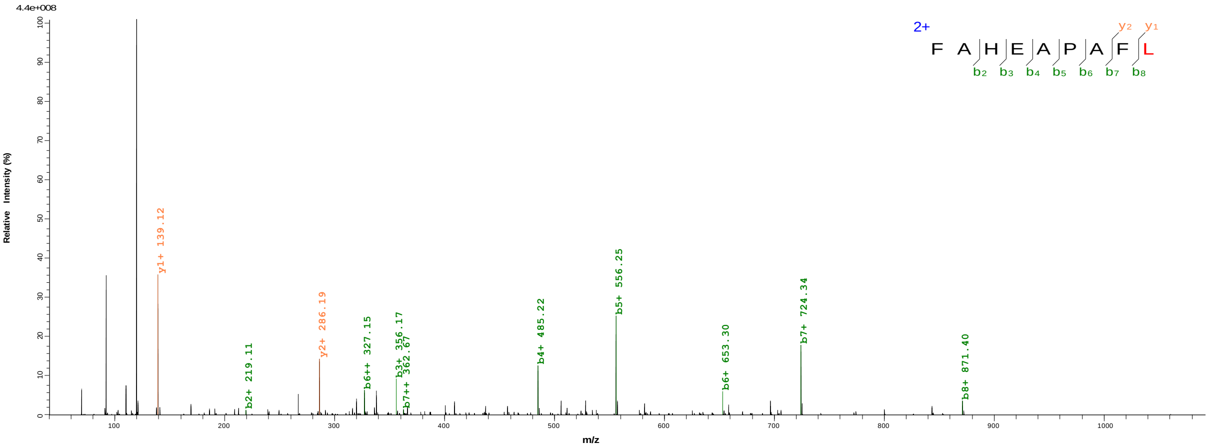

4.4e+008
Relative Intensity (%)
m/z
2+
F A H E A P A F L
y2 y1
b2 b3 b4 b5 b6 b7 b8
y1+ 139.12
b2+ 219.11
y2+ 286.19
b6++ 327.15
b3+ 356.17
b7++ 362.67
b4+ 485.22
b5+ 556.25
b6+ 653.30
b7+ 724.34
b8+ 871.40

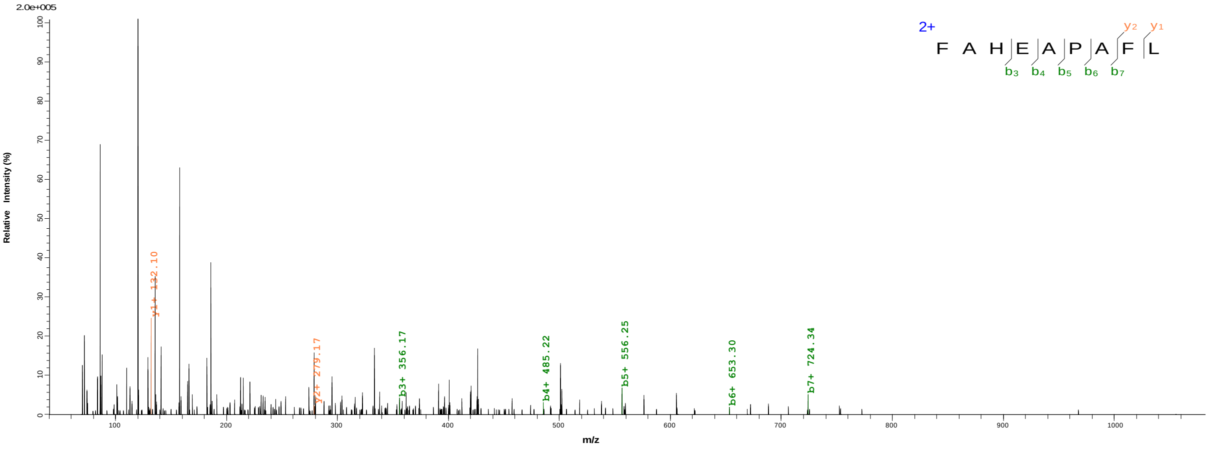

2.0e+005
Relative Intensity (%)
m/z
2+
F A H E A P A F L
y2 y1
b3 b4 b5 b6 b7
y1+ 132.10
y2+ 279.17
b3+ 356.17
b4+ 485.22
b5+ 556.25
b6+ 653.30
b7+ 724.34

ILKGGSGTY

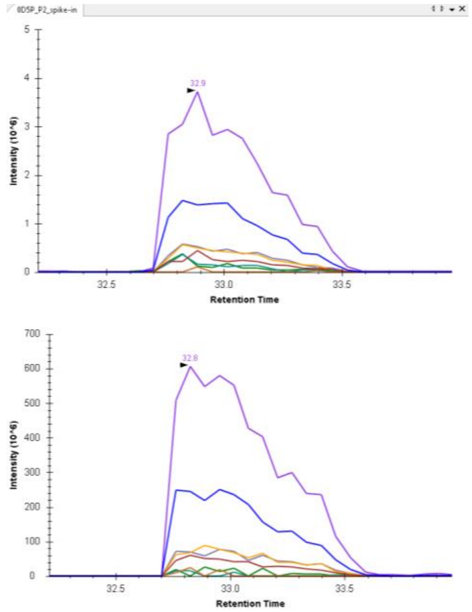

8D5P_P2_spike-in
Intensity (10^6)
Retention Time
32.9
32.8

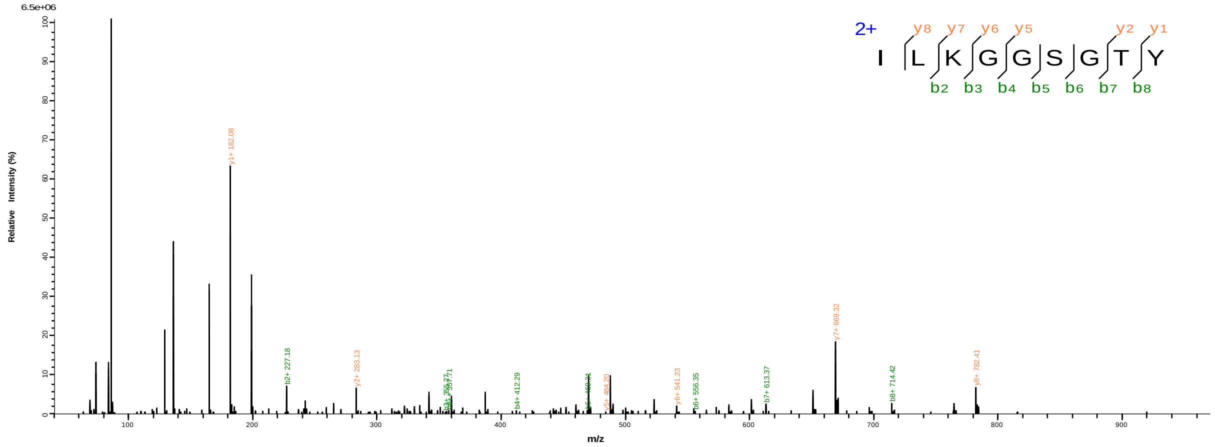

6.5e+06
Relative Intensity (%)
m/z
2+
I L K G G S G T Y
y8 y7 y6 y5 y2 y1
b2 b3 b4 b5 b6 b7 b8
y1+ 182.08
b2+ 227.18
y2+ 283.13
b4+ 412.29
y6+ 541.23
b6+ 556.35
b7+ 613.37
y7+ 669.32
b8+ 714.42
y8+ 782.41

HYYVSMDAL

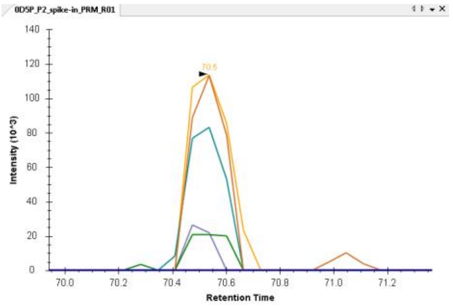

0D5P_P2_spike-in_PRM_R01
Intensity (10^3)
Retention Time
70.6

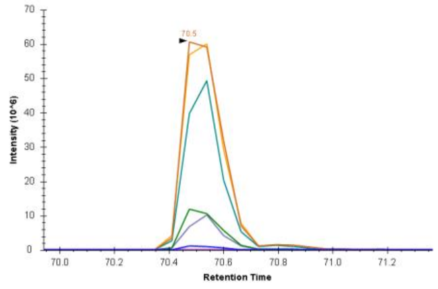

Intensity (10^6)
Retention Time
70.5

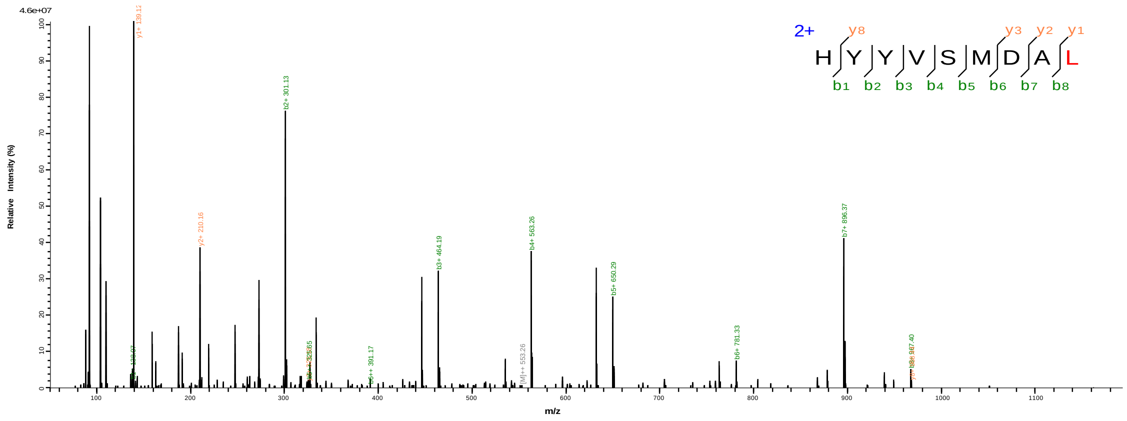

4.6e+07
Relative Intensity (%)
m/z
2+
H Y Y V S M D A L
y8 y3 y2 y1
b1 b2 b3 b4 b5 b6 b7 b8
y1+ 139.11
y2+ 210.16
b2+ 301.13
b3+ 464.19
b4+ 563.26
b5+ 650.29
b6+ 781.33
b7+ 896.37
b8+ 967.40

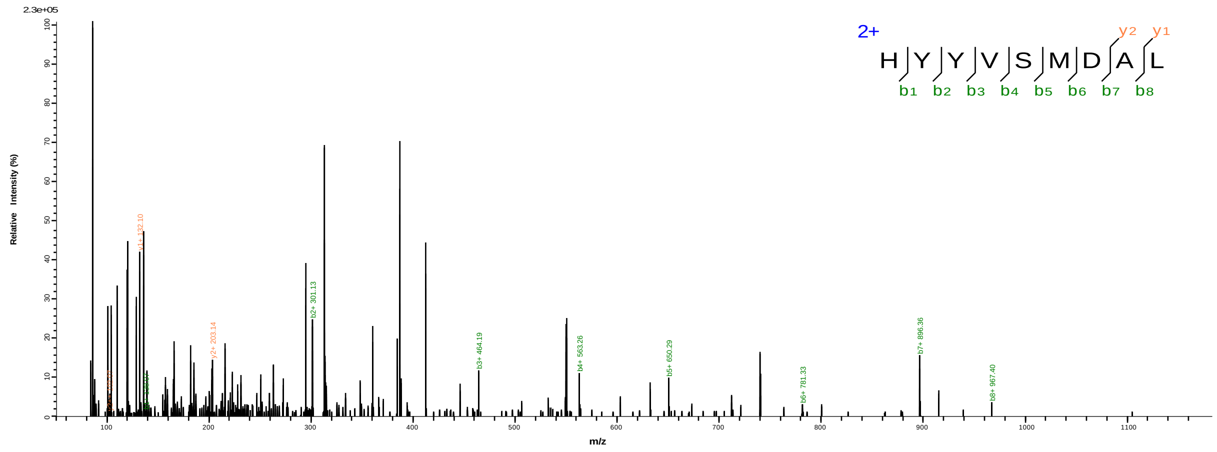

2.3e+05
Relative Intensity (%)
m/z
2+
H Y Y V S M D A L
y2 y1
b1 b2 b3 b4 b5 b6 b7 b8
y1+ 132.10
y2+ 203.14
b2+ 301.13
b3+ 464.19
b4+ 563.26
b5+ 650.29
b6+ 781.33
b7+ 896.36
b8+ 967.40

FSHEGPAF

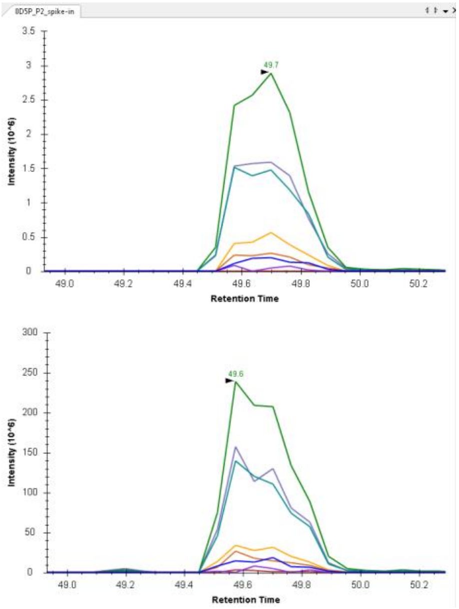

Intensity (10^6)
Retention Time
49.7
49.6

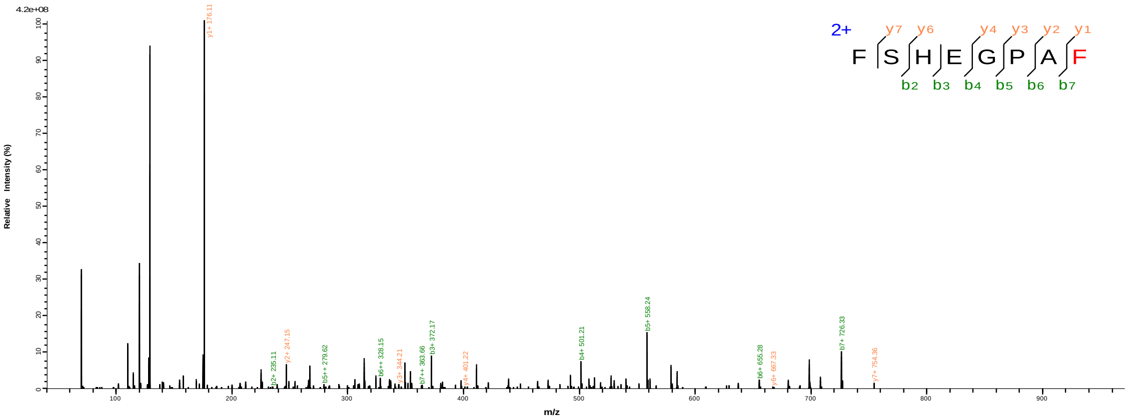

4.2e+08
Relative Intensity (%)
m/z
2+
F S H E G P A F
y7 y6 y4 y3 y2 y1
b2 b3 b4 b5 b6 b7

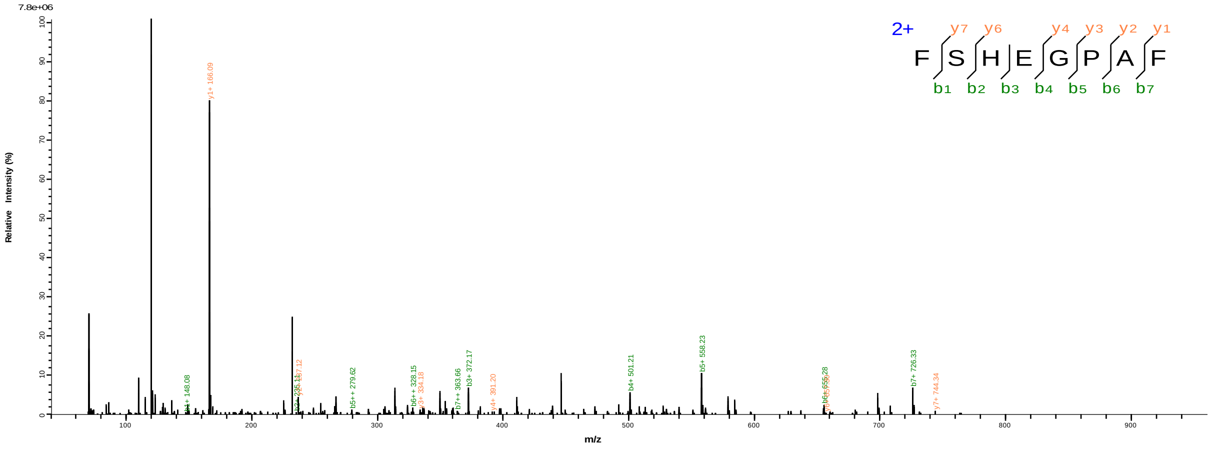

7.8e+06
Relative Intensity (%)
m/z
2+
F S H E G P A F
y7 y6 y4 y3 y2 y1
b1 b2 b3 b4 b5 b6 b7

HYYSVKKTF

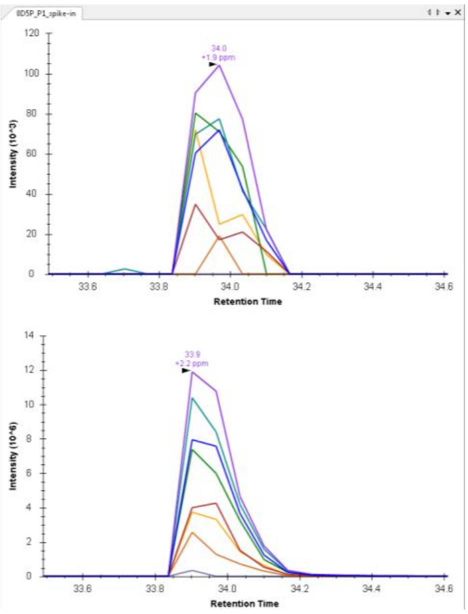

8D5P_P1_spike-in
34.0
+1.9 ppm
Intensity (10^3)
Retention Time
33.9
+2.2 ppm
Intensity (10^6)
Retention Time

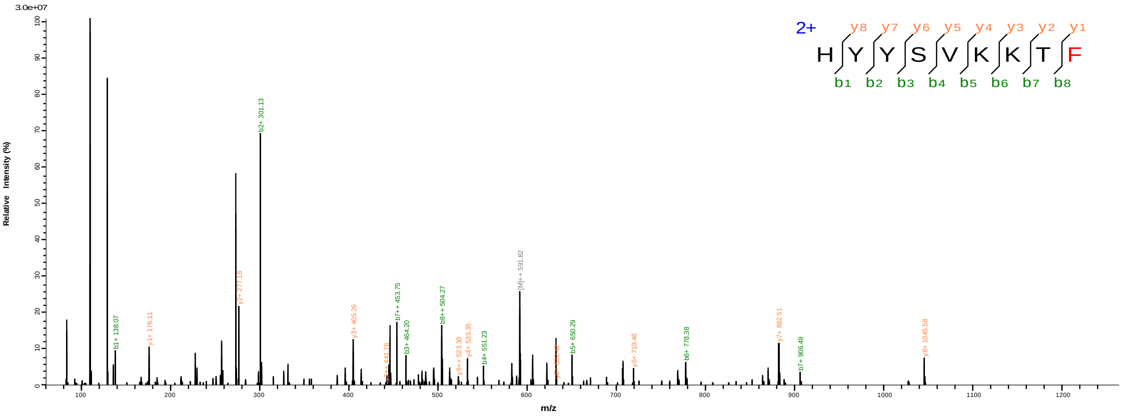

3.0e+07
Relative Intensity (%)
m/z
2+
H Y Y S V K K T F
y8 y7 y6 y5 y4 y3 y2 y1
b1 b2 b3 b4 b5 b6 b7 b8
b2+ 301.13
[M]++ 591.82

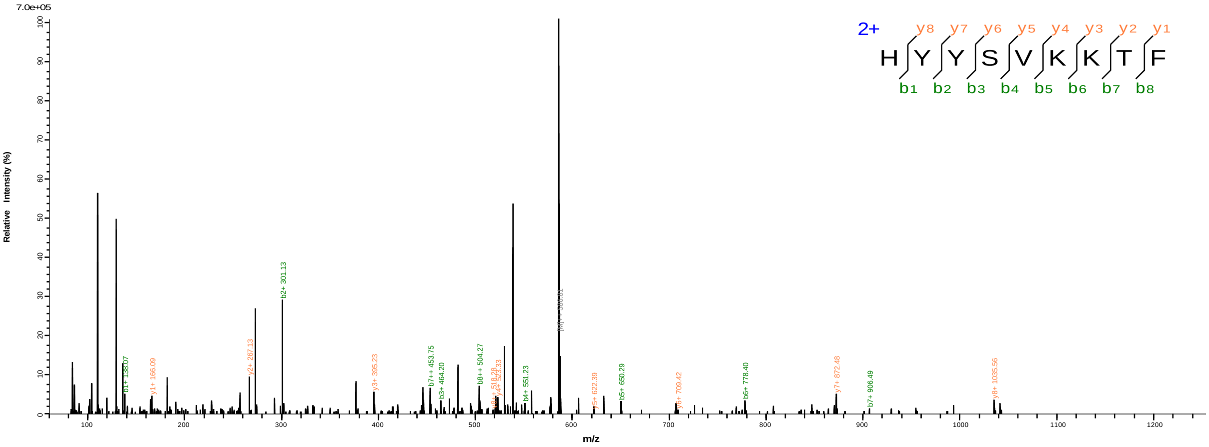

7.0e+05
Relative Intensity (%)
m/z
2+
H Y Y S V K K T F
y8 y7 y6 y5 y4 y3 y2 y1
b1 b2 b3 b4 b5 b6 b7 b8
b2+ 301.13

KVFKNGNAF

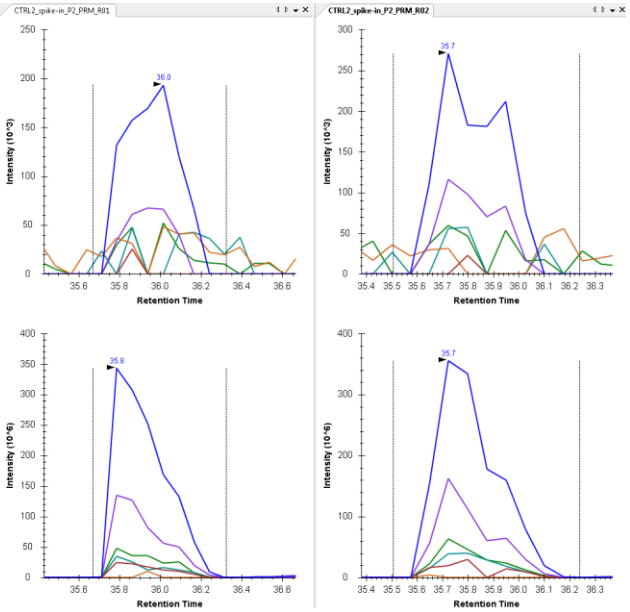
CTRL2_spike-in_P2_PRM_R01
CTRL2_spike-in_P2_PRM_R02
Intensity (10^3)
Intensity (10^6)
Retention Time
36.0
35.7
35.8
35.7

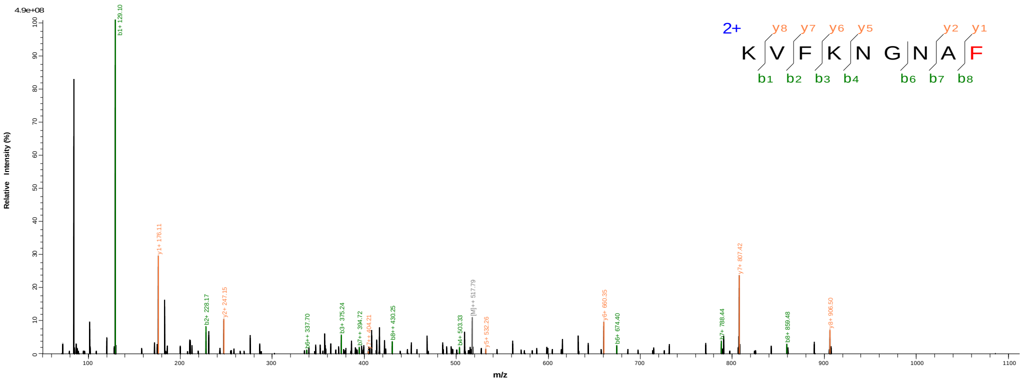
4.9e+08
Relative Intensity (%)
m/z
b1+ 129.10
y1+ 176.11
y7+ 807.42
2+
K V F K N G N A F
y8 y7 y6 y5 y2 y1
b1 b2 b3 b4 b6 b7 b8

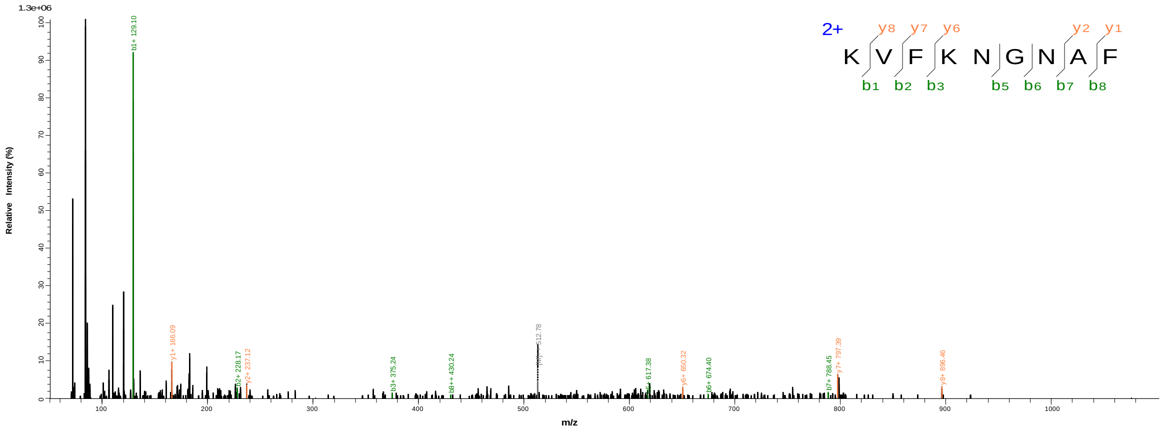
1.3e+06
Relative Intensity (%)
m/z
b1+ 129.10
y1+ 166.09
2+
K V F K N G N A F
y8 y7 y6 y2 y1
b1 b2 b3 b5 b6 b7 b8

NMDGPGGHY

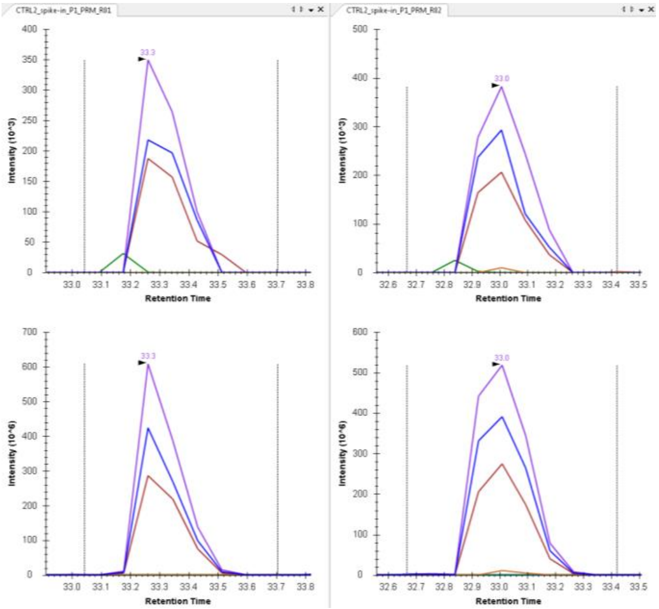
CTRL2_spike-in_P1_PRM_R01
CTRL2_spike-in_P1_PRM_R02
Intensity (10^3)
Intensity (10^6)
Retention Time
33.3
33.0

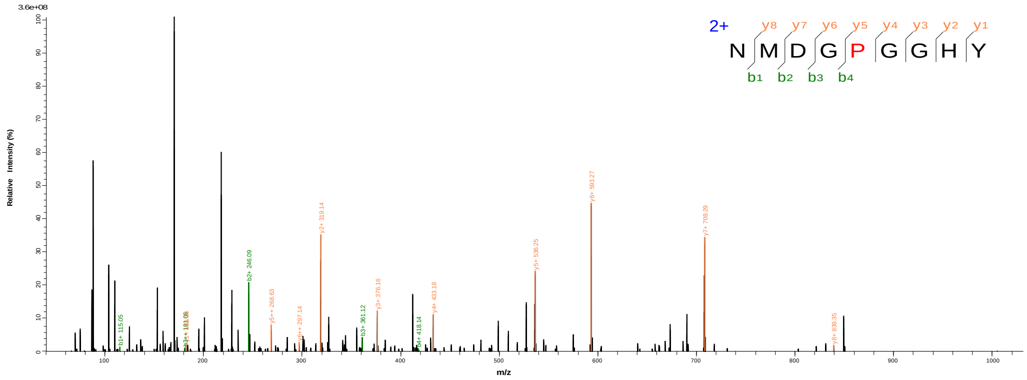
3.6e+08
Relative Intensity (%)
m/z
2+
y8 y7 y6 y5 y4 y3 y2 y1
N M D G P G G H Y
b1 b2 b3 b4

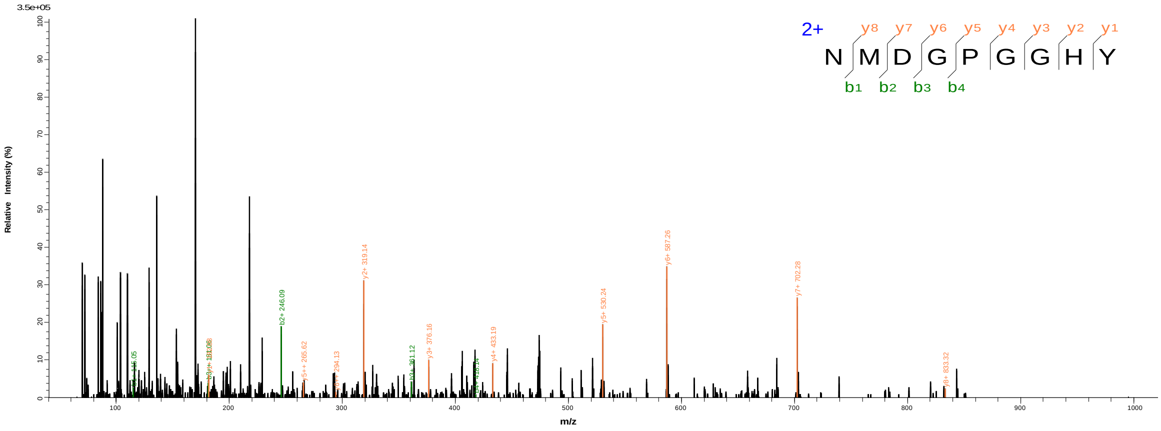
3.5e+05
Relative Intensity (%)
m/z
2+
y8 y7 y6 y5 y4 y3 y2 y1
N M D G P G G H Y
b1 b2 b3 b4

AAFDRAVHF

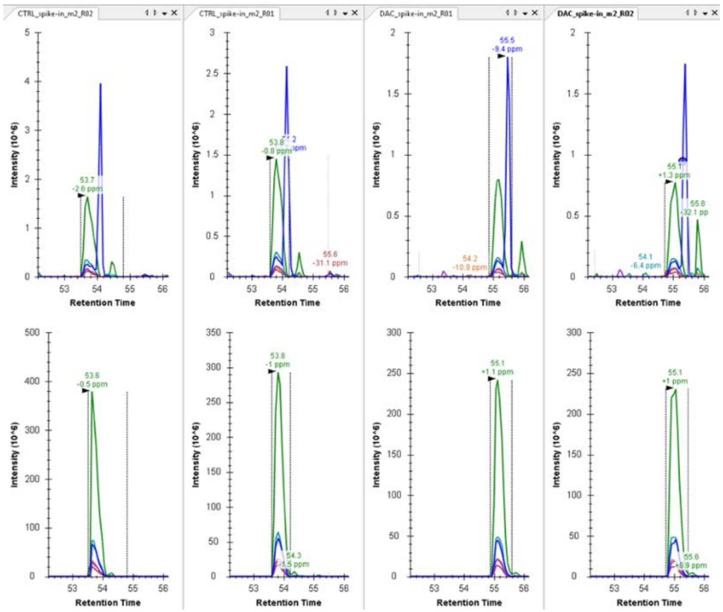
CTRL_spike-in_m2_R02
CTRL_spike-in_m2_R01
DAC_spike-in_m2_R01
DAC_spike-in_m2_R02
Intensity (10^6)
Retention Time

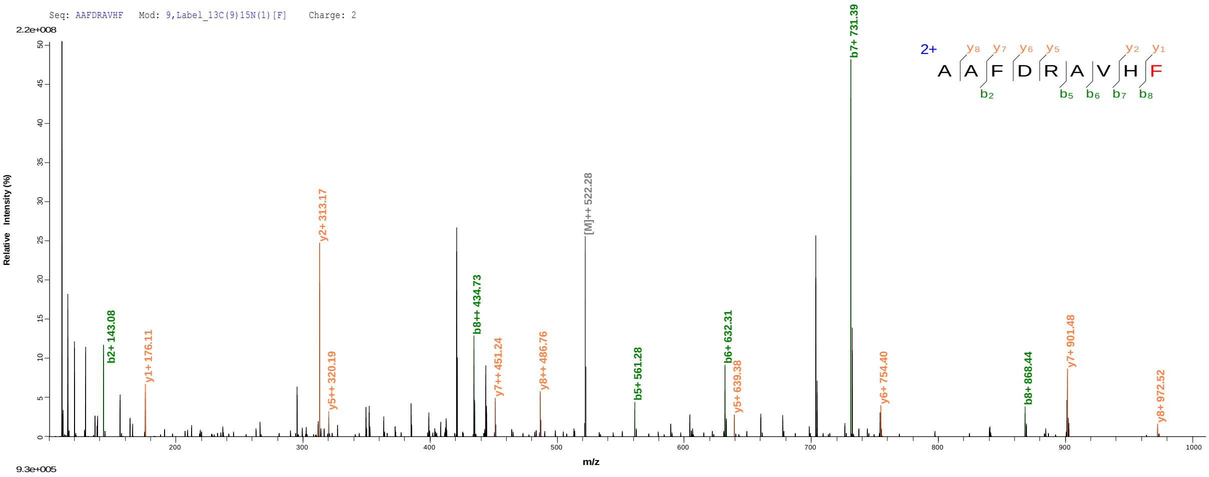
Seq: AAFDRAVHF Mod: 9,Label_13C(9)15N(1)[F] Charge: 2
2.2e+008
Relative Intensity (%)
m/z
b7+ 731.39
[M]++ 522.28
y2+ 313.17
b8++ 434.73
b2+ 143.08
y1+ 176.11
y5++ 320.19
y7++ 451.24
y8++ 486.76
b5+ 561.28
b6+ 632.31
y5+ 639.38
y6+ 754.40
b8+ 868.44
y7+ 901.48
y8+ 972.52
2+
A A F D R A V H F

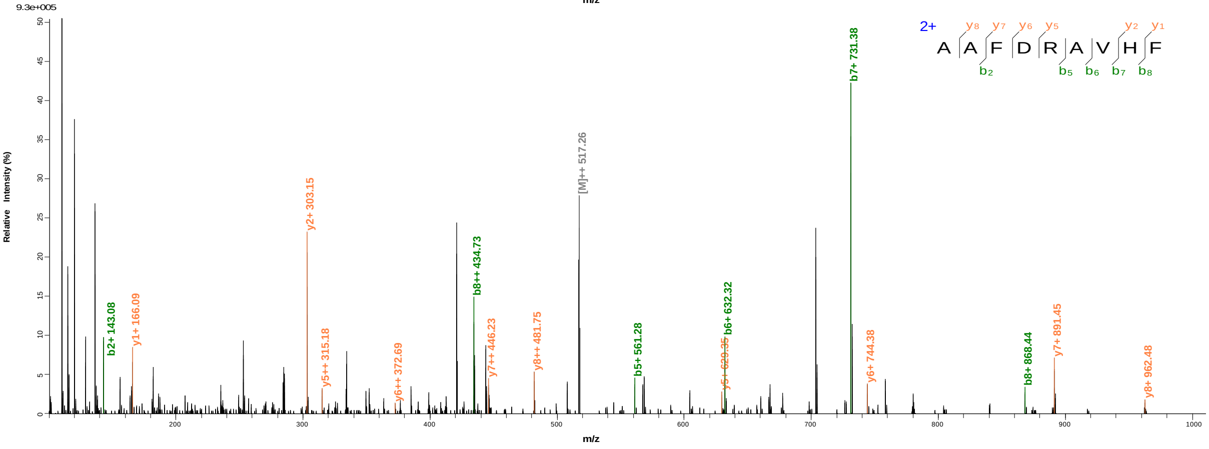
9.3e+005
Relative Intensity (%)
m/z
b7+ 731.38
[M]++ 517.26
y2+ 303.15
b8++ 434.73
b2+ 143.08
y1+ 166.09
y5++ 315.18
y6++ 372.69
y7++ 446.23
y8++ 481.75
b5+ 561.28
b6+ 632.32
y6+ 744.38
b8+ 868.44
y7+ 891.45
y8+ 962.48
2+
A A F D R A V H F

ETDIEMETRY – C3N-02671 tumor

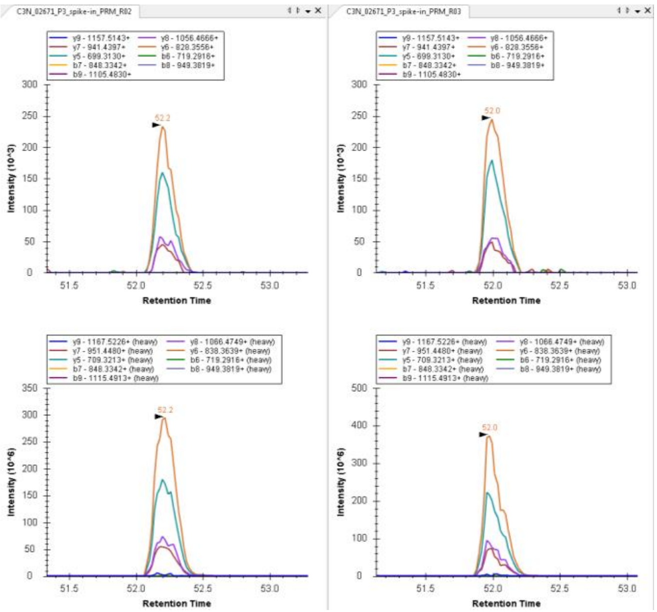

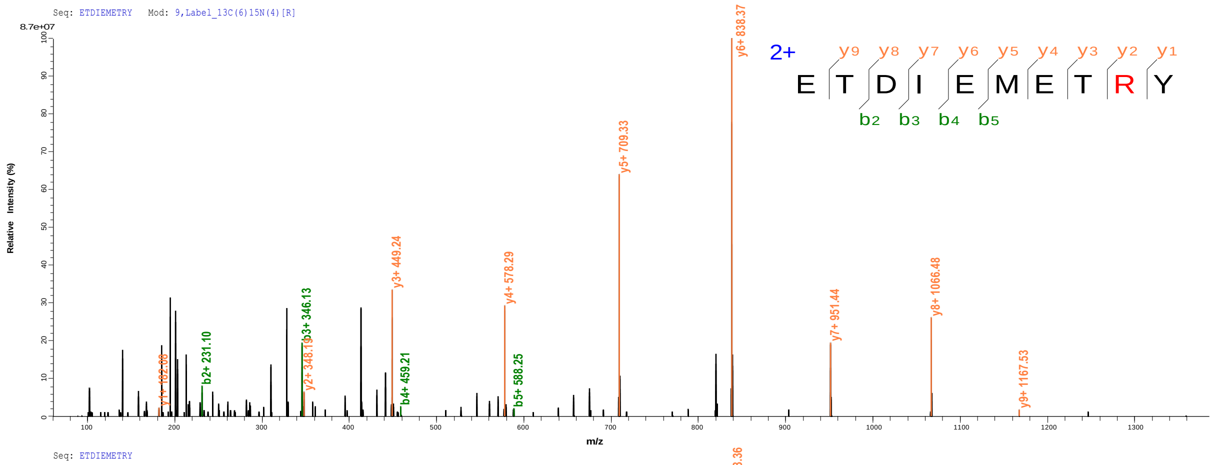

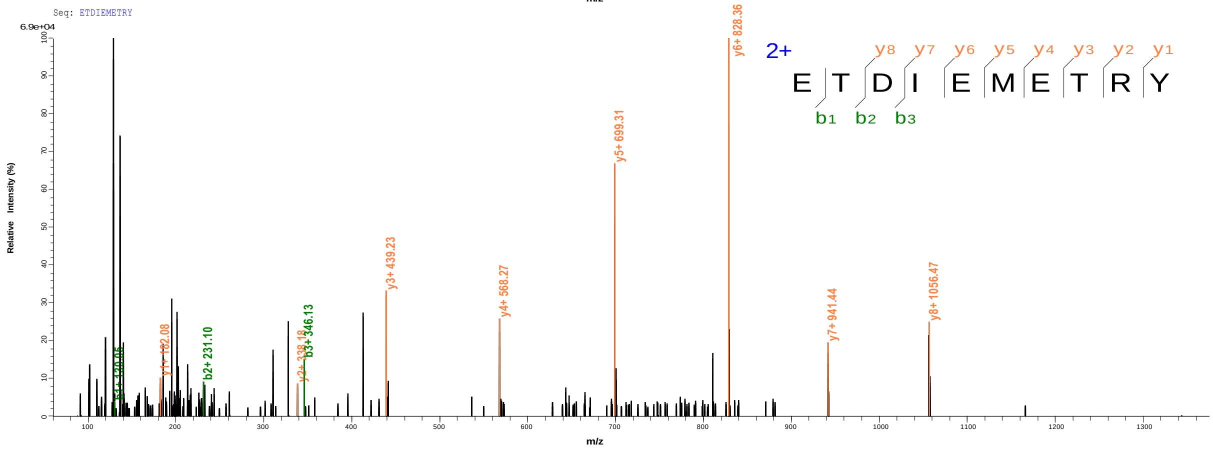

KSDLSKPLSY – C3N-02671 tumor

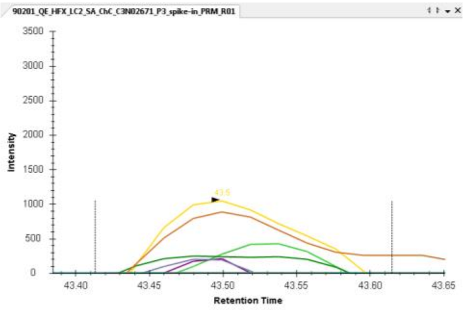

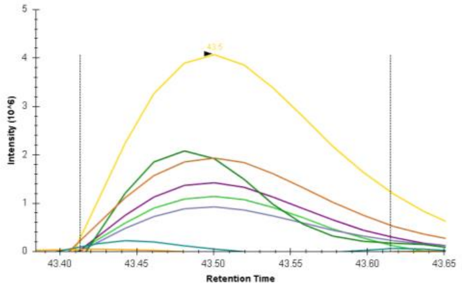

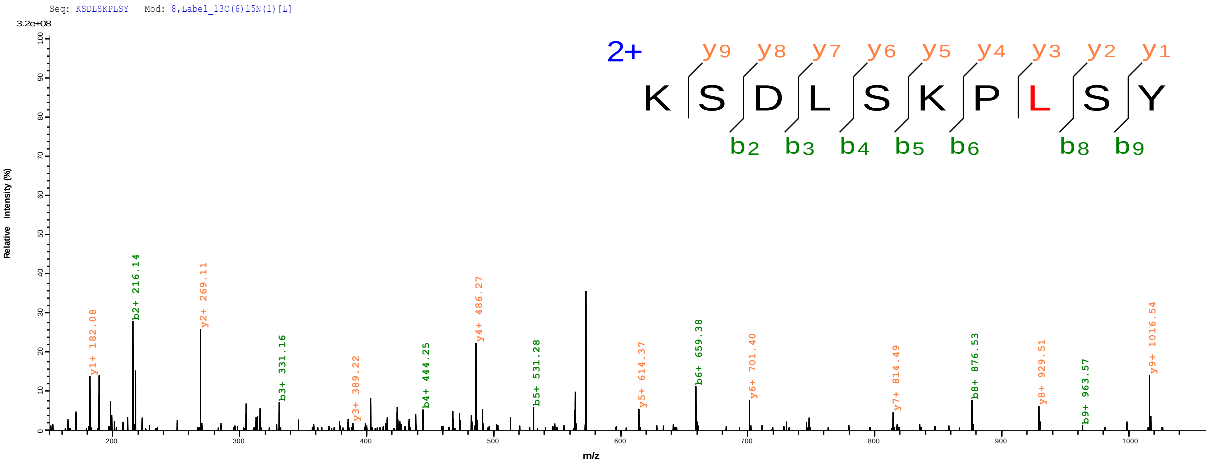

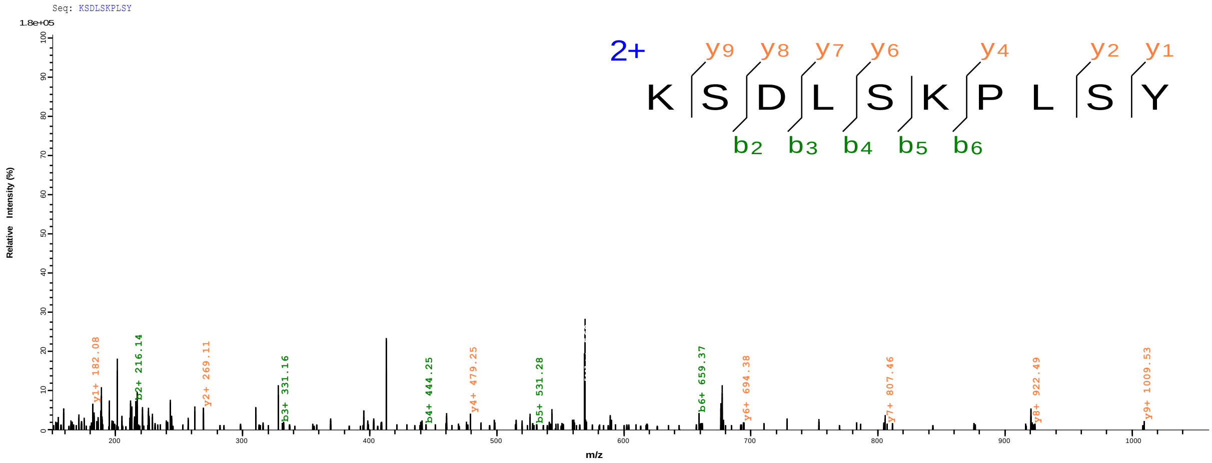

YLDPAQQNLY – C3N-02671 tumor

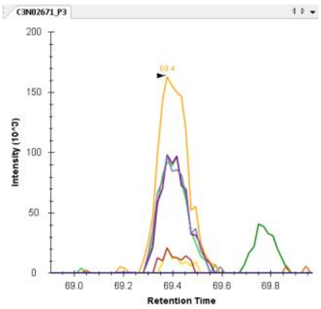

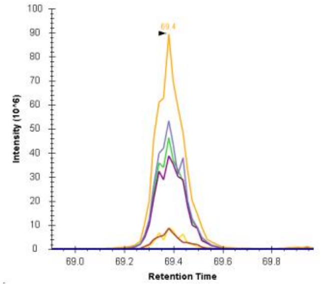

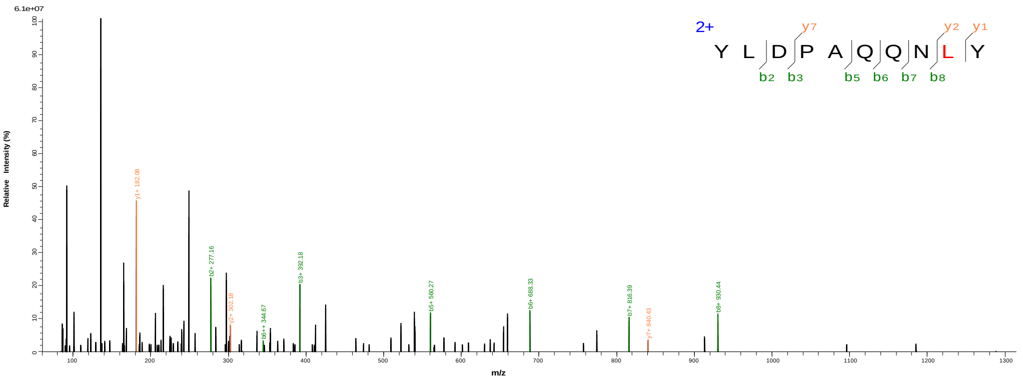

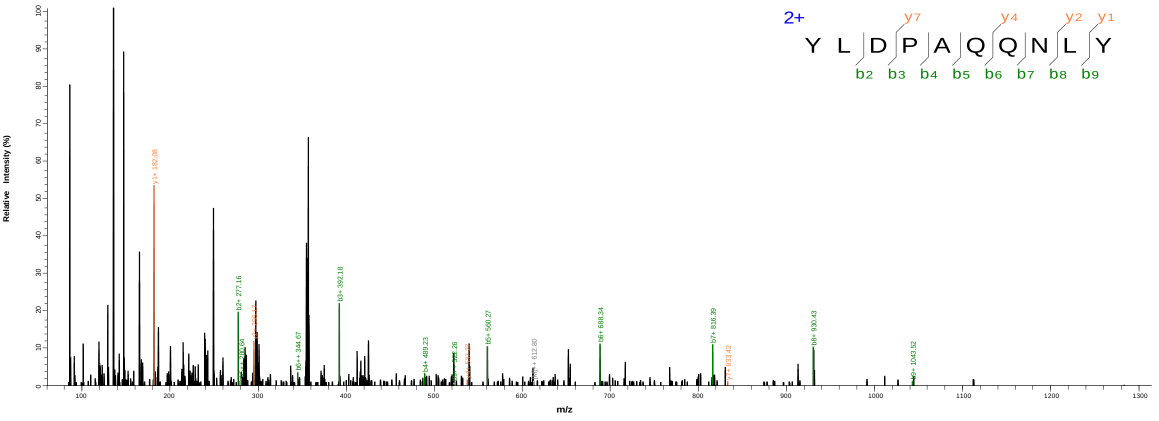

VTDQASHIY – C3N-02671 tumor

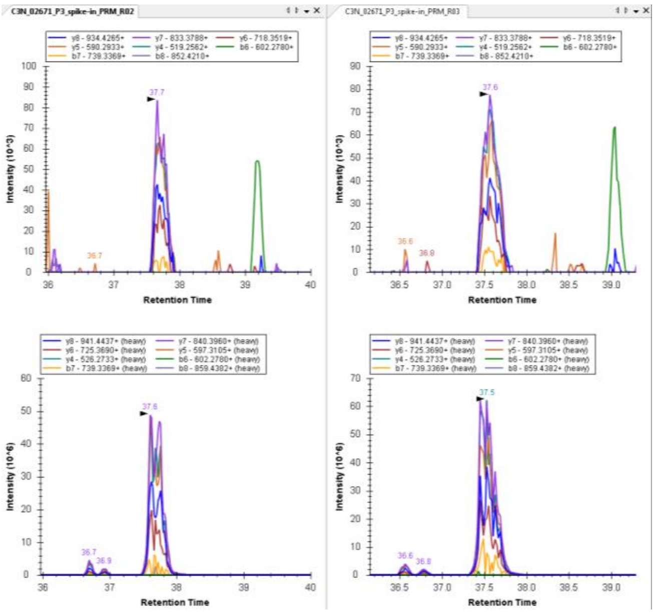

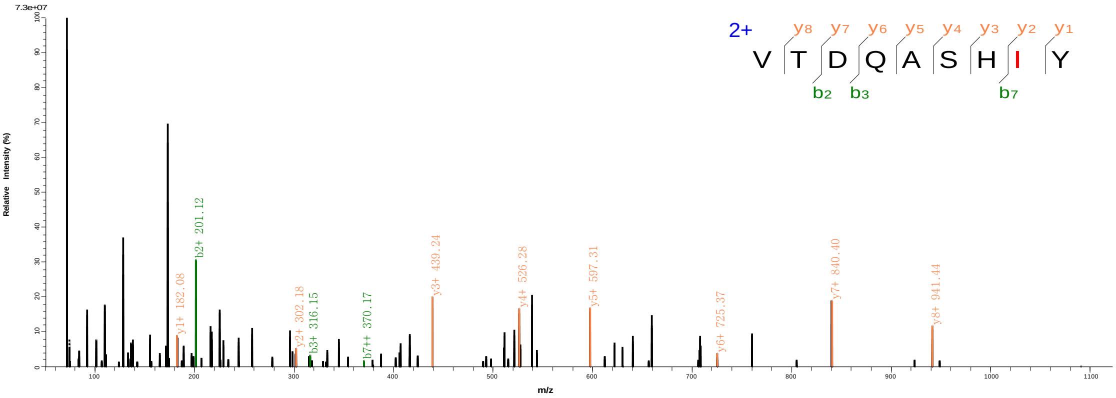

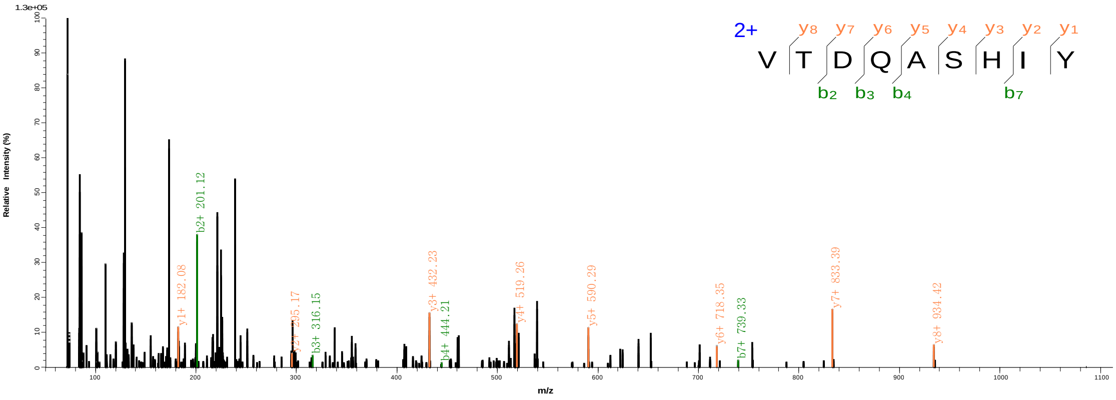

VTDQASHIY – 0MM745

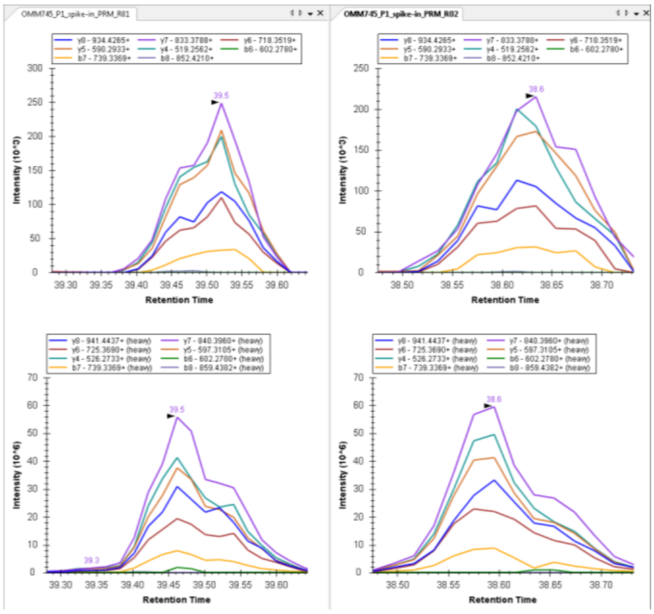

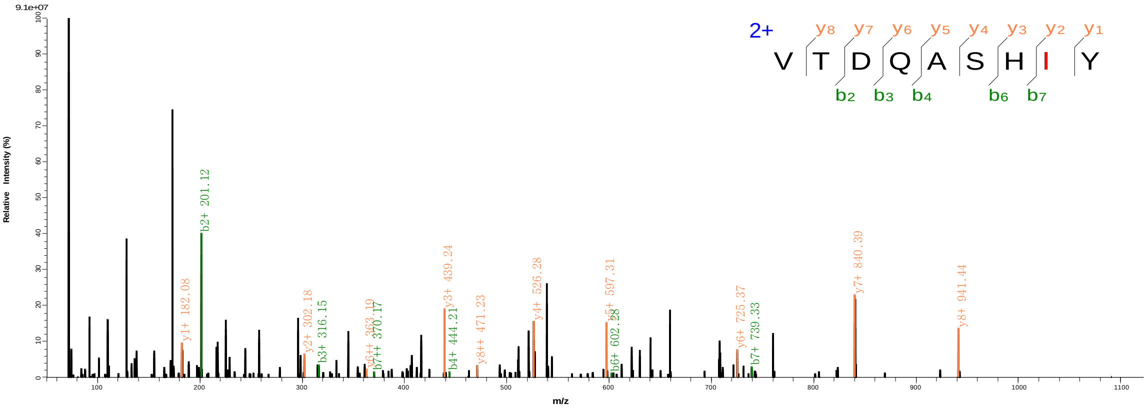

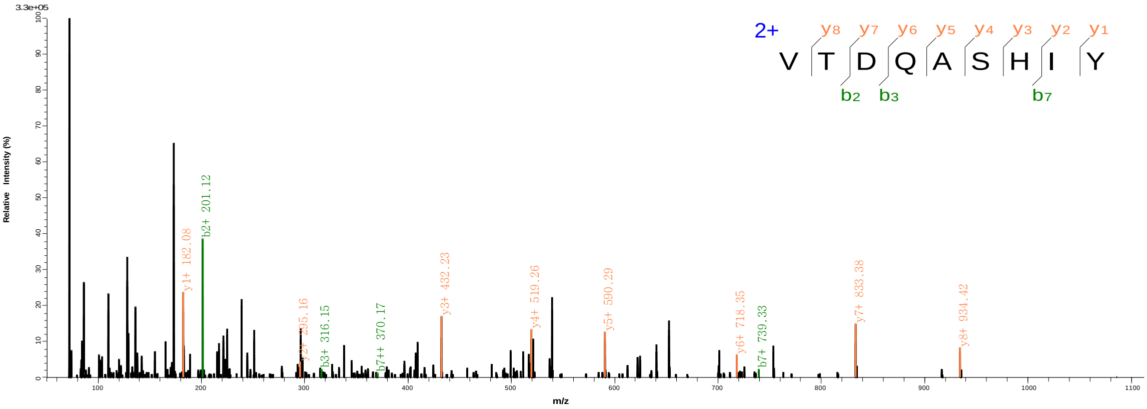

AAFDRAVHF – 0NVC

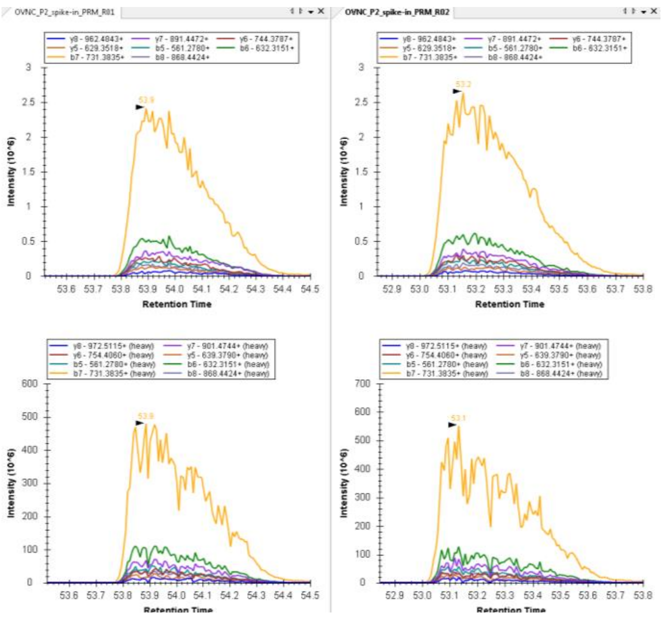

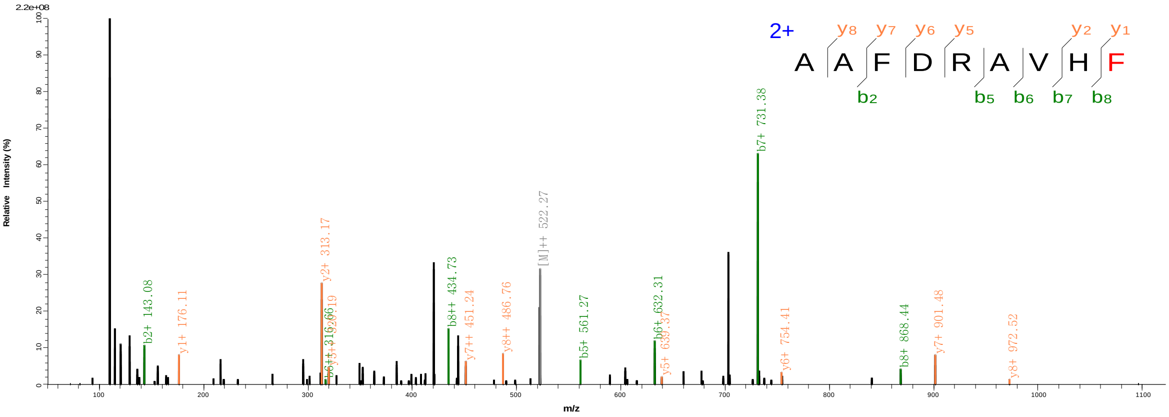

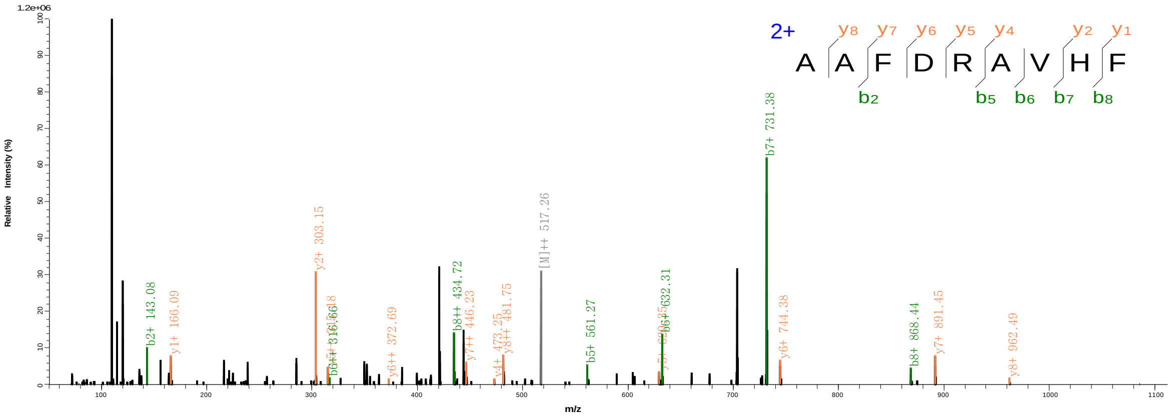

KVFKNGNAF – T1015A

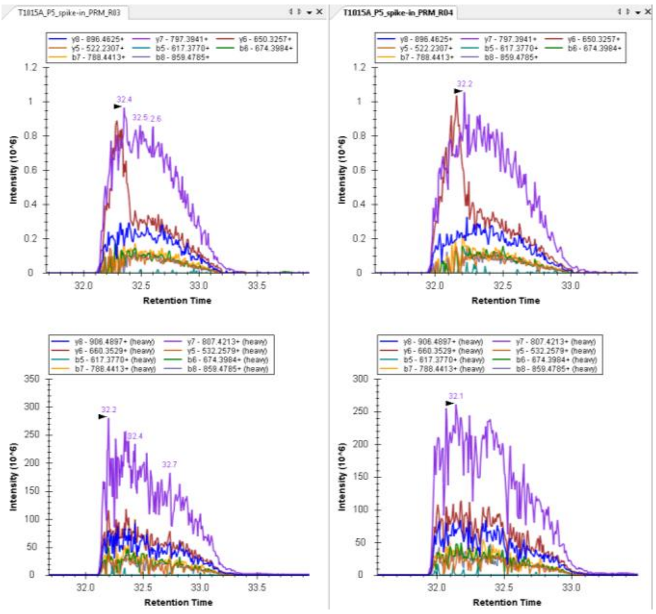

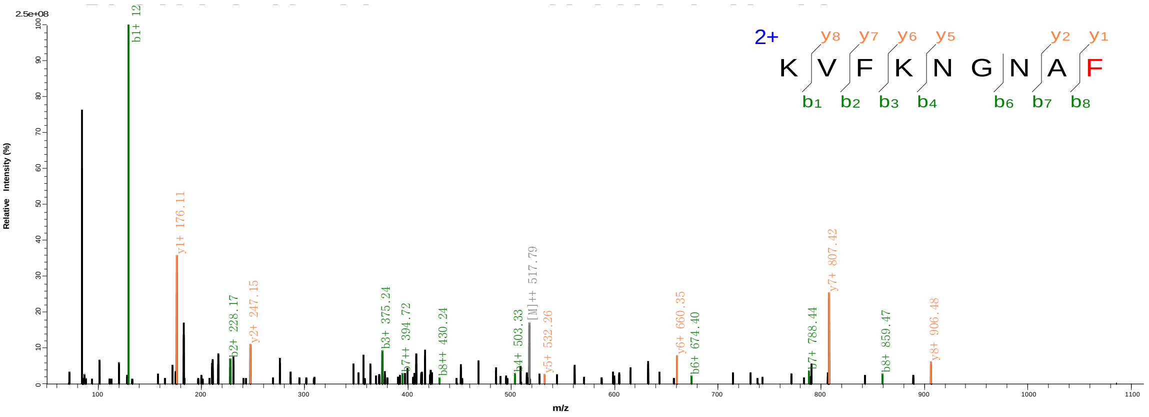

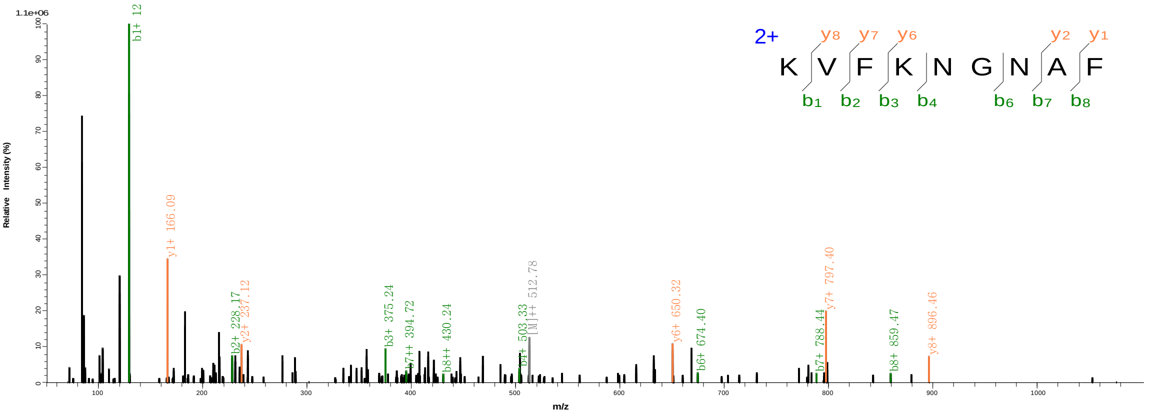

APKSSSGFSL – Me275

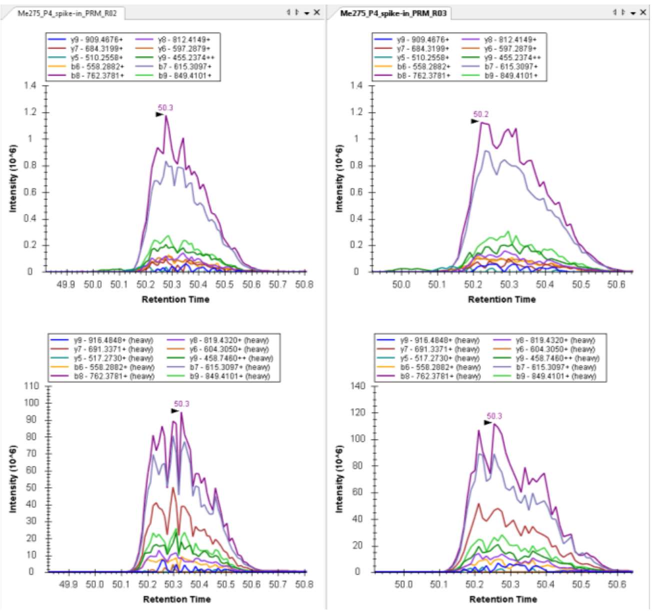

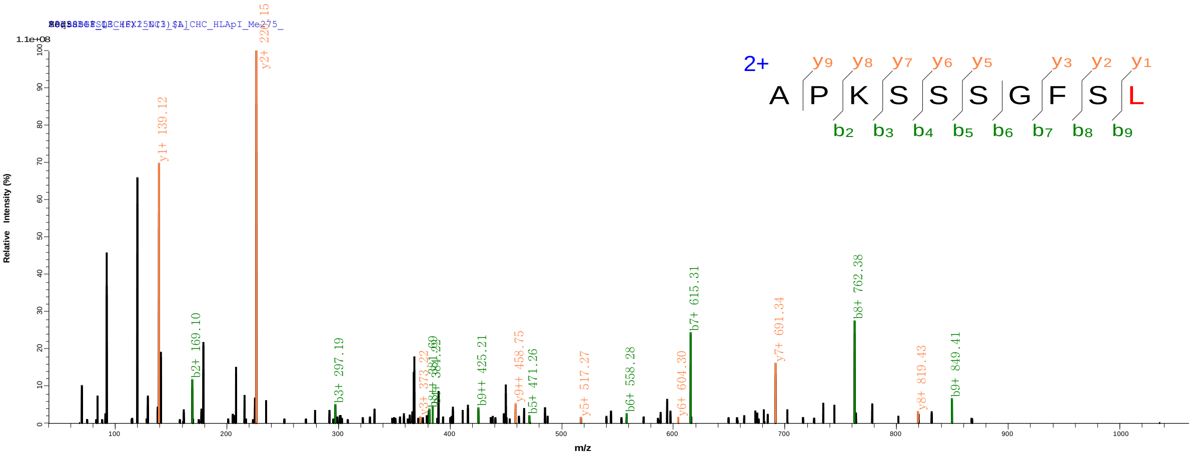

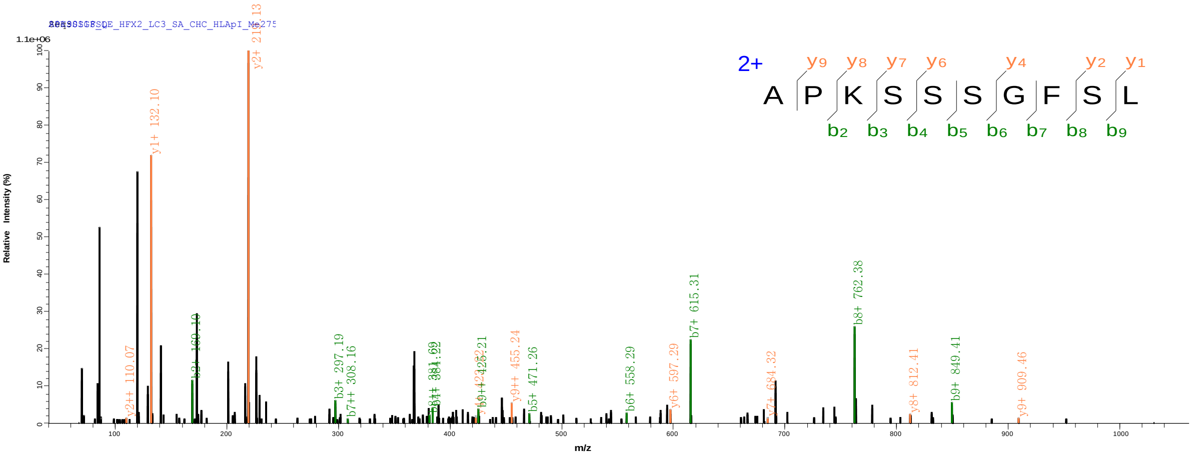

Supplement: Supplementary file 9 — Supplementary Data 7 [file 41467_2020_14968_MOESM9_ESM.pdf]
