# Supplementary material for: Integrated proteogenomic deep sequencing and analytics accurately identify non-canonical peptides in tumor immunopeptidomes
Source: Nat Commun. 2020 Mar 10;11:1293. doi: 10.1038/s41467-020-14968-9 (PMC7064602; doi:10.1038/s41467-020-14968-9)
Supplement: Supplementary file 11 — Reporting Summary [file 41467_2020_14968_MOESM11_ESM.pdf]

natureresearch

Corresponding author(s): Dr. Michal Bassani-Sternberg

Last updated by author(s): 2020/02/07

Reporting Summary

Nature Research wishes to improve the reproducibility of the work that we publish. This form provides structure for consistency and transparency in reporting. For further information on Nature Research policies, see Authors & Referees and the Editorial Policy Checklist .

Statistics

For all statistical analyses, confirm that the following items are present in the figure legend, table legend, main text, or Methods section.

n/a	Confirmed	
	x	The exact sample size (*n*) for each experimental group/condition, given as a discrete number and unit of measurement
	x	A statement on whether measurements were taken from distinct samples or whether the same sample was measured repeatedly
	x	The statistical test(s) used AND whether they are one- or two-sided<br>*Only common tests should be described solely by name; describe more complex techniques in the Methods section.*
x		A description of all covariates tested
	x	A description of any assumptions or corrections, such as tests of normality and adjustment for multiple comparisons
x		A full description of the statistical parameters including central tendency (e.g. means) or other basic estimates (e.g. regression coefficient) AND variation (e.g. standard deviation) or associated estimates of uncertainty (e.g. confidence intervals)
	x	For null hypothesis testing, the test statistic (e.g. *F*, *t*, *r*) with confidence intervals, effect sizes, degrees of freedom and *P* value noted<br>*Give P values as exact values whenever suitable.*
x		For Bayesian analysis, information on the choice of priors and Markov chain Monte Carlo settings
x		For hierarchical and complex designs, identification of the appropriate level for tests and full reporting of outcomes
x		Estimates of effect sizes (e.g. Cohen's *d*, Pearson's *r*), indicating how they were calculated

Our web collection on statistics for biologists contains articles on many of the points above.

Software and code

Policy information about availability of computer code

Data collection	Softwares used in this study are publically available and stated in the Methods section where applicable.<br>- DNA and RNA sequencing<br>- bcl2fastq Conversion Software (v. 1.84, Illumina)<br>- bcl2fastq2w Conversion Software (v.2.20, Illumina)
Data analysis	Softwares used in this study are publically available and stated in the Methods section where applicable.<br>-PRM: Skyline v4.1.0.18169, Proteowizard v3.0.18136, pLabel v2.4.0.8<br>-Exome analyses: GenomeAnalysisTK (GATK) v3.7, Picard Tools v 2.9.0<br>-HLA typing: Assign TruSight software v2.1<br>-RNA sequencing analyses: RNA-Star v2.4.2a, Cufflinks v2.2.1, GTEx v7<br>-MS analyses: MaxQuant v1.5.9.4i, Comet 2017.01 rev. 2, Apache Spark cluster computing framework<br>-HLA-peptide binding prediction: MixMHCpred v2<br>-Ribo-Seq analyses: Bowtie v.2.3.5, STAR 2.6.1a_08-27, SaTAnn v1.0, RiboseQC v1.0<br>-TE analyses: hisat2 v2.1.0, featureCounts v1.6.2, limma v3.36.5<br>-scRNAseq analyses: Cell Ranger v.3.0.1, Seurat v3<br>-General: GraphPad Prism 8, Perseus 1.5.5.3, RStudio 3.5.1 and Python 3.6, STRING-db v11, GTEx v7, ComplexHeatmap v1.99.4, SSRCalc vQ.0<br>-NewAnce: The NewAnce code is available on the following GitHub link: https://github.com/bassanilab/NewAnce.git

For manuscripts utilizing custom algorithms or software that are central to the research but not yet described in published literature, software must be made available to editors/reviewers. We strongly encourage code deposition in a community repository (e.g. GitHub). See the Nature Research guidelines for submitting code & software for further information.

Data

Policy information about availability of data

All manuscripts must include a data availability statement. This statement should provide the following information, where applicable:

- Accession codes, unique identifiers, or web links for publicly available datasets
- A list of figures that have associated raw data
- A description of any restrictions on data availability

Sequence data have been deposited into the European Genome-phenome Archive (EGA), which is hosted by the EBI and the CRG, under accession numbers EGAS00001003723 and EGAS00001003724. MS raw files, corresponding fasta reference files and NewAnce outputs have been deposited into the ProteomeXchange Consortium via the PRIDE partner repository with the dataset identifier PXD013649. Databases can be accessed through: The GENCODE v221.2 from https://www.gencodegenes.org/releases/22.html. The human reference genome GRCh38/hg38 from http://hgdownload.cse.ucsc.edu/goldenPath/hg38/bigZips/. Human ENCODE non-coding transcripts from https://www.gencodegenes.org/human/release_24lift37.html. GTEx v7 from https://www.gtexportal.org/home/datasets. The UniProt/TrEMBL database from https://www.uniprot.org/uniprot/?query=*&fil=proteome%3AUP000005640+AND+organism%3A%22Homo+sapiens+%28Human%29+%5B9606%5D%22. The source data underlying the Figures and Supplementary Figures, where applicable, are provided as a Source Data file.

Field-specific reporting

Please select the one below that is the best fit for your research. If you are not sure, read the appropriate sections before making your selection.

☒ Life sciences ☐ Behavioural & social sciences ☐ Ecological, evolutionary & environmental sciences

For a reference copy of the document with all sections, see nature.com/documents/nr-reporting-summary-flat.pdf

Life sciences study design

All studies must disclose on these points even when the disclosure is negative.

Sample size	No statistical methods were used to determine sample size for proteogenomics analyses. Ultimately, sample size was determined by the access and availability of patient samples and patient material, as well as lab capacity. Sample size is sufficient for this proof-of-concept study showing that tumor non-canonical peptides can be identified for every patient.
Data exclusions	No data was excluded
Replication	Mass spectrometry discovery based studies were replicated at least twice (please see Supplementary Data 1 for detailed information on replicates for MS-based analyses), and were reproducible. Sequencing data has not been replicated due to the significant cost of additional sequencing, which were determined to not bring additional value to the results presented. T cell based assays were replicated at least twice and the data can be reproduced.
Randomization	Randomization was not applied in these experiments as this was not applicable to our present study. This is due to the fact that it was a proof-of-concept exploratory research with a small set of samples. The study was solely based on identifying non-canonical antigens in immunopeptidomics data.
Blinding	The study was not blinded as this is not applicable/relevant to our current study to identify non-canonical HLAp. This is due to the fact that it was a proof-of-concept exploratory research with a small set of samples. The study was solely based on identifying non-canonical antigens in immunopeptidomics data.

Reporting for specific materials, systems and methods

We require information from authors about some types of materials, experimental systems and methods used in many studies. Here, indicate whether each material, system or method listed is relevant to your study. If you are not sure if a list item applies to your research, read the appropriate section before selecting a response.

Materials & experimental systems

n/a	Involved in the study	
☐	☒	Antibodies
☐	☒	Eukaryotic cell lines
☒	☐	Palaeontology
☒	☐	Animals and other organisms
☐	☒	Human research participants
☒	☐	Clinical data

Methods

n/a	Involved in the study	
☒	☐	ChIP-seq
☒	☐	Flow cytometry
☒	☐	MRI-based neuroimaging

Antibodies

Antibodies used

Anti HLA-I antibody : from hybridoma "HB-95"

Company name : ATCC
Catalog number : HB-95
Lot number: 7001294
Clone name: W6/32
Antigenic determinant: HLA-A, B, C
Isotype: IgG2a
Host: mouse
Cell type: Hybridoma: B lymphocyte
Clonality: monoclonal

Anti HLA-II antibody : from hybridoma "HB-145"

Company name : ATCC
Catalog number : HB-145
Lot number:59681660
Clone name: iva12
Antigenic determinant: HLA DR, DP, DQ
Isotype: IgG1; kappa light chain
Host: mouse
Cell type: Hybridoma: B lymphocyte
Clonality: monoclonal

- IFNgamma ELISPOT assay (Mabtech)

- CD3 antibody
Company name : Miltenyi Biotec
Catalog number : 170-076-124
Antigen: CD3
Clone: OKT3
Isotype: mouse IgG2aκ
Alternative names of antigen: CD3e, IMD18, T3E, TCRE

Validation

Validation by vendor following ATCC guidelines. Certificate of Analysis can be found here:https://www.lgcstandards-atcc.org/Products/All/HB-95.aspx?geo_country=ch#documentation and https://www.lgcstandards-atcc.org/Products/All/HB-145.aspx?geo_country=ch#documentation
Additionally, anti-HLA-I and -II antibodies were validated directly in our laboratory, through the use of these antibodies for immuno-affinity purification of HLA-I and -II peptides from cell lines and tissue samples. These peptides were measured by mass spectrometry, and their characteristics fit that of HLA-I and -II peptides, respectively.

For the CD3 antibody, validation was performed by the vendor following Miltenyi guidelines: https://www.miltenyibiotec.com/CH-en/products/cell-manufacturing-platform/macs-gmp-portfolio/activation-and-expansion-tools/macs-gmp-cd3-pure-okt3.html#0-2-mg-in-1-ml

Eukaryotic cell lines

Policy information about cell lines

Cell line source(s)

Melanoma cell lines (0D5P, 0MM745, 0NVC) were provided by the Center of Experimental Therapy CHUV, Lausanne.

Melanoma cell lines (T1015A, T1185B, Me275, Me290) were provided as a kind gift by Prof. Daniel Speiser from the Department of Fundamental Oncology, University of Lausanne.

Authentication

Samples were authenticated by comparing the names labelled on the vials received with the providers' information. Molecular HLA typing was performed.

Mycoplasma contamination

Cells were tested negative for mycoplasma contamination

Commonly misidentified lines
(See ICLAC register)

No commonly misidentified lines were used

Human research participants

Policy information about studies involving human research participants

Population characteristics

This information can also be found in Supplementary Data 2 in the manuscript.

Sample Name	Type of sample	Gender	Stage/Grade
0D5P	Nodular melanoma	M	T:3b; N:0; M:1c; Stage IV
0MM745	Malignant melanoma	M	T: 1a; N: 0; M: 0; Stage unknown
0NVC	Superficial spreading melanoma	F	T:2a; N:3; M:0; Stage IIIC
Me275	Melanoma	M	NaN
Me290	Melanoma	F	NaN
T1015A	Melanoma	M	NaN
T1185B	Melanoma	F	NaN
C3N02289	LUSC	M	Grade 2
C3N02671	LUAD	F	Grade 2

Recruitment

Patient derived cell lines and tissues were already biobanked. We selected patient samples based on the availability of large sample material to be able to perform immunopeptidomics, or based on the successful establishment of patient-derived tumor cell lines. Overall, this should not have had any impact on the results obtained.

Ethics oversight

Informed consent of the participants was obtained following requirements of the institutional review board (Ethics Commission, CHUV, Bioethics Committee, Poznan University of Medical Sciences, Poznań, Poland).

Note that full information on the approval of the study protocol must also be provided in the manuscript.
